# Supplementary material for: What outcomes are associated with developing and implementing co-produced interventions in acute healthcare settings? A rapid evidence synthesis
Source: BMJ Open. 2017 Jul 11;7(7):e014650. doi: 10.1136/bmjopen-2016-014650 (PMC5734495; doi:10.1136/bmjopen-2016-014650)
Supplement: Supplementary material 1 [file bmjopen-2016-014650supp001.pdf]

Supplementary file 1 Search strategy (MEDLINE example)

Database: Ovid MEDLINE(R) <1996 to November Week 3 2015>

----

1 co-produc*.tw. (1149)

2 coproduc*.tw. (620)

3 co design*.tw. (53)

4 codesign*.tw. (16)

5 co creat*.tw. (171)

6 cocreat*.tw. (52)

7 (co research and design).tw. (1)

8 experience based design*.tw. (7)

9 participatory design*.tw. (149)

10 (experience adj3 design).tw. (252)

11 (evidence* adj2 design*).tw. (550)

12 EBC?.tw. (1604)

13 collaborative design.tw. (44)

14 human centred design.tw. (10)

15 human centered design.tw. (38)

16 people centred design.tw. (0)

17 people centered design.tw. (0)

18 inclusive design.tw. (32)

19 practice led design.tw. (1)

20 practice based design.tw. (2)

21 interactive design.tw. (31)

22 open design.tw. (147)

23 user centred design.tw. (51)

24 user centered design.tw. (177)

25 or/1-24 [co-design] (5093)

26 (acute adj (setting* or hospital* or care or healthcare)).tw. (15023)

27 ((secondary or speciali?ed) adj care).tw. (4410)

28 hospital care.tw. (4817)

29 exp Hospitals/ (117870)

30 Hospitalization/ (52132)

31 (hospitali?ation* or hospitali?ed).tw. (119506)

32 Inpatients/ (12602)

33 inpatient*.tw. (52413)

34 emergency medicine/ (8188)

35 Emergency treatment/ (8750)

36 emergency hospital admission*.tw. (240)

37 emergency hospitali#ation.tw. (116)

38 critical care/ (14911)

39 ambulatory care/ (20495)

40 urgent care.tw. (960)

41 or/26-40 [acute terms] (343100)

42 Delivery of Health Care/ (45257)

43 Efficiency, Organizational/ (17793)

44 Efficiency/ (4714)

45 Health Services Research/ (23584)

46 "Outcome Assessment (Health Care)"/ (50681)

47 patient outcome assessment/ (1713)

48 exp Program Evaluation/ (54529)

49 Quality Assurance, Health Care/ (38609)

50 Quality Improvement/ (9440)

51 Quality Indicators, Health Care/ (11744)

52 Quality of Health Care/ (43880)

53 (bench mark* or benchmark*).tw,kf. (17315)

54 (delivery adj2 health*).tw,kf. (12585)

55 (efficien* adj2 (assess* or assurance* or evaluat* or improv* or indicat* or measur* or test*)).tw,kf. (23714)

56 (performance adj2 (assess* or assurance* or evaluat* or improv* or indicat* or measur* or test*)).tw,kf. (74378)

57 (outcome* adj2 (assess* or assurance* or evaluat* or improv* or

indicat* or measur* or test*)).tw,kf. (256084)

58 (program* adj2 (assess* or assurance* or evaluat* or improv* or

indicat* or measur* or test*)).tw,kf. (27836)

59 or/42-58 [outcome or evaluation terms] (611767)

60 25 and 41 and 59 [co-design and acute care and outcome or evaluation terms] (45)

61 exp animals/ not humans.sh. (2031513)

62 60 not 61 [human only studies] (45)

63 limit 62 to (english language and yr="2005 - 2015") (40
