# Supplementary material for: What outcomes are associated with developing and implementing co-produced interventions in acute healthcare settings? A rapid evidence synthesis
Source: BMJ Open. 2017 Jul 11;7(7):e014650. doi: 10.1136/bmjopen-2016-014650 (PMC5734495; doi:10.1136/bmjopen-2016-014650)
Supplement: Supplementary material 3 [file bmjopen-2016-014650supp003.pdf]

**Supplementary file**

Quality assessment records: What outcomes are associated with developing and implementing co-produced interventions in acute healthcare settings? A rapid evidence synthesis. (Clarke,D.J., Jones, F., Harris, R., Robert, G. 2017)

Checklist CREATE Rapid evidence synthesis. Quality appraisal tool. The full guidance can be accessed at:

https://www.nice.org.uk/article/pmg4/chapter/1%20introduction

| | | |
|---|---|---|
| **Study identification:** Include author, title, reference, year of publication | Bowen et al, How was it for you? Experiences of participatory design in the UK health service. CoDesign, 9:4, 230-246, | |
| **Guidance topic:** | **Key research question/aim:**<br><br>The aim of the project being evaluated was: ' a 12-month service improvement project focused on the medical outpatient service for older people,.<br><br>The aim (of this publication) was to explore how the project reveals issues of wider relevance to participatory health service design and to suggest general tactics for dealing with them. | |
| **Checklist completed by:** | DJC | |
| **Theoretical approach** Co-design using EBD located within a wider discussion of participatory approaches in healthcare and the NHS. | | |
| **1. Is a qualitative approach appropriate?**<br><br>For example:<br><br>• Does the research question seek to understand processes or structures, or illuminate subjective experiences or meanings?<br><br>• Could a quantitative approach better have addressed the research question? | **Appropriate √**<br><br>Inappropriate<br><br>Not sure | Comments:<br><br>Appropriate method to address the main focus of the study which was on 'Experiences of participatory design in the UK health service' and used semi-structured interviews and a thematic approach to the analysis of the interview data. |

| | | |
|---|---|---|
| **2. Is the study clear in what it seeks to do?**<br><br>For example:<br><br>• Is the purpose of the study discussed – aims/objectives/research question/s?<br><br>• Is there adequate/appropriate reference to the literature?<br><br>• Are underpinning values/assumptions/theory discussed? | **Clear √**<br><br>Unclear<br><br>Mixed | Comments:<br><br>Although lacks specific aims and research questions the purpose and focus of the evaluation work is clear.<br><br>`the work is located in the wider literature on participatory health service design in general and on the use of the EBD method specifically'. This refers to underlying assumption related to participation, i.e. that it should be more than consultation and go beyond surveys and directly involve users' experiences in face to face co-design activity with staff. |
| **Study design** | | |
| **3. How defensible/rigorous is the research design/methodology?**<br><br>For example:<br><br>• Is the design appropriate to the research question?<br><br>• Is a rationale given for using a qualitative approach?<br><br>• Are there clear accounts of the rationale/justification for the sampling, data collection and data analysis techniques used?<br><br>• Is the selection of cases/sampling strategy theoretically justified? | **Defensible √**<br><br>Indefensible<br><br>Not sure | Comments:<br><br>Appropriate means of data collection and analysis. Small sample size and no specific justification for the qualitative design.<br><br>The sample reflects the participants in the main EBD project but is not otherwise discussed or justified. |
| **Data collection** | | |

| | | |
|---|---|---|
| **4. How well was the data collection carried out?**<br><br>For example:<br><br>• Are the data collection methods clearly described?<br><br>• Were the appropriate data collected to address the research question?<br><br>• Was the data collection and record keeping systematic? | **Appropriately √**<br><br>Inappropriately<br><br>Not sure/inadequately reported | Comments:<br><br>Methods were clearly described, it was clear who undertook data collection and how. Some indication is provided of the focus of the questions asked of participants but no actual questions are presented in the paper.<br><br>No specific comments are made re record keeping although the description of data management as part of analysis is clear and appropriate. |
| **Trustworthiness** | | |
| **5. Is the role of the researcher clearly described?**<br><br>For example:<br><br>• Has the relationship between the researcher and the participants been adequately considered?<br><br>• Does the paper describe how the research was explained and presented to the participants? | Clearly described<br><br>Unclear<br><br>**Not described √** | Comments:<br><br>Two researchers, who were not members of the original BOSOP project team, conducted the interviews.<br><br>The relationship between the researcher and the participants was not discussed.<br><br>How the research was explained and presented to the participants was not discussed. |
| **6. Is the context clearly described?**<br><br>For example:<br><br>• Are the characteristics of the participants and settings clearly defined?<br><br>• Were observations made in a sufficient variety of circumstances | **Clear √**<br><br>Unclear<br><br>Not sure | Comments:<br><br>Context bias was not considered. No observations were undertake, this was a small scale interview study.<br><br>The overall characteristics of the interview participants are outlined (e.g. patient or staff, if staff role type is stated)  Notes who declined to participate and that a £10 voucher was given to participants. |

| | | |
|---|---|---|
| • Was context bias considered | | |
| **7. Were the methods reliable?**<br><br>For example:<br><br>• Was data collected by more than 1 method?<br><br>• Is there justification for triangulation, or for not triangulating?<br><br>• Do the methods investigate what they claim to? | **Reliable √**<br><br>Unreliable<br><br>Not sure | Comments:<br><br>Only interviews were used in this study (n=11). Data to evaluate the original project is described, there is no specific consideration of data triangulation. The interviews are clearly focused on participants' experiences and address the overall research aim. |
| **Analysis** | | |
| **8. Is the data analysis sufficiently rigorous?**<br><br>For example:<br><br>• Is the procedure explicit – i.e. is it clear how the data was analysed to arrive at the results? (Yes)<br><br>• How systematic is the analysis, is the procedure reliable/dependable? (Yes)<br><br>• Is it clear how the themes and concepts were derived from the data? (yes) | **Rigorous √**<br><br>Not rigorous<br><br>Not sure/not reported | Comments: This is clearly outlined, is consistent with good practice in interview based qualitative research. The thematic analysis approach is clearly described, see across. |
| **9. Is the data 'rich'?**<br><br>For example:<br><br>• How well are the contexts of the data described? | **Rich √**<br><br>Poor<br><br>Not sure/not reported | Comments: A number of extracts from participants' interviews are reported and commented on in the discussion of the main themes emerging from the interview data analysis. Different views are explored although the sample size is limited so meaningful comparisons between participant views are vert limited. |

| | | |
|---|---|---|
| • Has the diversity of perspective and content been explored?<br>• How well has the detail and depth been demonstrated?<br>• Are responses compared and contrasted across groups/sites? | | |
| **10. Is the analysis reliable?**<br>For example:<br>• Did more than 1 researcher theme and code transcripts/data?<br>• If so, how were differences resolved?<br>• Did participants feed back on the transcripts/data if possible and relevant?<br>• Were negative/discrepant results addressed or ignored? | **Reliable √**<br>Unreliable<br>Not sure/not reported | Comments:<br>There appears to have been independent and joint researcher involvement in coding and thematic development. No participant feedback was sought on transcripts. As stated above the sample size was small so there appeared to be broad consensus amongst participants rather than negative/discrepant results, but 'negative' views were reported where they were identified. |
| **11. Are the findings convincing?**<br>For example:<br>• Are the findings clearly presented?<br>• Are the findings internally coherent?<br>• Are extracts from the original data included?<br>• Are the data appropriately referenced?<br>• Is the reporting clear and coherent? | **Convincing √**<br>Not convincing<br>Not sure | Comments:<br>Five themes were identified and discussed clearly and coherently. Original data extracts were included. The discussion section refers to linked and relevant literature in the field. |

| | | |
|---|---|---|
| **12. Are the findings relevant to the aims of the study?** | **Relevant √**<br><br>Irrelevant<br><br>Partially relevant | Comments: Focus directly on participants' experiences of the involvement in the EBD project. |
| **13. Conclusions**<br><br>For example:<br><br>• How clear are the links between data, interpretation and conclusions?<br><br>• Are the conclusions plausible and coherent?<br><br>• Have alternative explanations been explored and discounted?<br><br>• Does this enhance understanding of the research topic?<br><br>• Are the implications of the research clearly defined?<br><br>**Is there adequate discussion of any limitations encountered?** | **Adequate √**<br><br>Inadequate<br><br>Not sure | Comments:<br><br>Conclusions are clear, plausible and directly related to evaluation of the experience of the EBD method. The findings appear to have been contributory to further development of the EBD method which became EBCD though informing the Kings Fund resources/tool kit.<br><br>Limitations of the methods and the study were clearly identified in the discussion section and their impact of the findings considered. |
| **Ethics** | | |
| **14. How clear and coherent is the reporting of ethics?**<br><br>For example:<br><br>• Have ethical issues been taken into consideration?<br><br>• Are they adequately discussed e.g. do they | **Appropriate √**<br><br>Inappropriate<br><br>Not sure/not reported | Comments: Clear that ethical approval was obtained nut ethical issues were not discussed in the report other than that consent was obtained. |

| | | |
|---|---|---|
| address consent and anonymity?<br>• Have the consequences of the research been considered i.e. raising expectations, changing behaviour?<br>• Was the study approved by an ethics committee? | | |
| **Overall assessment** | | |
| **As far as can be ascertained from the paper, how well was the study conducted? (see guidance notes)** | **++**<br>+<br>− | Comments: Small descriptive study but clearly presented overall with core areas of the research e.g. data collection and analysis clearly reported and appropriate to the study focus. This statement applies: '*All or most of the checklist criteria have been fulfilled, where they have not been fulfilled the conclusions are very unlikely to alter'*. |

| | |
|---|---|
| **Study identification:** Include author, title, reference, year of publication | Bowen et al, How was it for you? Experiences of participatory design in the UK health service. CoDesign, 9:4, 230-246, |
| **Guidance topic:** Co-production in acute healthcare settings. | **Key research question/aim:** The authors were concerned that there had been only limited tangible service improvement at the end of the project BOSOP project so they designed a post-project evaluation study to test their previous hypotheses about why this might be the case against the viewpoints of other stakeholders within the EBD project and to refine understanding. |
| **Checklist completed by:** | RH |
| **Theoretical approach:** No specific theoretical framework was reported | |

| | | |
|---|---|---|
| **1. Is a qualitative approach appropriate?**<br>For example:<br>• Does the research question seek to understand processes or structures, or illuminate subjective experiences or meanings?<br>• Could a quantitative approach better have addressed the research question? | Appropriate ✓<br>Inappropriate<br>Not sure | Comments: |
| **2. Is the study clear in what it seeks to do?**<br>For example:<br>• Is the purpose of the study discussed – aims/objectives/research question/s?<br>• Is there adequate/appropriate reference to the literature?<br>• Are underpinning values/assumptions/theory discussed? | Clear ✓<br>Unclear<br>Mixed | Comments: |
| **Study design** | | |
| **3. How defensible/rigorous is the research design/methodology?**<br>For example:<br>• Is the design appropriate to the research question?<br>• Is a rationale given for using a qualitative approach?<br>• Are there clear accounts of the rationale/justification for the sampling, data collection and data analysis techniques | Defensible ✓<br>Indefensible<br>Not sure | Comments: |

| | | |
|---|---|---|
| used?<br>• Is the selection of cases/sampling strategy theoretically justified? | | |
| **Data collection** | | |
| **4. How well was the data collection carried out?**<br>For example:<br>• Are the data collection methods clearly described?<br>• Were the appropriate data collected to address the research question?<br>• Was the data collection and record keeping systematic? | Appropriately ✓<br>Inappropriately<br>Not sure/inadequately reported | Comments: |
| **Trustworthiness** | | |

| | | |
|---|---|---|
| **5. Is the role of the researcher clearly described?**<br>For example:<br>• Has the relationship between the researcher and the participants been adequately considered?<br>• Does the paper describe how the research was explained and presented to the participants? | Clearly described ✓<br>Unclear<br>Not described | Comments: |
| **6. Is the context clearly described?**<br>For example:<br>• Are the characteristics of the participants and settings clearly defined?<br>• Were observations made in a sufficient variety of circumstances<br>• Was context bias considered | Clear ✓<br>Unclear<br>Not sure | Comments: |
| **7. Were the methods reliable?**<br>For example:<br>• Was data collected by more than 1 method?<br>• Is there justification for triangulation, or for not triangulating? | Reliable ✓<br>Unreliable<br>Not sure | Comments: |

| | | |
|---|---|---|
| • Do the methods investigate what they claim to? | | |
| **Analysis** | | |
| **8. Is the data analysis sufficiently rigorous?**<br>For example:<br>• Is the procedure explicit – i.e. is it clear how the data was analysed to arrive at the results?<br>• How systematic is the analysis, is the procedure reliable/dependable?<br>• Is it clear how the themes and concepts were derived from the data? | Rigorous<br>Not rigorous ✓<br>Not sure/not reported | Comments: |
| **9. Is the data 'rich'?**<br>For example:<br>• How well are the contexts of the data described?<br>• Has the diversity of perspective and content been explored?<br>• How well has the detail and depth been demonstrated?<br>• Are responses compared and contrasted across groups/sites? | Rich ✓<br>Poor<br>Not sure/not reported | Comments: |
| **10. Is the analysis reliable?** | Reliable ✓ | Comments: |

| | | |
|---|---|---|
| For example:<br>• Did more than 1 researcher theme and code transcripts/data?<br>• If so, how were differences resolved?<br>• Did participants feed back on the transcripts/data if possible and relevant?<br>• Were negative/discrepant results addressed or ignored? | Unreliable<br>Not sure/not reported | |
| **11. Are the findings convincing?**<br>For example:<br>• Are the findings clearly presented?<br>• Are the findings internally coherent?<br>• Are extracts from the original data included?<br>• Are the data appropriately referenced?<br>• Is the reporting clear and coherent? | Convincing ✓<br>Not convincing<br>Not sure | Comments: |
| **12. Are the findings relevant to the aims of the study?** | Relevant ✓<br>Irrelevant<br>Partially relevant | Comments: |
| **13. Conclusions**<br>For example:<br>• How clear are the links between data, interpretation and | Adequate ✓<br>Inadequate<br>Not sure | Comments: |

| | | |
|---|---|---|
| conclusions?<br>• Are the conclusions plausible and coherent?<br>• Have alternative explanations been explored and discounted?<br>• Does this enhance understanding of the research topic?<br>• Are the implications of the research clearly defined?<br>**Is there adequate discussion of any limitations encountered?** | | |
| **Ethics** | | |
| **14. How clear and coherent is the reporting of ethics?**<br>For example:<br>• Have ethical issues been taken into consideration?<br>• Are they adequately discussed e.g. do they address consent and anonymity?<br>• Have the consequences of the research been considered i.e. raising expectations, changing behaviour?<br>• Was the study approved by an ethics committee? | Appropriate ✓<br>Inappropriate<br>Not sure/not reported | Comments: |
| **Overall assessment** | | |
| **As far as can be ascertained from the paper, how well was the study conducted? (see guidance notes)** | ++ ✓ | Comments: |

| | | |
|---|---|---|
| | +<br>- | |

| | | |
|---|---|---|
| **Study identification:** Include author, title, reference, year of publication | Boyd et al, Improving healthcare through the use of co-design. The New Zealand Medical Journal, 125,76–87. | |
| **Guidance topic:** | **Key research question/aim:** No research questions. Aims stated only as follows: A Patient Co-Design of Breast Service Project was set up to improve referral processes and develop clinical guidelines for patients with breast cancer. It aimed to use co-design methods to understand patients experiences, make small services changes (not defined), make recommendations for further service change and develop a model for working with patients that could be used in other services. It also sought to work with patients in a new and more participatory way . | |
| **Checklist completed by:** | DJC | |
| **Theoretical approach** Locates the service improvement project in the broad area of co-design (with reference to co-production) and describes a project using EBD based methods but with six elements as opposed to the four seen for example in Bowen et al. Describes a clear commitment to the equity (in participating in service improvement based on experiences of patients), and understanding and improving patient experiences. | | |
| **1. Is a qualitative approach appropriate?**<br>For example:<br>• Does the research question seek to understand processes or structures, or illuminate subjective experiences or meanings?<br>• Could a quantitative approach better have | **Appropriate √**<br>Inappropriate<br>Not sure | Comments: Qualitative methods are used as part of the service improvement approach, rather than as research methods.<br>A simple quantitative survey tool was also employed. |

| | | |
|---|---|---|
| addressed the research question? | | |
| **2. Is the study clear in what it seeks to do?**<br><br>For example:<br><br>• Is the purpose of the study discussed – aims/objectives/research question/s?<br><br>• Is there adequate/appropriate reference to the literature?<br><br>• Are underpinning values/assumptions/theory discussed? | **Clear √**<br><br>Unclear<br><br>Mixed | Comments: Aims are only broadly outlined but the intentions of the project and desired impact on services to improve patient experiences were clear. |
| **Study design** | | |
| **3. How defensible/rigorous is the research design/methodology?**<br><br>For example:<br><br>• Is the design appropriate to the research question?<br><br>• Is a rationale given for using a qualitative approach?<br><br>• Are there clear accounts of the rationale/justification for the sampling, data collection and data analysis techniques used?<br><br>• Is the selection of cases/sampling strategy theoretically justified? | **Defensible √**<br><br>Indefensible<br><br>Not sure | Comments: In the sense that this was a structured service improvement approach based on an established approach EBD. No research questions were developed as such but there were questions driving the co-design workshops focused on patient experience.<br><br>There is no justification for sampling as such but there is for the patient journey mapping and the co-design workshops. |

| | | |
|---|---|---|
| **Data collection** | | |
| **4. How well was the data collection carried out?**<br>For example:<br>• Are the data collection methods clearly described?<br>• Were the appropriate data collected to address the research question?<br>• Was the data collection and record keeping systematic? | Appropriately<br>Inappropriately<br>Not sure/**inadequately reported √** | Comments: The methods were appropriate and relevant to the intended project outcomes but were reported in only a limited way. |
| **Trustworthiness** | | |
| **5. Is the role of the researcher clearly described?**<br>For example:<br>• Has the relationship between the researcher and the participants been adequately considered?<br>• Does the paper describe how the research was explained and presented to the participants? | Clearly described<br>Unclear<br>**Not described √** | Comments: The project as a whole is described but this refers to a number of facilitators and not to a single or small group of researchers as such. |
| **6. Is the context clearly described?**<br>For example:<br>• Are the characteristics of the participants and | Clear<br>Unclear<br>**Not sure √** | Comments: There is again, broad overview of the services in a particular health district. The ages of women attending, the backgrounds, referral systems, treatment times etc are not referred to. |

| | | |
|---|---|---|
| settings clearly defined?<br>• Were observations made in a sufficient variety of circumstances<br>• Was context bias considered | | |
| **7. Were the methods reliable?**<br>For example:<br>• Was data collected by more than 1 method?<br>• Is there justification for triangulation, or for not triangulating?<br>• Do the methods investigate what they claim to? | **Reliable √**<br>Unreliable<br>Not sure | Comments: Adequate description of methods appropriate to address the aims of the project. |
| **Analysis** | | |
| **8. Is the data analysis sufficiently rigorous?**<br>For example:<br>• Is the procedure explicit – i.e. is it clear how the data was analysed to arrive at the results?<br>• How systematic is the analysis, is the procedure reliable/dependable?<br>• Is it clear how the themes and concepts were derived from the data? | Rigorous<br>Not rigorous<br>Not sure**/not reported √** | Comments: no comment on this important issue. |

| | | |
|---|---|---|
| **9. Is the data 'rich'?**<br><br>For example:<br><br>• How well are the contexts of the data described?<br><br>• Has the diversity of perspective and content been explored?<br><br>• How well has the detail and depth been demonstrated?<br><br>• Are responses compared and contrasted across groups/sites? | Rich<br><br>Poor<br><br>Not sure/**not reported fully √** | Comments: There is descriptive summary in each of the three areas of data collected but no illustrative direct quotations from participants or staff. |
| **10. Is the analysis reliable?**<br><br>For example:<br><br>• Did more than 1 researcher theme and code transcripts/data?<br><br>• If so, how were differences resolved?<br><br>• Did participants feed back on the transcripts/data if possible and relevant?<br><br>• Were negative/discrepant results addressed or ignored? | Reliable<br><br>Unreliable<br><br>Not sure/**not reported √** | Comments: As above, no comment. |
| **11. Are the findings convincing?**<br><br>For example:<br><br>• Are the findings clearly presented? | **Convincing √**<br><br>Not convincing<br><br>Not sure | Comments: Accepting the limitations in reporting, the findings are convincing. |

| | | |
|---|---|---|
| • Are the findings internally coherent?<br>• Are extracts from the original data included?<br>• Are the data appropriately referenced?<br>• Is the reporting clear and coherent? | | |
| **12. Are the findings relevant to the aims of the study?** | **Relevant √**<br><br>Irrelevant<br><br>Partially relevant | Comments: |
| **13. Conclusions**<br><br>For example:<br>• How clear are the links between data, interpretation and conclusions?<br>• Are the conclusions plausible and coherent?<br>• Have alternative explanations been explored and discounted?<br>• Does this enhance understanding of the research topic?<br>• Are the implications of the research clearly defined?<br><br>**Is there adequate discussion of any limitations encountered?** | Adequate<br><br>**Inadequate √**<br><br>Not sure | Comments: There is a discussion section with recommendations but no conclusion section as such. The discussion does add to this specific literature (limited as it is) and there are indications for further research. The limitations of the study itself are acknowledged, i.e no evaluation and no follow up to determine whether planned improvements had been implemented and sustained. |
| **Ethics** | | |

| | | |
|---|---|---|
| **14. How clear and coherent is the reporting of ethics?**<br><br>For example:<br><br>• Have ethical issues been taken into consideration?<br><br>• Are they adequately discussed e.g. do they address consent and anonymity?<br><br>• Have the consequences of the research been considered i.e. raising expectations, changing behaviour?<br><br>• Was the study approved by an ethics committee? | Appropriate<br><br>Inappropriate<br><br>Not sure/**not reported √** | Comments: No indication that this was required if this was regarded as a service improvement project. |
| **Overall assessment** | | |
| **As far as can be ascertained from the paper, how well was the study conducted? (see guidance notes)** | ++<br><br>**+ √**<br><br>− | Comments: A very limited report; largely a service improvement report. |

| | |
|---|---|
| **Study identification:** Include author, title, reference, year of publication | Boyd et al, Improving healthcare through the use of co-design. The New Zealand Medical Journal, 125,76–87. |
| **Guidance topic:** | **Key research question/aim:** No research questions. Aims: A Patient Co-Design of Breast Service Project was set up to improve referral processes and develop clinical guidelines for patients with breast cancer. It aimed to use co-design methods to understand patients' experiences, make small services changes, make recommendations for further service change and develop a model for working with patients that could be |

| | | |
|---|---|---|
| | used in other services. It also sought to work with patients in a new and more participatory way. | |
| **Checklist completed by:** | FJ | |
| **Theoretical approach** Locates the service improvement project in the broad area of co-design (with reference to co-production) and describes a project using EBD based methods but with six elements as opposed to the four seen for example in Bowen et al. | | |
| **1. Is a qualitative approach appropriate?**<br>For example:<br>• Does the research question seek to understand processes or structures, or illuminate subjective experiences or meanings?<br>• Could a quantitative approach better have addressed the research question? | Appropriate **√**<br>Inappropriate<br>Not sure | Comments: |
| **2. Is the study clear in what it seeks to do?**<br>For example:<br>• Is the purpose of the study discussed – aims/objectives/research question/s?<br>• Is there adequate/appropriate reference to the literature?<br>• Are underpinning values/assumptions/theory discussed? | Clear **√**<br>Unclear<br>Mixed | Comments: |

| **Study design** | | |
|---|---|---|
| **3. How defensible/rigorous is the research design/methodology?**<br><br>For example:<br><br>• Is the design appropriate to the research question?<br><br>• Is a rationale given for using a qualitative approach?<br><br>• Are there clear accounts of the rationale/justification for the sampling, data collection and data analysis techniques used?<br><br>• Is the selection of cases/sampling strategy theoretically justified? | Defensible **√**<br><br>Indefensible<br><br>Not sure | Comments: |
| **Data collection** | | |
| **4. How well was the data collection carried out?**<br><br>For example:<br><br>• Are the data collection methods clearly described?<br><br>• Were the appropriate data collected to address the research question?<br><br>• Was the data collection and record keeping systematic? | Appropriately<br><br>Inappropriately<br><br>Not sure/inadequately reported **√** | Comments: |

| Trustworthiness | | |
|---|---|---|
| **5. Is the role of the researcher clearly described?**<br><br>For example:<br><br>• Has the relationship between the researcher and the participants been adequately considered?<br><br>• Does the paper describe how the research was explained and presented to the participants? | Clearly described<br><br>Unclear<br><br>Not described **√** | Comments: |
| **6. Is the context clearly described?**<br><br>For example:<br><br>• Are the characteristics of the participants and settings clearly defined?<br><br>• Were observations made in a sufficient variety of circumstances<br><br>• Was context bias considered | Clear<br><br>Unclear<br><br>Not sure **√** | Comments: |
| **7. Were the methods reliable?**<br><br>For example:<br><br>• Was data collected by more than 1 method?<br><br>• Is there justification for triangulation, or for not triangulating?<br><br>• Do the methods investigate what they claim | Reliable **√**<br><br>Unreliable<br><br>Not sure | Comments: |

| | | |
|---|---|---|
| to? | | |
| **Analysis** | | |
| **8. Is the data analysis sufficiently rigorous?**<br><br>For example:<br><br>• Is the procedure explicit – i.e. is it clear how the data was analysed to arrive at the results?<br><br>• How systematic is the analysis, is the procedure reliable/dependable?<br><br>• Is it clear how the themes and concepts were derived from the data? | Rigorous<br><br>Not rigorous<br><br>Not sure/not reported **√** | Comments: |
| **9. Is the data 'rich'?**<br><br>For example:<br><br>• How well are the contexts of the data described?<br><br>• Has the diversity of perspective and content been explored?<br><br>• How well has the detail and depth been demonstrated?<br><br>• Are responses compared and contrasted across groups/sites? | Rich<br><br>Poor<br><br>Not sure/not reported fully **√** | Comments: |
| **10. Is the analysis reliable?** | Reliable | Comments: |

| | | |
|---|---|---|
| For example:<br>• Did more than 1 researcher theme and code transcripts/data?<br>• If so, how were differences resolved?<br>• Did participants feed back on the transcripts/data if possible and relevant?<br>• Were negative/discrepant results addressed or ignored? | Unreliable<br>Not sure/not reported **√** | |
| **11. Are the findings convincing?**<br>For example:<br>• Are the findings clearly presented?<br>• Are the findings internally coherent?<br>• Are extracts from the original data included?<br>• Are the data appropriately referenced?<br>• Is the reporting clear and coherent? | Convincing **√**<br>Not convincing<br>Not sure | Comments: |
| **12. Are the findings relevant to the aims of the study?** | Relevant **√**<br>Irrelevant<br>Partially relevant | Comments: |
| **13. Conclusions**<br>For example: | Adequate<br>Inadequate **√** | Comments: |

| | | |
|---|---|---|
| • How clear are the links between data, interpretation and conclusions?<br>• Are the conclusions plausible and coherent?<br>• Have alternative explanations been explored and discounted?<br>• Does this enhance understanding of the research topic?<br>• Are the implications of the research clearly defined?<br>**Is there adequate discussion of any limitations encountered?** | Not sure | |
| **Ethics** | | |
| **14. How clear and coherent is the reporting of ethics?**<br>For example:<br>• Have ethical issues been taken into consideration?<br>• Are they adequately discussed e.g. do they address consent and anonymity?<br>• Have the consequences of the research been considered i.e. raising expectations, changing behaviour?<br>• Was the study approved by an ethics committee? | Appropriate<br>Inappropriate<br>Not sure/not reported **√** | Comments: |

| **Overall assessment** | | |
|---|---|---|
| **As far as can be ascertained from the paper, how well was the study conducted? (see guidance notes)** | ++<br>**+ √**<br>− | Comments: The methods were appropriate and relevant to the intended project outcomes. The paper is limited by omissions in a number of the areas indicated above, principally, little information on data analysis and the reporting of findings. |

| | | |
|---|---|---|
| **Study identification:** Include author, title, reference, year of publication | Golden.B.R. et al, 2011 Improving the patient experience through design. Healthcare Quarterly. 14(3)32-41 | |
| **Guidance topic:** Co-production in acute healthcare settings. | **Key research question/aim:** PMH's vision was to create a new space and experience that was truly patient centric and world class. (P32) No specific aims and objectives were stated. | |
| **Checklist completed by:** | FJ | |
| **Theoretical approach:**<br>Reference to ethnography and 'Rotman Creative design process | | |
| **1. Is a qualitative approach appropriate?**<br>For example:<br>• Does the research question seek to understand processes or structures, or illuminate subjective experiences or meanings?<br>• Could a quantitative approach better have | Appropriate ✓<br>Inappropriate<br>Not sure | Comments:<br>The study report different qualitative methods used to inform, develop and explore service experience in an outpatient cancer setting. Its is not a qualitative study as such. |

| | | |
|---|---|---|
| addressed the research question? | | |
| **2. Is the study clear in what it seeks to do?**<br>For example:<br>• Is the purpose of the study discussed – aims/objectives/research question/s?<br>• Is there adequate/appropriate reference to the literature?<br>• Are underpinning values/assumptions/theory discussed? | Clear<br>Unclear<br>Mixed ✓ | Comments:<br>There are no aims but the purpose is clear to improve the experience of patients. Minimal reference to relevant literature, reads more like a commentary/report than reporting the findings from a distinct study. |
| **Study design** | | |
| **3. How defensible/rigorous is the research design/methodology?**<br>For example:<br>• Is the design appropriate to the research question?<br>• Is a rationale given for using a qualitative approach?<br>• Are there clear accounts of the rationale/justification for the sampling, data collection and data analysis techniques used?<br>• Is the selection of cases/sampling strategy | Defensible<br>Indefensible<br>Not sure ✓ | Comments:<br>The paper reports the 3 stages of Rotman Creative design, the first 2 stages include multiple methods. But these are not clearly linked to any research question. Many of the data collection methods involved researcher led activities, which followed a series of stages without full rationale and justification.<br>There was no evidence of sampling and inadequate detail about cases included. |

| | | |
|---|---|---|
| theoretically justified? | | |
| **Data collection** | | |
| **4. How well was the data collection carried out?**<br>For example:<br>• Are the data collection methods clearly described?<br>• Were the appropriate data collected to address the research question?<br>• Was the data collection and record keeping systematic? | Appropriately<br>Inappropriately<br>Not sure/<br>inadequately reported ✓ | Comments:<br>Data collection methods briefly described but not in a systematic or logical way. No detail about how data was extracted, recorded etc |
| **Trustworthiness** | | |

| | | |
|---|---|---|
| **5. Is the role of the researcher clearly described?**<br>For example:<br>• Has the relationship between the researcher and the participants been adequately considered?<br>• Does the paper describe how the research was explained and presented to the participants? | Clearly described<br>Unclear<br>Not described ✓ | Comments: |
| **6. Is the context clearly described?**<br>For example:<br>• Are the characteristics of the participants and settings clearly defined?<br>• Were observations made in a sufficient variety of circumstances<br>• Was context bias considered | Clear ✓<br>Unclear<br>Not sure | Comments:<br>There were 'patient personas' described which were supposed to represent patients using the services, no other details<br>The setting was described but not in any great detail, more to illustrate particular problems, issues. |
| **7. Were the methods reliable?**<br>For example:<br>• Was data collected by more than 1 method?<br>• Is there justification for triangulation, or for not triangulating? | Reliable<br>Unreliable<br>Not sure ✓ | Comments:<br>Not reported; could be triangulation as more than one method was used but this was not reported as such. |

| | | |
|---|---|---|
| • Do the methods investigate what they claim to? | | |
| **Analysis** | | |
| **8. Is the data analysis sufficiently rigorous?**<br>For example:<br>• Is the procedure explicit – i.e. is it clear how the data was analysed to arrive at the results?<br>• How systematic is the analysis, is the procedure reliable/dependable?<br>• Is it clear how the themes and concepts were derived from the data? | Rigorous<br>Not rigorous<br>Not sure/ ✓ | Comments:<br>Inconsistent and inadequate reporting of data analysis methods<br>For example the interview data was subjected to line by line coding and then synthesised into a mind map. The method of analysis and approach used was not stated. |
| **9. Is the data 'rich'?**<br>For example:<br>• How well are the contexts of the data described?<br>• Has the diversity of perspective and content been explored?<br>• How well has the detail and depth been demonstrated?<br>• Are responses compared and contrasted | Rich<br>Poor<br>Not sure✓ | Comments:<br>Quotes were given from patients involved in photo journaling; need mind map illustrated the diversity and context of the project. But the findings weren't presented systematically and preferential reporting of certain experiences/comments couldn't be ruled out. The diversity of perspective presented by authors is hard to appraise. |

| | | |
|---|---|---|
| across groups/sites? | | |
| **10. Is the analysis reliable?**<br>For example:<br>• Did more than 1 researcher theme and code transcripts/data?<br>• If so, how were differences resolved?<br>• Did participants feed back on the transcripts/data if possible and relevant?<br>• Were negative/discrepant results addressed or ignored? | Reliable<br>Unreliable<br>Not sure/not reported ✓ | Comments: |
| **11. Are the findings convincing?**<br>For example:<br>• Are the findings clearly presented?<br>• Are the findings internally coherent?<br>• Are extracts from the original data included?<br>• Are the data appropriately referenced?<br>• Is the reporting clear and coherent? | Convincing<br>Not convincing<br>Not sure ✓ | Comments: The data findings were not consistently reported. There was more detail about some stages than other in sections. Some extracts from original data included, but this was also inconsistent. |
| **12. Are the findings relevant to the aims of the study?** | Relevant ✓<br>Irrelevant | Comments: |

| | | |
|---|---|---|
| | Partially relevant | |
| **13. Conclusions**<br>For example:<br>• How clear are the links between data, interpretation and conclusions?<br>• Are the conclusions plausible and coherent?<br>• Have alternative explanations been explored and discounted?<br>• Does this enhance understanding of the research topic?<br>• Are the implications of the research clearly defined?<br>**Is there adequate discussion of any limitations encountered?** | Adequate<br>Inadequate<br>Not sure ✓ | Comments:<br>The aims were not fully stated therefore it is not clear if the conclusions are accurate.<br>The paper provides an in-depth description of a staged design approach but the lack of clarity and way in which the findings are reported makes it difficult to confirm if the findings are plausible |
| **Ethics** | | |
| **14. How clear and coherent is the reporting of ethics?**<br>For example:<br>• Have ethical issues been taken into consideration?<br>• Are they adequately discussed e.g. do they | Appropriate<br>Inappropriate<br>Not sure/not reported ✓ | Comments:<br>Not reported |

| | | |
|---|---|---|
| address consent and anonymity?<br>• Have the consequences of the research been considered i.e. raising expectations, changing behaviour?<br>• Was the study approved by an ethics committee? | | |
| **Overall assessment** | | |
| **As far as can be ascertained from the paper, how well was the study conducted? (see guidance notes)** | ++<br>**+** ✓<br>- | Comments: |

| | |
|---|---|
| **Study identification:** Include author, title, reference, year of publication | Golden.B.R. et al, 2011 Improving the patient experience through design. Healthcare Quarterly. 14(3)32-41 |
| **Guidance topic:** Co-production in acute healthcare settings. | **Key research question/aim:** PMH's vision was to create a new space and experience that was truly patient centric and world class. (P32) No specific aims and objectives were stated. |
| **Checklist completed by:** | RH |
| **Theoretical approach:** No specific theoretical framework was reportedly used although the paper does refer to ethnography. The Rotman Creative Design process was used. This involved 3 main activities: Gear 1. empathy and deep human understanding; Gear 2. Concept visualisation; Gear 3. Strategic business design. | |

| | | |
|---|---|---|
| **1. Is a qualitative approach appropriate?**<br>For example:<br>• Does the research question seek to understand processes or structures, or illuminate subjective experiences or meanings?<br>• Could a quantitative approach better have addressed the research question? | Appropriate ✓<br>Inappropriate<br>Not sure | Comments: The paper does not report a research study as such. It reports the first stages of a co-design process but the later stages where a specific intervention is designed and evaluated is not reported. |
| **2. Is the study clear in what it seeks to do?**<br>For example:<br>• Is the purpose of the study discussed – aims/objectives/research question/s?<br>• Is there adequate/appropriate reference to the literature?<br>• Are underpinning values/assumptions/theory discussed? | Clear<br>Unclear<br>Mixed ✓ | Comments: The aims are not specifically stated although from reading the paper it is clear what they are attempting to do. The researchers do cite literature about the process of co-design they are using but the study feels half finished! |
| **Study design** | | |
| **3. How defensible/rigorous is the research design/methodology?**<br>For example:<br>• Is the design appropriate to the research question? | Defensible<br>Indefensible<br>Not sure ✓ | Comments: The approach used is defensible however, there is a lack of details about how participants were selected, no. of participants / observation sessions etc. |

| | | |
|---|---|---|
| • Is a rationale given for using a qualitative approach?<br>• Are there clear accounts of the rationale/justification for the sampling, data collection and data analysis techniques used?<br>• Is the selection of cases/sampling strategy theoretically justified? | | |
| **Data collection** | | |
| **4. How well was the data collection carried out?**<br>For example:<br>• Are the data collection methods clearly described?<br>• Were the appropriate data collected to address the research question?<br>• Was the data collection and record keeping systematic? | Appropriately<br>Inappropriately<br>Not sure/**inadequately reported** ✓ | Comments: There was quite a few methods used and how these worked together is not reported. There is a lack of detail about how individual methods as well. |
| **Trustworthiness** | | |

| | | |
|---|---|---|
| **5. Is the role of the researcher clearly described?**<br>For example:<br>• Has the relationship between the researcher and the participants been adequately considered?<br>• Does the paper describe how the research was explained and presented to the participants? | Clearly described<br>Unclear<br>Not described ✓ | Comments: |
| **6. Is the context clearly described?**<br>For example:<br>• Are the characteristics of the participants and settings clearly defined?<br>• Were observations made in a sufficient variety of circumstances<br>• Was context bias considered | Clear ✓<br>Unclear<br>Not sure | Comments: Characteristics of some patients were reported in detail but not all, particularly carers. Observations were made in a variety of circumstances although context bias was not considered in the paper. |
| **7. Were the methods reliable?**<br>For example:<br>• Was data collected by more than 1 method?<br>• Is there justification for triangulation, or for not triangulating? | Reliable ✓<br>Unreliable<br>Not sure | Comments: Not reported as such although there were data from a range of methods. |

| | | |
|---|---|---|
| • Do the methods investigate what they claim to? | | |
| **Analysis** | | |
| **8. Is the data analysis sufficiently rigorous?**<br>For example:<br>• Is the procedure explicit – i.e. is it clear how the data was analysed to arrive at the results?<br>• How systematic is the analysis, is the procedure reliable/dependable?<br>• Is it clear how the themes and concepts were derived from the data? | Rigorous<br>Not rigorous<br>Not sure/**not reported** ✓ | Comments: Analysis not reported in sufficient detail |
| **9. Is the data 'rich'?**<br>For example:<br>• How well are the contexts of the data described?<br>• Has the diversity of perspective and content been explored?<br>• How well has the detail and depth been demonstrated?<br>• Are responses compared and contrasted across groups/sites? | Rich ✓<br>Poor<br>Not sure/not reported | Comments: The data as reported is rich although whether data analysis is systematic and comprehensive is not reported. |

| | | |
|---|---|---|
| **10. Is the analysis reliable?**<br>For example:<br>• Did more than 1 researcher theme and code transcripts/data?<br>• If so, how were differences resolved?<br>• Did participants feed back on the transcripts/data if possible and relevant?<br>• Were negative/discrepant results addressed or ignored? | Reliable<br>Unreliable<br>Not sure/not reported ✓ | Comments: How data were analysed is not reported. |
| **11. Are the findings convincing?**<br>For example:<br>• Are the findings clearly presented?<br>• Are the findings internally coherent?<br>• Are extracts from the original data included?<br>• Are the data appropriately referenced?<br>• Is the reporting clear and coherent? | Convincing ✓<br>Not convincing<br>Not sure | Comments: This findings were quite compelling although not presented in a form that was easy to follow. |
| **12. Are the findings relevant to the aims of the study?** | Relevant ✓<br>Irrelevant<br>Partially relevant | Comments: although the study is not complete – the design and implementation of an intervention is not reported. |

| | | |
|---|---|---|
| **13. Conclusions**<br>For example:<br>• How clear are the links between data, interpretation and conclusions?<br>• Are the conclusions plausible and coherent?<br>• Have alternative explanations been explored and discounted?<br>• Does this enhance understanding of the research topic?<br>• Are the implications of the research clearly defined?<br>**Is there adequate discussion of any limitations encountered?** | Adequate<br>Inadequate<br>Not sure ✓ | Comments: The aims are not fulfilled as reported in this paper as the intervention developed is not reported. Feels unfinished. There is useful, interesting findings though. And the value of the co-design approach is demonstrated. |
| **Ethics** | | |
| **14. How clear and coherent is the reporting of ethics?**<br>For example:<br>• Have ethical issues been taken into consideration?<br>• Are they adequately discussed e.g. do they address consent and anonymity?<br>• Have the consequences of the research been | Appropriate<br>Inappropriate<br>Not sure/not reported ✓ | Comments: Not addressed in the paper |

| | | |
|---|---|---|
| considered i.e. raising expectations, changing behaviour?<br>• Was the study approved by an ethics committee? | | |
| **Overall assessment** | | |
| **As far as can be ascertained from the paper, how well was the study conducted? (see guidance notes)** | ++<br>**+** ✓<br>− | Comments: |

| | | |
|---|---|---|
| **Study identification:** Include author, title, reference, year of publication | Larkin, M et al, 2015 On the Brink of Genuinely Collaborative Care: Experience-Based Co-Design in Mental Health. Qualitative Health Research2015, Vol. 25(11) 1463–1476 | |
| **Guidance topic:** Co-production in acute healthcare settings. | **Key research question/aim:**<br><br>Can 3 qualitative studies and an adapted form of EBCD translate into service improvements in acute mental health services | |
| **Checklist completed by:** | FJ | |
| **Theoretical approach: evaluation of existing EBCD improvement projects; no other details about theoretical approach** | | |
| **1. Is a qualitative approach appropriate?** | Appropriate ✓ | Comments: interviews used to inform co-design process |

| | | |
|---|---|---|
| For example:<br><br>• Does the research question seek to understand processes or structures, or illuminate subjective experiences or meanings?<br><br>• Could a quantitative approach better have addressed the research question? | Inappropriate<br><br>Not sure | |
| **2. Is the study clear in what it seeks to do?**<br><br>For example:<br><br>• Is the purpose of the study discussed – aims/objectives/research question/s?<br><br>• Is there adequate/appropriate reference to the literature?<br><br>• Are underpinning values/assumptions/theory discussed? | Clear ✓<br><br>Unclear<br><br>Mixed | Comments: |
| **Study design** | | |
| **3. How defensible/rigorous is the research design/methodology?**<br><br>For example:<br><br>• Is the design appropriate to the research question?<br><br>• Is a rationale given for using a qualitative | Defensible ✓<br><br>Indefensible<br><br>Not sure | Comments: |

| | | |
|---|---|---|
| approach?<br><br>• Are there clear accounts of the rationale/justification for the sampling, data collection and data analysis techniques used?<br><br>• Is the selection of cases/sampling strategy theoretically justified? | | |
| **Data collection** | | |
| **4. How well was the data collection carried out?**<br><br>For example:<br><br>• Are the data collection methods clearly described?<br><br>• Were the appropriate data collected to address the research question?<br><br>• Was the data collection and record keeping systematic? | Appropriately ✓<br><br>Inappropriately<br><br>Not sure/inadequately reported | Comments: full findings of interview analyses are reported elsewhere |
| **Trustworthiness** | | |

| | | |
|---|---|---|
| **5. Is the role of the researcher clearly described?**<br>For example:<br>• Has the relationship between the researcher and the participants been adequately considered?<br>• Does the paper describe how the research was explained and presented to the participants? | Clearly described<br>Unclear ✓<br>Not described | Comments: role and activities of researcher not explicitly stated but inferred from descriptions of researchers involvement in co-design process |
| **6. Is the context clearly described?**<br>For example:<br>• Are the characteristics of the participants and settings clearly defined?<br>• Were observations made in a sufficient variety of circumstances<br>• Was context bias considered | Clear<br>Unclear ✓<br>Not sure | Comments: no comment on content bias, no observations done; characteristics of participants not described ( ? this is due to the fact that they are reported in another publication) |
| **7. Were the methods reliable?**<br>For example:<br>• Was data collected by more than 1 method?<br>• Is there justification for triangulation, or for not triangulating? | Reliable ✓<br>Unreliable<br>Not sure | Comments: interviews only but triangulated through feedback groups and co-design groups |

| | | |
|---|---|---|
| • Do the methods investigate what they claim to? | | |
| **Analysis** | | |
| **8. Is the data analysis sufficiently rigorous?**<br>For example:<br>• Is the procedure explicit – i.e. is it clear how the data was analysed to arrive at the results?<br>• How systematic is the analysis, is the procedure reliable/dependable?<br>• Is it clear how the themes and concepts were derived from the data? | Rigorous<br>Not rigorous<br>Not sure/not reported ✓ | Comments: little detail about how themes derived but ? this is due to the fact that they are reported in another publication |
| **9. Is the data 'rich'?**<br>For example:<br>• How well are the contexts of the data described?<br>• Has the diversity of perspective and content been explored?<br>• How well has the detail and depth been demonstrated?<br>• Are responses compared and contrasted | Rich<br>Poor<br>Not sure/not reported ✓ | Comments: little detail about how themes derived but ? this is due to the fact that they are reported in another publication. But findings are relevant to reflecting on the context and experience of doing the project using adapted EBCD |

| | | |
|---|---|---|
| across groups/sites? | | |
| **10. Is the analysis reliable?**<br>For example:<br>• Did more than 1 researcher theme and code transcripts/data?<br>• If so, how were differences resolved?<br>• Did participants feed back on the transcripts/data if possible and relevant?<br>• Were negative/discrepant results addressed or ignored? | Reliable<br>Unreliable<br>Not sure/not reported ✓ | Comments: a/above |
| **11. Are the findings convincing?**<br>For example:<br>• Are the findings clearly presented?<br>• Are the findings internally coherent?<br>• Are extracts from the original data included?<br>• Are the data appropriately referenced?<br>• Is the reporting clear and coherent? | Convincing ✓<br>Not convincing<br>Not sure | Comments:<br>a/above |
| **12. Are the findings relevant to the aims of the study?** | Relevant ✓ | Comments: |

| | | |
|---|---|---|
| | Irrelevant<br>Partially relevant | |
| **13. Conclusions**<br>For example:<br>• How clear are the links between data, interpretation and conclusions?<br>• Are the conclusions plausible and coherent?<br>• Have alternative explanations been explored and discounted?<br>• Does this enhance understanding of the research topic?<br>• Are the implications of the research clearly defined?<br>**Is there adequate discussion of any limitations encountered?** | Adequate ✓<br>Inadequate<br>Not sure | Comments: |
| **Ethics** | | |
| **14. How clear and coherent is the reporting of ethics?**<br>For example:<br>• Have ethical issues been taken into consideration?<br>• Are they adequately discussed e.g. do they | Appropriate ✓<br>Inappropriate<br>Not sure/not reported | Comments: |

| | | |
|---|---|---|
| address consent and anonymity?<br>• Have the consequences of the research been considered i.e. raising expectations, changing behaviour?<br>• Was the study approved by an ethics committee? | | |
| **Overall assessment** | | |
| **As far as can be ascertained from the paper, how well was the study conducted? (see guidance notes)** | ++ ✓<br>Revised to + after independent third review. | Comments: assessment given relevant to the type of reporting (ie experience of adapted EBCD- primary interview data referred to only) |

| | |
|---|---|
| **Study identification:** Include author, title, reference, year of publication | Larkin, M et al, 2015 On the Brink of Genuinely Collaborative Care: Experience-Based Co-Design in Mental Health. Qualitative Health Research2015, Vol. 25(11) 1463–1476 |
| **Guidance topic:** Co-production in acute healthcare settings. | Key research question/aim: Not clearly stated however the researchers aimed to use their existing research to develop patient care to better meet the needs of young people with psychosis. EBCD was considered to be a good way to translate research findings into service improvements. |
| **Checklist completed by:** | RH |

| | | |
|---|---|---|
| **Theoretical approach:** No specific theoretical framework was reported | | |
| **1. Is a qualitative approach appropriate?**<br>For example:<br>• Does the research question seek to understand processes or structures, or illuminate subjective experiences or meanings?<br>• Could a quantitative approach better have addressed the research question? | Appropriate ✓<br>Inappropriate<br>Not sure | Comments: |
| **2. Is the study clear in what it seeks to do?**<br>For example:<br>• Is the purpose of the study discussed – aims/objectives/research question/s?<br>• Is there adequate/appropriate reference to the literature?<br>• Are underpinning values/assumptions/theory discussed? | Clear ✓<br>Unclear<br>Mixed | Comments: |
| **Study design** | | |
| **3. How defensible/rigorous is the research design/methodology?**<br>For example: | Defensible<br>Indefensible | Comments: the qualitative study does appear to be rigorous however, the audit after the co-design aspects of the paper is much less clear. |

| | | |
|---|---|---|
| • Is the design appropriate to the research question?<br>• Is a rationale given for using a qualitative approach?<br>• Are there clear accounts of the rationale/justification for the sampling, data collection and data analysis techniques used?<br>• Is the selection of cases/sampling strategy theoretically justified? | Not sure ✓ | |
| **Data collection** | | |
| **4. How well was the data collection carried out?**<br>For example:<br>• Are the data collection methods clearly described?<br>• Were the appropriate data collected to address the research question?<br>• Was the data collection and record keeping systematic? | Appropriately ✓<br>Inappropriately<br>Not sure/inadequately reported | Comments: the qualitative study does appear to be rigorous however, the audit after the co-design aspects of the paper is much less clear |
| **Trustworthiness** | | |

| | | |
|---|---|---|
| **5. Is the role of the researcher clearly described?**<br>For example:<br>• Has the relationship between the researcher and the participants been adequately considered?<br>• Does the paper describe how the research was explained and presented to the participants? | Clearly described<br>Unclear ✓<br>Not described | Comments: |
| **6. Is the context clearly described?**<br>For example:<br>• Are the characteristics of the participants and settings clearly defined?<br>• Were observations made in a sufficient variety of circumstances<br>• Was context bias considered | Clear ✓<br>Unclear<br>Not sure | Comments: |
| **7. Were the methods reliable?**<br>For example:<br>• Was data collected by more than 1 method?<br>• Is there justification for triangulation, or for not triangulating? | Reliable<br>Unreliable<br>Not sure ✓ | Comments: not reported |

| | | |
|---|---|---|
| • Do the methods investigate what they claim to? | | |
| **Analysis** | | |
| **8. Is the data analysis sufficiently rigorous?**<br>For example:<br>• Is the procedure explicit – i.e. is it clear how the data was analysed to arrive at the results?<br>• How systematic is the analysis, is the procedure reliable/dependable?<br>• Is it clear how the themes and concepts were derived from the data? | Rigorous<br>Not rigorous<br>Not sure/not reported ✓ | Comments: not reported |
| **9. Is the data 'rich'?**<br>For example:<br>• How well are the contexts of the data described?<br>• Has the diversity of perspective and content been explored?<br>• How well has the detail and depth been demonstrated?<br>• Are responses compared and contrasted | Rich ✓<br>Poor<br>Not sure/not reported | Comments: I think there is a lot of information about conducting EBCD but it doesn't fit easily in the usual quality assessments. However, I think the complexity of what we are looking at makes it difficult. |

| | | |
|---|---|---|
| across groups/sites? | | |
| **10. Is the analysis reliable?**<br>For example:<br>• Did more than 1 researcher theme and code transcripts/data?<br>• If so, how were differences resolved?<br>• Did participants feed back on the transcripts/data if possible and relevant?<br>• Were negative/discrepant results addressed or ignored? | Reliable<br>Unreliable<br>Not sure/not reported ✓ | Comments: not much detail given |
| **11. Are the findings convincing?**<br>For example:<br>• Are the findings clearly presented?<br>• Are the findings internally coherent?<br>• Are extracts from the original data included?<br>• Are the data appropriately referenced?<br>• Is the reporting clear and coherent? | Convincing ✓<br>Not convincing<br>Not sure | Comments: The findings aren't really 'findings' as such. It is a discussion of implementing EBCD. The information is convincing in terms of what we learn about the challenges and facilitators of using EBCD |
| **12. Are the findings relevant to the aims of the study?** | Relevant ✓<br>Irrelevant | Comments: |

| | | |
|---|---|---|
| | Partially relevant | |
| **13. Conclusions**<br>For example:<br>• How clear are the links between data, interpretation and conclusions?<br>• Are the conclusions plausible and coherent?<br>• Have alternative explanations been explored and discounted?<br>• Does this enhance understanding of the research topic?<br>• Are the implications of the research clearly defined?<br>**Is there adequate discussion of any limitations encountered?** | Adequate ✓<br>Inadequate<br>Not sure | Comments: |
| **Ethics** | | |
| **14. How clear and coherent is the reporting of ethics?**<br>For example:<br>• Have ethical issues been taken into consideration?<br>• Are they adequately discussed e.g. do they | Appropriate✓<br>Inappropriate<br>Not sure/not reported | Comments: |

| | | |
|---|---|---|
| address consent and anonymity?<br>• Have the consequences of the research been considered i.e. raising expectations, changing behaviour?<br>• Was the study approved by an ethics committee? | | |
| **Overall assessment** | | |
| **As far as can be ascertained from the paper, how well was the study conducted? (see guidance notes)** | ++<br>+ ✓<br>− | Comments: |

| | |
|---|---|
| **Study identification:** Include author, title, reference, year of publication | Locock, L et al, (2014) Using a national archive of patient experience narratives to promote local patient-centered quality improvement: an ethnographic process evaluation of 'accelerated' experience-based co-design. Journal of Health Services Research &Policy2014, Vol. 19(4) 200–207 |
| **Guidance topic:** Co-production in acute healthcare settings. | **Key research question/aim:** |
| **Checklist completed by:** | FJ |
| **Theoretical approach: A-EBCD v EBCD (12 existing studies)  Ethnographic process evaluation using multiple methods** | |

| | | |
|---|---|---|
| **1. Is a qualitative approach appropriate?**<br>For example:<br>• Does the research question seek to understand processes or structures, or illuminate subjective experiences or meanings?<br>• Could a quantitative approach better have addressed the research question? | Appropriate ✓<br>Inappropriate<br>Not sure | Comments: |
| **2. Is the study clear in what it seeks to do?**<br>For example:<br>• Is the purpose of the study discussed – aims/objectives/research question/s?<br>• Is there adequate/appropriate reference to the literature?<br>• Are underpinning values/assumptions/theory discussed? | Clear ✓<br>Unclear<br>Mixed | Comments: |
| **Study design** | | |
| **3. How defensible/rigorous is the research design/methodology?**<br>For example:<br>• Is the design appropriate to the research question?<br>• Is a rationale given for using a qualitative approach?<br>• Are there clear accounts of the rationale/justification for | Defensible ✓<br>Indefensible<br>Not sure | Comments: |

| | | |
|---|---|---|
| the sampling, data collection and data analysis techniques used?<br>• Is the selection of cases/sampling strategy theoretically justified? | | |
| **Data collection** | | |
| **4. How well was the data collection carried out?**<br>For example:<br>• Are the data collection methods clearly described?<br>• Were the appropriate data collected to address the research question?<br>• Was the data collection and record keeping systematic? | Appropriately ✓<br>Inappropriately<br>Not sure/inadequately reported | Comments:<br>Limited reporting of findings – no detail about pathway and impact of improvement activities |
| **Trustworthiness** | | |

| | | |
|---|---|---|
| **5. Is the role of the researcher clearly described?**<br>For example:<br>• Has the relationship between the researcher and the participants been adequately considered?<br>• Does the paper describe how the research was explained and presented to the participants? | Clearly described<br>Unclear<br>Not described ✓ | Comments: |
| **6. Is the context clearly described?**<br>For example:<br>• Are the characteristics of the participants and settings clearly defined?<br>• Were observations made in a sufficient variety of circumstances<br>• Was context bias considered | Clear<br>Unclear ✓<br>Not sure | Comments:<br>Very little detail about context- Lung cancer and ITU; context bas not considered |
| **7. Were the methods reliable?**<br>For example:<br>• Was data collected by more than 1 method?<br>• Is there justification for triangulation, or for not triangulating? | Reliable ✓<br>Unreliable<br>Not sure | Comments: |

| | | |
|---|---|---|
| • Do the methods investigate what they claim to? | | |
| **Analysis** | | |
| **8. Is the data analysis sufficiently rigorous?**<br>For example:<br>• Is the procedure explicit – i.e. is it clear how the data was analysed to arrive at the results?<br>• How systematic is the analysis, is the procedure reliable/dependable?<br>• Is it clear how the themes and concepts were derived from the data? | Rigorous ✓<br>Not rigorous<br>Not sure/not reported | Comments: |
| **9. Is the data 'rich'?**<br>For example:<br>• How well are the contexts of the data described?<br>• Has the diversity of perspective and content been explored?<br>• How well has the detail and depth been demonstrated?<br>• Are responses compared and contrasted across groups/sites? | Rich<br>Poor<br>Not sure/not reported ✓ | Comments: minimal reporting of context, and whether responses were compared/contrasted |
| **10. Is the analysis reliable?** | Reliable | Comments: |

| | | |
|---|---|---|
| For example:<br>• Did more than 1 researcher theme and code transcripts/data?<br>• If so, how were differences resolved?<br>• Did participants feed back on the transcripts/data if possible and relevant?<br>• Were negative/discrepant results addressed or ignored? | Unreliable<br>Not sure/not reported ✓ | |
| **11. Are the findings convincing?**<br>For example:<br>• Are the findings clearly presented?<br>• Are the findings internally coherent?<br>• Are extracts from the original data included?<br>• Are the data appropriately referenced?<br>• Is the reporting clear and coherent? | Convincing ✓<br>Not convincing<br>Not sure | Comments: |
| **12. Are the findings relevant to the aims of the study?** | Relevant ✓<br>Irrelevant<br>Partially relevant | Comments: |
| **13. Conclusions**<br>For example: | Adequate ✓<br>Inadequate | Comments: |

| | | |
|---|---|---|
| • How clear are the links between data, interpretation and conclusions?<br>• Are the conclusions plausible and coherent?<br>• Have alternative explanations been explored and discounted?<br>• Does this enhance understanding of the research topic?<br>• Are the implications of the research clearly defined?<br>**Is there adequate discussion of any limitations encountered?** | Not sure | |
| **Ethics** | | |
| **14. How clear and coherent is the reporting of ethics?**<br>For example:<br>• Have ethical issues been taken into consideration?<br>• Are they adequately discussed e.g. do they address consent and anonymity?<br>• Have the consequences of the research been considered i.e. raising expectations, changing behaviour?<br>• Was the study approved by an ethics committee? | Appropriate ✓<br>Inappropriate<br>Not sure/not reported | Comments: |
| **Overall assessment** | | |
| **As far as can be ascertained from the paper, how well was** | ++ ✓ | Comments: |

| | | |
|---|---|---|
| **the study conducted? (see guidance notes)** | | |

| | | |
|---|---|---|
| **Study identification:** Include author, title, reference, year of publication | Locock, L et al, (2014) Using a national archive of patient experience narratives to promote local patient-centered quality improvement: an ethnographic process evaluation of 'accelerated' experience-based co-design. Journal of Health Services Research &Policy2014, Vol. 19(4) 200–207. | |
| **Guidance topic:** Co-production in acute healthcare settings. | **Key research question/aim: 3 questions identified.**<br>1. What improvement activities does the approach stimulate (for what cost) and how do these compare with traditional EBCD?<br>2. How does using films of national rather than local narratives affect the level and quality of engagement with service improvement by local NHS staff?<br>3. How well do local patients feel national narratives represent themes important to their own experience? | |
| **Checklist completed by:** | DJC | |
| **Theoretical approach: A-EBCD v EBCD (12 existing studies) Ethnographic process evaluation using multiple methods** | | |
| **1. Is a qualitative approach appropriate?**<br>For example:<br>• Does the research question seek to understand processes or structures, or illuminate subjective experiences or meanings?<br>• Could a quantitative approach better have addressed the research question? | Appropriate ✓<br>Inappropriate<br>Not sure | Comments: Important to understand factors influencing the implementation of the accelerated approach and to observe the process 'first hand', same ethnographer involved though out all stages of data collection.<br>Also compared this study's survey data with that from evaluations of 12 previous EBCD studies. |
| **2. Is the study clear in what it seeks to do?** | Clear ✓ | Comments: Comparing AEBCD with EBCD by investigated whether archive film could replace the need for local |

| | | |
|---|---|---|
| For example:<br><br>• Is the purpose of the study discussed – aims/objectives/research question/s?<br><br>• Is there adequate/appropriate reference to the literature?<br><br>• Are underpinning values/assumptions/theory discussed? | Unclear<br><br>Mixed | discovery interviews (and film) so making EBCD faster and cheaper; aimed to halve the standard EBCD cycle from 12 to 6 months. |
| **Study design** | | |
| **3. How defensible/rigorous is the research design/methodology?**<br><br>For example:<br><br>• Is the design appropriate to the research question?<br><br>• Is a rationale given for using a qualitative approach?<br><br>• Are there clear accounts of the rationale/justification for the sampling, data collection and data analysis techniques used?<br><br>• Is the selection of cases/sampling strategy theoretically justified? | Defensible ✓<br><br>Indefensible<br><br>Not sure | Comments: Clear justification for the methods adopted, consistent with good practice in ethnographic process evaluation. |
| **Data collection** | | |
| **4. How well was the data collection carried out?**<br><br>For example: | Appropriately ✓<br><br>Inappropriately | Comments: Clear description of data collection methods, more limited reporting of findings other than for improvement activities. |

| | | |
|---|---|---|
| • Are the data collection methods clearly described?<br>• Were the appropriate data collected to address the research question?<br>• Was the data collection and record keeping systematic? | Not sure/inadequately reported | |
| **Trustworthiness** | | |
| **5. Is the role of the researcher clearly described?**<br>For example:<br>• Has the relationship between the researcher and the participants been adequately considered?<br>• Does the paper describe how the research was explained and presented to the participants? | Clearly described<br>Unclear<br>Not described ✓ | Comments: |
| **6. Is the context clearly described?**<br>For example:<br>• Are the characteristics of the participants and settings clearly defined?<br>• Were observations made in a sufficient variety of circumstances | Clear<br>Unclear ✓<br>Not sure | Comments:<br>Little detail about context, focus specifically on impact of using a national versus us locally derived film, disease specific condition less relevant in this publication. |

| | | |
|---|---|---|
| • Was context bias considered | | |
| **7. Were the methods reliable?**<br>For example:<br>• Was data collected by more than 1 method?<br>• Is there justification for triangulation, or for not triangulating?<br>• Do the methods investigate what they claim to? | Reliable ✓<br>Unreliable<br>Not sure | Comments: Appropriate and clearly articulated. |
| **Analysis** | | |
| **8. Is the data analysis sufficiently rigorous?**<br>For example:<br>• Is the procedure explicit – i.e. is it clear how the data was analysed to arrive at the results?<br>• How systematic is the analysis, is the procedure reliable/dependable?<br>• Is it clear how the themes and concepts were derived from the data? | Rigorous ✓<br>Not rigorous<br>Not sure/not reported | Comments: Brief summary of approaches used, all consistent with the ethnographic process evaluation and the survey data generated. |
| **9. Is the data 'rich'?**<br>For example:<br>• How well are the contexts of the data described? | Rich<br>Poor<br>Not sure/not reported ✓ | Comments: minimal reporting of context focus specifically on impact of using a national versus us locally derived film, disease specific condition and location of the study less relevant in this publication. |

| | | |
|---|---|---|
| • Has the diversity of perspective and content been explored?<br>• How well has the detail and depth been demonstrated?<br>• Are responses compared and contrasted across groups/sites? | | |
| **10. Is the analysis reliable?**<br>For example:<br>• Did more than 1 researcher theme and code transcripts/data?<br>• If so, how were differences resolved?<br>• Did participants feed back on the transcripts/data if possible and relevant?<br>• Were negative/discrepant results addressed or ignored? | Reliable<br>Unreliable<br>Not sure/not reported ✓ | Comments: Reports a two day workshop with the study team but no other details. |
| **11. Are the findings convincing?**<br>For example:<br>• Are the findings clearly presented?<br>• Are the findings internally coherent?<br>• Are extracts from the original data included?<br>• Are the data appropriately referenced?<br>• Is the reporting clear and coherent? | Convincing ✓<br>Not convincing<br>Not sure | Comments: |

| | | |
|---|---|---|
| **12. Are the findings relevant to the aims of the study?** | Relevant ✓<br>Irrelevant<br>Partially relevant | Comments: |
| **13. Conclusions**<br>For example:<br>• How clear are the links between data, interpretation and conclusions?<br>• Are the conclusions plausible and coherent?<br>• Have alternative explanations been explored and discounted?<br>• Does this enhance understanding of the research topic?<br>• Are the implications of the research clearly defined?<br>**Is there adequate discussion of any limitations encountered?** | Adequate ✓<br>Inadequate<br>Not sure | Comments: |
| **Ethics** | | |
| **14. How clear and coherent is the reporting of ethics?**<br>For example:<br>• Have ethical issues been taken into consideration?<br>• Are they adequately discussed e.g. do they address consent and anonymity?<br>• Have the consequences of the research been considered | Appropriate ✓<br>Inappropriate<br>Not sure/not reported | Comments: |

| | | |
|---|---|---|
| i.e. raising expectations, changing behaviour?<br>• Was the study approved by an ethics committee? | | |
| **Overall assessment** | | |
| **As far as can be ascertained from the paper, how well was the study conducted? (see guidance notes)** | ++ ✓ | Comments: |

| | |
|---|---|
| **Study identification:** Include author, title, reference, year of publication | Piper, D. et al, 2012 Utilizing experience-based co-design to improve the experience of patients accessing emergency departments in New South Wales public hospitals: an evaluation study. Health Services Management Research2012;25: 162 – 172 |
| **Guidance topic:** Co-production in acute healthcare settings. | **Key research question/aim:**<br><br>What specific improvements did EBCD deliver for patients, carers and staff in the Emergency Departments involved in the project?<br><br>What did it feel like to take part in EBCD as compared to other redesign approaches? What did participants identify as the 'must do' or key success factors in EBCD?<br><br>What can the pilot tell us about the likely sustainability and spread of improvements brought about by EBCD?<br><br>What lessons can be drawn from the pilot about future EBCD projects in New South Wales? |

| | | |
|---|---|---|
| **Checklist completed by:** | FJ | |
| **Theoretical approach:** evaluation of existing EBCD improvement projects; no other details about theoretical approach | | |
| **1. Is a qualitative approach appropriate?**<br>For example:<br>• Does the research question seek to understand processes or structures, or illuminate subjective experiences or meanings?<br>• Could a quantitative approach better have addressed the research question? | Appropriate ✓<br>Inappropriate<br>Not sure | Comments: |
| **2. Is the study clear in what it seeks to do?**<br>For example:<br>• Is the purpose of the study discussed – aims/objectives/research question/s?<br>• Is there adequate/appropriate reference to the literature?<br>• Are underpinning values/assumptions/theory discussed? | Clear ✓<br>Unclear<br>Mixed | Comments: |
| **Study design** | | |
| **3. How defensible/rigorous is the research design/methodology?** | Defensible | Comments: |

| | | |
|---|---|---|
| For example:<br>• Is the design appropriate to the research question?<br>• Is a rationale given for using a qualitative approach?<br>• Are there clear accounts of the rationale/justification for the sampling, data collection and data analysis techniques used?<br>• Is the selection of cases/sampling strategy theoretically justified? | Indefensible<br>Not sure ✓ | Not much detail about sampling. Selection of cases not justified. |
| **Data collection** | | |
| **4. How well was the data collection carried out?**<br>For example:<br>• Are the data collection methods clearly described?<br>• Were the appropriate data collected to address the research question?<br>• Was the data collection and record keeping systematic? | Appropriately ✓<br>Inappropriately<br>Not sure/inadequately reported | Comments: |
| **Trustworthiness** | | |

| | | |
|---|---|---|
| **5. Is the role of the researcher clearly described?**<br>For example:<br>• Has the relationship between the researcher and the participants been adequately considered?<br>• Does the paper describe how the research was explained and presented to the participants? | Clearly described<br>Unclear ✓<br>Not described | Comments: interview and document data analysed by an independent analyst |
| **6. Is the context clearly described?**<br>For example:<br>• Are the characteristics of the participants and settings clearly defined?<br>• Were observations made in a sufficient variety of circumstances<br>• Was context bias considered | Clear ✓<br>Unclear<br>Not sure | Comments: no comment on content bias |
| **7. Were the methods reliable?**<br>For example:<br>• Was data collected by more than 1 method?<br>• Is there justification for triangulation, or for not triangulating? | Reliable ✓<br>Unreliable<br>Not sure | Comments:<br>Interviews with participants, analysis of project reports, interviews senior managers |

| | | |
|---|---|---|
| • Do the methods investigate what they claim to? | | |
| **Analysis** | | |
| **8. Is the data analysis sufficiently rigorous?**<br>For example:<br>• Is the procedure explicit – i.e. is it clear how the data was analysed to arrive at the results?<br>• How systematic is the analysis, is the procedure reliable/dependable?<br>• Is it clear how the themes and concepts were derived from the data? | Rigorous<br>Not rigorous<br>Not sure/not ✓reported | Comments:<br>Not clear how findings were arrived at |
| **9. Is the data 'rich'?**<br>For example:<br>• How well are the contexts of the data described?<br>• Has the diversity of perspective and content been explored?<br>• How well has the detail and depth been demonstrated?<br>• Are responses compared and contrasted across groups/sites? | Rich<br>Poor<br>Not sure/not reported ✓ | Comments: no illustrative quotes. Data structured into main categories (barriers and achievements) but no themes/sub-themes |

| | | |
|---|---|---|
| **10. Is the analysis reliable?**<br>For example:<br>• Did more than 1 researcher theme and code transcripts/data?<br>• If so, how were differences resolved?<br>• Did participants feed back on the transcripts/data if possible and relevant?<br>• Were negative/discrepant results addressed or ignored? | Reliable<br>Unreliable<br>Not sure/not reported ✓ | Comments: |
| **11. Are the findings convincing?**<br>For example:<br>• Are the findings clearly presented?<br>• Are the findings internally coherent?<br>• Are extracts from the original data included?<br>• Are the data appropriately referenced?<br>• Is the reporting clear and coherent? | Convincing ✓<br>Not convincing<br>Not sure | Comments:<br>Finding reported clearly but as a commentary rather than using a traditional way with themes/sub-themes –illustrative quotes etc. |
| **12. Are the findings relevant to the aims of the study?** | Relevant ✓<br>Irrelevant<br>Partially relevant | Comments: |

| | | |
|---|---|---|
| **13. Conclusions**<br><br>For example:<br><br>• How clear are the links between data, interpretation and conclusions?<br><br>• Are the conclusions plausible and coherent?<br><br>• Have alternative explanations been explored and discounted?<br><br>• Does this enhance understanding of the research topic?<br><br>• Are the implications of the research clearly defined?<br><br>**Is there adequate discussion of any limitations encountered?** | Adequate ✓<br><br>Inadequate<br><br>Not sure | Comments: |
| **Ethics** | | |
| **14. How clear and coherent is the reporting of ethics?**<br><br>For example:<br><br>• Have ethical issues been taken into consideration?<br><br>• Are they adequately discussed e.g. do they address consent and anonymity?<br><br>• Have the consequences of the research been considered i.e. raising expectations, changing behaviour? | Appropriate<br><br>Inappropriate<br><br>Not sure/not reported ✓ | Comments:<br><br>No mention of ethical approval |

| | | |
|---|---|---|
| • Was the study approved by an ethics committee? | | |
| **Overall assessment** | | |
| **As far as can be ascertained from the paper, how well was the study conducted? (see guidance notes)** | + ✓ | Comments: |

| | |
|---|---|
| **Study identification:** Include author, title, reference, year of publication | Piper, D. et al, 2012 Utilizing experience-based co-design to improve the experience of patients accessing emergency departments in New South Wales public hospitals: an evaluation study. Health Services Management Research2012;25: 162 – 172 |
| **Guidance topic:** Co-production in acute healthcare settings. | **Key research question/aim:** Not clearly stated - This paper presents the findings from three evaluations of two subsequent multisite EBCD programmes conducted by New South Wales Health (Australia) over two years involving seven hospital sites to take stock of the impact of EBCD on patients' experience of ED care. |
| **Checklist completed by:** | RH |
| **Theoretical approach:** No specific theoretical framework was reported | |

| | | |
|---|---|---|
| **1. Is a qualitative approach appropriate?**<br>For example:<br>• Does the research question seek to understand processes or structures, or illuminate subjective experiences or meanings? | Appropriate ✓<br>Inappropriate<br>Not sure | Comments: |

| | | |
|---|---|---|
| • Could a quantitative approach better have addressed the research question? | | |
| **2. Is the study clear in what it seeks to do?**<br>For example:<br>• Is the purpose of the study discussed – aims/objectives/research question/s?<br>• Is there adequate/appropriate reference to the literature?<br>• Are underpinning values/assumptions/theory discussed? | Clear✓<br>Unclear<br>Mixed | Comments: |
| **Study design** | | |
| **3. How defensible/rigorous is the research design/methodology?**<br>For example:<br>• Is the design appropriate to the research question?<br>• Is a rationale given for using a qualitative approach?<br>• Are there clear accounts of the rationale/justification for the sampling, data collection and data analysis techniques used?<br>• Is the selection of cases/sampling strategy theoretically justified? | Defensible ✓<br>Indefensible<br>Not sure | Comments: |

| | | |
|---|---|---|
| **Data collection** | | |
| **4. How well was the data collection carried out?**<br>For example:<br>• Are the data collection methods clearly described?<br>• Were the appropriate data collected to address the research question?<br>• Was the data collection and record keeping systematic? | Appropriately ✓<br>Inappropriately<br>Not sure/inadequately reported | Comments: not a great deal of detail is given |
| **Trustworthiness** | | |
| **5. Is the role of the researcher clearly described?**<br>For example:<br>• Has the relationship between the researcher and the participants been adequately considered?<br>• Does the paper describe how the research was explained and presented to the participants? | Clearly described<br>Unclear ✓<br>Not described | Comments: |
| **6. Is the context clearly described?** | Clear ✓ | Comments: |

| | | |
|---|---|---|
| For example:<br><br>• Are the characteristics of the participants and settings clearly defined?<br><br>• Were observations made in a sufficient variety of circumstances<br><br>• Was context bias considered | Unclear<br><br>Not sure | |
| **7. Were the methods reliable?**<br>For example:<br><br>• Was data collected by more than 1 method?<br><br>• Is there justification for triangulation, or for not triangulating?<br><br>• Do the methods investigate what they claim to? | Reliable<br><br>Unreliable<br><br>Not sure ✓ | Comments: varied across sites – not fully reported in an auditable way |
| **Analysis** | | |
| **8. Is the data analysis sufficiently rigorous?**<br>For example:<br><br>• Is the procedure explicit – i.e. is it clear how the data was analysed to arrive at the results?<br><br>• How systematic is the analysis, is the procedure reliable/dependable?<br><br>• Is it clear how the themes and concepts were | Rigorous<br><br>Not rigorous<br><br>Not sure/not reported ✓ | Comments: one researcher did analysis, not much further detail given. |

| | | |
|---|---|---|
| derived from the data? | | |
| **9. Is the data 'rich'?**<br>For example:<br>• How well are the contexts of the data described?<br>• Has the diversity of perspective and content been explored?<br>• How well has the detail and depth been demonstrated?<br>• Are responses compared and contrasted across groups/sites? | Rich<br>Poor<br>Not sure/not reported ✓ | Comments: not a lot of detail |
| **10. Is the analysis reliable?**<br>For example:<br>• Did more than 1 researcher theme and code transcripts/data?<br>• If so, how were differences resolved?<br>• Did participants feed back on the transcripts/data if possible and relevant?<br>• Were negative/discrepant results addressed or ignored? | Reliable<br>Unreliable<br>Not sure/not reported ✓ | Comments: not much detail given, one researcher |
| **11. Are the findings convincing?** | Convincing ✓ | Comments: |

| | | |
|---|---|---|
| For example:<br>• Are the findings clearly presented?<br>• Are the findings internally coherent?<br>• Are extracts from the original data included?<br>• Are the data appropriately referenced?<br>• Is the reporting clear and coherent? | Not convincing<br>Not sure | |
| **12. Are the findings relevant to the aims of the study?** | Relevant ✓<br>Irrelevant<br>Partially relevant | Comments: |
| **13. Conclusions**<br>For example:<br>• How clear are the links between data, interpretation and conclusions?<br>• Are the conclusions plausible and coherent?<br>• Have alternative explanations been explored and discounted?<br>• Does this enhance understanding of the research topic?<br>• Are the implications of the research clearly defined?<br>**Is there adequate discussion of any limitations** | Adequate ✓<br>Inadequate<br>Not sure | Comments: |

| | | |
|---|---|---|
| **encountered?** | | |
| **Ethics** | | |
| **14. How clear and coherent is the reporting of ethics?**<br>For example:<br>• Have ethical issues been taken into consideration?<br>• Are they adequately discussed e.g. do they address consent and anonymity?<br>• Have the consequences of the research been considered i.e. raising expectations, changing behaviour?<br>• Was the study approved by an ethics committee? | Appropriate<br>Inappropriate<br>Not sure/not reported ✓ | Comments: Not addressed in the paper |
| **Overall assessment** | | |
| **As far as can be ascertained from the paper, how well was the study conducted? (see guidance notes)** | ++<br>**+** ✓<br>− | Comments: |

| | | |
|---|---|---|
| **Study identification:** Include author, title, reference, year of publication | Multiple sclerosis outpatient future groups: improving the quality of participant interaction and ideation tools within service improvement activities. Alison Thomson, Carol Rivas and Gavin Giovannoni. BMC Health Services Research (2015) 15:105. DOI 10.1186/s12913-015-0773-8 | |
| **Guidance topic:** | **Key research question/aim:**<br><br>The aim of the study is to consider how the role of a professional designer as the facilitator can develop more radical ideas, beyond solving simple problems, from patients and staff in a service improvement activity. | |
| **Checklist completed by:** | SH | |
| **Theoretical approach** | | |
| **1. Is a qualitative approach appropriate?**<br><br>For example:<br><br>• Does the research question seek to understand processes or structures, or illuminate subjective experiences or meanings?<br><br>• Could a quantitative approach better have addressed the research question? | Appropriate ✓<br><br>Inappropriate<br><br>Not sure | Comments: Appropriate - Illuminates subjective experiences which could not have been carried out by quantitative approaches. |
| **2. Is the study clear in what it seeks to do?**<br><br>For example:<br><br>• Is the purpose of the study discussed – | Clear ✓<br><br>Unclear<br><br>Mixed | Comments: The aim and the EBCD approach is explained and discussed. The references are appropriate. |

| | | |
|---|---|---|
| aims/objectives/research question/s?<br>• Is there adequate/appropriate reference to the literature?<br>• Are underpinning values/assumptions/theory discussed? | | |
| **Study design** | | |
| **3. How defensible/rigorous is the research design/methodology?**<br>For example:<br>• Is the design appropriate to the research question?<br>• Is a rationale given for using a qualitative approach?<br>• Are there clear accounts of the rationale/justification for the sampling, data collection and data analysis techniques used?<br>• Is the selection of cases/sampling strategy theoretically justified? | Defensible ✓<br>Indefensible<br>Not sure | Comments: The focus group approach is appropriate for eliciting the views of staff and patients. The rationale for the sampling of the staff and patient participant groups are justified. |
| **Data collection** | | |
| **4. How well was the data collection carried out?**<br>For example:<br>• Are the data collection methods clearly | Appropriately ✓<br>Inappropriately | Comments: The mapping of the staff and patient journey and the development of service improvement are clearly described. The data collection is systematic and includes verbatim quotations, photographs |

| | | |
|---|---|---|
| described?<br><br>• Were the appropriate data collected to address the research question?<br><br>• Was the data collection and record keeping systematic? | Not sure/inadequately reported | and diagrams. |
| **Trustworthiness** | | |
| **5. Is the role of the researcher clearly described?**<br><br>For example:<br><br>• Has the relationship between the researcher and the participants been adequately considered?<br><br>• Does the paper describe how the research was explained and presented to the participants? | Clearly described ✓<br><br>Unclear<br><br>Not described | Comments: The relationships are described. The participants were involved with the research on an on-going basis via the design-led process. |
| **6. Is the context clearly described?**<br><br>For example:<br><br>• Are the characteristics of the participants and settings clearly defined?<br><br>• Were observations made in a sufficient variety of circumstances<br><br>• Was context bias considered | Clear ✓<br><br>Unclear<br><br>Not sure | Comments: The context of the MS outpatient clinic was clearly defined. A range of staff participants were interviewed and male and female patients included. The authors acknowledge that the final context of use should be considered. |
| **7. Were the methods reliable?** | Reliable ✓ | Comments: The methods were reliable.. Analogies and props were used to elicit rich data. Non-participant observation of the focus groups was |

| | | |
|---|---|---|
| For example:<br><br>• Was data collected by more than 1 method?<br><br>• Is there justification for triangulation, or for not triangulating?<br><br>• Do the methods investigate what they claim to? | Unreliable<br><br>Not sure | also carried out. |
| **Analysis** | | |
| **8. Is the data analysis sufficiently rigorous?**<br><br>For example:<br><br>• Is the procedure explicit – i.e. is it clear how the data was analysed to arrive at the results?<br><br>• How systematic is the analysis, is the procedure reliable/dependable?<br><br>• Is it clear how the themes and concepts were derived from the data? | Rigorous<br><br>Not rigorous ✓<br><br>Not sure/not reported | Comments: It is unclear as the analysis is only briefly reported but it is stated that thematic analysis was carried out using NVivo v (8) and grounded theory principles were used. |
| **9. Is the data 'rich'?**<br><br>For example:<br><br>• How well are the contexts of the data described?<br><br>• Has the diversity of perspective and content been explored?<br><br>• How well has the detail and depth been demonstrated? | Rich ✓<br><br>Poor<br><br>Not sure/not reported | Comments: The data includes relevant conversation excerpts and photographs of work completed by the groups. |

| | | |
|---|---|---|
| • Are responses compared and contrasted across groups/sites? | | |
| **10. Is the analysis reliable?**<br><br>For example:<br><br>• Did more than 1 researcher theme and code transcripts/data?<br><br>• If so, how were differences resolved?<br><br>• Did participants feed back on the transcripts/data if possible and relevant?<br><br>• Were negative/discrepant results addressed or ignored? | Reliable<br><br>Unreliable<br><br>Not sure/not reported ✓ | Comments: |
| **11. Are the findings convincing?**<br><br>For example:<br><br>• Are the findings clearly presented?<br><br>• Are the findings internally coherent?<br><br>• Are extracts from the original data included?<br><br>• Are the data appropriately referenced?<br><br>• Is the reporting clear and coherent? | Convincing ✓<br><br>Not convincing<br><br>Not sure | Comments: The findings are clearly presented and include extracts from the original data in then form of quotations and photographs. |
| **12. Are the findings relevant to the aims of the study?** | Relevant x<br><br>Irrelevant | Comments: The findings are relevant and show that design-led methods can facilitate the development of ideas and enhance service improvement activities. |

| | | |
|---|---|---|
| | Partially relevant | |
| **13. Conclusions**<br><br>For example:<br><br>• How clear are the links between data, interpretation and conclusions?<br><br>• Are the conclusions plausible and coherent?<br><br>• Have alternative explanations been explored and discounted?<br><br>• Does this enhance understanding of the research topic?<br><br>• Are the implications of the research clearly defined?<br><br>**Is there adequate discussion of any limitations encountered?** | Adequate ✓<br><br>Inadequate<br><br>Not sure | Comments: The conclusions are plausible and enhance the research topic. The implication that social science methods can be used to integrate design-led approaches in service improvement is clearly defined.<br><br>The limitations are discussed adequately. |
| **Ethics** | | |
| **14. How clear and coherent is the reporting of ethics?**<br><br>For example:<br><br>• Have ethical issues been taken into consideration?<br><br>• Are they adequately discussed e.g. do they address consent and anonymity?<br><br>• Have the consequences of the research been | Appropriate<br><br>Inappropriate<br><br>Not sure/not reported ✓ | Comments: Only very brief references to ethical considerations. It is stated that ethical approval was given. |

| | | |
|---|---|---|
| considered i.e. raising expectations, changing behaviour?<br>• Was the study approved by an ethics committee? | | |
| **Overall assessment** | | |
| **As far as can be ascertained from the paper, how well was the study conducted? (see guidance notes)** | ++<br>+ ✓<br>− | Comments: |

| | |
|---|---|
| **Study identification:** Include author, title, reference, year of publication | Thompson, A., Rivas, C., Giovannoni, G. Multiple sclerosis outpatient future groups: improving the quality of participant interaction and ideation tools within service improvement activities. BMC Health Services Research (2015) 15:105. DOI 10.1186/s12913-015-0773-8 |
| **Guidance topic:** | **Key research question/aim:**<br>The aim of the study is to consider how the role of a professional designer as the facilitator can develop more radical ideas, beyond solving simple problems, from patients and staff in a service improvement activity. |
| **Checklist completed by:** | DJC |
| **Theoretical approach** | |

| | | |
|---|---|---|
| **1. Is a qualitative approach appropriate?**<br><br>For example:<br><br>• Does the research question seek to understand processes or structures, or illuminate subjective experiences or meanings?<br><br>• Could a quantitative approach better have addressed the research question? | Appropriate ✓<br><br>Inappropriate<br><br>Not sure | Comments: |
| **2. Is the study clear in what it seeks to do?**<br><br>For example:<br><br>• Is the purpose of the study discussed – aims/objectives/research question/s?<br><br>• Is there adequate/appropriate reference to the literature?<br><br>• Are underpinning values/assumptions/theory discussed? | Clear ✓<br><br>Unclear<br><br>Mixed | Comments: |
| **Study design** | | |
| **3. How defensible/rigorous is the research design/methodology?**<br><br>For example:<br><br>• Is the design appropriate to the research question?<br><br>• Is a rationale given for using a qualitative | Defensible ✓<br><br>Indefensible<br><br>Not sure | Comments: |

| | | |
|---|---|---|
| approach?<br><br>• Are there clear accounts of the rationale/justification for the sampling, data collection and data analysis techniques used?<br><br>• Is the selection of cases/sampling strategy theoretically justified? | | |
| **Data collection** | | |
| **4. How well was the data collection carried out?**<br><br>For example:<br><br>• Are the data collection methods clearly described?<br><br>• Were the appropriate data collected to address the research question?<br><br>• Was the data collection and record keeping systematic? | Appropriately ✓<br><br>Inappropriately<br><br>Not sure/inadequately reported | Comments: |
| **Trustworthiness** | | |
| **5. Is the role of the researcher clearly described?**<br><br>For example:<br><br>• Has the relationship between the researcher and the participants been adequately considered?<br><br>• Does the paper describe how the research was | Clearly described ✓<br><br>Unclear<br><br>Not described | Comments: |

| | | |
|---|---|---|
| explained and presented to the participants? | | |
| **6. Is the context clearly described?**<br><br>For example:<br><br>• Are the characteristics of the participants and settings clearly defined?<br><br>• Were observations made in a sufficient variety of circumstances<br><br>• Was context bias considered | Clear ✓<br><br>Unclear<br><br>Not sure | Comments: |
| **7. Were the methods reliable?**<br><br>For example:<br><br>• Was data collected by more than 1 method?<br><br>• Is there justification for triangulation, or for not triangulating?<br><br>• Do the methods investigate what they claim to? | Reliable ✓<br><br>Unreliable<br><br>Not sure | Comments: |
| **Analysis** | | |
| **8. Is the data analysis sufficiently rigorous?**<br><br>For example:<br><br>• Is the procedure explicit – i.e. is it clear how the data was analysed to arrive at the results?<br><br>• How systematic is the analysis, is the procedure | Rigorous<br><br>Not rigorous<br><br>Not sure/not reported ✓ | Comments: Analysis is briefly outlined, reports using thematic analysis and grounded theory principles but no more information is provided.<br><br>. |

| | | |
|---|---|---|
| reliable/dependable?<br>• Is it clear how the themes and concepts were derived from the data? | | |
| **9. Is the data 'rich'?**<br>For example:<br>• How well are the contexts of the data described?<br>• Has the diversity of perspective and content been explored?<br>• How well has the detail and depth been demonstrated?<br>• Are responses compared and contrasted across groups/sites? | Rich ✓<br>Poor<br>Not sure/not reported | Comments: The data includes relevant conversation excerpts and photographs of work completed by the groups. |
| **10. Is the analysis reliable?**<br>For example:<br>• Did more than 1 researcher theme and code transcripts/data?<br>• If so, how were differences resolved?<br>• Did participants feed back on the transcripts/data if possible and relevant?<br>• Were negative/discrepant results addressed or ignored? | Reliable<br>Unreliable<br>Not sure/not reported ✓ | Comments: |

| | | |
|---|---|---|
| **11. Are the findings convincing?**<br><br>For example:<br><br>• Are the findings clearly presented?<br>• Are the findings internally coherent?<br>• Are extracts from the original data included?<br>• Are the data appropriately referenced?<br>• Is the reporting clear and coherent? | Convincing ✓<br><br>Not convincing<br><br>Not sure | Comments: Clear and comprehensive reporting of the findings with inclusion of extracts from the original data in the form of quotations and photographs. |
| **12. Are the findings relevant to the aims of the study?** | Relevant ✓<br><br>Irrelevant<br><br>Partially relevant | Comments: |
| **13. Conclusions**<br><br>For example:<br><br>• How clear are the links between data, interpretation and conclusions?<br>• Are the conclusions plausible and coherent?<br>• Have alternative explanations been explored and discounted?<br>• Does this enhance understanding of the research topic?<br>• Are the implications of the research clearly defined? | Adequate ✓<br><br>Inadequate<br><br>Not sure | Comments: |

| | | |
|---|---|---|
| **Is there adequate discussion of any limitations encountered?** | | |
| **Ethics** | | |
| **14. How clear and coherent is the reporting of ethics?**<br><br>For example:<br>• Have ethical issues been taken into consideration?<br>• Are they adequately discussed e.g. do they address consent and anonymity?<br>• Have the consequences of the research been considered i.e. raising expectations, changing behaviour?<br>• Was the study approved by an ethics committee? | Appropriate<br><br>Inappropriate<br><br>Not sure/not reported ✓ | Comments: No detailed consideration of ethical issues. Ethical approval was reported in the paper. |
| **Overall assessment** | | |
| **As far as can be ascertained from the paper, how well was the study conducted? (see guidance notes)** | ++<br><br>+ ✓<br><br>− | Comments: |

| | | |
|---|---|---|
| **Study identification:** Include author, title, reference, year of publication | Tollyfield, R. 2014 Facilitating an accelerated experience-based co-design project. British Journal of Nursing, 23, (3):136-141 | |
| **Guidance topic:** Co-production in acute healthcare settings. | **Key research question/aim:** Aims of the paper are stated but not the aims of the actual study. Later in the paper, it is stated that the authors explored the potential of creating and testing a 35-minute film using patient and carer narrative from a national archive of interviews held by the Health Experiences Research Group at the University of Oxford. | |
| **Checklist completed by:** | RH | |
| **Theoretical approach:** No specific theoretical framework was reported | | |
| **1. Is a qualitative approach appropriate?**<br>For example:<br>• Does the research question seek to understand processes or structures, or illuminate subjective experiences or meanings?<br>• Could a quantitative approach better have addressed the research question? | Appropriate<br>Inappropriate<br>Not sure ✓ | Comments: This paper does not report a research study specifically – there is another study by the team that is cited. This is a discussion of facilitating the EBCD process which also addressed the barriers and facilitators to using EBCD in acute healthcare settings. |
| **2. Is the study clear in what it seeks to do?**<br>For example:<br>• Is the purpose of the study discussed – aims/objectives/research question/s? | Clear<br>Unclear<br>Mixed ✓ | Comments: The study in which Tollyfield acted as facilitator for an AEBCD project is clear but not reported in this paper. |

| | | |
|---|---|---|
| • Is there adequate/appropriate reference to the literature?<br>• Are underpinning values/assumptions/theory discussed? | | |
| **Study design** | | |
| **3. How defensible/rigorous is the research design/methodology?**<br>For example:<br>• Is the design appropriate to the research question?<br>• Is a rationale given for using a qualitative approach?<br>• Are there clear accounts of the rationale/justification for the sampling, data collection and data analysis techniques used?<br>• Is the selection of cases/sampling strategy theoretically justified? | Defensible<br>Indefensible<br>Not sure ✓ | Comments: |
| **Data collection** | | |
| **4. How well was the data collection carried out?**<br>For example: | Appropriately<br>Inappropriately | Comments: |

| | | |
|---|---|---|
| • Are the data collection methods clearly described?<br>• Were the appropriate data collected to address the research question?<br>• Was the data collection and record keeping systematic? | Not sure/inadequately reported ✓ | |
| **Trustworthiness** | | |
| **5. Is the role of the researcher clearly described?**<br>For example:<br>• Has the relationship between the researcher and the participants been adequately considered?<br>• Does the paper describe how the research was explained and presented to the participants? | Clearly described<br>Unclear ✓<br>Not described | Comments: |
| **6. Is the context clearly described?**<br>For example:<br>• Are the characteristics of the participants and settings clearly defined? | Clear<br>Unclear<br>Not sure ✓ | Comments: |

| | | |
|---|---|---|
| • Were observations made in a sufficient variety of circumstances<br>• Was context bias considered | | |
| **7. Were the methods reliable?**<br>For example:<br>• Was data collected by more than 1 method?<br>• Is there justification for triangulation, or for not triangulating?<br>• Do the methods investigate what they claim to? | Reliable<br>Unreliable<br>Not sure ✓ | Comments: not reported |
| **Analysis** | | |
| **8. Is the data analysis sufficiently rigorous?**<br>For example:<br>• Is the procedure explicit – i.e. is it clear how the data was analysed to arrive at the results?<br>• How systematic is the analysis, is the procedure reliable/dependable?<br>• Is it clear how the themes and concepts | Rigorous<br>Not rigorous<br>Not sure/not reported ✓ | Comments: not reported |

| | | |
|---|---|---|
| were derived from the data? | | |
| **9. Is the data 'rich'?**<br>For example:<br>• How well are the contexts of the data described?<br>• Has the diversity of perspective and content been explored?<br>• How well has the detail and depth been demonstrated?<br>• Are responses compared and contrasted across groups/sites? | Rich<br>Poor<br>Not sure/not reported ✓ | Comments: There is a lot of useful information about facilitating EBCD |
| **10. Is the analysis reliable?**<br>For example:<br>• Did more than 1 researcher theme and code transcripts/data?<br>• If so, how were differences resolved?<br>• Did participants feed back on the transcripts/data if possible and relevant?<br>• Were negative/discrepant results addressed or ignored? | Reliable<br>Unreliable<br>Not sure/not reported ✓ | Comments: Not much detail given |

| | | |
|---|---|---|
| **11. Are the findings convincing?**<br><br>For example:<br><br>• Are the findings clearly presented?<br>• Are the findings internally coherent?<br>• Are extracts from the original data included?<br>• Are the data appropriately referenced?<br>• Is the reporting clear and coherent? | Convincing ✓<br><br>Not convincing<br><br>Not sure | Comments: The findings aren't really 'findings' as such. It is a discussion of implementing AEBCD. The information is convincing in terms of what we learn about the challenges and facilitators of using AEBCD |
| **12. Are the findings relevant to the aims of the study?** | Relevant ✓<br><br>Irrelevant<br><br>Partially relevant | Comments: |
| **13. Conclusions**<br><br>For example:<br><br>• How clear are the links between data, interpretation and conclusions?<br>• Are the conclusions plausible and coherent?<br>• Have alternative explanations been explored and discounted?<br>• Does this enhance understanding of the research topic? | Adequate ✓<br><br>Inadequate<br><br>Not sure | Comments: |

| | | |
|---|---|---|
| • Are the implications of the research clearly defined?<br>**Is there adequate discussion of any limitations encountered?** | | |
| **Ethics** | | |
| **14. How clear and coherent is the reporting of ethics?**<br>For example:<br>• Have ethical issues been taken into consideration?<br>• Are they adequately discussed e.g. do they address consent and anonymity?<br>• Have the consequences of the research been considered i.e. raising expectations, changing behaviour?<br>• Was the study approved by an ethics committee? | Appropriate<br>Inappropriate<br>Not sure/not reported ✓ | Comments: |
| **Overall assessment** | | |
| **As far as can be ascertained from the paper, how well was the study conducted? (see guidance notes)** | ++<br>**+** ✓<br>- | Comments: Reports on facilitation of the Locock et al (2014) study. Provides a different perspective in focusing on the author's experiences and reflections on facilitating an EBCD process. The paper does not report the research aspects of the Locock et al study. |

| | | |
|---|---|---|
| **Study identification:** Include author, title, reference, year of publication | Tollyfield, R. 2014 Facilitating an accelerated experience-based co-design project. British Journal of Nursing, 23, (3):136-141 | |
| **Guidance topic:** Co-production in acute healthcare settings. | **Key research question/aim:** Aims of the paper are stated but not the aims of the actual study. | |
| **Checklist completed by:** | DJC | |
| **Theoretical approach:** No specific theoretical framework was reported | | |
| **1. Is a qualitative approach appropriate?**<br>For example:<br>• Does the research question seek to understand processes or structures, or illuminate subjective experiences or meanings?<br>• Could a quantitative approach better have addressed the research question? | Appropriate<br>Inappropriate<br>Not sure ✓ | Comments: This is a personal reflection and description of facilitating an AEBCD project. The value in the CREATE RES is that the paper directly addresses the barriers and facilitators to using AEBCD/EBCD in acute healthcare settings. |
| **2. Is the study clear in what it seeks to do?**<br>For example:<br>• Is the purpose of the study discussed – aims/objectives/research question/s?<br>• Is there adequate/appropriate reference to the literature? | Clear<br>Unclear<br>Mixed ✓ | Comments: The study in which Tollyfield acted as AEBCD facilitator is clear but the research conducted is not the focus of this paper. |

| | | |
|---|---|---|
| • Are underpinning values/assumptions/theory discussed? | | |
| **Study design** | | |
| **3. How defensible/rigorous is the research design/methodology?**<br>For example:<br>• Is the design appropriate to the research question?<br>• Is a rationale given for using a qualitative approach?<br>• Are there clear accounts of the rationale/justification for the sampling, data collection and data analysis techniques used?<br>• Is the selection of cases/sampling strategy theoretically justified? | Defensible<br>Indefensible<br>Not sure ✓ | Comments: |
| **Data collection** | | |
| **4. How well was the data collection carried out?**<br>For example:<br>• Are the data collection methods clearly described? | Appropriately<br>Inappropriately<br>Not sure/inadequately | Comments: |

| | | |
|---|---|---|
| • Were the appropriate data collected to address the research question?<br><br>• Was the data collection and record keeping systematic? | reported ✓ | |
| **Trustworthiness** | | |
| **5. Is the role of the researcher clearly described?**<br>For example:<br><br>• Has the relationship between the researcher and the participants been adequately considered?<br><br>• Does the paper describe how the research was explained and presented to the participants? | Clearly described<br>Unclear ✓<br>Not described | Comments: |
| **6. Is the context clearly described?**<br>For example:<br><br>• Are the characteristics of the participants and settings clearly defined?<br><br>• Were observations made in a sufficient variety of circumstances | Clear<br>Unclear<br>Not sure ✓ | Comments: |

| | | |
|---|---|---|
| • Was context bias considered | | |
| **7. Were the methods reliable?**<br>For example:<br>• Was data collected by more than 1 method?<br>• Is there justification for triangulation, or for not triangulating?<br>• Do the methods investigate what they claim to? | Reliable<br>Unreliable<br>Not sure ✓ | Comments: |
| **Analysis** | | |
| **8. Is the data analysis sufficiently rigorous?**<br>For example:<br>• Is the procedure explicit – i.e. is it clear how the data was analysed to arrive at the results?<br>• How systematic is the analysis, is the procedure reliable/dependable?<br>• Is it clear how the themes and concepts were derived from the data? | Rigorous<br>Not rigorous<br>Not sure/not reported ✓ | Comments: |
| **9. Is the data 'rich'?** | Rich | Comments: Provides a range of information about facilitating AEBCD/EBCD projects in |

| | | |
|---|---|---|
| For example:<br>• How well are the contexts of the data described?<br>• Has the diversity of perspective and content been explored?<br>• How well has the detail and depth been demonstrated?<br>• Are responses compared and contrasted across groups/sites? | Poor<br>Not sure/not reported ✓ | practice. |
| **10. Is the analysis reliable?**<br>For example:<br>• Did more than 1 researcher theme and code transcripts/data?<br>• If so, how were differences resolved?<br>• Did participants feed back on the transcripts/data if possible and relevant?<br>• Were negative/discrepant results addressed or ignored? | Reliable<br>Unreliable<br>Not sure/not reported ✓ | Comments: |
| **11. Are the findings convincing?**<br>For example:<br>• Are the findings clearly presented? | Convincing ✓<br>Not convincing<br>Not sure | Comments: Does not report research findings per se but rather the authors experiences in facilitating an AEBCD project. |

| | | |
|---|---|---|
| • Are the findings internally coherent?<br>• Are extracts from the original data included?<br>• Are the data appropriately referenced?<br>• Is the reporting clear and coherent? | | |
| **12. Are the findings relevant to the aims of the study?** | Relevant ✓<br>Irrelevant<br>Partially relevant | Comments: Very useful information on barriers and facilitators to AEBCD/EBCD in an acute care setting. |
| **13. Conclusions**<br>For example:<br>• How clear are the links between data, interpretation and conclusions?<br>• Are the conclusions plausible and coherent?<br>• Have alternative explanations been explored and discounted?<br>• Does this enhance understanding of the research topic?<br>• Are the implications of the research clearly defined?<br>**Is there adequate discussion of any limitations** | Adequate ✓<br>Inadequate<br>Not sure | Comments: |

| | | |
|---|---|---|
| **encountered?** | | |
| **Ethics** | | |
| **14. How clear and coherent is the reporting of ethics?**<br>For example:<br>• Have ethical issues been taken into consideration?<br>• Are they adequately discussed e.g. do they address consent and anonymity?<br>• Have the consequences of the research been considered i.e. raising expectations, changing behaviour?<br>• Was the study approved by an ethics committee? | Appropriate<br>Inappropriate<br>Not sure/not reported ✓ | Comments: |
| **Overall assessment** | | |
| **As far as can be ascertained from the paper, how well was the study conducted? (see guidance notes)** | ++<br>**+** ✓<br>- | Comments: Reports on facilitation of the Locock et al (2014) study but focuses on Tollyfield's experiences and reflections in facilitating an EBCD process. |

| | | |
|---|---|---|
| **Study identification:** Include author, title, reference, year of publication | Tsianakas,V et al, 2012 Implementing patient-centred cancer care: using experience-based co-design to improve patient experience in breast and lung cancer services. Supportive Care in Cancer (2012) 20:2639–2647 | |
| **Guidance topic:** Co-production in acute healthcare settings. | **Key research question/aim:** The aims of this paper are to describe the process by which patients and staff in these two services identified and implemented improvements in patient experience before (1) comparing the issues identified as shaping patient experiences in the different tumour groups and (2) exploring participants' reflections on the value and key characteristics of this approach to improving patient experiences. | |
| **Checklist completed by:** | RH | |
| **Theoretical approach:** No specific theoretical framework was reported | | |
| **1. Is a qualitative approach appropriate?**<br>For example:<br>• Does the research question seek to understand processes or structures, or illuminate subjective experiences or meanings?<br>• Could a quantitative approach better have addressed the research question? | Appropriate ✓<br>Inappropriate<br>Not sure | Comments: |
| **2. Is the study clear in what it seeks to do?**<br>For example:<br>• Is the purpose of the study discussed – aims/objectives/research question/s? | Clear✓<br>Unclear<br>Mixed | Comments: |

| | | |
|---|---|---|
| • Is there adequate/appropriate reference to the literature?<br>• Are underpinning values/assumptions/theory discussed? | | |
| **Study design** | | |
| **3. How defensible/rigorous is the research design/methodology?**<br>For example:<br>• Is the design appropriate to the research question?<br>• Is a rationale given for using a qualitative approach?<br>• Are there clear accounts of the rationale/justification for the sampling, data collection and data analysis techniques used?<br>• Is the selection of cases/sampling strategy theoretically justified? | Defensible ✓<br>Indefensible<br>Not sure | Comments: |
| **Data collection** | | |
| **4. How well was the data collection carried out?**<br>For example:<br>• Are the data collection methods clearly described?<br>• Were the appropriate data collected to address the | Appropriately ✓<br>Inappropriately<br>Not sure/inadequately reported | Comments: |

| | | |
|---|---|---|
| research question?<br>• Was the data collection and record keeping systematic? | | |
| **Trustworthiness** | | |
| **5. Is the role of the researcher clearly described?**<br>For example:<br>• Has the relationship between the researcher and the participants been adequately considered?<br>• Does the paper describe how the research was explained and presented to the participants? | Clearly described✓<br>Unclear<br>Not described | Comments: |
| **6. Is the context clearly described?**<br>For example:<br>• Are the characteristics of the participants and settings clearly defined?<br>• Were observations made in a sufficient variety of circumstances<br>• Was context bias considered | Clear<br>Unclear✓<br>Not sure | Comments: |

| | | |
|---|---|---|
| **7. Were the methods reliable?**<br>For example:<br>• Was data collected by more than 1 method?<br>• Is there justification for triangulation, or for not triangulating?<br>• Do the methods investigate what they claim to? | Reliable ✓<br>Unreliable<br>Not sure | Comments: |
| **Analysis** | | |
| **8. Is the data analysis sufficiently rigorous?**<br>For example:<br>• Is the procedure explicit – i.e. is it clear how the data was analysed to arrive at the results?<br>• How systematic is the analysis, is the procedure reliable/dependable?<br>• Is it clear how the themes and concepts were derived from the data? | Rigorous ✓<br>Not rigorous<br>Not sure/not reported | Comments: |
| **9. Is the data 'rich'?**<br>For example:<br>• How well are the contexts of the data described?<br>• Has the diversity of perspective and content been explored? | Rich ✓<br>Poor<br>Not sure/not reported | Comments: |

| | | |
|---|---|---|
| • How well has the detail and depth been demonstrated?<br>• Are responses compared and contrasted across groups/sites? | | |
| **10. Is the analysis reliable?**<br>For example:<br>• Did more than 1 researcher theme and code transcripts/data?<br>• If so, how were differences resolved?<br>• Did participants feed back on the transcripts/data if possible and relevant?<br>• Were negative/discrepant results addressed or ignored? | Reliable ✓<br>Unreliable<br>Not sure/not reported | Comments: |
| **11. Are the findings convincing?**<br>For example:<br>• Are the findings clearly presented?<br>• Are the findings internally coherent?<br>• Are extracts from the original data included?<br>• Are the data appropriately referenced?<br>• Is the reporting clear and coherent? | Convincing ✓<br>Not convincing<br>Not sure | Comments: |

| | | |
|---|---|---|
| **12. Are the findings relevant to the aims of the study?** | Relevant ✓<br>Irrelevant<br>Partially relevant | Comments: |
| **13. Conclusions**<br>For example:<br>• How clear are the links between data, interpretation and conclusions?<br>• Are the conclusions plausible and coherent?<br>• Have alternative explanations been explored and discounted?<br>• Does this enhance understanding of the research topic?<br>• Are the implications of the research clearly defined?<br>**Is there adequate discussion of any limitations encountered?** | Adequate ✓<br>Inadequate<br>Not sure | Comments: |
| **Ethics** | | |
| **14. How clear and coherent is the reporting of ethics?**<br>For example:<br>• Have ethical issues been taken into consideration?<br>• Are they adequately discussed e.g. do they address consent and anonymity? | Appropriate<br>Inappropriate<br>Not sure/not reported ✓ | Comments: Not addressed in the paper |

| | | |
|---|---|---|
| • Have the consequences of the research been considered i.e. raising expectations, changing behaviour?<br>• Was the study approved by an ethics committee? | | |
| **Overall assessment** | | |
| **As far as can be ascertained from the paper, how well was the study conducted? (see guidance notes)** | ++ ✓<br>**+**<br>− | Comments: |

| | |
|---|---|
| **Study identification:** Include author, title, reference, year of publication | Tsianakas,V et al, 2012 Implementing patient-centred cancer care: using experience-based co-design to improve patient experience in breast and lung cancer services. Supportive Care in Cancer (2012) 20:2639–2647 |
| **Guidance topic:** Co-production in acute healthcare settings. | **Key research question/aim:**<br><br>To use EBCD to enhance experiences for breast and lung cancer service patients; paper briefly reports on the process which led to service improvement s. But main questions focus on comparing issues identified as shaping experience in the different tumour groups 2) exploring participants' reflections on the value and key characteristics of the approach to improving patient experiences |
| **Checklist completed by:** | FJ |
| **Theoretical approach:** evaluation of existing EBCD improvement projects; no other details about theoretical approach | |

| | | |
|---|---|---|
| **1. Is a qualitative approach appropriate?**<br>For example:<br>• Does the research question seek to understand processes or structures, or illuminate subjective experiences or meanings?<br>• Could a quantitative approach better have addressed the research question? | Appropriate ✓<br>Inappropriate<br>Not sure | Comments: qualitative approach part of EBCD (observations and interviews) |
| **2. Is the study clear in what it seeks to do?**<br>For example:<br>• Is the purpose of the study discussed – aims/objectives/research question/s?<br>• Is there adequate/appropriate reference to the literature?<br>• Are underpinning values/assumptions/theory discussed? | Clear ✓<br>Unclear<br>Mixed | Comments: |
| **Study design** | | |
| **3. How defensible/rigorous is the research design/methodology?**<br>For example:<br>• Is the design appropriate to the | Defensible<br>Indefensible<br>Not sure ✓ | Comments:<br>Not much detail about sampling. Selection of cases not justified. |

| | | |
|---|---|---|
| research question?<br>• Is a rationale given for using a qualitative approach?<br>• Are there clear accounts of the rationale/justification for the sampling, data collection and data analysis techniques used?<br>• Is the selection of cases/sampling strategy theoretically justified? | | |
| **Data collection** | | |
| **4. How well was the data collection carried out?**<br>For example:<br>• Are the data collection methods clearly described?<br>• Were the appropriate data collected to address the research question?<br>• Was the data collection and record keeping systematic? | Appropriately ✓<br>Inappropriately<br>Not sure/inadequately reported | Comments: |
| **Trustworthiness** | | |

| | | |
|---|---|---|
| **5. Is the role of the researcher clearly described?**<br>For example:<br>• Has the relationship between the researcher and the participants been adequately considered?<br>• Does the paper describe how the research was explained and presented to the participants? | Clearly described<br>Unclear ✓<br>Not described | Comments: two researchers involved in viewing films independently and sharing understanding of touchpoints; 2 researchers (including 1 different researcher) developed composite films. All 3 researchers were involved with observations, interviews and analysis in some way - but relationship and explanations not given |
| **6. Is the context clearly described?**<br>For example:<br>• Are the characteristics of the participants and settings clearly defined?<br>• Were observations made in a sufficient variety of circumstances<br>• Was context bias considered | Clear ✓<br>Unclear<br>Not sure | Comments: no comment on content bias |
| **7. Were the methods reliable?**<br>For example:<br>• Was data collected by more than 1 method? | Reliable ✓<br>Unreliable<br>Not sure | Comments: |

| | | |
|---|---|---|
| • Is there justification for triangulation, or for not triangulating?<br>• Do the methods investigate what they claim to? | | |
| **Analysis** | | |
| **8. Is the data analysis sufficiently rigorous?**<br>For example:<br>• Is the procedure explicit – i.e. is it clear how the data was analysed to arrive at the results?<br>• How systematic is the analysis, is the procedure reliable/dependable?<br>• Is it clear how the themes and concepts were derived from the data? | Rigorous<br>Not rigorous<br>Not sure/not ✓reported | Comments:<br>Not clear how observational data were managed and analysed |
| **9. Is the data 'rich'?**<br>For example:<br>• How well are the contexts of the data described?<br>• Has the diversity of perspective and content been explored?<br>• How well has the detail and depth been | Rich ✓<br>Poor<br>Not sure/not reported | Comments: |

| | | |
|---|---|---|
| demonstrated?<br>• Are responses compared and contrasted across groups/sites? | | |
| **10. Is the analysis reliable?**<br>For example:<br>• Did more than 1 researcher theme and code transcripts/data?<br>• If so, how were differences resolved?<br>• Did participants feed back on the transcripts/data if possible and relevant?<br>• Were negative/discrepant results addressed or ignored? | Reliable<br>Unreliable<br>Not sure/not reported ✓ | Comments: no comment about how differences were resolved in thematic analysis ; participant feedback was an implicit part of the staff, patient groups |
| **11. Are the findings convincing?**<br>For example:<br>• Are the findings clearly presented?<br>• Are the findings internally coherent?<br>• Are extracts from the original data included?<br>• Are the data appropriately referenced? | Convincing ✓<br>Not convincing<br>Not sure | Comments:<br>Finding reported clearly but as a commentary rather than using a traditional way with themes/sub-themes –illustrative quotes etc. |

| | | |
|---|---|---|
| • Is the reporting clear and coherent? | | |
| **12. Are the findings relevant to the aims of the study?** | Relevant ✓<br>Irrelevant<br>Partially relevant | Comments: |
| **13. Conclusions**<br>For example:<br>• How clear are the links between data, interpretation and conclusions?<br>• Are the conclusions plausible and coherent?<br>• Have alternative explanations been explored and discounted?<br>• Does this enhance understanding of the research topic?<br>• Are the implications of the research clearly defined?<br>**Is there adequate discussion of any limitations encountered?** | Adequate ✓<br>Inadequate<br>Not sure | Comments: |
| **Ethics** | | |
| **14. How clear and coherent is the reporting** | Appropriate | Comments: |

| | | |
|---|---|---|
| **of ethics?**<br>For example:<br>• Have ethical issues been taken into consideration?<br>• Are they adequately discussed e.g. do they address consent and anonymity?<br>• Have the consequences of the research been considered i.e. raising expectations, changing behaviour?<br>• Was the study approved by an ethics committee? | Inappropriate<br>Not sure/not reported ✓ | No mention of ethical approval |
| **Overall assessment** | | |
| **As far as can be ascertained from the paper, how well was the study conducted? (see guidance notes)** | +<br>Following independent third review agreed to be ++ | Comments: |

***For this study which adopted a mixed methods approach, the CASP RCT checklist was used as well as the NICE (2012) qualitative research approval tool.** See http://media.wix.com/ugd/dded87_40b9ff0bf53840478331915a8ed8b2fb.pdf
The RCT was small scale and exploratory, nonetheless we regarded the quality, rigour and reporting of the study to be satisfactory to good.

| | | |
|---|---|---|
| **Study identification:** Include author, title, reference, year of publication | Enhancing the experience of carers in the chemotherapy outpatient setting: an exploratory randomised controlled trial to test impact, acceptability and feasibility of a complex intervention co-designed by carers and staff V. Tsianakas & G. Robert & A. Richardson & R. Verity & C. Oakley & T. Murrells & M. Flynn & E. Ream. Support Care Cancer. 2015. DOI 10.1007/s00520-015-2677-x | |
| **Guidance topic:** | **Key research question/aim:** To test the feasibility and acceptability of a complex intervention for carers that was co-designed by staff and carers of patients starting chemotherapy. | |
| **Checklist completed by:** | SH | |
| **Theoretical approach** | | |
| **1. Is a qualitative approach appropriate?**<br><br>For example:<br><br>• Does the research question seek to understand processes or structures, or illuminate subjective experiences or meanings?<br><br>• Could a quantitative approach better have addressed the research question? | Appropriate ✓<br><br>Inappropriate<br><br>Not sure | Comments: Appropriate as the study seeks to elicit the subjective views and meanings of carers and staff. A quantitative approach could not accomplish this. |
| **2. Is the study clear in what it seeks to do?**<br><br>For example: | Clear ✓<br><br>Unclear | Comments: The aims are discussed and are clear. There is adequate reference to the literature and underpinning theories, including international studies examining the information needs of cancer |

| | | |
|---|---|---|
| • Is the purpose of the study discussed – aims/objectives/research question/s?<br>• Is there adequate/appropriate reference to the literature?<br>• Are underpinning values/assumptions/theory discussed? | Mixed | patients. |
| **Study design** | | |
| **3. How defensible/rigorous is the research design/methodology?**<br>For example:<br>• Is the design appropriate to the research question?<br>• Is a rationale given for using a qualitative approach?<br>• Are there clear accounts of the rationale/justification for the sampling, data collection and data analysis techniques used?<br>• Is the selection of cases/sampling strategy theoretically justified? | Defensible ✓<br>Indefensible<br>Not sure | Comments: The design is appropriate for a feasibility and acceptability study. The rationale for the sampling and data collection is discussed. Carers of patients with lung, breast and colon cancer were chosen as they are among the most common cancers in the UK. |
| **Data collection** | | |
| **4. How well was the data collection carried out?**<br>For example: | Appropriately ✓<br>Inappropriately | Comments: the focus groups are clearly described and were appropriate for the research question. |

| | | |
|---|---|---|
| • Are the data collection methods clearly described?<br>• Were the appropriate data collected to address the research question?<br>• Was the data collection and record keeping systematic? | Not sure/inadequately reported | |
| **Trustworthiness** | | |
| **5. Is the role of the researcher clearly described?**<br><br>For example:<br>• Has the relationship between the researcher and the participants been adequately considered?<br>• Does the paper describe how the research was explained and presented to the participants? | Clearly described<br><br>Unclear ✓<br><br>Not described | Comments: Not described in detail. |
| **6. Is the context clearly described?**<br><br>For example:<br>• Are the characteristics of the participants and settings clearly defined?<br>• Were observations made in a sufficient variety of circumstances<br>• Was context bias considered | Clear ✓<br><br>Unclear<br><br>Not sure | Comments: The outpatient setting was clearly described. |

| | | |
|---|---|---|
| **7. Were the methods reliable?**<br><br>For example:<br><br>• Was data collected by more than 1 method?<br><br>• Is there justification for triangulation, or for not triangulating?<br><br>• Do the methods investigate what they claim to? | Reliable ✓<br><br>Unreliable<br><br>Not sure | Comments: The study described here is the qualitative part of a feasibility trial that included questionnaires and focus groups. The methods do investigate what they claim to. |
| **Analysis** | | |
| **8. Is the data analysis sufficiently rigorous?**<br><br>For example:<br><br>• Is the procedure explicit – i.e. is it clear how the data was analysed to arrive at the results?<br><br>• How systematic is the analysis, is the procedure reliable/dependable?<br><br>• Is it clear how the themes and concepts were derived from the data? | Rigorous<br><br>Not rigorous<br><br>Not sure/not reported ✓ | Comments: The method of analysis is only briefly described without sufficient detail to make a judgement about its rigour. |
| **9. Is the data 'rich'?**<br><br>For example:<br><br>• How well are the contexts of the data described?<br><br>• Has the diversity of perspective and content been explored?<br><br>• How well has the detail and depth been | Rich ✓<br><br>Poor<br><br>Not sure/not reported | Comments: the data is 'rich' in that it was exploring the acceptably of an intervention as part of a small feasibility study i.e. it set out what it aimed to do within the confines of the study. |

| | | |
|---|---|---|
| demonstrated?<br>• Are responses compared and contrasted across groups/sites? | | |
| **10. Is the analysis reliable?**<br>For example:<br>• Did more than 1 researcher theme and code transcripts/data?<br>• If so, how were differences resolved?<br>• Did participants feed back on the transcripts/data if possible and relevant?<br>• Were negative/discrepant results addressed or ignored? | Reliable<br>Unreliable<br>Not sure/not reported ✓ | Comments: The qualitative analysis was not reported in any detail. |
| **11. Are the findings convincing?**<br>For example:<br>• Are the findings clearly presented?<br>• Are the findings internally coherent?<br>• Are extracts from the original data included?<br>• Are the data appropriately referenced?<br>• Is the reporting clear and coherent? | Convincing ✓<br>Not convincing<br>Not sure | Comments: The findings are convincing in the context of a small feasibility study. The weaknesses are cited as the small size and the fact that the study was conducted at only one centre. |
| **12. Are the findings relevant to the aims of the study?** | Relevant ✓ | Comments: Yes the findings indicate that the intervention was acceptable. |

| | | |
|---|---|---|
| | Irrelevant<br><br>Partially relevant | |
| **13. Conclusions**<br><br>For example:<br><br>• How clear are the links between data, interpretation and conclusions?<br><br>• Are the conclusions plausible and coherent?<br><br>• Have alternative explanations been explored and discounted?<br><br>• Does this enhance understanding of the research topic?<br><br>• Are the implications of the research clearly defined?<br><br>**Is there adequate discussion of any limitations encountered?** | Adequate ✓<br><br>Inadequate<br><br>Not sure | Comments: the conclusions are plausible and indicate further research would be valuable.<br><br>The weaknesses are cited as the small size and the fact that the study was conducted at only one centre. |
| **Ethics** | | |
| **14. How clear and coherent is the reporting of ethics?**<br><br>For example:<br><br>• Have ethical issues been taken into consideration?<br><br>• Are they adequately discussed e.g. do they address consent and anonymity? | Appropriate<br><br>Inappropriate<br><br>Not sure/not reported ✓ | Comments: The study had ethical approval. No other details were reported. |

| | | |
|---|---|---|
| • Have the consequences of the research been considered i.e. raising expectations, changing behaviour?<br><br>• Was the study approved by an ethics committee? | | |
| **Overall assessment** | | |
| **As far as can be ascertained from the paper, how well was the study conducted? (see guidance notes)** | ++ ✓<br><br>+<br><br>− | Comments: |

| | |
|---|---|
| **Study identification:** Include author, title, reference, year of publication | Tsianakas,V. Robert,G. Richardson,A., Verity,R., Oakley,C. Murrells,T., Flynn,M., Ream,E. 2015 Enhancing the experience of carers in the chemotherapy outpatient setting: an exploratory randomised controlled trial to test impact, acceptability and feasibility of a complex intervention co-designed by carers and staff Ream. Support Care Cancer. DOI 10.1007/s00520-015-2677-x |
| **Guidance topic:** | **Key research question/aim:** To test the feasibility and acceptability of a complex intervention for carers that was co-designed by staff and carers of patients starting chemotherapy. |
| **Checklist completed by:** | DJC |
| **Theoretical approach:** Not reported, though locates the feasibility RCT in the context of the MRC guidance for development of complex interventions. | |

| | | |
|---|---|---|
| **1. Is a qualitative approach appropriate?**<br><br>For example:<br><br>• Does the research question seek to understand processes or structures, or illuminate subjective experiences or meanings?<br><br>• Could a quantitative approach better have addressed the research question? | Appropriate ✓<br><br>Inappropriate<br><br>Not sure | Comments: |
| **2. Is the study clear in what it seeks to do?**<br><br>For example:<br><br>• Is the purpose of the study discussed – aims/objectives/research question/s?<br><br>• Is there adequate/appropriate reference to the literature?<br><br>• Are underpinning values/assumptions/theory discussed? | Clear ✓<br><br>Unclear<br><br>Mixed | Comments: |
| **Study design** | | |
| **3. How defensible/rigorous is the research design/methodology?**<br><br>For example:<br><br>• Is the design appropriate to the research question?<br><br>• Is a rationale given for using a qualitative | Defensible ✓<br><br>Indefensible<br><br>Not sure | Comments: |

| | | |
|---|---|---|
| approach?<br>• Are there clear accounts of the rationale/justification for the sampling, data collection and data analysis techniques used?<br>• Is the selection of cases/sampling strategy theoretically justified? | | |
| **Data collection** | | |
| **4. How well was the data collection carried out?**<br>For example:<br>• Are the data collection methods clearly described?<br>• Were the appropriate data collected to address the research question?<br>• Was the data collection and record keeping systematic? | Appropriately ✓<br>Inappropriately<br>Not sure/inadequately reported | Comments: |
| **Trustworthiness** | | |
| **5. Is the role of the researcher clearly described?**<br>For example:<br>• Has the relationship between the researcher and the participants been adequately considered?<br>• Does the paper describe how the research was | Clearly described<br>Unclear ✓<br>Not described | Comments: Not described. |

| | | |
|---|---|---|
| explained and presented to the participants? | | |
| **6. Is the context clearly described?**<br><br>For example:<br><br>• Are the characteristics of the participants and settings clearly defined?<br><br>• Were observations made in a sufficient variety of circumstances<br><br>• Was context bias considered | Clear ✓<br><br>Unclear<br><br>Not sure | Comments: |
| **7. Were the methods reliable?**<br><br>For example:<br><br>• Was data collected by more than 1 method?<br><br>• Is there justification for triangulation, or for not triangulating?<br><br>• Do the methods investigate what they claim to? | Reliable ✓<br><br>Unreliable<br><br>Not sure | Comments: The study described is the qualitative part of a feasibility trial that included questionnaires and focus groups. |
| **Analysis** | | |
| **8. Is the data analysis sufficiently rigorous?**<br><br>For example:<br><br>• Is the procedure explicit – i.e. is it clear how the data was analysed to arrive at the results?<br><br>• How systematic is the analysis, is the procedure | Rigorous<br><br>Not rigorous<br><br>Not sure/not reported ✓ | Comments: |

| | | |
|---|---|---|
| reliable/dependable?<br>• Is it clear how the themes and concepts were derived from the data? | | |
| **9. Is the data 'rich'?**<br>For example:<br>• How well are the contexts of the data described?<br>• Has the diversity of perspective and content been explored?<br>• How well has the detail and depth been demonstrated?<br>• Are responses compared and contrasted across groups/sites? | Rich ✓<br>Poor<br>Not sure/not reported | Comments |
| **10. Is the analysis reliable?**<br>For example:<br>• Did more than 1 researcher theme and code transcripts/data?<br>• If so, how were differences resolved?<br>• Did participants feed back on the transcripts/data if possible and relevant?<br>• Were negative/discrepant results addressed or ignored? | Reliable<br>Unreliable<br>Not sure/not reported ✓ | Comments: Very limited reporting of analysis. |

| | | |
|---|---|---|
| **11. Are the findings convincing?**<br><br>For example:<br><br>• Are the findings clearly presented?<br>• Are the findings internally coherent?<br>• Are extracts from the original data included?<br>• Are the data appropriately referenced?<br>• Is the reporting clear and coherent? | Convincing ✓<br><br>Not convincing<br><br>Not sure | Comments: |
| **12. Are the findings relevant to the aims of the study?** | Relevant ✓<br><br>Irrelevant<br><br>Partially relevant | Comments: |
| **13. Conclusions**<br><br>For example:<br><br>• How clear are the links between data, interpretation and conclusions?<br>• Are the conclusions plausible and coherent?<br>• Have alternative explanations been explored and discounted?<br>• Does this enhance understanding of the research topic?<br>• Are the implications of the research clearly defined? | Adequate ✓<br><br>Inadequate<br><br>Not sure | Comments: |

| | | |
|---|---|---|
| **Is there adequate discussion of any limitations encountered?** | | |
| **Ethics** | | |
| **14. How clear and coherent is the reporting of ethics?**<br><br>For example:<br><br>• Have ethical issues been taken into consideration?<br><br>• Are they adequately discussed e.g. do they address consent and anonymity?<br><br>• Have the consequences of the research been considered i.e. raising expectations, changing behaviour?<br><br>• Was the study approved by an ethics committee? | Appropriate<br><br>Inappropriate<br><br>Not sure/not reported ✓ | Comments: Ethical approval is reported. |
| **Overall assessment** | | |
| **As far as can be ascertained from the paper, how well was the study conducted? (see guidance notes)** | ++ ✓<br><br>+<br><br>− | Comments: |

| | | |
|---|---|---|
| **Study identification:** Include author, title, reference, year of publication | Vennik et al, Co-production in healthcare: rhetoric and practice. International Review of Administrative Sciences. 82(1) 150–168 | |
| **Guidance topic:** Co-production in acute healthcare settings. | **Key research question/aim:** Why are hospitals interested in co-production activities and what are the experiences with co-production in practice? | |
| **Checklist completed by:** | RH | |
| **Theoretical approach:** No specific theoretical framework was reported | | |
| **1. Is a qualitative approach appropriate?**<br>For example:<br>• Does the research question seek to understand processes or structures, or illuminate subjective experiences or meanings?<br>• Could a quantitative approach better have addressed the research question? | Appropriate ✓<br>Inappropriate<br>Not sure | Comments: |
| **2. Is the study clear in what it seeks to do?**<br>For example:<br>• Is the purpose of the study discussed – aims/objectives/research question/s?<br>• Is there adequate/appropriate reference to the literature?<br>• Are underpinning values/assumptions/theory discussed? | Clear✓<br>Unclear<br>Mixed | Comments: |

| Study design | | |
|---|---|---|
| **3. How defensible/rigorous is the research design/methodology?**<br>For example:<br>• Is the design appropriate to the research question?<br>• Is a rationale given for using a qualitative approach?<br>• Are there clear accounts of the rationale/justification for the sampling, data collection and data analysis techniques used?<br>• Is the selection of cases/sampling strategy theoretically justified? | Defensible ✓<br>Indefensible<br>Not sure | Comments: |
| **Data collection** | | |
| **4. How well was the data collection carried out?**<br>For example:<br>• Are the data collection methods clearly described?<br>• Were the appropriate data collected to address the research question?<br>• Was the data collection and record keeping systematic? | Appropriately ✓<br>Inappropriately<br>Not sure/inadequately reported | Comments: |
| **Trustworthiness** | | |

| | | |
|---|---|---|
| **5. Is the role of the researcher clearly described?**<br>For example:<br>• Has the relationship between the researcher and the participants been adequately considered?<br>• Does the paper describe how the research was explained and presented to the participants? | Clearly described✓<br>Unclear<br>Not described | Comments: |
| **6. Is the context clearly described?**<br>For example:<br>• Are the characteristics of the participants and settings clearly defined?<br>• Were observations made in a sufficient variety of circumstances<br>• Was context bias considered | Clear ✓<br>Unclear<br>Not sure | Comments: |
| **7. Were the methods reliable?**<br>For example:<br>• Was data collected by more than 1 method?<br>• Is there justification for triangulation, or for not triangulating?<br>• Do the methods investigate what they claim to? | Reliable ✓<br>Unreliable<br>Not sure | Comments: |

| Analysis | | |
|---|---|---|
| **8. Is the data analysis sufficiently rigorous?**<br>For example:<br>• Is the procedure explicit – i.e. is it clear how the data was analysed to arrive at the results?<br>• How systematic is the analysis, is the procedure reliable/dependable?<br>• Is it clear how the themes and concepts were derived from the data? | Rigorous ✓<br>Not rigorous<br>Not sure/not reported | Comments: |
| **9. Is the data 'rich'?**<br>For example:<br>• How well are the contexts of the data described?<br>• Has the diversity of perspective and content been explored?<br>• How well has the detail and depth been demonstrated?<br>• Are responses compared and contrasted across groups/sites? | Rich ✓<br>Poor<br>Not sure/not reported | Comments: |
| **10. Is the analysis reliable?**<br>For example:<br>• Did more than 1 researcher theme and code transcripts/data?<br>• If so, how were differences resolved?<br>• Did participants feed back on the transcripts/data if possible | Reliable ✓<br>Unreliable<br>Not sure/not reported | Comments: |

| | | |
|---|---|---|
| and relevant?<br>• Were negative/discrepant results addressed or ignored? | | |
| **11. Are the findings convincing?**<br>For example:<br>• Are the findings clearly presented?<br>• Are the findings internally coherent?<br>• Are extracts from the original data included?<br>• Are the data appropriately referenced?<br>• Is the reporting clear and coherent? | Convincing ✓<br>Not convincing<br>Not sure | Comments: |
| **12. Are the findings relevant to the aims of the study?** | Relevant ✓<br>Irrelevant<br>Partially relevant | Comments: |
| **13. Conclusions**<br>For example:<br>• How clear are the links between data, interpretation and conclusions?<br>• Are the conclusions plausible and coherent?<br>• Have alternative explanations been explored and discounted?<br>• Does this enhance understanding of the research topic? | Adequate ✓<br>Inadequate<br>Not sure | Comments: |

| | | |
|---|---|---|
| • Are the implications of the research clearly defined?<br>**Is there adequate discussion of any limitations encountered?** | | |
| **Ethics** | | |
| **14. How clear and coherent is the reporting of ethics?**<br>For example:<br>• Have ethical issues been taken into consideration?<br>• Are they adequately discussed e.g. do they address consent and anonymity?<br>• Have the consequences of the research been considered i.e. raising expectations, changing behaviour?<br>• Was the study approved by an ethics committee? | Appropriate<br>Inappropriate<br>Not sure/not reported ✓ | Comments: Not addressed in the paper |
| **Overall assessment** | | |
| **As far as can be ascertained from the paper, how well was the study conducted? (see guidance notes)** | ++ ✓<br>**+**<br>− | Comments: |

| | | |
|---|---|---|
| **Study identification:** Include author, title, reference, year of publication | Vennik et al, 2016 Co-production in healthcare: rhetoric and practice. International Review of Administrative Sciences, 82(1) 150–168 | |
| **Guidance topic:** | **Key research question/aim:** See below, refers to the aim of the paper and not directly to the aim of the study directly. Does state: The article focuses on (research examining) hospitals involving patients and healthcare professionals in order to improve the quality of care on Dutch hospital wards.<br><br>The research question guiding this article is: Why are hospitals interested in co-production activities and what are the experiences with co-production in practice? | |
| **Checklist completed by:** | DJC | |
| **Theoretical approach:** Hospitals participating described as utilising co-production methods but the term co-design is also used. Four of five project teams indicated they would use EBCD methods but for various reasons decided to amend or remove some of the standard 6 stages in EBCD. Therefore, the approach is based on but not directly comparable with other EBCD projects. For example, patients were not always involved in the improvement and implementation process. | | |
| **1. Is a qualitative approach appropriate?**<br><br>For example:<br><br>• Does the research question seek to understand processes or structures, or illuminate subjective experiences or meanings?<br><br>• Could a quantitative approach better have addressed the research question? | Appropriate ✓<br><br>Inappropriate<br><br>Not sure | Comments:<br><br>Describes a mixed methods qualitative study designed to understand why and how the 5 hospital project teams were involving patients in co-production projects designed to lead to service quality improvement. Use of observations, interviews (about process and outcomes in the projects) and documentary analysis provided for data triangulation. |
| **2. Is the study clear in what it seeks to do?**<br><br>For example:<br><br>• Is the purpose of the study discussed – aims/objectives/research question/s? | Clear ✓<br><br>Unclear<br><br>Mixed | Comments:<br><br>The paper reviews some of the co-production literature both in the wider public services arena and also in the more limited health services context.<br><br>There is an overview of the aim of the study and a single research |

| | | |
|---|---|---|
| • Is there adequate/appropriate reference to the literature?<br><br>• Are underpinning values/assumptions/theory discussed? | | question: *Why are hospitals interested in co-production activities and what are the experiences with co-production in practice?*<br><br>The research is a mixed methods approach to the above rather than an evaluation per se. The researcher's own assumptions and values are not discussed although the values and descriptive theory underpinning co-production is considered. |
| **Study design** | | |
| **3. How defensible/rigorous is the research design/methodology?**<br><br>For example:<br><br>• Is the design appropriate to the research question?<br><br>• Is a rationale given for using a qualitative approach?<br><br>• Are there clear accounts of the rationale/justification for the sampling, data collection and data analysis techniques used?<br><br>• Is the selection of cases/sampling strategy theoretically justified? | Defensible √<br><br>Indefensible<br><br>Not sure | Comments: |
| **Data collection** | | |
| **4. How well was the data collection carried out?**<br><br>For example:<br><br>• Are the data collection methods clearly | Appropriately √<br><br>Inappropriately | Comments: |

| | | |
|---|---|---|
| described?<br>• Were the appropriate data collected to address the research question?<br>• Was the data collection and record keeping systematic? | Not sure/inadequately reported | |
| **Trustworthiness** | | |
| **5. Is the role of the researcher clearly described?**<br>For example:<br>• Has the relationship between the researcher and the participants been adequately considered?<br>• Does the paper describe how the research was explained and presented to the participants? | Clearly described<br>Unclear √<br>Not described | Comments: |
| **6. Is the context clearly described?**<br>For example:<br>• Are the characteristics of the participants and settings clearly defined?<br>• Were observations made in a sufficient variety of circumstances<br>• Was context bias considered | Clear √<br>Unclear<br>Not sure | Comments: |
| **7. Were the methods reliable?** | Reliable √ | Comments: |

| | | |
|---|---|---|
| For example:<br>• Was data collected by more than 1 method?<br>• Is there justification for triangulation, or for not triangulating?<br>• Do the methods investigate what they claim to? | Unreliable<br>Not sure | |
| **Analysis** | | |
| **8. Is the data analysis sufficiently rigorous?**<br>For example:<br>• Is the procedure explicit – i.e. is it clear how the data was analysed to arrive at the results?<br>• How systematic is the analysis, is the procedure reliable/dependable?<br>• Is it clear how the themes and concepts were derived from the data? | Rigorous ✓<br>Not rigorous<br>Not sure/not reported | Comments: |
| **9. Is the data 'rich'?**<br>For example:<br>• How well are the contexts of the data described?<br>• Has the diversity of perspective and content been explored?<br>• How well has the detail and depth been demonstrated? | Rich ✓<br>Poor<br>Not sure/not reported | Comments: |

| | | |
|---|---|---|
| • Are responses compared and contrasted across groups/sites? | | |
| **10. Is the analysis reliable?**<br><br>For example:<br><br>• Did more than 1 researcher theme and code transcripts/data?<br><br>• If so, how were differences resolved?<br><br>• Did participants feed back on the transcripts/data if possible and relevant?<br><br>• Were negative/discrepant results addressed or ignored? | Reliable ✓<br><br>Unreliable<br><br>Not sure/not reported | Comments: |
| **11. Are the findings convincing?**<br><br>For example:<br><br>• Are the findings clearly presented?<br><br>• Are the findings internally coherent?<br><br>• Are extracts from the original data included?<br><br>• Are the data appropriately referenced?<br><br>• Is the reporting clear and coherent? | Convincing ✓<br><br>Not convincing<br><br>Not sure | Comments: |
| **12. Are the findings relevant to the aims of the study?** | Relevant √<br><br>Irrelevant | Comments: |

| | | |
|---|---|---|
| | Partially relevant | |
| **13. Conclusions**<br><br>For example:<br><br>• How clear are the links between data, interpretation and conclusions?<br><br>• Are the conclusions plausible and coherent?<br><br>• Have alternative explanations been explored and discounted?<br><br>• Does this enhance understanding of the research topic?<br><br>• Are the implications of the research clearly defined?<br><br>**Is there adequate discussion of any limitations encountered?** | Adequate √<br><br>Inadequate<br><br>Not sure | Comments: |
| **Ethics** | | |
| **14. How clear and coherent is the reporting of ethics?**<br><br>For example:<br><br>• Have ethical issues been taken into consideration?<br><br>• Are they adequately discussed e.g. do they address consent and anonymity?<br><br>• Have the consequences of the research been | Appropriate<br><br>Inappropriate<br><br>Not sure/not reported √ | Comments: There are no comments made about ethical review and approval. There is no discussion of consent or other ethical issues which may have arisen in relation to observations or interviews. Permission for recording of interviews and some observations is referred to briefly. |

| | | |
|---|---|---|
| considered i.e. raising expectations, changing behaviour?<br>• Was the study approved by an ethics committee? | | |
| **Overall assessment** | | |
| **As far as can be ascertained from the paper, how well was the study conducted? (see guidance notes)** | ++ √<br>+<br>− | Comments: |
