# Supplementary material for: What outcomes are associated with developing and implementing co-produced interventions in acute healthcare settings? A rapid evidence synthesis
Source: BMJ Open. 2017 Jul 11;7(7):e014650. doi: 10.1136/bmjopen-2016-014650 (PMC5734495; doi:10.1136/bmjopen-2016-014650)
Supplement: Supplementary material 2 [file bmjopen-2016-014650supp002.pdf]

Supplementary file 2 Empirical studies included in review

Publication/ country	Form of co-production	Setting	Methods	Reported Outcomes	Barriers and facilitators	Health economic evaluation	Quality rating
Bowen, S. et al, 2013 How was it for you? Experiences of participatory design in the UK health service. CoDesign, 9:4, 230-246<br><br>UK	Experience Based Design (EBD)	Medical outpatient service for older people. In a Teaching Hospital NHS Trust	Face to face (2) or telephone (9), semi-structured interviews (n= 11)	*Reported EBD Outcomes:*<br>• New template for patient appointment letters<br>• Design proposals for new way-finding materials (signage and maps)<br>• Proposal for layout of roads surrounding the outpatient building<br>• Video of patient stories distributed to all staff in the Trust to highlight older people's experiences,<br>• Forum theatre training event to improve outpatient staff awareness of customer care.<br>*Interview Findings*:<br>• Doing EBD (4 subthemes).<br>• Reflecting on EBD & Better Outpatient Services for Older People' (5 subthemes).	*Barriers:*<br>• Perception that participants were not doing the designing<br>• Perception that too many people were involved<br>• Ageing physical infrastructure Bureaucratic nature of trust procedures<br>• Low expectations of extent of change<br>• Availability of financial resources<br>• Staff workload<br>*Facilitators*:<br>• Sharing stories to gain perspectives of others<br>• Emotional mapping to highlight touchpoints (experiences and areas for improvement)<br>• Involvement in co-designing, being listened to by hospital staff	Not evident or reported.	++
Boyd. H. 2011 et al, Improving healthcare through the use of co-design. The New Zealand Medical Journal, 125,76–87	Experience Based Design (EBD) (Adapted)	Breast Services (inpatient & outpatient) in two hospitals in one District Health Board.	Patient journey mapping, experience surveys and co-design workshops.	• Patient journey map for staff review<br>• Patient Information folder to help navigate the service<br>• 7 new information leaflets,<br>• Patient held record to track appointments	*Barriers:*<br>• Staff workloads; EBD in addition to usual roles<br>• Financial restraints for hospitals and services<br>• Evaluation and planning for sustainability not factored into the	Not evident or reported.	+

Publication/ country	Form of co-production	Setting	Methods	Reported Outcomes	Barriers and facilitators	Health economic evaluation	Quality rating
New Zealand				• Patient focused visual patient journey guide<br>• Poster providing (better) communication guidance for staff and patients<br>• New design mammography gown,<br>• Co-design toolkit and website for other NZ services	EBD project<br>*Facilitators:*<br>• Engaging with patients at the outset of the co-design process before decisions are made<br>• Adopting a flexible approach to enable patients still receiving treatment to participate over time<br>• Working to get key staff buy in (clinical, managerial, administrative) & continued engagement<br>• Find & use ways to challenges participants' thinking, to work outside of their 'comfort zone'		
Golden.B.R. et al, 2011 Improving the patient experience through design. Healthcare Quarterly. 14(3)32-41<br><br>Canada	Rotman Creative Design Process<br>(co-design approach)	Cancer research and treatment centre.<br>Systemic therapy:<br>chemotherapy and transfusion services.	Ethnographic observations. Photojournalling & follow up interviews.<br>Staff discussion group: patient vignettes/ story-boarding ideal patient journey<br>Patient discussion group & storyboarding ideal patient journey.	• Prototypes and a jointly designed ideal patent journey	Not specifically reported.<br><br>Indicates staff workload and patient treatment scheduling pressures precluded bringing the two groups together to co-design service improvements.	Not evident or reported.<br><br>Project funded by charitable & corporate donations to the hospital & the University.	+
Larkin, M et al, 2015 On the Brink of Genuinely Collaborative Care: Experience-	Experience-based Co-Design<br>(EBCD)	Early Intervention Services and Inpatient units (n =7)	In depth interviews with service users (n=6), parents (n=6), inpatient staff (n=9).	Six priorities for service improvement identified from interviews and feedback group work:	*Facilitators:*<br>• Support from senior NHS staff<br>• Researchers leading the processes	Not evident or reported	+

Publication/ country	Form of co-production	Setting	Methods	Reported Outcomes	Barriers and facilitators	Health economic evaluation	Quality rating
Based Co-Design in Mental Health.Qualitative Health Research2015, Vol. 25(11) 1463–1476<br><br>UK	(adapted)	at two hospitals in the Midlands.<br><br>Mental health services for young people and families.	Feedback groups (20) involving 150 stakeholders.<br><br>Co-design event (50 participants).	• pathways in and out of hospital<br>• providing staff with a rewarding and well-supported role<br>• communicating with families and service-users<br>• recovery-focused practice<br>• creating a positive environment for everyone<br>• recognizing and sharing good practice across professions and services<br>• Co-design groups at the co-design event produced 13 action plans to address the 6 priorities	• Involvement of people internal and external to the service in the EBCD team aided project management<br>• Sensitivity to the needs and experiences of services uses in this setting in identifying touchpoints and developing trigger films to share with staff<br><br>*Barriers:*<br>• Challenging for some staff to hear strong service-users and carer views on services<br>• Lack of time and high level organisational support for Steering Group to complete implementation plans<br>• Steering group lacking the power to bring about change in organisations<br>• Organizational restructuring resulting in some study collaborators and "champions" leaving or changing role,<br>• Improvements requiring input from other departments or requiring strategic, budgetary, or staffing commitment<br>• Lack of organizational capacity to respond flexibly or directly to "bottom-up" service development		
Locock, L et al, (2014) Using a	Accelerated Experience-	In intensive care and	Process evaluation using ethnographic	*Re: AEBCD*	*Anticipated Barriers:*	In AEBCD the cost of	++

Publication/ country	Form of co-production	Setting	Methods	Reported Outcomes	Barriers and facilitators	Health economic evaluation	Quality rating
national archive of patient experience narratives to promote local patient-centered quality improvement: an ethnographic process evaluation of 'accelerated' experience-based co-design. Journal of Health Services Research &Policy2014, Vol. 19(4) 200–207<br><br>UK	based Co-Design (AEBCD)	lung cancer services in two English NHS hospitals	methods:<br>• observations of AEBCD processes and events (155 hours)<br>• interviews (n=30)<br>• repeated group interviews with patient participants<br>• reflective diaries (n=22)<br>• service improvement logs (n=4)<br>• evaluation questionnaires (post AEBCD events)	Trigger films developed through secondary analysis if existing films from the Oxford University collections of patient experience interviews were acceptable to patients and staff and were used successfully in subsequent co-design work.<br><br>*Re: Service improvements*<br>48 co-design activities across the four patient pathways. Examples reported were:<br>• Improved cross-site information booklet for patients transferring to another hospital for surgery<br>• A redesigned discharge summary with input from all professions<br>• More comfortable V-shaped pillows for postoperative patients<br>• Sourcing clocks to aid patient orientation in ICU<br>• Changed process for porters to remove waste avoiding ICU rest times<br>• New private room identified for receiving support after diagnosis<br>• Introducing mini 'Schwartz rounds' on ICU	Films developed from a national archive would be considered inappropriate or an inaccurate reflection of care in a specific hospital.<br><br>Barrier not evident: 85% of participants (patients and relatives) reported films to be good or excellent.<br><br>No other barriers reported<br><br>*Facilitators:*<br>• Regular face-to-face meetings with patients in co-design groups reminded staff of who change was for and why it mattered<br>• Co-design groups facilitated active and empowered partnerships between patient and staff	developing a trigger film was £8289, compared to £30,485 for EBCD.<br><br>Co-design<br>phase costs, were £20,276<br>(over half of this was facilitator salary).	

Publication/ country	Form of co-production	Setting	Methods	Reported Outcomes	Barriers and facilitators	Health economic evaluation	Quality rating
Piper, D. et al, 2012 Utilizing experience-based co-design to improve the experience of patients accessing emergency departments in New South Wales public hospitals: an evaluation study. Health Services Management Research2012;25: 162 – 172<br><br>Australia	Experience-based Co-Design (EBCD)	Emergency Department (EDs) & related services in two (programme) stages in seven public hospitals in New South Wales, Australia.	Review of EBCD process and evaluation reports provided by participating sites.<br><br>Interviews of patients, clinicians and managers, and also of high-level project managers including executive<br><br>sponsors, clinical leads, project managers across sites (n=117)	EBCD processes provided opportunities to work together meaningfully and enabled patients, carers and staff to develop a deeper understanding of each other's experience of care processes, and this strengthened relationships among those involved in EBCD.<br><br>Examples of co-designed strategies implemented and sustained at the programme sites included:<br><br>• Recruitment of a full-time cleaner<br>• Installation of a new air conditioning unit in the waiting room<br>• Installation of a blanket warming cupboard<br>• Installation of food vending machines in the waiting area<br>• Access to free refreshments<br>• Installation of pay phones in the waiting area<br>• Patient access to 'sick-bay' bags in the waiting area<br>• Increased security presence in the emergency room.<br><br>22 examples are provided in the	*Barriers:*<br>• Staff viewed EBCD as a burden additional to already busy schedule<br>• Limits in implementation officers' & clinical leads' EBCD roles, reportedly reduced impact of programmes & long-term change<br>• Consumer recruitment & retention was difficult due to the transitory nature of the care episode<br>*Facilitators:*<br>• Develop a consumer engagement strategy, using on-going communication including face-to-face bi-monthly re-connect meetings, telephone & email contact/updates<br>• Integrating the EBCD process into everyday work practices rather than presenting EBCD as a separate task<br>• Adequate resources & reporting opportunities to executive meetings<br>• Clear accountability for implementation, allow adequate time to embed solutions into daily practice and cultures of EDs<br>• Appointing a liaison person to: ensure regular participant contact; manage governance of project & processes; communicate & contextualize aims & timeframes of EBCD to staff; develop a communication strategy to inform all stakeholders of the status & impact	Not evident or reported.	+

Publication/ country	Form of co-production	Setting	Methods	Reported Outcomes	Barriers and facilitators	Health economic evaluation	Quality rating
				publication.<br><br>Implementation strategies also identified.	of solutions on an on-going basis		
Thomson,A., Rivas,C., and Giovannoni. G. Multiple sclerosis outpatient future groups: improving the quality of participant interaction and ideation tools within service improvement activities. BMC Health Services Research (2015) 15:105. DOI 10.1186/s12913-015-0773-8<br><br>UK	Participatory approach drawing on techniques from the speculative design field.	An outpatient clinic for patients with multiple sclerosis in London, UK.	3 staged process using focus groups (future groups). In stages 1 and 3 patient participants and staff participants worked separately. In stage 2 the two groups were combined.<br><br>Process completed in 3 months<br><br>Speculative design techniques included: directed patient and staff journey mapping; combining patient and staff maps of an ideal (patient) journey (using analogies and props-a set of travel related documents); developing service improvements (represented on researcher developed separate patient and	• Volunteer in the outpatient clinic, linked to a Patient Advisory Group meeting 3 monthly<br>• A guide to the MS clinic for new outpatients<br>• An MS clinic dictionary (covering medical terms related to MS and frequently used in the clinic)<br>• A walking map with distances to the clinic (from the underground or disabled parking areas or local coffee shops)<br>• Magazine replenishment (as a clinic resource)<br>• Informal staff training programme (developments in MS research and care)	Facilitators:<br>• Adding (speculative) design led techniques to a participatory approach broadly similar to the 6 stage EBCD approach<br>• Adapting the design led props for the specific healthcare context<br>• Involving the design researcher directly in the (future) focus groups<br>• Including senior staff/managers in the stage 3 (future) focus groups to facilitate understanding of proposed service developments and their implementation.<br>Barriers:<br>• Adapting design led methods for health services improvements projects may reduce their creative contribution if used in a procedural manner<br>• Participant engagement with design led metaphor and analogy driven techniques need to be considered carefully and reflect local and disease specific contexts	Not evident or reported.	+

Publication/ country	Form of co-production	Setting	Methods	Reported Outcomes	Barriers and facilitators	Health economic evaluation	Quality rating
			staff conference style posters (prototyping).				
Tollyfield, R. 2014 Facilitating an accelerated experience-based co-design project. British Journal of Nursing, 2014, l23, (3):136-141<br><br>UK	Accelerated Experience-based Co-Design (AEBCD)	Specialist cardiothoracic intensive therapy unit (ITU) in London.<br><br>(One of the four units involved in the Locock et al 2014 study)	Reflection on the role of facilitator in an AEBCD project in one of two ITUs participating in the project.	Reports on the experience of and learning from facilitation of the AEBCD study conducted by Locock et al (2014).<br><br>• Promoting staff engagement a key aspect of the project- via posters, emails, one-to-one discussions and a ITU weekly newsletter<br>• Developing solutions to low patient recruitment levels<br>• Providing facilitation of the main project events and co-design meetings<br>• Prompting and supporting implementation of planned change	*Facilitators:*<br>• Project supported and endorsed by senior managers and clinicians- Director of Nursing, critical care service manager, ITU consultant physician<br>• Formal training in EBCD methods and on-going email and telephone support from staff experienced in using EBCD<br>• Access to and use of the online Kings Fund EBCD toolkit<br>*Barriers:*<br>• facilitator role time consuming and impacted significantly on time for clinical work (externally funded role for experienced ITU clinician at this site)<br>• learning about the AEBCD process whilst undertaking project organisational tasks and activities against a defined timeline	Not reported.<br><br>See Locock et al (above) for costs of facilitator salaries.	+
Tsianakas,V et al, 2012 Implementing patient-centred cancer care: using	Experience-based Co-Design (EBCD)	A large, inner-city cancer centre in	Ethnographic observation (219 hours)<br>Filmed narrative	*16 breast cancer working group outcomes including*:	*Barriers:*<br><br>Not specifically identified in the	Not evident or reported	++

Publication/ country	Form of co-production	Setting	Methods	Reported Outcomes	Barriers and facilitators	Health economic evaluation	Quality rating
experience-based co-design to improve patient experience in breast and lung cancer services. Supportive Care in Cancer (2012) 20:2639–2647<br><br>UK		England.<br><br>(Breast and lung cancer patients)	patient interviews (n=36)<br><br>Staff interviews (n= 63)<br><br>Post project interviews with<br><br>Staff (n=4)<br><br>and patients (n=5)	• Administrative staff receive customer-care training and are shown patients' DVD<br>• Healthcare assistants' interpersonal skills assessed prior to recruitment<br>• Managers and administrative staff use values-based performance tool which can improve patients' experience<br>• Changes to structure of clinics to reduce waiting times<br>• Patients receiving same chemotherapy treatment given option to receive information in a group<br><br>*11 lung cancer working group outcomes including:*<br><br>• Establishment of second breaking-bad-news room<br>• Guidance on diagnosis procedures included in junior doctors' induction<br>• Promotion of information and support centres at different sites (advertising at hospital entrance)<br>• Patient DVD 'welcome to cancer services' for newly diagnosed and	publication.<br><br>• As EBCD does not explicitly seek to recruit a representative sample of patients but those who are motivated to 'tell their story' and participate in the co-design work. This may lead to the experiences of 'harder to reach' patient groups remaining unheard.<br><br>*Facilitators:*<br><br>• Genuine and direct patient and carer involvement (relative to other service improvement projects in which patients and staff had participated).<br>• Patient participants taking direct responsibility for the work and its outcomes<br>• The EBCD change process itself together with the direct and active participation of staff and patients produced implementation and action, and contributed to spread and sustainability		

Publication/ country	Form of co-production	Setting	Methods	Reported Outcomes	Barriers and facilitators	Health economic evaluation	Quality rating
				referred patients			
Tsianakas,V., Robert ,G., Richardson,A., Verity,R., Oakley,C., Murrells,T., Flynn,M., Ream,E. Enhancing the experience of carers in the chemotherapy outpatient setting: an exploratory randomised controlled trial to test impact, acceptability and feasibility of a complex intervention co-designed by carers and staff . Support Care Cancer. 2015. DOI 10.1007/s00520-015-2677-x<br><br>UK	The study reports an RCT to test the impact and feasibility of a complex intervention (Take Care). The intervention was developed previously in the same hospital using a co-design approach – Experience-Based Co-Design.	Chemo-therapy day unit (breast, lung or colorectal cancer) in a London teaching hospital.	A two phase mixed methods design including i) an exploratory RCT (impact-measured through before and after postal questionnaire completion), ii) focus groups with carers and healthcare professionals (feasibility and acceptability)	• Carers in the intervention group had better understanding of the symptoms and side effects compared with the control group.<br>• Carers in the intervention group had more confidence in coping than the control group.<br>• There were no differences between the 2 groups for emotional well-being<br>• Health care professionals and carers confirmed the feasibility and acceptability of the EBCD derived Take Care intervention<br>• Patients and HCPs reported the intervention was educational, enhanced knowledge of symptoms and side effects, and increased confidence of carers.	Not reported in terms of EBCD<br><br>Reported limitations of the study included:<br>• Small sample size, no power calculation<br>• Exploratory trial in a single centre limits generalisability<br>• Self-report questionnaire responses may be subject to recall bias	Not evident or reported	+
Vennik et al, Co-production in healthcare:	Experience-based Co-Design (EBCD)	One haematolog y and four	Semi-structured interviews (n=27)	Improvement areas identified by patients were similar across hospitals. Apart from some specific	Barriers:<br>• Individual level- some physicians	Not evident or reported	++

Publication/ country	Form of co-production	Setting	Methods	Reported Outcomes	Barriers and facilitators	Health economic evaluation	Quality rating
rhetoric and practice. International Review of Administrative Sciences 2016, Vol. 82(1) 150–168 Netherlands	in 4 centres (Adapted) Four project teams planned to use EBCD methodology; all adapted the method. Two used focus groups to gather patient experiences instead of filmed interviews. Two did not interview professionals. One did not use co-design workgroups concluding that it would require too much from participants; staff were not immediately convinced of the added value of this	oncology departments in 5 Dutch hospitals	Observations (70 hours) Documentary analysis	experiences with medical treatments, they mostly concerned aspects of quality of care including: • Decoration of waiting rooms, • Waiting times, -Communication, and coordination with general practitioners. • Psychosocial care is now proactively offered during consultations at one hospital, • A dental care in oncology leaflet was introduced at another hospital Improvement issues identified were related to individual professions, hospital wards, the hospital organization and other healthcare providers.	afraid of being criticised by patients, • Organisational barriers-pressure of work and other project commitments, changes in staffing levels, service re-organisations during the 12 months projects • EBCD largely seen as 'additional work' • Local policies and national trends can impact on engagement with co-production, no staff time freed for co-production/co-design, staff expected to deliver treatment and care and then engage in service improvement in their own time. • Fee-for-service systems do not directly reward projects like EBCD/co-production Facilitators: • Motivated people who are willing to work on EBCD/co-production in their own time • Selecting the 'right patients', inviting patients who have a critical point of view, are mentally and physically well enough condition to participate, and able to tell their story. • It is not so much what patients say that is important; it is the process and the way they are given the opportunity to have their say (in EBCD/co-production) The active role of patients creates a sense of urgency		

Publication/ country	Form of co-production	Setting	Methods	Reported Outcomes	Barriers and facilitators	Health economic evaluation	Quality rating
	phase				to act on the improvement issues raised<br>• The process of co-production/EBCD stimulated staffs' thinking about how to realize quality improvements<br>• Keeping projects 'small', not attempting to improve everything at once and focusing<br>• Patients' input can gain legitimacy for improvement plans and facilitate implementation		
