# Supplementary material for: What outcomes are associated with developing and implementing co-produced interventions in acute healthcare settings? A rapid evidence synthesis
Source: BMJ Open. 2017 Jul 11;7(7):e014650. doi: 10.1136/bmjopen-2016-014650 (PMC5734495; doi:10.1136/bmjopen-2016-014650)
Supplement: Author's manuscript [file bmjopen-2016-014650.draft_revisions.pdf]

BMJ Open

What outcomes are associated with developing and implementing co-produced interventions in acute healthcare settings? A rapid evidence synthesis.

Journal:	*BMJ Open*
Manuscript ID	bmjopen-2016-014650
Article Type:	Research
Date Submitted by the Author:	09-Oct-2016
Complete List of Authors:	Clarke, David; University of Leeds, Academic Unit of Elderly Care and Rehabilitation<br>Jones, Fiona; St Georges University of London,<br>Harris, Ruth; King\'s College London,<br>Robert, Glenn; King's College London,
**Primary Subject Heading**:	Health services research
Secondary Subject Heading:	Evidence based practice, Qualitative research
Keywords:	rapid evidence synthesis, co-production, acute healthcare

SCHOLARONE™
Manuscripts

For peer review only

What outcomes are associated with developing and implementing co-produced interventions in acute healthcare settings? A rapid evidence synthesis.

Corresponding author:
Dr David Clarke
Lecturer in Stroke Care,
Academic Unit of Elderly Care and Rehabilitation
Leeds Institute of Health Sciences
Temple Bank House
Bradford Royal Infirmary
Bradford
BD9 6RJ
Tel: 01274 383441
Mobile: 07503727990
Email: d.j.clarke@leeds.ac.uk

Professor Fiona Jones,
Professor of Rehabilitation Research,
Faculty of Health Social Care and Education
St George's University of London
2nd Floor Grosvenor Wing
Cranmer Terrace
London SW17 0RE
f.jones@sgul.kingston.ac.uk

Professor Ruth Harris,
Professor of Healthcare for Older Adults,
King's College London
Florence Nightingale Faculty of Nursing and Midwifery
Room 2.24, James Clerk Maxwell Building
57 Waterloo Road
SE1 8WA
ruth.harris@kcl.ac.uk
Tel: 020 7848 3708

Professor Glenn Robert,
Professor of Healthcare Quality & Innovation,
Kings College London
Florence Nightingale Faculty of Nursing and Midwifery
James Clerk Maxwell Building
Waterloo
United Kingdom
Telephone: +44 (0)20 7848 3063
E-mail: glenn.robert@kcl.ac.uk

on behalf of the Collaborative Rehabilitation in Acute Stroke (CREATE) team

Word count: 4466 excluding title page, abstract, references, figures and tables.

For peer review only

What outcomes are associated with developing and implementing co-produced interventions in acute healthcare settings? A rapid evidence synthesis.

Abstract

Background: interest in the use of co-production as an intervention for improving healthcare quality is increasing. Co-production goes beyond simply consulting patients about their experiences; it enables both patients and staff to work in partnership to improve the quality of services. Little is known regarding the impact of co-production on patient, staff or organizational outcomes in acute healthcare settings.

Aims: to identify and appraise reported outcomes of co-production as an intervention to improve quality of services in acute healthcare settings.

Design: rapid evidence synthesis.

Data Sources: Medline, Cinahl, Web of Science, Embase, HMIC, Cochrane Database of Systematic Reviews, SCIE, Proquest Dissertation and Theses, EThOS, OpenGrey (2005-16); hand searching of journals CoDesign; Design Journal; Design Issues.

Study selection: studies reporting patient, staff or organisational outcomes associated with using co-co-production approaches in an acute healthcare setting

Findings: 710 titles and abstracts were screened, 24 papers underwent full text review; 11 papers were included in the evidence synthesis. One study was a feasibility randomised controlled trial, three were process evaluations and seven used qualitative methods. Reported outcomes related to: (a) the value of patient and staff involvement in co-production processes; (b) the generation of ideas for changes to processes, practices and clinical environments; and (c) tangible service change and impact on patient experiences. Lessons for implementing co-production in acute healthcare settings were identified. Only one study included cost analysis; none reported an economic evaluation. No studies assessed the sustainability of changes made due to co-production.

Conclusions: despite increasing interest in and advocacy for co-production there is a lack of rigorous evaluation in acute healthcare settings. Future studies should focus on both the clinical and cost-effectiveness of co-production relative to other forms of quality improvement interventions as well as potentially broader impacts on the values and behaviours of participants.

Key words: Rapid evidence synthesis, co-production, acute healthcare

For peer review only

Strengths and limitations of this study

This study is the first to systematically review outcomes associated with developing and implementing co-produced interventions in acute healthcare settings.

We have identified a lack of rigorous evaluation of effectiveness and cost effectiveness of co-produced interventions in the acute healthcare sector at both the service and system level.

The study did not access and review the grey literature and therefore may not have identified reports of co-production projects published only in that literature.

We included only studies where full-text reports published in English within the last 10 years were available, it is possible that co-production studies published in other languages have been overlooked by our review. .

For peer review only

What outcomes are associated with developing and implementing co-produced interventions in acute healthcare settings: a rapid evidence synthesis.

BACKGROUND

There is renewed interest in and advocacy for adoption of 'co-production' as a means of co-creating value across the public sector. However, as the term increasingly enters mainstream management (and healthcare quality improvement) discourses there is a sense that it may be being misused or misunderstood, thereby running the risk of becoming denuded of meaning or losing any association with its radical roots.

Co-production was first conceptualised in the United States in the 1970s, originally as a response to the lack of recognition of service users in service delivery [1]. The creation of time banks, a system reliant on the participation of volunteers who are also service users [2,3] showed how collaborative interventions that involve people with long-term psychosocial needs could contribute to improved community links [4]. Since this early work a variety of terms have become evident in the growing literature on co-production, and a range of related approaches are being enacted in different ways and at various levels throughout public and health sector services [5-11]. For the purposes of this paper we follow Osborne et al recent definition of co-production as 'the voluntary or involuntary involvement of public service users in any of the design, management, delivery and/or evaluation of public services' [8].

In the healthcare context co-production is promoted as harnessing the knowledge of patients, carers and staff to make changes about which they care most [12]. Berwick has recently argued for a greater focus on co-production as part of a proposed 'third era' of medicine [13]. Potential benefits might include from a patient perspective improved satisfaction with the experience of care, reduction in complications associated with treatment and improved health outcomes. From a staff and organizational perspective, the case for co-production may include improvements in staff wellbeing, increased recognition of the need for a better understanding of patient perspectives and changes in attitudes towards working with patients as partners in quality improvement.

In a recent commentary Batalden et al, whilst arguing that where healthcare activities are co-produced, services, providers and service users become far more effective agents of change, note

For peer review only - http://bmjopen.bmj.com/site/about/guidelines.xhtml

that current systems can both support and constrain partnerships between patients and professionals (and that historically this kind of partnership has been unequal) [12]. The evidence base in terms of robust evaluation of the impact of co-production on patient outcomes appeared limited; Batalden et al argue this raises a series of questions for proponents of co-production including the need to determine '[m]easures that accurately reflect co-produced service and its results [and] measures which will help us understand co-productive processes' [12: 7].

Osborne et al, starting from the premise that co-production is (or has become over time) a 'woolly word', helpfully critiqued its conceptualization within the policy and practice literature, linked it directly to the co-creation of value in public service delivery and proposed a framework for differentiating 'the cluster of related concepts that are contained within the term 'co-production' [8]. This framework comprises 'four ideal types of value that are co-created in public service delivery by the iterative interactions of service users and service professionals with public service delivery systems'; namely co-production, co-design, co-construction and co-innovation. Voorberg et al conducted a systematic review of the literature (1987-2013) relating to co-creation/co-production with citizens in public innovation across all sectors concluding that most studies focus on the identification of influential factors, while hardly any attention was paid to outcomes [14]. Greenhalgh et al report a narrative review of different models of co-creation relevant to community-based health services; they identify key success principles but note that 'impact is by no means guaranteed' [5].

For the purposes of this rapid evidence synthesis (RES) we identify co-production and co-design as specific approaches to co-creating value at the service (or clinical microsystem) level within an acute healthcare organization. Co-design is an approach to participatory design (traditionally of a new product) that seeks to actively involve all stakeholders (e.g. staff, patients, citizens) in a process to help ensure the result meets their needs and is usable. Such approaches are increasingly being used often situated in the emerging Service Design field [15,16] as part of co-production projects. Specific forms of co-design such as Experience Based Design (EBD) [17,18] and Experience-based Co-design (EBCD) [19] have been developed and applied in the healthcare sector. We also follow Osborne et al conceptualisation of their final two 'types of value' (co-construction and co-innovation) as being concerned with service systems (rather than individual services) [8]; we return to this important distinction at the end of this paper.

We undertook this RES to inform an ongoing study (Collaborative Rehabilitation in Acute Stroke-CREATE) which is bringing together staff, former patients and carers to review and jointly co-produce the way in which therapeutic rehabilitation-related activity is provided in stroke units in the early days and weeks after stroke [20]. In the absence of existing published evidence, this RES aims to identify and evaluate reported outcomes of co-produced interventions designed to achieve patient focused quality improvements in acute healthcare settings. To inform the empirical study our secondary objectives were to identify in such settings:

- those elements of co-production processes that lead to patient focused quality improvements;
- any evidence of cost-benefit analysis, cost reduction or other economic evaluation associated with co-produced interventions;
- barriers and facilitators reported to influence the implementation and use of co-production methods and implementation of co-produced interventions; and
- issues which impact sustainability of quality improvements following the use of co-production.

REVIEW METHODS

For peer review only - http://bmjopen.bmj.com/site/about/guidelines.xhtml

Whilst there are no agreed international guidelines for the design and conduct of RES [21] there is overall agreement the process involves providing an overview of existing research on a defined topic area, together with a synthesis of the evidence provided by these studies to address specific review questions. RES are typically completed in 2-6 six months; this does not normally allow for all stages of traditional effectiveness reviews [21-23]. However, RES are increasingly valued by policy makers who require rapid answers to specific questions to inform policy revision or service development [21,24].

Limitations of RES include the potential for introduction of bias. Sources of bias can include limiting the scope of the search to published literature or specific databases, to a specific focus within a larger topic, or to a reduced timespan of publication. In RES of effectiveness, meta-analyses may be omitted. It is important therefore that RES are conducted systematically; methods used and limitations introduced by these should be reported transparently [21-23].

This RES was conducted between January and June 2016. We used a systematic approach and included full-text evidence published in English within the last 10 years. Search terms and databases searched were agreed by researchers and Information Specialists at the University of Leeds. Search terms selected used focus on co-production in acute healthcare settings (see supplementary file 1).

Box 1: Database and hand searches

Medline, Cinahl, Web of Science, Embase, HMIC, Cochrane Database of Systematic Reviews, SCIE, Proquest Dissertation and Theses, EThOS and OpenGrey.<br><br>The following journals were hand searched:<br>CoDesign; The Design Journal; and Design Issues.

Database searches were conducted for the period January 1st 2005 to 31st January 2016. Given existing reviews [5, 14] and the CREATE study focus, we reviewed post 2005 evidence and only that reporting on studies in acute healthcare settings. We completed citation tracking of 5 seminal papers; five experts in co-production were requested to nominate 3-5 seminal papers relevant to our review. Inclusion criteria are shown in table 1:

Table 1: inclusion criteria

• Reports research using a co-creation or co-production, or co-design or Experience-Based Co-Design approach in an acute healthcare setting
• Reports patient or staff or organisational outcomes resulting from research using a co-creation, co-production, co-design, Experience-Based Co-Design approach in an acute healthcare setting<br><br>Outcomes of interest include:<br>- any measure of the outcome of co-produced interventions on patient focused quality improvements in acute healthcare settings as reported by patients or families or caregivers, or health service providers<br>- including patient reported outcome measures and patient or staff reported experience measures

For peer review only - http://bmjopen.bmj.com/site/about/guidelines.xhtml

- using qualitative or quantitative data
• Acute healthcare settings include:<br>- emergency departments/accident and emergency departments<br>- adult inpatient facilities including: acute medical or surgical admission units (often termed MAUs or SAUs), acute medical or surgical units, acute trauma units, acute neurological units, intensive or critical care units, acute care of the elderly or geriatric units, medical oncology or cancer services<br>- adult outpatient facilities including medical, surgical, trauma, neurology, care of the elderly or geriatrics, medical oncology or cancer services

Two reviewers independently read all titles and abstracts. Discrepancies in retain or reject decisions were addressed by discussion between reviewers with recourse to a third where consensus could not be reached. Retained full text papers were independently read by three reviewers and retained or rejected based on the inclusion criteria.

Three reviewers completed data extraction. We used quality appraisal checklists developed for quantitative and qualitative studies [25] (Table 2). For each study two reviewers undertook data extraction and quality appraisal independently. Studies were not excluded on the basis of quality appraisal; this was used to inform the discussion of the evidence identified in the review. We adopted a mixed research synthesis approach [26] drawing on methods reported by Boger et al [27]. We used an integrated design which entailed grouping studies for synthesis not by methods (i.e., qualitative and quantitative) but rather by findings viewed as answering the same research questions, or addressing the same aspects of a target phenomenon.

RESULTS:

A total of 712 titles and abstracts were identified for screening from which 24 papers were identified for full text review. Following review, eleven publications were included in the final evidence synthesis (figure 1); full details of each study can be found in supplementary file 2.

Table 2: Quality Assessment study rating criteria [25]

++ All or most of the checklist criteria have been fulfilled, where they have not been fulfilled the conclusions are very unlikely to alter.
+ Some of the checklist criteria have been fulfilled, where they have not been fulfilled, or not adequately described, the conclusions are unlikely to alter.
– Few or no checklist criteria have been fulfilled and the conclusions are likely or very likely to alter.

Papers were published between 2008 and January 2016, with studies conducted in five countries (Canada, England, New Zealand, Australia, Netherlands). Settings included intensive care units (n=1), inpatient and outpatient oncology services, including breast, lung, colorectal and haematology services (n=5), mental health services (n=1), emergency departments (n=1), an outpatient clinic for people with multiple sclerosis (n=1), and older peoples outpatient services (n=1). A further study evaluated co-design projects in five Dutch hospitals in oncology departments (n=4) and a haematology department.

For peer review only - http://bmjopen.bmj.com/site/about/guidelines.xhtml

One study was a feasibility randomised controlled trial (RCT) of a co-designed intervention [28]; the remaining studies drew on qualitative methods although these were rarely described in detail. Data collection methods included self-report postal questionnaires, ethnographic observations of patient journeys through services and of staff working practices, semi-structured interviews with staff and patients (sometimes filmed), focus groups and emotional mapping exercises. Data analysis techniques were described in any detail in only six of the eleven publications [29-34]. Most papers focused on the processes used to understand and co-design within services rather than evaluating outcomes; the exception being the feasibility RCT [28].

Quality assessment ratings are shown in the final column of the table in supplementary file 2. Four studies were rated at ++ with the remaining seven at +. Ratings largely reflect the omission of detail on research methods, particularly in relation to stating research aims, questions, sampling decisions and discussion of data analysis and findings.

Figure 1: Adapted Prisma diagram insert here

Use of co-production methods

Methods reported included: a feasibility RCT of the impact of a co-designed intervention [28], participatory design [35], creative design [38], co-production [34], experienced-based design (EBD)[30,40], experienced-based co-design (EBCD) [31-34]and accelerated experienced-based co-design (AEBCD) [29,39]. EBD or variants of this approach was reported in eight of eleven included studies, with a further study testing an intervention previously designed using EBCD [37]. The use of a staged process for co-production was evident across all studies. Projects ran for 3 months to over one year.

There was also variation in levels of participant engagement. In an oncology service, whilst patients and a wide range of staff were included in the creative design project, they participated separately in the three stages of the study and did not come together to share and discuss their respective experiences [38]. In the Thomson et al study a designer led, three-stage process was used in which carers and staff came together only once [35].

Reported outcomes of co-produced interventions

Our primary aim was to identify and evaluate the reported outcomes of co-produced interventions designed to achieve patient-focused quality improvements in acute healthcare settings. Overall, patients reported positive experiences of participation, consistent with those associated with a wide range of co-production in the public and healthcare sector [14, 36]. Co-production or co-design projects were effective in generating a wide range of ideas and specific suggestions related to improving patients' experiences across the different settings. Reported service changes and quality improvements are summarised in table 3. However, clarity regarding implementation of improvements to services and/or evidence of resulting impact on patient outcomes was one of the main limitations of the reporting in the majority of the studies.

For peer review only - http://bmjopen.bmj.com/site/about/guidelines.xhtml

The feasibility RCT [28]evaluating the impact of a co-designed intervention to improve the knowledge, experience and emotional well-being of carers of people with breast, lung or colorectal cancer was the only study reporting use of a validated outcome measure, the GHQ-12 [28]. The other quantitative measures used in this study were adapted from existing measures (n=2, for example, supportive care needs) or developed by the study team (n=2, for example, experience of care and knowledge of chemotherapy). Carers who received the intervention reported significantly better understanding of symptoms and side effects and that their information needs were more often met compared with the control group [28]. Confidence in coping improved between baseline and follow-up for the intervention group and declined for the control (although differences were not statistically significant). Accepting the limitations of the single centre and small sample size, this study provided evidence for the effectiveness of the carer focused intervention developed in an earlier study by carers and staff using EBCD methods [37]. In the remaining 10 studies there were no reported instances of use of validated outcome measures, such as quality of life measures, nor any instances of validated patient reported experience (PREM) or outcome measures (PROM).

Four studies [30, 32, 34, 38], did not clearly differentiate between the findings from the co-production or co-design processes used and patient focused quality improvement outcomes. In these studies it was difficult to distinguish between ideas for improvement generated by co-design activities and tangible service improvements that had either been introduced or were planned. However, the reported 'outcomes' of the studies can be categorised in three main ways and are summarised in table 3 below:

1) patient and staff involvement in the co-production or co-design processes;

2) generating ideas and suggestions for changes to processes, practices and clinical environments impacting on patients' and/or carers' experiences of a service, (and often indirectly on staffs');

3) tangible change in services and impact on patient or carer experiences.

Elements of co-production that led to quality improvement

Tsianakas et al [33] identified genuine and direct patient and carer involvement (relative to other service improvement projects in which patients and staff had participated) and linked this to patient participants taking direct responsibility for the work and its outcomes as being success factors linked to the EBCD process itself. Similar claims were evident in other EBD and EBCD projects [29, 30,34, 40], where patients perceived there to be a partnership with staff in the co-production process[29,30] and where staff reported facilitated co-design groups directly contributed to active and empowered partnerships between staff and patients in which provided genuine opportunities for patients to 'have their say'[29,30,34]. There appeared to be an equally powerful impact on some staff participating in co-production in that the methods required them engage with and listen to patients in ways most had not experienced previously [31,33,34,40]. In these studies, co-production methods provided the means to ensure patients' experiences, concerns and ideas for change were captured, presented to and discussed with staff in services; active engagement of patients in this way gave both legitimacy and urgency to service improvement plans [34, particularly where managers were involved in or actively supported co-production projects [32,34,39,40]

However, despite clear evidence of the contribution of co-design activities in generating ideas for patient focused service improvements (see table 3), in some projects staff and patients were frustrated by the lack of progress from problem and solution identification to actual quality improvements [30-32, 34]. In one case, the funded project appeared to end before concrete action planning to effect change, based on the ideas generated, had occurred [38]. Researchers ascribed this to a combination of the time taken to undertake co-design activities and the often slow processes of change in complex health service organisations.

For peer review only - http://bmjopen.bmj.com/site/about/guidelines.xhtml

Economic evaluation

There were no cost-benefit analyses related to reported outcomes for any of the studies. Only one study reported a cost analysis of co-production methods by comparing the cost of an accelerated EBCD approach (AEBCD) to standard EBCD [29]. A significant reduction in costs was reported for AEBCD. The cost of developing patient experience (trigger) films fell from £30,485 to £8,289 and, as the film developed could be used in future projects, this was a one-off cost. This study was also the only one to quantify costs of the co-design stages which were £20,276; over half of this sum was for the salary of local facilitators.

Other authors alluded to the costs associated with participatory or co-design methods in terms of the largely time-related costs involved in freeing staff to take part in or run the projects, or costs associated with actual or planned service improvements. These include, for example, improved signage [30], provision of personal care equipment [31], information booklets/leaflets, DVDs [29,33,34,35,40], a website and web based toolkit [40]or costs associated with having blood tests conducted off site [39]. However, no actual or estimated costs were reported for these elements. Similarly, nine of the eleven publications reported on researcher led, designer led or researcher supported projects but in only two cases were actual research costs traceable [29, 38].

Barriers and facilitators to implementation

The eleven studies encompassed nine projects conducted in diverse acute healthcare settings, although few of the reported barriers or facilitators were specific to project or setting. Reported barriers encountered in using co-production approaches in acute healthcare settings included a lack of support, resources or managerial authority to bring about structural or environmental changes [30,31,32,34,40]. Practical or logistical problems included, for example, ensuring frail elderly people could attend regular co-design meetings [30]. Recruiting patients and carers and retaining them through the different stages of projects were highlighted by a number of researchers [29, 31-32, 34, 39]. In at least one project [31] some patients participated only in discovery or exploratory phases, then having 'shared' their experiences with staff, declined further participation arguing that they were no longer using the service. Researchers also identified the need to plan for and manage patients' understanding of what may be a radically different form of engagement with hospital staff, often quite unlike that experienced previously by users of health services[29,30,33]. In the studies reviewed, this was particularly in relation to the frequency and duration of their involvement and also in relation to the time it may take to bring about changes in the structures, processes and sometimes the physical environments of services [30,38,40].

In the majority of projects staff engagement was in addition to usual clinical or managerial roles; a high level of interest and commitment to co-production activity was identified in almost all projects. However, this was impacted on in at least five projects by staffs' frustration at the expectation that they would undertake co-production/co-design work in their own time, that they could not allocate time out of their routine work and that additional support was often not provided by more senior staff [30-32,34, 40]. For some staff this made participation almost impossible [34]; for others it meant projects did not progress as expected [32] or contributed to tensions in co-design groups or between researchers and participants [30]. The duration of projects also increased the likelihood that staff turnover would impact on project leadership or involvement [31-32]. The need for structured and on-going managerial and organisational support was highlighted [29-31, 33, 35, 39,40] but only two studies expressly refer to governance or oversight groups set up to support co-design projects [30-31].

In projects where facilitators were engaged formally and funded to manage or oversee projects, it was more likely that projects (a) maintained momentum and were delivered as planned, (b) engaged and retained patients, carers and staff and (c) generated concrete examples of areas where patients'

For peer review only - http://bmjopen.bmj.com/site/about/guidelines.xhtml

or carers' experiences could be improved [29,32-33,39]. In some studies, researchers (some of whom were designers) facilitated staff and patient engagement in the projects. Where designers were directly involved, our review suggests that they introduced ways of thinking and working which successfully challenged staff and patients to reconceptualise everyday processes and activities. This was achieved using metaphor games, design experiments, visual storyboards, prototyping, future focus groups, and emotional mapping; approaches not familiar to most health service staff [29-30, 35, 38]. However, with the exception of Locock et al [29] the costs associated with facilitator or researcher involvement are not identified.

Despite the barriers identified above, the studies suggest researchers and participants across settings viewed the benefits of this level of patient and staff involvement in structured co-production/co-design projects as outweighing the challenges.

Sustainability

A number of concrete changes in service organisation, care environments and patient and in carer experiences occurring either during or closely related to co-production activities were reported, see table 3 [29,30-35,40]. However, none of the eleven studies explored whether co-production or co-design as a way of working had been sustained, or whether improvements made as a result of such approaches were sustained over any length of time. Bowen et al [30] indicate that some changes may take two years or more to effect (for example, changes in hospital signage and routing), a period of time long after staff and patients had ceased to be actively involved in specific projects; another study reported that planned changes may not be achieved at all despite extending the life of projects [32].

[**Insert table 3 here**]

DISCUSSION

This rapid evidence synthesis has summarised the peer reviewed evidence relating to the use of co-production approaches in focused quality improvement projects in the acute healthcare sector. It should be noted that many reports of co-production/co-design projects undertaken as local quality improvement work are reported in the grey literature; these have not been considered in this review. Different forms of co-production/co-design were most commonly reported and few criticisms of these methods were reported, in part reflecting an almost certain positive publication bias. However, critical reflections are emerging [34, 41,42]. These, in part explore the so-called 'dark side' of co-production recognising, for example: the potential for reproducing inequality as those who are most able to are those that co-produce; the risks of 'captured' co-production where participants are compelled, sometimes unknowingly, to co-produce; co-production as a substitution of labour (what Fotaki terms 'a race to the bottom in times of austerity' [41]); and co-production being appropriated as cover for inherently political decisions.

The kind of patient focused quality improvements reported in the identified studies can largely be considered examples of simple practical changes or 'sweating the small things' [43]. However, from the perspective of patients' experiences of and journeys through a service, these issues were likely to make care and treatment more humane and more person centred, as well as making journeys easier to engage with and negotiate. However, both Bowen et al [30] and Thomson et al [35] argue that the ideation tools in EBD and EBCD were limited and that incorporating designerly thinking into co-production projects could stimulate greater creativity in designing alternative solutions in sometimes rigid health services.

The relative lack of robust evaluations we found may also reflect the stage of adoption of these types of methods in this specific setting; as we report, there is currently only one published

For peer review only - http://bmjopen.bmj.com/site/about/guidelines.xhtml

feasibility RCT. However, there is a large-scale stepped wedge randomised controlled trial under way in nine Australian community mental health teams which is evaluating the effect of co-design on psychosocial recovery outcomes (such as willingness to ask for help and personal confidence and hope) [44]. Robust and routine evaluation of the costs and impacts of using co-production methods are required so that those leading and participating in quality improvement efforts in acute healthcare organizations can make informed decisions regarding their adoption in addition to (or instead of) other quality improvement methods. However, reliance on relatively broad and insensitive measures - such as patient satisfaction - may overlook the real value placed by patients and staff on changes in the personal behaviour, attitudes, and culture of healthcare teams. The studies reviewed here consistently referred to these service level benefits of co-production for participants and services, as well as pointing to potential service system level effects such as those identified by Osborne et al [8].

As others have highlighted, the use of similar approaches in the community health care sector have not always 'follow[ed] through to significant and sustainable redesign' [5]. A recent narrative review of four different models of co-creation relevant to community-based health services proposed three 'key success principles', namely (1) a systems perspective (assuming emergence, local adaptation, and nonlinearity); (2) the framing of research as a creative enterprise with human experience at its core; and (3) an emphasis on process (the framing of the programme, the nature of relationships, and governance and facilitation arrangements, especially the style of leadership and how conflict is managed). The review authors proposed that co-creation 'failures' could often be tracked back to abandoning (or never adopting) these principles [5]. The publications in this RES do not specifically identify these principles and provide no clear evidence that such principles underpinned the planned research and service improvements noted. However, individually and collectively, included studies highlighted problems which arose when explicit adoption of such principles was not evident in the set up and delivery of the reported co-production projects in acute healthcare settings. Although the acute healthcare sector faces challenges associated with rapid patient turnover and with patients sometimes critically or terminally ill, the majority of studies in this RES demonstrate that co-production is possible and valued by participants. These findings have helped in planning for the introduction of the EBCD approach in the CREATE study. Although none of the studies reviewed were conducted in stroke services, the facilitators and barriers identified in the RES, together with the principles identified in the Greenhalgh et al review [5] indicate key areas for our research team to address with managers, staff, patients and relatives participating in the EBCD project activity.

Whilst, the potential benefits of using co-production in acute healthcare settings have been identified these must be set against the concerns identified by participants in the studies we reviewed that such approaches can be challenging to set up and implement in busy clinical environments where no formal, practical and financial provision is made for staff, patient and carer involvement on a sustained basis. Accelerated approaches may offer a partial solution but organisation level understanding of and commitment to these kinds of service improvement methods - as also identified by Greenhalgh et al [5] - remains key to their likely effectiveness.

CONCLUSIONS

We have identified a lack of rigorous evaluation of effectiveness and cost effectiveness of co-produced interventions in the acute healthcare sector at both the service and system level. Traditionally, co-production has been applied in community settings but there is growing recognition of the value of a more collective contribution to quality improvement work from those who are both delivering and receiving any form of public service [12,45]. At the clinical microsystem level within the healthcare sector there has been increasing adoption of co-production involving patients and staff, typically drawing on co-design approaches, as a means of improving the quality of frontline services. Health services, whether public or private, operate within increasingly severe financial restraints where funding for quality improvement work is limited. Without robust critique, evaluation and evidence of the co-creation of value there is a danger of co-production becoming

For peer review only

For peer review only - http://bmjopen.bmj.com/site/about/guidelines.xhtml

another management fad or fashion [46], with the meaning of the term itself appropriated and co-opted but no longer 'co-produced'.

Acknowledgements:

This project is funded by the National Institute for Health Research Health Services and Delivery Research Programme (project number 13/114/95). The views and opinions expressed therein are those of the authors and do not necessarily reflect those of the Health Services and Delivery Research Programme, NIHR, NHS or the Department of Health.

The arguments presented in this paper were partly informed by discussions during a workshop at the University of Nottingham on 7-8 July 2016 organised by Professor Andrew Gray and Professor Bruce Stafford: 'The Individual and the State: co-production and interdependency in the mixed economy of public service consumption'.

Our thanks go to Deirdre Andre and Sally Dalton, Information Specialists at the University of Leeds who helped refine the search terms and strategy and conducted the database searches. Tino Kulnic assisted with the initial screening of abstracts. Stephanie Honey and Parminder Dhiman, research fellows, also assisted with some of the data extraction.

Declaration of competing interests:

We have read and understood BMJ policy on declaration of interests and declare that we have no competing interests.

Author contributions:

DJC, FJ, RH and GR designed the rapid evidence synthesis and developed the search terms. GR conducted hand searches of the journals: CoDesign, The Design Journal, and Design Issues. DJC and FJ screened all abstracts and agreed on full text for review. DJC, RH and FJ reviewed all full texts and agreed inclusions and exclusions. DJC, FJ and RH independently completed data extraction and quality assessment for all included papers. DJC prepared the manuscript and GH, RH and FJ reviewed and revised the manuscript. All authors agreed the final version of the manuscript.

References

1. Realpe A and Wallace LM. What is co-production? London; The Health Foundation, 2016 http://personcentredcare.health.org.uk/sites/default/files/resources/what_is_co-production.pdf, accessed 19th August 2016
2. Boyle D, Clarke S and Burns S. Aspects of co-production: the implications for work, health and volunteering. London: New Economics Foundation 2006.
3. Needham C and Carr S (2009). SCIE Research briefing 31: co-production: an emerging evidence base for adult social care transformation. London: Social Care Institute for Excellence. http://www.scie.org.uk/publications/briefings/briefing31/ Accessed 22nd September 2016
4. Boyle D, Clarke S and Burns S (2006). Hidden work: co-production by people outside paid employment. London: Joseph Rowntree Foundation 2006.
5. Greenhalgh T, Jackson C, Shaw S. et al Achieving Research Impact Through Co-creation in Community-Based Health Services: Literature Review and Case Study. *The Milbank Quarterly*, 2016 94: 392–429.
6. Donetto S, Pierri P, Tsianakas V et al . 'Experience-based Co-design and healthcare improvement: realising participatory design in the public sector', *The Design Journal*, 2015 18(2): 227-248

For peer review only - http://bmjopen.bmj.com/site/about/guidelines.xhtml

7. Bovaird T (2007). 'Beyond engagement and participation: user and community co-production of public services'. *Public Administration Review*, 2007; 67: 846–860

8. Osborne SP, Radnor Z and Strokosch K Co-production and the co-creation of value in public services: a suitable case for treatment? *Public Management Review*, 2016 18(5): 639-653

9. Boyle D, Coote A, Sherwood S et al Right here, right now. Taking co-production into the mainstream. Discussion Paper. London, NEF/NESTA 2010.

10. Scottish Co-production Network. Co-production – how we make a difference together, http://www.coproductionscotland.org.uk/resources/, accessed 19th August 2016

11. Fugini M, Bracci E and Sicilia M. Co-production in the Public Sector. Experiences and Challenges. London: Springer 2016.

12. Batalden M, Batalden P, Margolis P et al. Co-production of healthcare service. *BMJ Qual Saf* BMJ Qual Saf 2016;25:509-517.

13. Berwick DM. Era 3 for Medicine and Health Care. *JAMA*. 2016;315(13):1329-1330. doi:10.1001/jama.2016.1509

14. Voorberg, W.H. Bekkers, V. J. J. M. Tummers, L. G. (2014): A Systematic Review of Co-Creation and Co-Production: Embarking on the social innovation journey, *Public Management Review*, 2015; 17(9): 13335-1357

15. Sangiorgi, D. (2011). 'Transformative services and transformation design'. *Int Journal of Design*, 2011; 5: 29–40.

16. Sangiorgi D and Prendiville A (eds.). (In press) Designing for Service: Key issues and new directions. Bloomsbury Publishing; London

17. Bate SP and Robert G. Bringing user experience to health care improvement: the concepts, methods and practices of experience-based design. Oxford; Radcliffe Publishing 2007

18. Bate SP and Robert G. 'Experience-based design: from redesigning the system around the patient to co-designing services with the patient', *Qual & Safin Health Care*, 2006; 15(5): 307-310

19. Robert G, Cornwell J, Locock L, et al Patients and staff as co-designers of healthcare services. *BMJ* 2015; 350:g7714

20. Jones F, Clarke D, Robert G et al. Using co-production to improve patient carer and staff experiences in health care organizations: a multi-centre, mixed methods evaluation in inpatient stroke units. NIHR- HS&DR Project 2016 13/114/95, http://www.nets.nihr.ac.uk/__data/assets/pdf_file/0010/162469/PRO-13-114-95.pdf

21. Featherstone RM, Dryden, D.M., Foisy, M. et al. Advancing knowledge of rapid reviews: an analysis of results, conclusions and recommendations from published review articles examining rapid reviews. *Syst Rev* 2015 4(1):50. DOI 10.1186/s13643-015-0040-4

22. Khangura, S., Konnyu, K., Cushman, R., et al. Evidence summaries: the evolution of a rapid review approach. *Syst Rev* 2012; 1(1), DOI:10.1186/2046-4053-1-10

23. Ganann, R., Ciliska, D., & Thomas, H. Expediting systematic reviews: methods and implications of rapid reviews. *Implementation Science*, 2010 5: 56. DOI: 10.1186/1748-5908-5-56

24. Bevan, N. Connelly,N., Francis,J., et al Rapid Evidence Assessment Toolkit. Civil Service. http://webarchive.nationalarchives.gov.uk/20140509095856/http:/www.civilservice.gov.uk/networks/gsr/resources-and-guidance/rapid-evidence-assessment 2010 Accessed 5th February 2016

For peer review only - http://bmjopen.bmj.com/site/about/guidelines.xhtml

25. NICE Methods for the development of NICE public health guidance (3rd edition). https://www.nice.org.uk/article/pmg4/chapter/1%20introduction 2010 Accessed 5th February 2016

26. Sandelowski M, Voils CI, Barrosso J Defining and Designing Mixed Research Synthesis Studies. *Research in the Schools* 2006 13 (1): 29

27. Boger E, Ellis J, Latter S, et al. (2015) Self-Management and Self-Management Support Outcomes: A Systematic Review and Mixed Research Synthesis of Stakeholder Views. *PLoS ONE* 2015 10(7): e0130990.

28. Tsianakas V, Robert G, Richardson A, et al. (2015) 'Enhancing the experience of carers in the chemotherapy outpatient setting: an exploratory randomised controlled trial to test the impact, acceptability and feasibility of a complex intervention co-designed by carers and staff', *Supportive Care in Cancer* 2015 23(10):3069-80

29. Locock, L Robert G, Boaz A, et al, Using a national archive of patient experience narratives to promote local patient-centered quality improvement: an ethnographic process evaluation of 'accelerated' experience-based co-design. *Journal of Health Services Research &Policy* 2014, 19(4) 200–207

30. Bowen, S. McSeveny, K, Lockley, E et al, How was it for you? Experiences of participatory design in the UK health service. *CoDesign* 2013 9:4, 230-246

31. Piper, D. Ledema R, Gray J, et al, Utilizing experience-based co-design to improve the experience of patients accessing emergency departments in New South Wales public hospitals: an evaluation study. *Health Services Management Research* 2012;25: 162 – 172

32. Larkin, M Boden, ZVR, Newton, E et al, On the Brink of Genuinely Collaborative Care: Experience-Based Co-Design in Mental Health. *Qualitative Health Research* 2015, 25(11) 1463–1476

33. Tsianakas,V Robert G, Maben J, et al, Implementing patient-centred cancer care: using experience-based co-design to improve patient experience in breast and lung cancer services. *Supportive Care in Cancer* 2012 20:2639–2647

34. Vennik,D van de Bovenkamp, H.M. Putters, K et al, Co-production in healthcare: rhetoric and practice. *Int Rev Administrative Sciences* 2016; 82(1) 150–168

35. Thomson A, Rivas C, Giovannoni G. Improving the quality of participant interaction and ideation tools within service improvement activities. *BMC Health Services Res*, 15:105

36. Palumbo, R. "Contextualizing co-production of health care: a systematic literature review", *Int Journal of Public Sector Management* 2015 29 (1): 72 – 90

37. Ream E, Tsianakas V, Verity R et al Enhancing the role of carers in the outpatient chemotherapy setting: a participatory action research project. Final report. London; King's College London 2013 http://www.dimblebycancercare.org/wp-content/uploads/2013/09/Dimbleby-report-executive-summary-pdf-3rd-Sept.pdf, accessed 20th August 2016

38. Golden.B.R. Hannam, R., Fraser H. et al, Improving the patient experience through design. *Healthcare Quarterly* 2011. 14(3)32-41

39. Tollyfield, R. Facilitating an accelerated experience-based co-design project. *Brit Journal of Nurs*, 2014, 23, (3):136-141

40. Boyd. H. McKernon, S, Mullin, B et al, Improving healthcare through the use of co-design. The *New Zealand Medical Journal* 2011 125,76–87

41. Fotaki, M. Co-Production under the financial crisis and austerity. A means of democratizing public services or a race to the bottom? *Journal of Management Inquiry* 2015: 24(4): 433-438.

For peer review only - http://bmjopen.bmj.com/site/about/guidelines.xhtml

42. Meijer A. Coproduction as a structural transformation of the public sector. *Int Journal of Public Sector Management* 2016 29(6): 596-611

43. Moore C and Buchanan D. Sweat the small stuff: a case study of small-scale change processes and consequences in acute care. *Health Serv Manage Res* 2013; 26: 9–9.

44. Palmer V, Chondros P, Piper D, et al. The CORE Study protocol: a stepped wedge cluster randomized controlled trial to test a co-design technique to optimize psychosocial recovery outcomes for people affected by mental illness. *BMJ Open* 2015 5:e006688 DOI:10.1136

45. Robert G and MacDonald A. (In press) 'Infrastructuring for organisational creativity and quality improvement in the healthcare sector: 'designerly' or 'design-like?'. In: D Sangiorgi and A Prendiville (eds). Designing for Service. Contemporary Issues and Novel Spaces, London: Bloomsbury

46. Walshe K. Pseudoinnovation: the development and spread of healthcare quality improvement methodologies, *International Journal for Quality in Health Care* 2009, 21 (3) 153-159

For peer review only

For peer review only - http://bmjopen.bmj.com/site/about/guidelines.xhtml

Table 3: Reported outcomes of co-production in acute healthcare settings

Type of outcomes	Evidence synthesis	Studies	Example
**Patient & staff involvement**	Patients perceived being really listened to by staff, having the sense that involvement was genuinely collaborative and that staff were committed to working with patients to improve services.	29,30, 31,33,39	Key to success was thought to be the strong relationship between patients and staff that has been built over time. (33)<br>Co-production' approach was seen to give patients, carers and staff a deeper understanding of each others' experience of the processes of care. It provided an opportunity to work meaningfully together, and this strengthened relationships among those involved in EBCD (31)<br>*'It was good, it felt as though [the hospital] and the professional staff were really interested … There was a comradeship if that's the right word …' (Older Patient- 30p)*
	Patients and staff recognised levels of participation and collaboration were distinct from any quality improvement activity they had experienced previously.	30,31,33,34	EBD's techniques facilitated building trust and rapport (30)<br>EBCD was perceived as a significantly higher level of genuine direct patient and carer involvement (relative to other service improvement projects in which patients and staff had participated) - *'the constant feedback [being] really, really useful', and '[needing] the eyeballing of each other to make it work'.* (33)<br>The EBCD process was thought to have given patients a greater sense of direct responsibility for the work and its outcomes (33, 34) and led to an increased understanding of the 'other' perspective, resulting in a potentially broader cultural change in mind-sets and behaviour. Staff participating in the co-design groups reported a greater sense of empowerment to make changes to their service (33).<br>Allowed nurses to reconnect with their core values of caring and compassion; development of a sense of empathy with patients was also evident.
	Staff realised that they often really did not have a good understanding and insight into patients' experiences of the services they provided.	33,34, 39, 40	Letting their 'own' patients talk about their experiences in a common 'action' setting affects professional staff, as patient stories make visible what the impact of (already known) problems really is on patients. The active role of patients and the way they express their experiences creates a sense of urgency to act on the improvement issues raised. (34)<br>Staff identified that working with patients in this honest and open sharing process was moving and at times humbling (33).
	There were also negative perceptions reported by a small number of patients and staff. These related to questioning the value of the amount of staff and patient time invested, and to not being able to see or experience actual	30,31	*'[The project] Seemed to go on for a long time . . . 'and I don't think we were properly prepared for that . . . my initial impression was that it would be perhaps a couple of interviews, and a discussion [Older patient-30:p]*<br>Both patients and staff identified that some service changes did not happen e.g. 'not sure if it has had any effect' (patient), 'in some areas, the area has not been improved' (staff), 'there's a few changes … [but] … there was some really serious things … they were highlighted, there was no resolution' (staff) (30)<br>Authors noted that although consensus reached quite easily, staff groups had tendency to focus on more positive

For peer review only - http://bmjopen.bmj.com/site/about/guidelines.xhtml

	service changes.		aspects of care and recent developments/improvements whereas service users and carers were more openly dissatisfied(31).
	Negative staff experiences and perceptions were also evident; these all related to challenges in conducting co-production or co-design projects.	30,31, 32, 34,40	Staff (and patients) reported little or no change had occurred. *'there's got to be something at the end of it'* (Staff Member-30p).<br>Staff viewed EBCD as a burden, additional to an already busy schedule (31,34).<br>Staff workloads increased where EBCD was additional to usual roles (30, 32, 34, 40).<br>Lack of time and high level organisational support for Steering Group to complete implementation plans (32).<br>Lack of organisational capacity to respond to 'bottom up' service development (32).<br>Fee for service systems do not directly reward (or encourage) EBCD/co-production projects (34).
**Generating ideas & suggestions for changes to processes, practices & clinical environments**	The number of ideas generated or issues highlighted ranged from 5-11 core issues, through 48 co-design activities to 400 ideas generated by staff in an oncology service project.		
	A significant number of factors affecting patient experience from across the range of acute healthcare settings related to the structure and process of care and service provision.<br>Changes spanned:<br>-information and communication processes,<br>-environmental and organisational changes (and to a lesser extent)<br>-staff training and development.	30,33,37,41, 34, 40, 32	New template for patient letters (30)<br>A patient journey map and accompanying journey guide was developed linked to a patient held record (40).<br>A clinic guide and clinic (terms) dictionary were developed for new patients (35).<br>Redesigned discharge summary, improved cross site information booklet for patients transferring to another hospital for surgery (29)<br>Patient focused visual journey guide (40),<br>Proposals for layout of roads surrounding the outpatient building, design proposals for new way-finding materials (signage and maps) (30).<br>Guidance on diagnosis procedures included in junior doctors' induction (33).<br>Informal staff training programme-'recent developments in' (35).<br>A co-design toolkit and website for other services in New Zealand(40).

For peer review only - http://bmjopen.bmj.com/site/about/guidelines.xhtml

	Acute hospital admission and care have impacts beyond individual patients; involvement of families and carers - evident in five of the studies - appears to have helped ensure that those involved in the patients journey were also able to comment on and shape that experience.	28, 32, 33,	Administrative staff receive customer care training watch the patient experiences) DVD (33).<br>Ensuring that when carers came to visit, they were greeted appropriately at the door of the unit (32)<br>Clinic structures changed to reduce waiting times (33).<br>Carers information leaflet increases knowledge and understanding of symptoms and side effects and carers confidence (28).
	The structured co-production and co-design methods employed empowered both patients and staff to collaboratively review services and to plan and undertake changes in their workplaces.	29, 30, 31,33, 35,40,	Despite concerns about activities being '*a bit daunting at first*', participants supported researchers' perceptions that sharing experiences via storytelling and emotional mapping helped to build empathy and cohesion in the project group (30).<br>The opportunity to hear directly from patients and carers reportedly had a transforming effect on some staff. *'Our data suggest face-to-face encounters with patients in co-design groups often had a profound effect on staffing making them think differently about their practice and reconnect with their core professional values, resulting in renewed motivation* (29p).
	Involving designers/design researchers in co-production projects added value though challenging staff and patients way of thinking about their healthcare journey and experiences and how these could be improved within existing service settings. However, as design led techniques may be unfamiliar to staff and patients these and their purpose need to be carefully explained if they are not be seen as time wasting or games.	30,35,38	Forum theatre training to improve staff awareness of 'customer care' (30).<br>Use of metaphor, analogy and prototyping techniques (travel documents linked to an ideal patient journey). The props and the analogy facilitated the co-design process by enabling participants to not just suggest solutions to problems but create and share more radical suggestions for change (such as initiating a Patient Advisory Group) (35).<br>Photojournalling and storyboarding (38)
**Tangible change in services & impact on patient or carer experiences**	Patient and carer experience: Overall patients reported positive experiences of participation. Despite the barriers identified participants across settings viewed the benefits of patient and staff involvement in structured co-production/co-design projects as outweighing the challenges.	28, 29,31,33,40	Carers who received the co-designed intervention reported significantly better understanding of symptoms and side effects and that their information needs were more often met than the control group. Confidence in coping improved between baseline and follow-up for the intervention group and declined for the control)(28)<br>Patients can opt to receive chemotherapy treatment information in a group with others having the same treatment (33).<br>More comfortable V shaped pillows for post-operative patients (29).<br>Changed process for porters to remove waste avoiding ICU rest times (29)

For peer review only - http://bmjopen.bmj.com/site/about/guidelines.xhtml

			Provision of free refreshments (patients and families) (31).<br><br>New design for mammography gown (40).
	Service changes (processes and environmental change): It was not always possible to distinguish changes that had been implemented from those which were planned. Reporting of service improvement outcomes per-se was a limitation of the included studies, but the projects reported largely indicated that the structured co-production and co-design methods employed empowered both patients and staff to collaboratively review services and to plan and undertake changes in their workplaces.	29,31,33,34,35,40	Introduction of mini-Schwartz rounds on ICU (29).<br><br>New private room identified for receiving support after diagnosis (29).<br><br>Installation of a new air-conditioning unit in the waiting room, installation of a blanket warming cupboard, installation of payphones in waiting areas (31)<br><br>Changes to structure of clinics to reduce waiting times (33).<br><br>Decoration of waiting rooms (34)<br><br>Volunteer in the outpatient clinic linked to a patient advisory group (35).<br><br>Psychosocial care offered proactively during consultations (one hospital) (34).<br><br>Patient held record to track appointments (40)

For peer review only - http://bmjopen.bmj.com/site/about/guidelines.xhtml

For peer review only

For peer review only

For peer review only - http://bmjopen.bmj.com/site/about/guidelines.xhtml

For peer review only

For peer review only - http://bmjopen.bmj.com/site/about/guidelines.xhtml

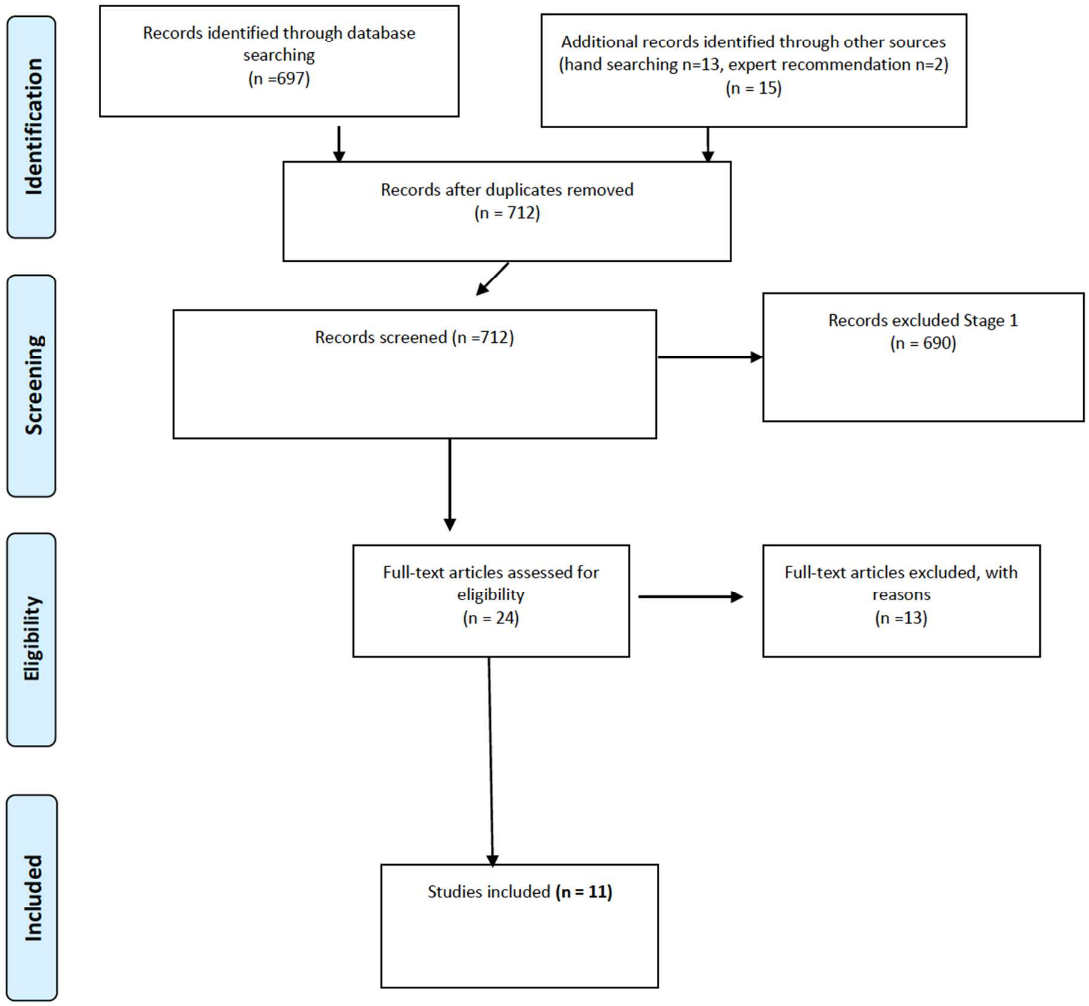
Identification
Records identified through database searching (n =697)
Additional records identified through other sources (hand searching n=13, expert recommendation n=2) (n = 15)
Records after duplicates removed (n = 712)
Screening
Records screened (n =712)
Records excluded Stage 1 (n = 690)
Eligibility
Full-text articles assessed for eligibility (n = 24)
Full-text articles excluded, with reasons (n =13)
Included
Studies included (n = 11)

Supplementary file 1 Search strategy (MEDLINE example)

Database: Ovid MEDLINE(R) <1996 to November Week 3 2015>

----

1 co-produc*.tw. (1149)

2 coproduc*.tw. (620)

3 co design*.tw. (53)

4 codesign*.tw. (16)

5 co creat*.tw. (171)

6 cocreat*.tw. (52)

7 (co research and design).tw. (1)

8 experience based design*.tw. (7)

9 participatory design*.tw. (149)

10 (experience adj3 design).tw. (252)

11 (evidence* adj2 design*).tw. (550)

12 EBC?.tw. (1604)

13 collaborative design.tw. (44)

14 human centred design.tw. (10)

15 human centered design.tw. (38)

16 people centred design.tw. (0)

17 people centered design.tw. (0)

18 inclusive design.tw. (32)

19 practice led design.tw. (1)

20 practice based design.tw. (2)

21 interactive design.tw. (31)

22 open design.tw. (147)

23 user centred design.tw. (51)

24 user centered design.tw. (177)

25 or/1-24 [co-design] (5093)

26 (acute adj (setting* or hospital* or care or healthcare)).tw. (15023)

27 ((secondary or speciali?ed) adj care).tw. (4410)

28 hospital care.tw. (4817)

29 exp Hospitals/ (117870)

30 Hospitalization/ (52132)

31 (hospitali?ation* or hospitali?ed).tw. (119506)

32 Inpatients/ (12602)

33 inpatient*.tw. (52413)

For peer review only

For peer review only - http://bmjopen.bmj.com/site/about/guidelines.xhtml

34 emergency medicine/ (8188)

35 Emergency treatment/ (8750)

36 emergency hospital admission*.tw. (240)

37 emergency hospitali#ation.tw. (116)

38 critical care/ (14911)

39 ambulatory care/ (20495)

40 urgent care.tw. (960)

41 or/26-40 [acute terms] (343100)

42 Delivery of Health Care/ (45257)

43 Efficiency, Organizational/ (17793)

44 Efficiency/ (4714)

45 Health Services Research/ (23584)

46 "Outcome Assessment (Health Care)"/ (50681)

47 patient outcome assessment/ (1713)

48 exp Program Evaluation/ (54529)

49 Quality Assurance, Health Care/ (38609)

50 Quality Improvement/ (9440)

51 Quality Indicators, Health Care/ (11744)

52 Quality of Health Care/ (43880)

53 (bench mark* or benchmark*).tw,kf. (17315)

54 (delivery adj2 health*).tw,kf. (12585)

55 (efficien* adj2 (assess* or assurance* or evaluat* or improv* or indicat* or measur* or test*)).tw,kf. (23714)

56 (performance adj2 (assess* or assurance* or evaluat* or improv* or indicat* or measur* or test*)).tw,kf. (74378)

57 (outcome* adj2 (assess* or assurance* or evaluat* or improv* or indicat* or measur* or test*)).tw,kf. (256084)

58 (program* adj2 (assess* or assurance* or evaluat* or improv* or indicat* or measur* or test*)).tw,kf. (27836)

59 or/42-58 [outcome or evaluation terms] (611767)

60 25 and 41 and 59 [co-design and acute care and outcome or evaluation terms] (45)

61 exp animals/ not humans.sh. (2031513)

62 60 not 61 [human only studies] (45)

63 limit 62 to (english language and yr="2005 - 2015") (40

For peer review only

Supplementary file 2 Empirical studies included in review

Publication/ country	Form of co-production	Setting	Methods	Reported Outcomes	Barriers and facilitators	Health economic evaluation	Quality rating
Bowen, S. et al, 2013 How was it for you? Experiences of participatory design in the UK health service. CoDesign, 9:4, 230-246<br><br>UK	Experience Based Design (EBD)	Medical outpatient service for older people. In a Teaching Hospital NHS Trust	Face to face (2) or telephone (9), semi-structured interviews (n= 11)	*Reported EBD Outcomes:*<br>• New template for patient appointment letters<br>• Design proposals for new way-finding materials (signage and maps)<br>• Proposal for layout of roads surrounding the outpatient building<br>• Video of patient stories distributed to all staff in the Trust to highlight older people's experiences,<br>• Forum theatre training event to improve outpatient staff awareness of customer care.<br>*Interview Findings*:<br>• Doing EBD (4 subthemes).<br>• Reflecting on EBD & Better Outpatient Services for Older People' (5 subthemes).	*Barriers:*<br>• Perception that participants were not doing the designing<br>• Perception that too many people were involved<br>• Ageing physical infrastructure Bureaucratic nature of trust procedures<br>• Low expectations of extent of change<br>• Availability of financial resources<br>• Staff workload<br>*Facilitators*:<br>• Sharing stories to gain perspectives of others<br>• Emotional mapping to highlight touchpoints (experiences and areas for improvement)<br>• Involvement in co-designing, being listened to by hospital staff	Not evident or reported.	++
Boyd. H. 2011 et al, Improving healthcare through the use of co-design. The New Zealand Medical Journal, 125,76–87	Experience Based Design (EBD) (Adapted)	Breast Services (inpatient & outpatient) in two hospitals in one District Health Board.	Patient journey mapping, experience surveys and co-design workshops.	• Patient journey map for staff review<br>• Patient Information folder to help navigate the service<br>• 7 new information leaflets,<br>• Patient held record to track appointments	*Barriers:*<br>• Staff workloads; EBD in addition to usual roles<br>• Financial restraints for hospitals and services<br>• Evaluation and planning for sustainability not factored into the	Not evident or reported.	+

For peer review only

Publication/ country	Form of co-production	Setting	Methods	Reported Outcomes	Barriers and facilitators	Health economic evaluation	Quality rating
New Zealand				• Patient focused visual patient journey guide<br>• Poster providing (better) communication guidance for staff and patients<br>• New design mammography gown,<br>• Co-design toolkit and website for other NZ services	EBD project<br>*Facilitators:*<br>• Engaging with patients at the outset of the co-design process before decisions are made<br>• Adopting a flexible approach to enable patients still receiving treatment to participate over time<br>• Working to get key staff buy in (clinical, managerial, administrative) & continued engagement<br>• Find & use ways to challenges participants' thinking, to work outside of their 'comfort zone'		
Golden.B.R. et al, 2011 Improving the patient experience through design. Healthcare Quarterly. 14(3)32-41<br><br>Canada	Rotman Creative Design Process<br>(co-design approach)	Cancer research and treatment centre.<br>Systemic therapy:<br>chemotherapy and transfusion services.	Ethnographic observations. Photojournalling & follow up interviews.<br>Staff discussion group: patient vignettes/ story-boarding ideal patient journey<br>Patient discussion group & storyboarding ideal patient journey.	• Prototypes and a jointly designed ideal patent journey	Not specifically reported.<br><br>Indicates staff workload and patient treatment scheduling pressures precluded bringing the two groups together to co-design service improvements.	Not evident or reported.<br><br>Project funded by charitable & corporate donations to the hospital & the University.	+
Larkin, M et al, 2015 On the Brink of Genuinely Collaborative Care: Experience-	Experience-based Co-Design<br>(EBCD)	Early Intervention Services and Inpatient units (n =7)	In depth interviews with service users (n=6), parents (n=6), inpatient staff (n=9).	Six priorities for service improvement identified from interviews and feedback group work:	*Facilitators:*<br>• Support from senior NHS staff<br>• Researchers leading the processes	Not evident or reported	+

For peer review only

Publication/ country	Form of co-production	Setting	Methods	Reported Outcomes	Barriers and facilitators	Health economic evaluation	Quality rating
Based Co-Design in Mental Health.Qualitative Health Research2015, Vol. 25(11) 1463–1476<br><br>UK	(adapted)	at two hospitals in the Midlands.<br><br>Mental health services for young people and families.	Feedback groups (20) involving 150 stakeholders.<br><br>Co-design event (50 participants).	• pathways in and out of hospital<br>• providing staff with a rewarding and well-supported role<br>• communicating with families and service-users<br>• recovery-focused practice<br>• creating a positive environment for everyone<br>• recognizing and sharing good practice across professions and services<br>• Co-design groups at the co-design event produced 13 action plans to address the 6 priorities	• Involvement of people internal and external to the service in the EBCD team aided project management<br>• Sensitivity to the needs and experiences of services uses in this setting in identifying touchpoints and developing trigger films to share with staff<br><br>*Barriers:*<br>• Challenging for some staff to hear strong service-users and carer views on services<br>• Lack of time and high level organisational support for Steering Group to complete implementation plans<br>• Steering group lacking the power to bring about change in organisations<br>• Organizational restructuring resulting in some study collaborators and "champions" leaving or changing role,<br>• Improvements requiring input from other departments or requiring strategic, budgetary, or staffing commitment<br>• Lack of organizational capacity to respond flexibly or directly to "bottom-up" service development		
Locock, L et al, (2014) Using a	Accelerated Experience-	In intensive care and	Process evaluation using ethnographic	*Re: AEBCD*	*Anticipated Barriers:*	In AEBCD the cost of	++

For peer review only - http://bmjopen.bmj.com/site/about/guidelines.xhtml

Publication/ country	Form of co-production	Setting	Methods	Reported Outcomes	Barriers and facilitators	Health economic evaluation	Quality rating
national archive of patient experience narratives to promote local patient-centered quality improvement: an ethnographic process evaluation of 'accelerated' experience-based co-design. Journal of Health Services Research &Policy2014, Vol. 19(4) 200–207<br><br>UK	based Co-Design (AEBCD)	lung cancer services in two English NHS hospitals	methods:<br>• observations of AEBCD processes and events (155 hours)<br>• interviews (n=30)<br>• repeated group interviews with patient participants<br>• reflective diaries (n=22)<br>• service improvement logs (n=4)<br>• evaluation questionnaires (post AEBCD events)	Trigger films developed through secondary analysis if existing films from the Oxford University collections of patient experience interviews were acceptable to patients and staff and were used successfully in subsequent co-design work.<br><br>*Re: Service improvements*<br>48 co-design activities across the four patient pathways. Examples reported were:<br>• Improved cross-site information booklet for patients transferring to another hospital for surgery<br>• A redesigned discharge summary with input from all professions<br>• More comfortable V-shaped pillows for postoperative patients<br>• Sourcing clocks to aid patient orientation in ICU<br>• Changed process for porters to remove waste avoiding ICU rest times<br>• New private room identified for receiving support after diagnosis<br>• Introducing mini 'Schwartz rounds' on ICU	Films developed from a national archive would be considered inappropriate or an inaccurate reflection of care in a specific hospital.<br><br>Barrier not evident: 85% of participants (patients and relatives) reported films to be good or excellent.<br><br>No other barriers reported<br><br>*Facilitators:*<br>• Regular face-to-face meetings with patients in co-design groups reminded staff of who change was for and why it mattered<br>• Co-design groups facilitated active and empowered partnerships between patient and staff	developing a trigger film was £8289, compared to £30,485 for EBCD.<br><br>Co-design<br>phase costs, were £20,276<br>(over half of this was facilitator salary).	

For peer review only

Publication/ country	Form of co-production	Setting	Methods	Reported Outcomes	Barriers and facilitators	Health economic evaluation	Quality rating
Piper, D. et al, 2012 Utilizing experience-based co-design to improve the experience of patients accessing emergency departments in New South Wales public hospitals: an evaluation study. Health Services Management Research2012;25: 162 – 172<br><br>Australia	Experience-based Co-Design (EBCD)	Emergency Department (EDs) & related services in two (programme) stages in seven public hospitals in New South Wales, Australia.	Review of EBCD process and evaluation reports provided by participating sites.<br><br>Interviews of patients, clinicians and managers, and also of high-level project managers including executive sponsors, clinical leads, project managers across sites (n=117)	EBCD processes provided opportunities to work together meaningfully and enabled patients, carers and staff to develop a deeper understanding of each other's experience of care processes, and this strengthened relationships among those involved in EBCD.<br><br>Examples of co-designed strategies implemented and sustained at the programme sites included:<br>• Recruitment of a full-time cleaner<br>• Installation of a new air conditioning unit in the waiting room<br>• Installation of a blanket warming cupboard<br>• Installation of food vending machines in the waiting area<br>• Access to free refreshments<br>• Installation of pay phones in the waiting area<br>• Patient access to 'sick-bay' bags in the waiting area<br>• Increased security presence in the emergency room.<br>22 examples are provided in the	*Barriers:*<br>• Staff viewed EBCD as a burden additional to already busy schedule<br>• Limits in implementation officers' & clinical leads' EBCD roles, reportedly reduced impact of programmes & long-term change<br>• Consumer recruitment & retention was difficult due to the transitory nature of the care episode<br>*Facilitators:*<br>• Develop a consumer engagement strategy, using on-going communication including face-to-face bi-monthly re-connect meetings, telephone & email contact/updates<br>• Integrating the EBCD process into everyday work practices rather than presenting EBCD as a separate task<br>• Adequate resources & reporting opportunities to executive meetings<br>• Clear accountability for implementation, allow adequate time to embed solutions into daily practice and cultures of EDs<br>• Appointing a liaison person to: ensure regular participant contact; manage governance of project & processes; communicate & contextualize aims & timeframes of EBCD to staff; develop a communication strategy to inform all stakeholders of the status & impact	Not evident or reported.	+

Publication/ country	Form of co-production	Setting	Methods	Reported Outcomes	Barriers and facilitators	Health economic evaluation	Quality rating
				publication.<br><br>Implementation strategies also identified.	of solutions on an on-going basis		
Thomson,A., Rivas,C., and Giovannoni. G. Multiple sclerosis outpatient future groups: improving the quality of participant interaction and ideation tools within service improvement activities. BMC Health Services Research (2015) 15:105. DOI 10.1186/s12913-015-0773-8<br><br>UK	Participatory approach drawing on techniques from the speculative design field.	An outpatient clinic for patients with multiple sclerosis in London, UK.	3 staged process using focus groups (future groups). In stages 1 and 3 patient participants and staff participants worked separately. In stage 2 the two groups were combined.<br><br>Process completed in 3 months<br><br>Speculative design techniques included: directed patient and staff journey mapping; combining patient and staff maps of an ideal (patient) journey (using analogies and props-a set of travel related documents); developing service improvements (represented on researcher developed separate patient and	• Volunteer in the outpatient clinic, linked to a Patient Advisory Group meeting 3 monthly<br>• A guide to the MS clinic for new outpatients<br>• An MS clinic dictionary (covering medical terms related to MS and frequently used in the clinic)<br>• A walking map with distances to the clinic (from the underground or disabled parking areas or local coffee shops)<br>• Magazine replenishment (as a clinic resource)<br>• Informal staff training programme (developments in MS research and care)	Facilitators:<br>• Adding (speculative) design led techniques to a participatory approach broadly similar to the 6 stage EBCD approach<br>• Adapting the design led props for the specific healthcare context<br>• Involving the design researcher directly in the (future) focus groups<br>• Including senior staff/managers in the stage 3 (future) focus groups to facilitate understanding of proposed service developments and their implementation.<br>Barriers:<br>• Adapting design led methods for health services improvements projects may reduce their creative contribution if used in a procedural manner<br>• Participant engagement with design led metaphor and analogy driven techniques need to be considered carefully and reflect local and disease specific contexts	Not evident or reported.	+

For peer review only

Publication/ country	Form of co-production	Setting	Methods	Reported Outcomes	Barriers and facilitators	Health economic evaluation	Quality rating
			staff conference style posters (prototyping).				
Tollyfield, R. 2014 Facilitating an accelerated experience-based co-design project. British Journal of Nursing, 2014, l23, (3):136-141<br><br>UK	Accelerated Experience-based Co-Design (AEBCD)	Specialist cardiothoracic intensive therapy unit (ITU) in London.<br><br>(One of the four units involved in the Locock et al 2014 study)	Reflection on the role of facilitator in an AEBCD project in one of two ITUs participating in the project.	Reports on the experience of and learning from facilitation of the AEBCD study conducted by Locock et al (2014).<br><br>• Promoting staff engagement a key aspect of the project- via posters, emails, one-to-one discussions and a ITU weekly newsletter<br>• Developing solutions to low patient recruitment levels<br>• Providing facilitation of the main project events and co-design meetings<br>• Prompting and supporting implementation of planned change	*Facilitators:*<br>• Project supported and endorsed by senior managers and clinicians- Director of Nursing, critical care service manager, ITU consultant physician<br>• Formal training in EBCD methods and on-going email and telephone support from staff experienced in using EBCD<br>• Access to and use of the online Kings Fund EBCD toolkit<br>*Barriers:*<br>• facilitator role time consuming and impacted significantly on time for clinical work (externally funded role for experienced ITU clinician at this site)<br>• learning about the AEBCD process whilst undertaking project organisational tasks and activities against a defined timeline	Not reported.<br><br>See Locock et al (above) for costs of facilitator salaries.	+
Tsianakas,V et al, 2012 Implementing patient-centred cancer care: using	Experience-based Co-Design (EBCD)	A large, inner-city cancer centre in	Ethnographic observation (219 hours)<br>Filmed narrative	*16 breast cancer working group outcomes including*:	*Barriers:*<br><br>Not specifically identified in the	Not evident or reported	++

For peer review only

Publication/ country	Form of co-production	Setting	Methods	Reported Outcomes	Barriers and facilitators	Health economic evaluation	Quality rating
experience-based co-design to improve patient experience in breast and lung cancer services. Supportive Care in Cancer (2012) 20:2639–2647<br><br>UK		England.<br><br>(Breast and lung cancer patients)	patient interviews (n=36)<br><br>Staff interviews (n= 63)<br><br>Post project interviews with<br><br>Staff (n=4)<br><br>and patients (n=5)	• Administrative staff receive customer-care training and are shown patients' DVD<br>• Healthcare assistants' interpersonal skills assessed prior to recruitment<br>• Managers and administrative staff use values-based performance tool which can improve patients' experience<br>• Changes to structure of clinics to reduce waiting times<br>• Patients receiving same chemotherapy treatment given option to receive information in a group<br><br>*11 lung cancer working group outcomes including:*<br><br>• Establishment of second breaking-bad-news room<br>• Guidance on diagnosis procedures included in junior doctors' induction<br>• Promotion of information and support centres at different sites (advertising at hospital entrance)<br>• Patient DVD 'welcome to cancer services' for newly diagnosed and	publication.<br><br>• As EBCD does not explicitly seek to recruit a representative sample of patients but those who are motivated to 'tell their story' and participate in the co-design work. This may lead to the experiences of 'harder to reach' patient groups remaining unheard.<br><br>*Facilitators:*<br><br>• Genuine and direct patient and carer involvement (relative to other service improvement projects in which patients and staff had participated).<br>• Patient participants taking direct responsibility for the work and its outcomes<br>• The EBCD change process itself together with the direct and active participation of staff and patients produced implementation and action, and contributed to spread and sustainability		

For peer review only - http://bmjopen.bmj.com/site/about/guidelines.xhtml

Publication/ country	Form of co-production	Setting	Methods	Reported Outcomes	Barriers and facilitators	Health economic evaluation	Quality rating
				referred patients			
Tsianakas,V., Robert ,G., Richardson,A., Verity,R., Oakley,C., Murrells,T., Flynn,M., Ream,E. Enhancing the experience of carers in the chemotherapy outpatient setting: an exploratory randomised controlled trial to test impact, acceptability and feasibility of a complex intervention co-designed by carers and staff . Support Care Cancer. 2015. DOI 10.1007/s00520-015-2677-x<br><br>UK	The study reports an RCT to test the impact and feasibility of a complex intervention (Take Care). The intervention was developed previously in the same hospital using a co-design approach – Experience-Based Co-Design.	Chemo-therapy day unit (breast, lung or colorectal cancer) in a London teaching hospital.	A two phase mixed methods design including i) an exploratory RCT (impact-measured through before and after postal questionnaire completion), ii) focus groups with carers and healthcare professionals (feasibility and acceptability)	• Carers in the intervention group had better understanding of the symptoms and side effects compared with the control group.<br>• Carers in the intervention group had more confidence in coping than the control group.<br>• There were no differences between the 2 groups for emotional well-being<br>• Health care professionals and carers confirmed the feasibility and acceptability of the EBCD derived Take Care intervention<br>• Patients and HCPs reported the intervention was educational, enhanced knowledge of symptoms and side effects, and increased confidence of carers.	Not reported in terms of EBCD<br><br>Reported limitations of the study included:<br>• Small sample size, no power calculation<br>• Exploratory trial in a single centre limits generalisability<br>• Self-report questionnaire responses may be subject to recall bias	Not evident or reported	+
Vennik et al, Co-production in healthcare:	Experience-based Co-Design (EBCD)	One haematolog y and four	Semi-structured interviews (n=27)	Improvement areas identified by patients were similar across hospitals. Apart from some specific	Barriers:<br>• Individual level- some physicians	Not evident or reported	++

For peer review only

**Publication/ country**	**Form of co-production**	**Setting**	**Methods**	**Reported Outcomes**	**Barriers and facilitators**	**Health economic evaluation**	**Quality rating**
rhetoric and practice. International Review of Administrative Sciences 2016, Vol. 82(1) 150–168 Netherlands	in 4 centres (Adapted) Four project teams planned to use EBCD methodology; all adapted the method. Two used focus groups to gather patient experiences instead of filmed interviews. Two did not interview professionals. One did not use co-design workgroups concluding that it would require too much from participants; staff were not immediately convinced of the added value of this	oncology departments in 5 Dutch hospitals	Observations (70 hours) Documentary analysis	experiences with medical treatments, they mostly concerned aspects of quality of care including: • Decoration of waiting rooms, • Waiting times, -Communication, and coordination with general practitioners. • Psychosocial care is now proactively offered during consultations at one hospital, • A dental care in oncology leaflet was introduced at another hospital Improvement issues identified were related to individual professions, hospital wards, the hospital organization and other healthcare providers.	afraid of being criticised by patients, • Organisational barriers-pressure of work and other project commitments, changes in staffing levels, service re-organisations during the 12 months projects • EBCD largely seen as 'additional work' • Local policies and national trends can impact on engagement with co-production, no staff time freed for co-production/co-design, staff expected to deliver treatment and care and then engage in service improvement in their own time. • Fee-for-service systems do not directly reward projects like EBCD/co-production Facilitators: • Motivated people who are willing to work on EBCD/co-production in their own time • Selecting the 'right patients', inviting patients who have a critical point of view, are mentally and physically well enough condition to participate, and able to tell their story. • It is not so much what patients say that is important; it is the process and the way they are given the opportunity to have their say (in EBCD/co-production) The active role of patients creates a sense of urgency		

Publication/ country	Form of co-production	Setting	Methods	Reported Outcomes	Barriers and facilitators	Health economic evaluation	Quality rating
	phase				to act on the improvement issues raised<br>• The process of co-production/EBCD stimulated staffs' thinking about how to realize quality improvements<br>• Keeping projects 'small', not attempting to improve everything at once and focusing<br>• Patients' input can gain legitimacy for improvement plans and facilitate implementation		

For peer review only

PRISMA 2009 Checklist

Section/topic	#	Checklist item	Reported on page #
**TITLE**			
Title	1	Identify the report as a systematic review, meta-analysis, or both.	4
**ABSTRACT**			
Structured summary	2	Provide a structured summary including, as applicable: background; objectives; data sources; study eligibility criteria, participants, and interventions; study appraisal and synthesis methods; results; limitations; conclusions and implications of key findings; systematic review registration number.	2
**INTRODUCTION**			
Rationale	3	Describe the rationale for the review in the context of what is already known.	3-4
Objectives	4	Provide an explicit statement of questions being addressed with reference to participants, interventions, comparisons, outcomes, and study design (PICOS).	4
**METHODS**			
Protocol and registration	5	Indicate if a review protocol exists, if and where it can be accessed (e.g., Web address), and, if available, provide registration information including registration number.	N/A
Eligibility criteria	6	Specify study characteristics (e.g., PICOS, length of follow-up) and report characteristics (e.g., years considered, language, publication status) used as criteria for eligibility, giving rationale.	5-6
Information sources	7	Describe all information sources (e.g., databases with dates of coverage, contact with study authors to identify additional studies) in the search and date last searched.	5
Search	8	Present full electronic search strategy for at least one database, including any limits used, such that it could be repeated.	Supp file1
Study selection	9	State the process for selecting studies (i.e., screening, eligibility, included in systematic review, and, if applicable, included in the meta-analysis).	6
Data collection process	10	Describe method of data extraction from reports (e.g., piloted forms, independently, in duplicate) and any processes for obtaining and confirming data from investigators.	6
Data items	11	List and define all variables for which data were sought (e.g., PICOS, funding sources) and any assumptions and simplifications made.	5-6
Risk of bias in individual studies	12	Describe methods used for assessing risk of bias of individual studies (including specification of whether this was done at the study or outcome level), and how this information is to be used in any data synthesis.	N/A
Summary measures	13	State the principal summary measures (e.g., risk ratio, difference in means).	N/A
Synthesis of results	14	Describe the methods of handling data and combining results of studies, if done, including measures of consistency (e.g., I^2) for each meta-analysis.	6

For peer review only

PRISMA 2009 Checklist

Section/topic	#	Checklist item	Reported on page #
Risk of bias across studies	15	Specify any assessment of risk of bias that may affect the cumulative evidence (e.g., publication bias, selective reporting within studies).	N/A
Additional analyses	16	Describe methods of additional analyses (e.g., sensitivity or subgroup analyses, meta-regression), if done, indicating which were pre-specified.	N/A
**RESULTS**			
Study selection	17	Give numbers of studies screened, assessed for eligibility, and included in the review, with reasons for exclusions at each stage, ideally with a flow diagram.	6 and 7
Study characteristics	18	For each study, present characteristics for which data were extracted (e.g., study size, PICOS, follow-up period) and provide the citations.	Supp file 2
Risk of bias within studies	19	Present data on risk of bias of each study and, if available, any outcome level assessment (see item 12).	N/A
Results of individual studies	20	For all outcomes considered (benefits or harms), present, for each study: (a) simple summary data for each intervention group (b) effect estimates and confidence intervals, ideally with a forest plot.	Supp file 2
Synthesis of results	21	Present results of each meta-analysis done, including confidence intervals and measures of consistency.	N/A
Risk of bias across studies	22	Present results of any assessment of risk of bias across studies (see Item 15).	N/A
Additional analysis	23	Give results of additional analyses, if done (e.g., sensitivity or subgroup analyses, meta-regression [see Item 16]).	N/A
**DISCUSSION**			
Summary of evidence	24	Summarize the main findings including the strength of evidence for each main outcome; consider their relevance to key groups (e.g., healthcare providers, users, and policy makers).	7-10 and Table 3
Limitations	25	Discuss limitations at study and outcome level (e.g., risk of bias), and at review-level (e.g., incomplete retrieval of identified research, reporting bias).	10-11
Conclusions	26	Provide a general interpretation of the results in the context of other evidence, and implications for future research.	
**FUNDING**			
Funding	27	Describe sources of funding for the systematic review and other support (e.g., supply of data); role of funders for the systematic review.	12

From: Moher D, Liberati A, Tetzlaff J, Altman DG, The PRISMA Group (2009). Preferred Reporting Items for Systematic Reviews and Meta-Analyses: The PRISMA Statement. PLoS Med 6(6): e1000097. doi:10.1371/journal.pmed1000097

For more information, visit: **www.prisma-statement.org**.

For peer review only - http://bmjopen.bmj.com/site/about/guidelines.xhtml

BMJ Open

What outcomes are associated with developing and implementing co-produced interventions in acute healthcare settings? A rapid evidence synthesis.

Journal:	*BMJ Open*
Manuscript ID	bmjopen-2016-014650.R1
Article Type:	Research
Date Submitted by the Author:	19-Feb-2017
Complete List of Authors:	Clarke, David; University of Leeds, Academic Unit of Elderly Care and Rehabilitation<br>Jones, Fiona; St Georges University of London,<br>Harris, Ruth; King\'s College London,<br>Robert, Glenn; King's College London,
**Primary Subject Heading**:	Health services research
Secondary Subject Heading:	Evidence based practice, Qualitative research
Keywords:	rapid evidence synthesis, co-production, acute healthcare, systematic review

SCHOLARONE™
Manuscripts

For peer review only

What outcomes are associated with developing and implementing co-produced interventions in acute healthcare settings? A rapid evidence synthesis.

Corresponding author:
Dr David Clarke
Associate Professor in Stroke Care,
Academic Unit of Elderly Care and Rehabilitation
Leeds Institute of Health Sciences
Temple Bank House
Bradford Royal Infirmary
Bradford
BD9 6RJ
Tel: 01274 383441
Mobile: 07503727990
Email: d.j.clarke@leeds.ac.uk

Professor Fiona Jones,
Professor of Rehabilitation Research,
Faculty of Health Social Care and Education
St George's University of London
2nd Floor Grosvenor Wing
Cranmer Terrace
London SW17 0RE
f.jones@sgul.kingston.ac.uk

Professor Ruth Harris,
Professor of Healthcare for Older Adults,
King's College London
Florence Nightingale Faculty of Nursing and Midwifery
Room 2.24, James Clerk Maxwell Building
57 Waterloo Road
SE1 8WA
ruth.harris@kcl.ac.uk
Tel: 020 7848 3708

Professor Glenn Robert,
Professor of Healthcare Quality & Innovation,
Kings College London
Florence Nightingale Faculty of Nursing and Midwifery
James Clerk Maxwell Building
Waterloo
United Kingdom
Telephone: +44 (0)20 7848 3063
E-mail: glenn.robert@kcl.ac.uk

on behalf of the Collaborative Rehabilitation in Acute Stroke (CREATE) team

Word count: 4989 excluding title page, abstract, references, figures and tables.

For peer review only

For peer review only - http://bmjopen.bmj.com/site/about/guidelines.xhtml

What outcomes are associated with developing and implementing co-produced interventions in acute healthcare settings? A rapid evidence synthesis.

Abstract

Background: Co-production is defined as the voluntary or involuntary involvement of users in the design, management, delivery and/or evaluation of services. Interest in co-production as an intervention for improving healthcare quality is increasing. In the acute healthcare context co-production is promoted as harnessing the knowledge of patients, carers and staff to make changes about which they care most. However, little is known regarding the impact of co-production on patient, staff or organizational outcomes in these settings.

Aims: to identify and appraise reported outcomes of co-production as an intervention to improve quality of services in acute healthcare settings.

Design: rapid evidence synthesis.

Data Sources: Medline, Cinahl, Web of Science, Embase, HMIC, Cochrane Database of Systematic Reviews, SCIE, Proquest Dissertation and Theses, EThOS, OpenGrey; CoDesign; Design Journal; Design Issues.

Study selection: studies reporting patient, staff or organisational outcomes associated with using co-co-production in an acute healthcare setting

Findings: 712 titles and abstracts were screened, 24 papers underwent full text review; 11 papers were included in the evidence synthesis. One study was a feasibility randomised controlled trial, three were process evaluations and seven used descriptive qualitative approaches. Reported outcomes related to: (a) the value of patient and staff involvement in co-production processes; (b) the generation of ideas for changes to processes, practices and clinical environments; and (c) tangible service changes and impacts on patient experiences. Only one study included cost analysis; none reported an economic evaluation. No studies assessed the sustainability of any changes made..

Conclusions: despite increasing interest in and advocacy for co-production there is a lack of rigorous evaluation in acute healthcare settings. Future studies should evaluate clinical and service outcomes as well as the cost-effectiveness of co-production relative to other forms of quality improvement. Potentially broader impacts on the values and behaviours of participants should also be considered.

Key words: Rapid evidence synthesis, systematic review, co-production, acute healthcare

For peer review only

Strengths and limitations of this study

This study is the first to systematically review outcomes associated with developing and implementing co-produced interventions in acute healthcare settings.

We have identified a lack of rigorous evaluation of effectiveness and cost effectiveness of co-produced interventions in the acute healthcare sector at both the service and system level.

The study did not access and review the much broader patient and public involvement, service improvement and clinical microsystems literatures unless specific terms such as 'co-production' and 'co-design' were used.

We only included studies where full-text reports published in English within the last 10 years could be retrieved; co-production studies published in other languages may have been overlooked by our review.

What outcomes are associated with developing and implementing co-produced interventions in acute healthcare settings: a rapid evidence synthesis.

BACKGROUND

There is renewed interest in and advocacy for adoption of 'co-production' as a means of co-creating value across the public sector. However, as the term increasingly enters mainstream management (and healthcare quality improvement) discourses there is a sense that it may be being misused or misunderstood, thereby running the risk of becoming denuded of meaning or losing any association with its radical roots.

Co-production was first conceptualised in the United States in the 1970s, originally as a response to the lack of recognition of service users in service delivery [1]. The creation of time banks, a system reliant on the participation of volunteers who are also service users [2,3] showed how collaborative interventions that involve people with long-term psychosocial needs could contribute to improved community links [4]. Since this early work a variety of terms have become evident in the growing literature on co-production, and a range of related approaches are being enacted in different ways and at various levels throughout public and health sector services [5-11]. For the purposes of this paper we follow Osborne et al recent definition of co-production as 'the voluntary or involuntary involvement of public service users in any of the design, management, delivery and/or evaluation of public services' [8].

In the healthcare context co-production is promoted as harnessing the knowledge of patients, carers and staff to make changes about which they care most [12-13]. A recent example of using co-production in this way is a project to deliver and evaluate a mental health service to meet the needs of black and minority ethnic communities [14]. Berwick has recently argued for a greater focus on such co-production as part of a proposed 'third era' of medicine [15]. Potential benefits might include from a patient perspective improved satisfaction with the experience of care, reduction in complications associated with treatment and improved health outcomes [12-14]. From a staff and organizational perspective, the case for co-production may include improvements in staff wellbeing, increased recognition of the need for a better understanding of patient perspectives and changes in attitudes towards working with patients as partners in quality improvement [12-15].

For peer review only - http://bmjopen.bmj.com/site/about/guidelines.xhtml

In a recent commentary Batalden et al, whilst arguing that where healthcare activities are co-produced, services, providers and service users become far more effective agents of change, note that current systems can both support and constrain partnerships between patients and professionals (and that historically this kind of partnership has been unequal) [12]. The evidence base in terms of robust evaluation of the impact of co-production on patient outcomes appeared limited. Batalden et al argue this raises a series of questions for proponents of co-production including the need to determine '[m]easures that accurately reflect co-produced service and its results [and] measures which will help us understand co-productive processes' [12: 7].

Osborne et al, starting from the premise that co-production is (or has become over time) a 'woolly word', helpfully critiqued its conceptualization within the policy and practice literature, linked it directly to the co-creation of value in public service delivery and proposed a framework for differentiating 'the cluster of related concepts that are contained within the term 'co-production' [8]. This framework comprises 'four ideal types of value that are co-created in public service delivery by the iterative interactions of service users and service professionals with public service delivery systems'; namely co-production, co-design, co-construction and co-innovation. Voorberg et al conducted a systematic review of the literature (1987-2013) relating to co-creation/co-production with citizens in public innovation across all sectors, concluding that most studies focus on the identification of influential factors, while hardly any attention was paid to outcomes [16]. Greenhalgh et al report a narrative review of different models of co-creation relevant to community-based health services; they identify key success principles but note that 'impact is by no means guaranteed' [5].

For the purposes of this rapid evidence synthesis (RES) we identify co-production and co-design as specific approaches to co-creating value at the service (or clinical microsystem [13]) level within an acute healthcare organization. Co-design is an approach to participatory design (albeit traditionally of a new product) that seeks to actively involve all stakeholders (e.g. staff, patients, citizens) in a process to help ensure the result meets their needs and is usable. Such approaches are increasingly being used, often situated in the emerging Service Design field [17-18] as part of co-production projects. Specific forms of co-design such as Experience Based Design (EBD) [19-20] and Experience-based Co-design (EBCD) [21] have been developed and applied in the healthcare sector. We also follow Osborne et al conceptualisation of their final two 'types of value' (co-construction and co-innovation) as being concerned with service systems (rather than individual services) [8]; we return to this important distinction at the end of this paper.

We undertook this RES partly to inform an ongoing study (Collaborative Rehabilitation in Acute Stroke- CREATE) which is bringing together staff, former patients and carers to review and jointly co-produce the way in which therapeutic rehabilitation-related activity is provided in stroke units in the early days and weeks after stroke [22]. In the absence of existing published evidence, this RES aims to identify and evaluate reported outcomes of co-produced interventions designed to achieve patient focused quality improvements in acute healthcare settings. To inform the empirical study our secondary objectives were to identify in such settings:

- those elements of co-production processes that lead to patient focused quality improvements;
- any evidence of cost-benefit analysis, cost reduction or other economic evaluation associated with co-produced interventions;
- barriers and facilitators reported to influence the implementation and use of co-production methods and implementation of co-produced interventions; and
- issues which impact sustainability of quality improvements following the use of co-production.

For peer review only - http://bmjopen.bmj.com/site/about/guidelines.xhtml

REVIEW METHODS

Whilst there are no agreed international guidelines for the design and conduct of RES [23] there is overall agreement the process involves providing an overview of existing research on a defined topic area, together with a synthesis of the evidence provided by these studies to address specific review questions. RES are typically completed in 2-6 six months; this does not normally allow for all stages of traditional effectiveness reviews [23-25]. However, RES are increasingly valued by policy makers who require rapid answers to specific questions to inform policy revision or service development [26-27].

Limitations of RES include the potential for introduction of bias. Sources of bias can include omission of critical appraisal of methodological quality of included papers. Similarly, limiting the scope of the search to published literature or specific databases, to a specific focus within a larger topic, or to a reduced timespan of publication can reduce the applicability of RES findings for some audiences. In RES of effectiveness, meta-analyses may be omitted. It is important therefore that RES are conducted systematically; methods used and limitations introduced by these should be reported transparently [23-26].

This RES was conducted between January and June 2016. We used a systematic approach and included full-text evidence published in English within the last 10 years. Search terms and databases searched were agreed by researchers and Information Specialists at the University of Leeds. Search terms used were specific to the use of co-production in acute healthcare settings (see supplementary file 1). To keep the search focused, we omitted broader search terms including cooperative behaviour, patient participation, collaborative approach, and service improvement.

Box 1: Database and hand searches

Medline, Cinahl, Web of Science, Embase, HMIC, Cochrane Database of Systematic Reviews, SCIE, Proquest Dissertation and Theses, EThOS and OpenGrey.<br><br>The following journals were hand searched:<br>CoDesign; The Design Journal; and Design Issues.

Database searches were conducted for the period January 1st 2005 to 31st January 2016. Given existing reviews [5, 16] and the CREATE study focus, we reviewed post 2005 evidence and only that reporting on studies in acute healthcare settings. We completed citation tracking of 5 seminal papers; five experts in co-production were requested to nominate 3-5 seminal papers relevant to our review. Inclusion criteria are shown in table 1:

Table 1: inclusion criteria

• Reports research using a co-creation or co-production, or co-design or Experience-Based Co-Design approach in an acute healthcare setting
• Reports patient or staff or organisational outcomes resulting from research using a co-creation, co-production, co-design, Experience-Based Co-Design approach in an acute healthcare setting<br><br>Outcomes of interest include:

For peer review only - http://bmjopen.bmj.com/site/about/guidelines.xhtml

- any measure of the outcome of co-produced interventions on patient focused quality improvements in acute healthcare settings as reported by patients or families or caregivers, or health service providers<br>- including patient reported outcome measures and patient or staff reported experience measures<br>- using qualitative or quantitative data
• Acute healthcare settings include:<br>- emergency departments/accident and emergency departments<br>- adult inpatient facilities including: acute medical or surgical admission units (often termed MAUs or SAUs), acute medical or surgical units, acute trauma units, acute neurological units, intensive or critical care units, acute care of the elderly or geriatric units, medical oncology or cancer services<br>- adult outpatient facilities including medical, surgical, trauma, neurology, care of the elderly or geriatrics, medical oncology or cancer services

Two reviewers independently read all titles and abstracts. Discrepancies in retain or reject decisions were addressed by discussion between reviewers with recourse to a third where consensus could not be reached. Three reviewers independently read the retrieved full text papers; initial decisions to retain or reject were made independently based on the inclusion criteria. All three reviewers then discussed retain and reject recommendations and reached a consensus agreement.

Three reviewers completed data extraction. We used quality appraisal checklists developed for quantitative and qualitative studies [28] (Table 2). These were selected as they were developed and used by the National Institute for Health and Care Excellence (NICE) in the appraisal of the quality of evidence underpinning NICE public health guidance. The checklists address fourteen areas of study quality ranging from theoretical approach through study design, data collection and analysis methods and also ethical review (examples in supplementary file 3). For each study two reviewers undertook data extraction and quality appraisal independently. Studies were not excluded on the basis of quality appraisal; this was used to inform the discussion of the evidence identified in the review. We adopted a mixed research synthesis approach [29] drawing on methods reported by Boger et al [30]. We used an integrated design which entailed grouping studies for synthesis not by methods (i.e., qualitative and quantitative) but rather by findings viewed as answering the same research questions, or addressing the same aspects of a target phenomenon.

RESULTS:

A total of 712 titles and abstracts were identified for screening from which 24 papers were identified for full text review. Following review, eleven publications were included in the final evidence synthesis (figure 1); full details of each study can be found in supplementary file 2.

Table 2: Quality Assessment study rating criteria [28]

++ All or most of the checklist criteria have been fulfilled, where they have not been fulfilled the conclusions are very unlikely to alter.
+ Some of the checklist criteria have been fulfilled, where they have not been fulfilled, or not adequately described, the conclusions are unlikely to alter.

For peer review only - http://bmjopen.bmj.com/site/about/guidelines.xhtml

– Few or no checklist criteria have been fulfilled and the conclusions are likely or very likely to alter.

Papers were published between 2008 and January 2016, with studies conducted in five countries (Canada, England, New Zealand, Australia, Netherlands). Settings included intensive care units (n=1), inpatient and outpatient oncology services, including breast, lung, colorectal and haematology services (n=5), mental health services (n=1), emergency departments (n=1), an outpatient clinic for people with multiple sclerosis (n=1), and older peoples outpatient services (n=1). A further study evaluated co-design projects in five Dutch hospitals; the projects were conducted in four oncology departments and a haematology department.

One study was a feasibility randomised controlled trial (RCT) of a co-designed intervention [31]. The study design was explicitly stated in only one of the remaining papers [32]; the remaining studies utilised descriptive qualitative approaches to evaluate changes in services or explore participant's experiences or views, although these were rarely described in detail. Data collection methods included self-report postal questionnaires; ethnographic observations of patient journeys through services and of staff working practices, semi-structured interviews with staff and patients (sometimes filmed), focus groups and emotional mapping exercises. Data analysis techniques were described in any detail in only six of the eleven publications [32-38]. Most papers focused on the processes used to understand and co-design within services rather than evaluating outcomes; the exception being the feasibility RCT [31].

Quality assessment ratings are shown in the final column of the table in supplementary file 2. Four studies were rated at ++ with the remaining seven at +. Ratings largely reflect the omission of detail on research methods, particularly in relation to stating research aims, questions, sampling decisions and discussion of data analysis and findings.

Figure 1: Adapted Prisma diagram insert here

Use of co-production methods

Methods reported included: a feasibility RCT of the impact of a co-designed intervention [31], participatory design [38], creative design [39], co-production [37], experienced-based design (EBD)[33,42], experienced-based co-design (EBCD) [34-37] and accelerated experienced-based co-design (AEBCD) [32,41]. EBD or variants of this approach was reported in eight of eleven included studies, with a further study [31] testing an intervention previously designed using EBCD [40]. Projects ran for 3 months to over one year. Whilst local tailoring of approaches was clear, the use of a staged process for co-production was evident across all studies. This indicated that whilst the classification of approaches varied, the majority demonstrated patient, carer and staff reflecting and working together using participative methods in joint review and co-design activities focused on improving patient and/or carer experiences.

There was variation in levels of participant engagement. In an oncology service, whilst patients and a wide range of staff were included in the creative design project, they participated separately in the three stages of the study and did not come together to share and discuss their respective experiences [39]. In the Thomson et al study a designer led, three-stage process was used in which carers and staff came together only once; this variation in participant engagement did not appear to have any impact of the range of practical service improvement outcomes [38]. However in the case of the Golden et al study [39] the lack of joint working between the patients, carers and staff may

For peer review only - http://bmjopen.bmj.com/site/about/guidelines.xhtml

have been a key reason why no specific outcomes or agreed plans to introduce the changes identified in the creative design process were reported.

Reported outcomes of co-produced interventions

Our primary aim was to identify and evaluate the reported outcomes of co-produced interventions designed to achieve patient-focused quality improvements in acute healthcare settings. The methods used to understand participants' experiences and obtain their views included observation, process mapping, interviews, focus groups and postal survey, more detail is reported in supplementary file 2. Overall, patients reported positive experiences of participation, consistent with those associated with a wide range of co-production in the public and healthcare sector [5,7,10,12, 16,]. Co-production or co-design projects were effective in generating a wide range of ideas and specific suggestions related to improving patients' experiences across the different settings. Reported service changes and quality improvements are summarised in table 3. However, clarity regarding implementation of improvements to services and/or evidence of resulting impact on patient outcomes was one of the main limitations of the reporting in the majority of the studies.

The feasibility RCT [31]evaluating the impact of a co-designed intervention to improve the knowledge, experience and emotional well-being of carers of people with breast, lung or colorectal cancer was the only study reporting use of a validated outcome measure, the GHQ-12 [31]. The other quantitative measures used in this study were adapted from existing measures (n=2, for example, supportive care needs) or developed by the study team (n=2, for example, experience of care and knowledge of chemotherapy). Carers who received the intervention reported significantly better understanding of symptoms and side effects and that their information needs were more often met compared with the control group [31]. Confidence in coping improved between baseline and follow-up for the intervention group and declined for the control (although differences were not statistically significant). Accepting the limitations of the single centre and small sample size, this study provided evidence for the effectiveness of the carer focused intervention developed in an earlier study by carers and staff using EBCD methods [40]. In the remaining 10 studies there were no reported instances of use of validated outcome measures, such as quality of life measures, nor any instances of validated patient reported experience (PREM) or outcome measures (PROM).

Four studies [33, 35, 37, 39], did not clearly differentiate between the findings from the co-production or co-design processes used and patient focused quality improvement outcomes. In these studies it was difficult to distinguish between ideas for improvement generated by co-design activities and tangible service improvements that had either been introduced or were planned. However, the reported 'outcomes' of the studies can be categorised in three main ways and are summarised in table 3 below:

1) patient and staff involvement in the co-production or co-design processes;

2) generating ideas and suggestions for changes to processes, practices and clinical environments impacting on patients' and/or carers' experiences of a service, (and often indirectly on staffs');

3) tangible change in services and impact on patient or carer experiences.

Elements of co-production that led to quality improvement

Not all authors commented directly on the process of co-production. Those that did identified that: co-production methods provided the means to ensure patients' experiences, concerns and ideas for change were captured, presented to and discussed with staff in services, and that active engagement of patients in this way gave both legitimacy and urgency to service improvement plans [36,37], particularly where managers were involved in or actively supported co-production projects

For peer review only - http://bmjopen.bmj.com/site/about/guidelines.xhtml

[35,37,41,42]. Tsianakas et al [36] identified genuine and direct patient and carer involvement (relative to other service improvement projects in which patients and staff had participated) and linked this to patient participants taking direct responsibility for the work and its outcomes as being success factors linked to the EBCD process itself. Similar claims were evident in other EBD and EBCD projects [32, 33,37, 42], where patients perceived there to be a partnership with staff in the co-production process[32,33] and where staff reported facilitated co-design groups directly contributed to active and empowered partnerships between staff and patients in which provided genuine opportunities for patients to 'have their say'[32,33,37]. There appeared to be an equally powerful impact on some staff participating in co-production in that the methods required them to engage with and listen to patients in ways most had not experienced previously [34,36,37,42].

However, despite clear evidence of the contribution of co-design activities in generating ideas for patient focused service improvements (see table 3), in some projects staff and patients were frustrated by the lack of progress from problem and solution identification to actual quality improvements [33-35, 37]. In one case, the funded project appeared to end before concrete action planning to effect change, based on the ideas generated, had occurred [39]. Researchers ascribed this to a combination of the time taken to undertake co-design activities and the often slow processes of change in complex health service organisations.

Economic evaluation

There were no cost-benefit analyses related to reported outcomes for any of the studies. Only one study reported a cost analysis of co-production methods by comparing the cost of an accelerated EBCD approach (AEBCD) to standard EBCD [32]. A significant reduction in costs was reported for AEBCD. The cost of developing patient experience (trigger) films fell from £30,485 to £8,289 and, as the film developed could be used in future projects, this was a one-off cost. This study was also the only one to quantify costs of the co-design stages which were £20,276; over half of this sum was for the salary of local facilitators.

Other authors alluded to the costs associated with participatory or co-design methods in terms of the largely time-related costs involved in freeing staff to take part in or run the projects, or costs associated with actual or planned service improvements. These include, for example, improved signage [33], provision of personal care equipment [34], information booklets/leaflets, DVDs [32,36,37,38,42], a website and web based toolkit [42]or costs associated with having blood tests conducted off site [39]. However, no actual or estimated costs were reported for these elements. Similarly, nine of the eleven publications reported on researcher led, designer led or researcher supported projects but in only two cases were actual research costs traceable [32, 39].

Barriers and facilitators to implementation

The eleven studies encompassed nine projects conducted in diverse acute healthcare settings, although few of the reported barriers or facilitators were specific to project or setting. The most commonly reported barriers encountered in using co-production approaches in acute healthcare settings included a lack of support, resources or managerial authority to bring about structural or environmental changes [33,34,35,37,42]. In addition, practical or logistical problems were identified, for example, ensuring frail elderly people could attend regular co-design meetings [33]. Recruiting patients and carers and retaining them through the different stages of projects were highlighted by a number of researchers [32, 34-35, 37, 41]. In at least one project [34] some patients participated only in discovery or exploratory phases, then having 'shared' their experiences with staff, declined further participation arguing that they were no longer using the service. Researchers also identified the need to plan for and manage patients' understanding of what may be a radically different form of engagement with hospital staff, often quite unlike that experienced previously by users of health services[32,33,36]. In the studies reviewed, the frequency and duration of their involvement and

For peer review only - http://bmjopen.bmj.com/site/about/guidelines.xhtml

also the time it may take to bring about changes in the structures, processes and sometimes the physical environments of services were highlighted as factors to be addressed with participants [33,39,42].

In the majority of projects staff engagement was in addition to usual clinical or managerial roles; nonetheless, a high level of interest in and commitment to co-production activity was identified in almost all projects. However, this was impacted on in at least five projects by staffs' frustration at the expectation that they would undertake co-production/co-design work in their own time, that they could not allocate time out of their routine work and that additional support was often not provided by more senior staff [33-35,37, 42]. For some staff this made participation almost impossible [37]; for others it meant projects did not progress as expected [35] or contributed to tensions in co-design groups or between researchers and participants [33]. The duration of projects also increased the likelihood that staff turnover would impact on project leadership or involvement [34-35]. The need for structured and on-going managerial and organisational support was highlighted [32-34, 36, 38, 41,42] but only two studies expressly refer to governance or oversight groups set up to support co-design projects [33-34].

In projects where facilitators were engaged formally and funded to manage or oversee projects, it was more likely that projects (a) maintained momentum and were delivered as planned, (b) engaged and retained patients, carers and staff and (c) generated concrete examples of areas where patients' or carers' experiences could be improved [32,35-36,41]. In some studies, researchers (some of whom were designers) facilitated staff and patient engagement in the projects. Where designers were directly involved, our review suggests that they introduced ways of thinking and working which successfully challenged staff and patients to reconceptualise everyday processes and activities. This was achieved using metaphor games, design experiments, visual storyboards, prototyping, future focus groups, and emotional mapping; approaches not familiar to most health service staff [32-33, 38, 39]. However, with the exception of Locock et al [32] the costs associated with facilitator or researcher involvement were not identified.

Despite the barriers identified above, the studies suggest researchers and participants across settings viewed the benefits of this level of patient and staff involvement in structured co-production/co-design projects as outweighing the challenges.

Sustainability

None of the eleven studies formally evaluated whether co-production or co-design as a way of working had been sustained, or whether improvements made as a result of such approaches were sustained over any length of time. However, a number of concrete changes in service organisation, care environments and patient and in carer experiences occurring either during or closely related to co-production activities were reported, see table 3 [32,33-38,42]. Bowen et al [33] indicate that some changes may take two years or more to effect (for example, changes in hospital signage and routing), a period of time long after staff and patients had ceased to be actively involved in specific projects; another study reported that planned changes may not be achieved at all despite extending the life of projects [35].

[**Insert table 3 here**]

DISCUSSION

This rapid evidence synthesis has summarised peer reviewed evidence relating to the use of co-production approaches in focused quality improvement projects in the acute healthcare sector. The exclusion of broad search terms relating to patient participation, service improvement and clinical microsystems is a limitation of this rapid evidence synthesis. We searched for evidence in a database

For peer review only - http://bmjopen.bmj.com/site/about/guidelines.xhtml

of grey literature from Europe (Open-Grey) but we note that many reports of co-production/co-design projects undertaken as local quality improvement work are reported in the wider grey literature and were not considered in this review. However,, the evidence synthesis drew on good practice guidelines for such reviews and was rigorously conducted [23, 25-26]. This study is the first to systematically review outcomes associated with developing and implementing co-produced interventions in acute healthcare settings.

Different forms of co-production/co-design were most commonly reported and few criticisms of these methods were identified, in part reflecting an almost certain positive publication bias. However, critical reflections are emerging [37, 43,44]. These, in part explore the so-called 'dark side' of co-production recognising, for example: the potential for reproducing inequality as those who are most able to are those that co-produce; the risks of 'captured' co-production where participants are compelled, sometimes unknowingly, to co-produce; co-production as a substitution of labour (what Fotaki terms 'a race to the bottom in times of austerity' [43]); and co-production being appropriated as cover for inherently political decisions.

The kind of patient focused quality improvements reported in the identified studies can largely be considered examples of simple practical changes or 'sweating the small things' [45]. However, from the perspective of patients' experiences of and journeys through a service, these issues were likely to make care and treatment more humane and more person centred, as well as making journeys easier to engage with and negotiate. However, both Bowen et al [33] and Thomson et al [38] argue that the ideation tools in EBD and EBCD were limited and that incorporating 'designerly' thinking into co-production projects could stimulate greater creativity and alternative solutions in sometimes rigid health services.

The relative lack of robust evaluations we found may also reflect the stage of adoption of these types of methods in acute healthcare settings as we report, there is currently only one published feasibility RCT. However, there is a large-scale stepped wedge randomised controlled trial under way in nine Australian community mental health teams which is evaluating the effect of co-design on psychosocial recovery outcomes (such as willingness to ask for help and personal confidence and hope) [46]. In future studies, robust and routine evaluation of the costs and impacts of using co-production methods are required so that those leading and participating in quality improvement efforts in acute healthcare organizations can make informed decisions regarding their adoption in addition to (or instead of) other quality improvement methods. However, reliance on relatively broad and insensitive measures - such as patient satisfaction surveys- may overlook the real value placed by patients and staff on changes in the personal behaviour, attitudes, and culture of healthcare teams. The studies reviewed here consistently referred to these service level benefits of co-production for participants and services, as well as pointing to potential service system level effects such as those identified by Osborne et al [8].

As others have highlighted, the use of similar approaches in the community health care sector have not always 'follow[ed] through to significant and sustainable redesign' [5]. A recent narrative review of four different models of co-creation relevant to community-based health services proposed three 'key success principles', namely (1) a systems perspective (assuming emergence, local adaptation, and nonlinearity); (2) the framing of research as a creative enterprise with human experience at its core; and (3) an emphasis on process (the framing of the programme, the nature of relationships, and governance and facilitation arrangements, especially the style of leadership and how conflict is managed). The review authors proposed that co-creation 'failures' could often be tracked back to abandoning (or never adopting) these principles [5]. The publications in our RES do not specifically identify these principles and provide no clear evidence that such principles underpinned the planned research and service improvements noted. However, individually and collectively, included studies highlighted problems which arose when explicit adoption of such principles was not evident in the set up and delivery of the reported co-production projects in acute healthcare settings. Although the acute healthcare sector faces challenges associated with rapid patient turnover and with patients

For peer review only - http://bmjopen.bmj.com/site/about/guidelines.xhtml

sometimes critically or terminally ill, the majority of studies in this RES demonstrate that co-production is possible and was valued by participants. These findings have helped in planning for the introduction of the EBCD approach in the CREATE study. Although none of the studies reviewed were conducted in stroke services, the facilitators and barriers identified in the RES, together with the principles identified in the Greenhalgh et al review [5] indicate key areas for research or service improvement teams to address with managers, staff, patients and relatives participating in the EBCD project activity. Specifically, future studies should evaluate clinically related outcomes, for example through validated patient reported experience and outcome measures. Cost-effectiveness of co-production projects can and should be evaluated using standard health economic approaches. Equally important is the impact of service change on factors such as patient access to and flow through services and evaluating whether changes are sustained overtime. Potentially broader impacts on the values and behaviours of participants, including staff, are more difficult to measure but could be evaluated qualitatively and quantitatively and incorporated in the evaluation of co-production projects in acute healthcare settings.

Whilst, the potential benefits of using co-production in acute healthcare settings have been identified these must be set against the concerns identified by participants in the studies we reviewed that such approaches can be challenging to set up and implement in busy clinical environments where no formal, practical and financial provision is made for staff, patient and carer involvement on a sustained basis. Accelerated approaches may offer a partial solution but organisation level understanding of and commitment to these kinds of service improvement methods - as also identified by Greenhalgh et al [5] - remains key to their likely effectiveness.

CONCLUSIONS

We have identified a lack of rigorous evaluation of effectiveness and cost effectiveness of co-produced interventions in the acute healthcare sector at both the service and system level. Traditionally, co-production has been applied in community settings but there is growing recognition of the value of a more collective contribution to quality improvement work from those who are both delivering and receiving any form of public service [12,47]. At the clinical microsystem level within the healthcare sector there has been increasing adoption of co-production involving patients and staff, typically drawing on co-design approaches, as a means of improving the quality of frontline services [12-13]. Health services, whether public or private, operate within increasingly severe financial restraints where funding for quality improvement work is limited. Without robust critique, evaluation and evidence of the co-creation of value there is a danger of co-production becoming another management fad or fashion [48], with the meaning of the term itself appropriated and co-opted but no longer 'co-produced'.

Acknowledgements:

This project is funded by the National Institute for Health Research Health Services and Delivery Research Programme (project number 13/114/95). The views and opinions expressed therein are those of the authors and do not necessarily reflect those of the Health Services and Delivery Research Programme, NIHR, NHS or the Department of Health.

The arguments presented in this paper were partly informed by discussions during a workshop at the University of Nottingham on 7-8 July 2016 organised by Professor Andrew Gray and Professor Bruce Stafford: 'The Individual and the State: co-production and interdependency in the mixed economy of public service consumption'.

Our thanks go to Deirdre Andre and Sally Dalton, Information Specialists at the University of Leeds who helped refine the search terms and strategy and conducted the database searches. Tino Kulnic assisted with the initial screening of abstracts. Stephanie Honey and Parminder Dhiman, research fellows, also assisted with some of the data extraction.

For peer review only - http://bmjopen.bmj.com/site/about/guidelines.xhtml

Declaration of competing interests:

We have read and understood BMJ policy on declaration of interests and declare that we have no competing interests.

Author contributions:

DJC, FJ, RH and GR designed the rapid evidence synthesis and developed the search terms. GR conducted hand searches of the journals: CoDesign, The Design Journal, and Design Issues. DJC and FJ screened all abstracts and agreed on full text for review. DJC, RH and FJ reviewed all full texts and agreed inclusions and exclusions. DJC, FJ and RH independently completed data extraction and quality assessment for all included papers. DJC prepared the manuscript and GH, RH and FJ reviewed and revised the manuscript. All authors agreed the final version of the manuscript.

References

1. Realpe A and Wallace LM. What is co-production? London; The Health Foundation, 2016 http://personcentredcare.health.org.uk/sites/default/files/resources/what_is_co-production.pdf, accessed 19th August 2016
2. Boyle D, Clarke S and Burns S. Aspects of co-production: the implications for work, health and volunteering. London: New Economics Foundation 2006.
3. Needham C and Carr S (2009). SCIE Research briefing 31: co-production: an emerging evidence base for adult social care transformation. London: Social Care Institute for Excellence. http://www.scie.org.uk/publications/briefings/briefing31/ Accessed 22nd September 2016
4. Boyle D, Clarke S and Burns S (2006). Hidden work: co-production by people outside paid employment. London: Joseph Rowntree Foundation 2006.
5. Greenhalgh T, Jackson C, Shaw S. et al Achieving Research Impact Through Co-creation in Community-Based Health Services: Literature Review and Case Study. *The Milbank Quarterly*, 2016 94: 392–429.
6. Donetto S, Pierri P, Tsianakas V et al . 'Experience-based Co-design and healthcare improvement: realising participatory design in the public sector', *The Design Journal*, 2015 18(2): 227-248
7. Bovaird T (2007). 'Beyond engagement and participation: user and community co-production of public services'. *Public Administration Review*, 2007; 67: 846–860
8. Osborne SP, Radnor Z and Strokosch K Co-production and the co-creation of value in public services: a suitable case for treatment? *Public Management Review*, 2016 18(5): 639-653
9. Boyle D, Coote A, Sherwood S et al Right here, right now. Taking co-production into the mainstream. Discussion Paper. London, NEF/NESTA 2010.
10. Scottish Co-production Network. Co-production – how we make a difference together, http://www.coproductionscotland.org.uk/resources/, accessed 19th August 2016
11. Fugini M, Bracci E and Sicilia M. Co-production in the Public Sector. Experiences and Challenges. London: Springer 2016.
12. Batalden M, Batalden P, Margolis P et al. Co-production of healthcare service. *BMJ Qual Saf* BMJ Qual Saf 2016;25:509-517.
13. Nelson, E.C. Batalden,P. B., Godfrey,M.M. Lazar,J.S. (Eds) Value by Design: Developing Clinical Microsystems to Achieve Organizational Excellence, 2011. New-Jersey, Jossey-Bass

For peer review only - http://bmjopen.bmj.com/site/about/guidelines.xhtml

14. Lwembe S, Green SA, Chigwende J, Ojwang T and Dennis R. (2017) Co-production as an approach to developing stakeholder partnerships to reduce mental health inequalities: an evaluation of a pilot service. *Primary Health Care Research & Development* **18**: 14–23, doi:10.1017/S1463423616000141

15. Berwick DM. Era 3 for Medicine and Health Care. *JAMA*. 2016;315(13):1329-1330. doi:10.1001/jama.2016.1509

16. Voorberg, W.H. Bekkers, V. J. J. M. Tummers, L. G. (2014): A Systematic Review of Co-Creation and Co-Production: Embarking on the social innovation journey, *Public Management Review*, 2015; 17(9): 13335-1357

17. Sangiorgi, D. (2011). 'Transformative services and transformation design'. *Int Journal of Design*, 2011; 5: 29–40.

18. Sangiorgi D and Prendiville A (eds.). (In press) Designing for Service: Key issues and new directions. Bloomsbury Publishing; London

19. Bate SP and Robert G. Bringing user experience to health care improvement: the concepts, methods and practices of experience-based design. Oxford; Radcliffe Publishing 2007

20. Bate SP and Robert G. 'Experience-based design: from redesigning the system around the patient to co-designing services with the patient', *Qual & Safin Health Care*, 2006; 15(5): 307-310

21. Robert G, Cornwell J, Locock L, et al Patients and staff as co-designers of healthcare services. *BMJ* 2015; 350:g7714

22. Jones F, Clarke D, Robert G et al. Using co-production to improve patient carer and staff experiences in health care organizations: a multi-centre, mixed methods evaluation in inpatient stroke units. NIHR- HS&DR Project 2016 13/114/95, http://www.nets.nihr.ac.uk/__data/assets/pdf_file/0010/162469/PRO-13-114-95.pdf

23. Featherstone RM, Dryden, D.M., Foisy, M. et al. Advancing knowledge of rapid reviews: an analysis of results, conclusions and recommendations from published review articles examining rapid reviews. *Syst Rev* 2015 4(1):50. DOI 10.1186/s13643-015-0040-4

24. Khangura, S., Konnyu, K., Cushman, R., et al. Evidence summaries: the evolution of a rapid review approach. *Syst Rev* 2012; 1(1), DOI:10.1186/2046-4053-1-10

25. Ganann, R., Ciliska, D., & Thomas, H. Expediting systematic reviews: methods and implications of rapid reviews. *Implementation Science*, 2010 5: 56. DOI: 10.1186/1748-5908-5-56

26. Bevan, N. Connelly,N., Francis,J., et al Rapid Evidence Assessment Toolkit. Civil Service. http://webarchive.nationalarchives.gov.uk/20140509095856/http:/www.civilservice.gov.uk/networks/gsr/resources-and-guidance/rapid-evidence-assessment 2010 Accessed 5th February 2016

27. Peterson, K., Floyd, N., Ferguson, L., Christensen, V., & Helfand, M. (2016). User survey finds rapid evidence reviews increased uptake of evidence by Veterans Health Administration leadership to inform fast-paced health-system decision-making. Systematic Reviews, 5(1), 132.

28. NICE Methods for the development of NICE public health guidance (3rd edition). https://www.nice.org.uk/article/pmg4/chapter/1%20introduction 2010 Accessed 5th February 2016

29. Sandelowski M, Voils CI, Barrosso J Defining and Designing Mixed Research Synthesis Studies. *Research in the Schools* 2006 13 (1): 29

30. Boger E, Ellis J, Latter S, et al. (2015) Self-Management and Self-Management Support Outcomes: A Systematic Review and Mixed Research Synthesis of Stakeholder Views. *PLoS ONE* 2015 10(7): e0130990.

For peer review only - http://bmjopen.bmj.com/site/about/guidelines.xhtml

31. Tsianakas V, Robert G, Richardson A, et al. (2015) 'Enhancing the experience of carers in the chemotherapy outpatient setting: an exploratory randomised controlled trial to test the impact, acceptability and feasibility of a complex intervention co-designed by carers and staff', *Supportive Care in Cancer* 2015 23(10):3069-80

32. Locock, L Robert G, Boaz A, et al, Using a national archive of patient experience narratives to promote local patient-centered quality improvement: an ethnographic process evaluation of 'accelerated' experience-based co-design. *Journal of Health Services Research &Policy* 2014, 19(4) 200–207

33. Bowen, S. McSeveny, K, Lockley, E et al, How was it for you? Experiences of participatory design in the UK health service. *CoDesign* 2013 9:4, 230-246

34. Piper, D. Ledema R, Gray J, et al, Utilizing experience-based co-design to improve the experience of patients accessing emergency departments in New South Wales public hospitals: an evaluation study. *Health Services Management Research* 2012;25: 162 – 172

35. Larkin, M Boden, ZVR, Newton, E et al, On the Brink of Genuinely Collaborative Care: Experience-Based Co-Design in Mental Health. *Qualitative Health Research* 2015, 25(11) 1463–1476

36. Tsianakas,V Robert G, Maben J, et al, Implementing patient-centred cancer care: using experience-based co-design to improve patient experience in breast and lung cancer services. *Supportive Care in Cancer* 2012 20:2639–2647

37. Vennik,D van de Bovenkamp, H.M. Putters, K et al, Co-production in healthcare: rhetoric and practice. *Int Rev Administrative Sciences* 2016; 82(1) 150–168

38. Thomson A, Rivas C, Giovannoni G. Improving the quality of participant interaction and ideation tools within service improvement activities. *BMC Health Services Res*, 15:105

39. Golden.B.R. Hannam, R., Fraser H. et al, Improving the patient experience through design. Healthcare Quarterly 2011. 14(3)32-41

40. Ream E, Tsianakas V, Verity R et al Enhancing the role of carers in the outpatient chemotherapy setting: a participatory action research project. Final report. London; King's College London 2013 http://www.dimblebycancercare.org/wp-content/uploads/2013/09/Dimbleby-report-executive-summary-pdf-3rd-Sept.pdf, accessed 20th August 2016

41. Tollyfield, R. Facilitating an accelerated experience-based co-design project. *Brit Journal of Nurs*, 2014, 23, (3):136-141

42. Boyd. H. McKernon, S, Mullin, B et al, Improving healthcare through the use of co-design. The *New Zealand Medical Journal* 2011 125,76–87

43. Fotaki, M. Co-Production under the financial crisis and austerity. A means of democratizing public services or a race to the bottom? *Journal of Management Inquiry* 2015: 24(4): 433-438.

44. Meijer A. Coproduction as a structural transformation of the public sector. *Int Journal of Public Sector Management* 2016 29(6): 596-611

45. Moore C and Buchanan D. Sweat the small stuff: a case study of small-scale change processes and consequences in acute care. *Health Serv Manage Res* 2013; 26: 9–9.

46. Palmer V, Chondros P, Piper D, et al. The CORE Study protocol: a stepped wedge cluster randomized controlled trial to test a co-design technique to optimize psychosocial recovery outcomes for people affected by mental illness. *BMJ Open* 2015 5:e006688 DOI:10.1136

47. Robert G and MacDonald A. (2017) 'Infrastructuring for organisational creativity and quality improvement in the healthcare sector: 'designerly' or 'design-like?'. In: D Sangiorgi and A

For peer review only - http://bmjopen.bmj.com/site/about/guidelines.xhtml

Prendiville (eds). Designing for Service. Contemporary Issues and Novel Spaces, London: Bloomsbury

48. Walshe K. Pseudoinnovation: the development and spread of healthcare quality improvement methodologies, *International Journal for Quality in Health Care* 2009, 21 (3) 153-159

For peer review only

For peer review only - http://bmjopen.bmj.com/site/about/guidelines.xhtml

Table 3: Reported outcomes of co-production in acute healthcare settings

Type of outcomes	Evidence synthesis	Studies	Example
**Patient & staff involvement**	Patients perceived being really listened to by staff, having the sense that involvement was genuinely collaborative and that staff were committed to working with patients to improve services.	32,33, 34,36,41	Key to success was thought to be the strong relationship between patients and staff that has been built over time. (36)<br>Co-production' approach was seen to give patients, carers and staff a deeper understanding of each others' experience of the processes of care. It provided an opportunity to work meaningfully together, and this strengthened relationships among those involved in EBCD (34)<br>*'It was good, it felt as though [the hospital] and the professional staff were really interested … There was a comradeship if that's the right word …' (Older Patient- 33p)*
	Patients and staff recognised levels of participation and collaboration were distinct from any quality improvement activity they had experienced previously.	33,34,36,37	EBD's techniques facilitated building trust and rapport (33)<br>EBCD was perceived as a significantly higher level of genuine direct patient and carer involvement (relative to other service improvement projects in which patients and staff had participated) - *'the constant feedback [being] really, really useful', and '[needing] the eyeballing of each other to make it work'.* (36)<br>The EBCD process was thought to have given patients a greater sense of direct responsibility for the work and its outcomes (36, 37) and led to an increased understanding of the 'other' perspective, resulting in a potentially broader cultural change in mind-sets and behaviour. Staff participating in the co-design groups reported a greater sense of empowerment to make changes to their service (36).<br>Allowed nurses to reconnect with their core values of caring and compassion; development of a sense of empathy with patients was also evident.
	Staff realised that they often really did not have a good understanding and insight into patients' experiences of the services they provided.	36,37, 41, 42	Letting their 'own' patients talk about their experiences in a common 'action' setting affects professional staff, as patient stories make visible what the impact of (already known) problems really is on patients. The active role of patients and the way they express their experiences creates a sense of urgency to act on the improvement issues raised. (37)<br>Staff identified that working with patients in this honest and open sharing process was moving and at times humbling (36).
	There were also negative perceptions reported by a small number of patients and staff. These related to questioning the value of the amount of staff and patient time invested, and to not being able to see or experience actual	33,34	*'[The project] Seemed to go on for a long time . . . 'and I don't think we were properly prepared for that . . . my initial impression was that it would be perhaps a couple of interviews, and a discussion [Older patient-33:p]*<br>Both patients and staff identified that some service changes did not happen e.g. 'not sure if it has had any effect' (patient), 'in some areas, the area has not been improved' (staff), 'there's a few changes … [but] … there was some really serious things … they were highlighted, there was no resolution' (staff) (33)<br>Authors noted that although consensus reached quite easily, staff groups had tendency to focus on more positive

For peer review only - http://bmjopen.bmj.com/site/about/guidelines.xhtml

	service changes.		aspects of care and recent developments/improvements whereas service users and carers were more openly dissatisfied(34).
	Negative staff experiences and perceptions were also evident; these all related to challenges in conducting co-production or co-design projects.	33,34, 35, 37,42	Staff (and patients) reported little or no change had occurred. *'there's got to be something at the end of it'* (Staff Member-33p).<br>Staff viewed EBCD as a burden, additional to an already busy schedule (34, 37).<br>Staff workloads increased where EBCD was additional to usual roles (33, 35, 37, 42).<br>Lack of time and high level organisational support for Steering Group to complete implementation plans (35).<br>Lack of organisational capacity to respond to 'bottom up' service development (35).<br>Fee for service systems do not directly reward (or encourage) EBCD/co-production projects (37).
**Generating ideas & suggestions for changes to processes, practices & clinical environments**	The number of ideas generated or issues highlighted ranged from 5-11 core issues, through 48 co-design activities to 400 ideas generated by staff in an oncology service project.		
	A significant number of factors affecting patient experience from across the range of acute healthcare settings related to the structure and process of care and service provision.<br>Changes spanned:<br>-information and communication processes,<br>-environmental and organisational changes (and to a lesser extent)<br>-staff training and development.	33,36,40,42, 37, 42, 35	New template for patient letters (33)<br>A patient journey map and accompanying journey guide was developed linked to a patient held record (42).<br>A clinic guide and clinic (terms) dictionary were developed for new patients (38).<br>Redesigned discharge summary, improved cross site information booklet for patients transferring to another hospital for surgery (32)<br>Patient focused visual journey guide (42),<br>Proposals for layout of roads surrounding the outpatient building, design proposals for new way-finding materials (signage and maps) (33).<br>Guidance on diagnosis procedures included in junior doctors' induction (36).<br>Informal staff training programme-'recent developments in' (38).<br>A co-design toolkit and website for other services in New Zealand(42).

For peer review only - http://bmjopen.bmj.com/site/about/guidelines.xhtml

	Acute hospital admission and care have impacts beyond individual patients; involvement of families and carers - evident in five of the studies - appears to have helped ensure that those involved in the patients journey were also able to comment on and shape that experience.	31, 35, 36,	Administrative staff receive customer care training watch the patient experiences) DVD (33).<br>Ensuring that when carers came to visit, they were greeted appropriately at the door of the unit (32)<br>Clinic structures changed to reduce waiting times (33).<br>Carers information leaflet increases knowledge and understanding of symptoms and side effects and carers confidence (28).
	The structured co-production and co-design methods employed empowered both patients and staff to collaboratively review services and to plan and undertake changes in their workplaces.	32, 33, 34,36, 38,42,	Despite concerns about activities being '*a bit daunting at first*', participants supported researchers' perceptions that sharing experiences via storytelling and emotional mapping helped to build empathy and cohesion in the project group (30).<br>The opportunity to hear directly from patients and carers reportedly had a transforming effect on some staff. *'Our data suggest face-to-face encounters with patients in co-design groups often had a profound effect on staffing making them think differently about their practice and reconnect with their core professional values, resulting in renewed motivation* (29p).
	Involving designers/design researchers in co-production projects added value though challenging staff and patients way of thinking about their healthcare journey and experiences and how these could be improved within existing service settings. However, as design led techniques may be unfamiliar to staff and patients these and their purpose need to be carefully explained if they are not be seen as time wasting or games.	33,38,39	Forum theatre training to improve staff awareness of 'customer care' (33).<br>Use of metaphor, analogy and prototyping techniques (travel documents linked to an ideal patient journey). The props and the analogy facilitated the co-design process by enabling participants to not just suggest solutions to problems but create and share more radical suggestions for change (such as initiating a Patient Advisory Group) (38).<br>Photojournalling and storyboarding (39)
**Tangible change in services & impact on patient or carer experiences**	Patient and carer experience: Overall patients reported positive experiences of participation. Despite the barriers identified participants across settings viewed the benefits of patient and staff involvement in structured co-production/co-design projects as outweighing the challenges.	31, 32,34,36,42	Carers who received the co-designed intervention reported significantly better understanding of symptoms and side effects and that their information needs were more often met than the control group. Confidence in coping improved between baseline and follow-up for the intervention group and declined for the control)(31)<br>Patients can opt to receive chemotherapy treatment information in a group with others having the same treatment (36).<br>More comfortable V shaped pillows for post-operative patients (32).<br>Changed process for porters to remove waste avoiding ICU rest times (32)

For peer review only - http://bmjopen.bmj.com/site/about/guidelines.xhtml

			Provision of free refreshments (patients and families) (34).<br>New design for mammography gown (42).
	Service changes (processes and environmental change): It was not always possible to distinguish changes that had been implemented from those which were planned. Reporting of service improvement outcomes per-se was a limitation of the included studies, but the projects reported largely indicated that the structured co-production and co-design methods employed empowered both patients and staff to collaboratively review services and to plan and undertake changes in their workplaces.	32,34,36,37,38,42	Introduction of mini-Schwartz rounds on ICU (32).<br>New private room identified for receiving support after diagnosis (32).<br>Installation of a new air-conditioning unit in the waiting room, installation of a blanket warming cupboard, installation of payphones in waiting areas (34)<br>Changes to structure of clinics to reduce waiting times (36).<br>Decoration of waiting rooms (37)<br>Volunteer in the outpatient clinic linked to a patient advisory group (38).<br>Psychosocial care offered proactively during consultations (one hospital) (37).<br>Patient held record to track appointments (42)

For peer review only - http://bmjopen.bmj.com/site/about/guidelines.xhtml

For peer review only

For peer review only

For peer review only - http://bmjopen.bmj.com/site/about/guidelines.xhtml

For peer review only

For peer review only - http://bmjopen.bmj.com/site/about/guidelines.xhtml

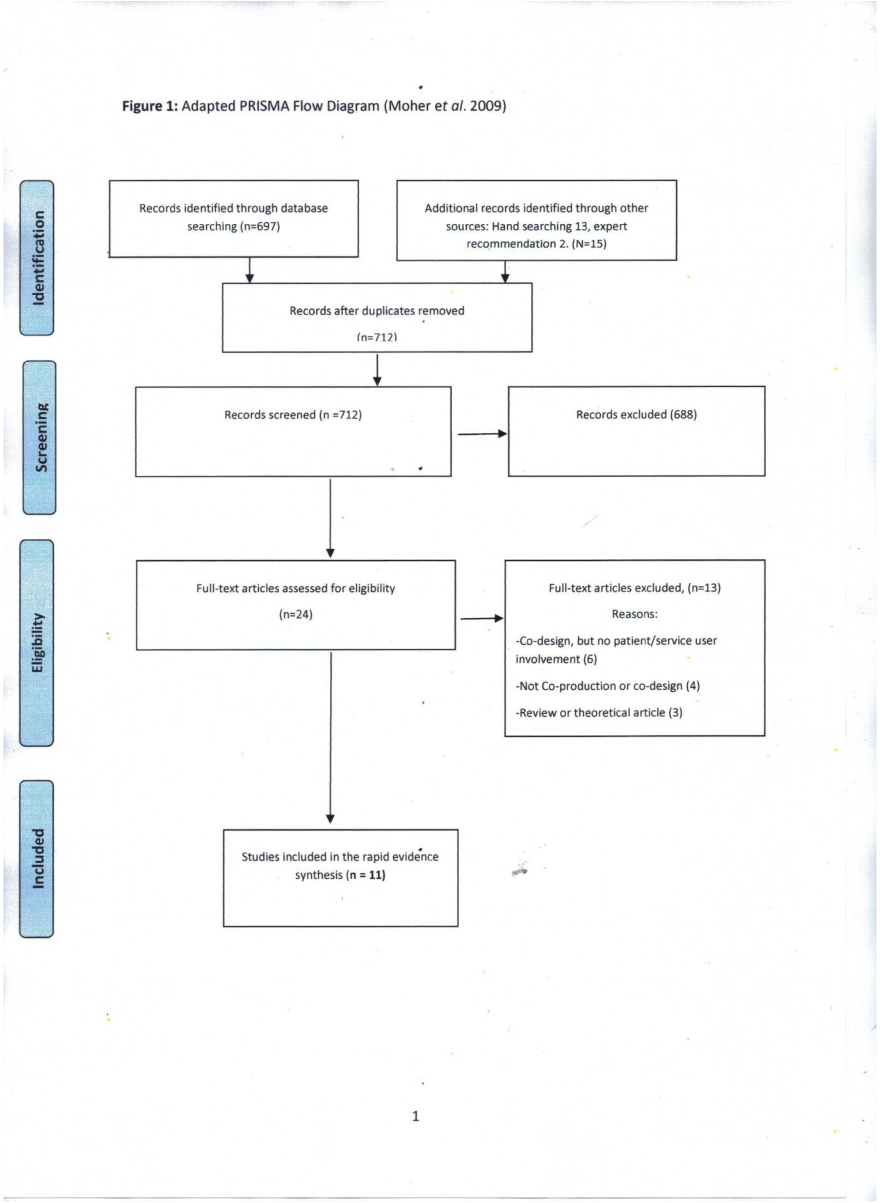

Figure 1 Adapted Prisma Flow Diagram

215x297mm (300 x 300 DPI)

CREATE Rapid evidence synthesis. Example of the quality appraisal tool used. The NICE (2010) guidance on use of the checklist can be accessed at: https://www.nice.org.uk/article/pmg4/chapter/1%20introduction

**Study identification:** Include author, title, reference, year of publication		
**Guidance topic:**	**Key research question/aim:**	
**Checklist completed by:**		
**Theoretical approach**		
**1. Is a qualitative approach appropriate?**<br>For example:<br>• Does the research question seek to understand processes or structures, or illuminate subjective experiences or meanings?<br>• Could a quantitative approach better have addressed the research question?	Appropriate<br>Inappropriate<br>Not sure	Comments:
**2. Is the study clear in what it seeks to do?**	Clear	Comments:

For peer review only

CREATE Rapid evidence synthesis. Example of the quality appraisal tool used. The NICE (2010) guidance on use of the checklist can be accessed at: https://www.nice.org.uk/article/pmg4/chapter/1%20introduction

For example:<br>• Is the purpose of the study discussed – aims/objectives/research question/s?<br>• Is there adequate/appropriate reference to the literature?<br>• Are underpinning values/assumptions/theory discussed?	Unclear<br>Mixed	
**Study design**		
**3. How defensible/rigorous is the research design/methodology?**<br>For example:<br>• Is the design appropriate to the research question?<br>• Is a rationale given for using a qualitative approach?<br>• Are there clear accounts of the rationale/justification for the sampling, data collection and data analysis	Defensible<br>Indefensible<br>Not sure	Comments:

For peer review only

CREATE Rapid evidence synthesis. Example of the quality appraisal tool used. The NICE (2010) guidance on use of the checklist can be accessed at: https://www.nice.org.uk/article/pmg4/chapter/1%20introduction

techniques used?<br>• Is the selection of cases/sampling strategy theoretically justified?		
**Data collection**		
**4. How well was the data collection carried out?**<br>For example:<br>• Are the data collection methods clearly described?<br>• Were the appropriate data collected to address the research question?<br>• Was the data collection and record keeping systematic?	Appropriately<br>Inappropriately<br>Not sure/inadequately reported	Comments:
**Trustworthiness**		
**5. Is the role of the researcher clearly described?**	Clearly described<br>Unclear	Comments:

For peer review only

CREATE Rapid evidence synthesis. Example of the quality appraisal tool used. The NICE (2010) guidance on use of the checklist can be accessed at: https://www.nice.org.uk/article/pmg4/chapter/1%20introduction

For example:<br>• Has the relationship between the researcher and the participants been adequately considered?<br>• Does the paper describe how the research was explained and presented to the participants?	Not described	
**6. Is the context clearly described?**<br>For example:<br>• Are the characteristics of the participants and settings clearly defined?<br>• Were observations made in a sufficient variety of circumstances<br>• Was context bias considered	Clear<br>Unclear<br>Not sure	Comments:
**7. Were the methods reliable?**<br>For example:<br>• Was data collected by more than 1 method?	Reliable<br>Unreliable<br>Not sure	Comments:

For peer review only

CREATE Rapid evidence synthesis. Example of the quality appraisal tool used. The NICE (2010) guidance on use of the checklist can be accessed at: https://www.nice.org.uk/article/pmg4/chapter/1%20introduction

• Is there justification for triangulation, or for not triangulating?<br><br>• Do the methods investigate what they claim to?		
**Analysis**		
**8. Is the data analysis sufficiently rigorous?**<br><br>For example:<br><br>• Is the procedure explicit – i.e. is it clear how the data was analysed to arrive at the results?<br><br>• How systematic is the analysis, is the procedure reliable/dependable?<br><br>• Is it clear how the themes and concepts were derived from the data?	Rigorous<br><br>Not rigorous<br><br>Not sure/not reported	Comments:
**9. Is the data 'rich'?**<br><br>For example:<br><br>• How well are the contexts of the data	Rich<br><br>Poor	Comments:

For peer review only

CREATE Rapid evidence synthesis. Example of the quality appraisal tool used. The NICE (2010) guidance on use of the checklist can be accessed at: https://www.nice.org.uk/article/pmg4/chapter/1%20introduction

described?<br>• Has the diversity of perspective and content been explored?<br>• How well has the detail and depth been demonstrated?<br>• Are responses compared and contrasted across groups/sites?	Not sure/not reported	
**10. Is the analysis reliable?**<br>For example:<br>• Did more than 1 researcher theme and code transcripts/data?<br>• If so, how were differences resolved?<br>• Did participants feed back on the transcripts/data if possible and relevant?<br>• Were negative/discrepant results addressed or ignored?	Reliable<br>Unreliable<br>Not sure/not reported	Comments:
**11. Are the findings convincing?**	Convincing	Comments:

For peer review only

CREATE Rapid evidence synthesis. Example of the quality appraisal tool used. The NICE (2010) guidance on use of the checklist can be accessed at: https://www.nice.org.uk/article/pmg4/chapter/1%20introduction

For example:<br>• Are the findings clearly presented?<br>• Are the findings internally coherent?<br>• Are extracts from the original data included?<br>• Are the data appropriately referenced?<br>• Is the reporting clear and coherent?	Not convincing<br>Not sure	
**12. Are the findings relevant to the aims of the study?**	Relevant<br>Irrelevant<br>Partially relevant	Comments:
**13. Conclusions**<br>For example:<br>• How clear are the links between data, interpretation and conclusions?<br>• Are the conclusions plausible and coherent?	Adequate<br>Inadequate<br>Not sure	Comments:

For peer review only

CREATE Rapid evidence synthesis. Example of the quality appraisal tool used. The NICE (2010) guidance on use of the checklist can be accessed at: https://www.nice.org.uk/article/pmg4/chapter/1%20introduction

• Have alternative explanations been explored and discounted?<br>• Does this enhance understanding of the research topic?<br>• Are the implications of the research clearly defined?<br>**Is there adequate discussion of any limitations encountered?**		
**Ethics**		
**14. How clear and coherent is the reporting of ethics?**<br>For example:<br>• Have ethical issues been taken into consideration?<br>• Are they adequately discussed e.g. do they address consent and anonymity?<br>• Have the consequences of the research been considered i.e. raising	Appropriate<br>Inappropriate<br>Not sure/not reported	Comments:

For peer review only

CREATE Rapid evidence synthesis. Example of the quality appraisal tool used. The NICE (2010) guidance on use of the checklist can be accessed at: https://www.nice.org.uk/article/pmg4/chapter/1%20introduction

expectations, changing behaviour?<br>• Was the study approved by an ethics committee?		
**Overall assessment**		
**As far as can be ascertained from the paper, how well was the study conducted? (see guidance notes)**	++<br>+<br>−	Comments:

For peer review only

Supplementary file 1 Search strategy (MEDLINE example)

Database: Ovid MEDLINE(R) <1996 to November Week 3 2015>

----

1 co-produc*.tw. (1149)

2 coproduc*.tw. (620)

3 co design*.tw. (53)

4 codesign*.tw. (16)

5 co creat*.tw. (171)

6 cocreat*.tw. (52)

7 (co research and design).tw. (1)

8 experience based design*.tw. (7)

9 participatory design*.tw. (149)

10 (experience adj3 design).tw. (252)

11 (evidence* adj2 design*).tw. (550)

12 EBC?.tw. (1604)

13 collaborative design.tw. (44)

14 human centred design.tw. (10)

15 human centered design.tw. (38)

16 people centred design.tw. (0)

17 people centered design.tw. (0)

18 inclusive design.tw. (32)

19 practice led design.tw. (1)

20 practice based design.tw. (2)

21 interactive design.tw. (31)

22 open design.tw. (147)

23 user centred design.tw. (51)

24 user centered design.tw. (177)

25 or/1-24 [co-design] (5093)

26 (acute adj (setting* or hospital* or care or healthcare)).tw. (15023)

27 ((secondary or speciali?ed) adj care).tw. (4410)

28 hospital care.tw. (4817)

29 exp Hospitals/ (117870)

30 Hospitalization/ (52132)

31 (hospitali?ation* or hospitali?ed).tw. (119506)

32 Inpatients/ (12602)

33 inpatient*.tw. (52413)

For peer review only

34 emergency medicine/ (8188)

35 Emergency treatment/ (8750)

36 emergency hospital admission*.tw. (240)

37 emergency hospitali#ation.tw. (116)

38 critical care/ (14911)

39 ambulatory care/ (20495)

40 urgent care.tw. (960)

41 or/26-40 [acute terms] (343100)

42 Delivery of Health Care/ (45257)

43 Efficiency, Organizational/ (17793)

44 Efficiency/ (4714)

45 Health Services Research/ (23584)

46 "Outcome Assessment (Health Care)"/ (50681)

47 patient outcome assessment/ (1713)

48 exp Program Evaluation/ (54529)

49 Quality Assurance, Health Care/ (38609)

50 Quality Improvement/ (9440)

51 Quality Indicators, Health Care/ (11744)

52 Quality of Health Care/ (43880)

53 (bench mark* or benchmark*).tw,kf. (17315)

54 (delivery adj2 health*).tw,kf. (12585)

55 (efficien* adj2 (assess* or assurance* or evaluat* or improv* or indicat* or measur* or test*)).tw,kf. (23714)

56 (performance adj2 (assess* or assurance* or evaluat* or improv* or indicat* or measur* or test*)).tw,kf. (74378)

57 (outcome* adj2 (assess* or assurance* or evaluat* or improv* or indicat* or measur* or test*)).tw,kf. (256084)

58 (program* adj2 (assess* or assurance* or evaluat* or improv* or indicat* or measur* or test*)).tw,kf. (27836)

59 or/42-58 [outcome or evaluation terms] (611767)

60 25 and 41 and 59 [co-design and acute care and outcome or evaluation terms] (45)

61 exp animals/ not humans.sh. (2031513)

62 60 not 61 [human only studies] (45)

63 limit 62 to (english language and yr="2005 - 2015") (40

For peer review only

Supplementary file 2 Empirical studies included in review

Publication/ country	Form of co-production	Setting	Methods	Reported Outcomes	Barriers and facilitators	Health economic evaluation	Quality rating
Bowen, S. et al, 2013 How was it for you? Experiences of participatory design in the UK health service. CoDesign, 9:4, 230-246<br><br>UK	Experience Based Design (EBD)	Medical outpatient service for older people. In a Teaching Hospital NHS Trust	Face to face (2) or telephone (9), semi-structured interviews (n= 11)	*Reported EBD Outcomes:*<br>• New template for patient appointment letters<br>• Design proposals for new way-finding materials (signage and maps)<br>• Proposal for layout of roads surrounding the outpatient building<br>• Video of patient stories distributed to all staff in the Trust to highlight older people's experiences,<br>• Forum theatre training event to improve outpatient staff awareness of customer care.<br>*Interview Findings*:<br>• Doing EBD (4 subthemes).<br>• Reflecting on EBD & Better Outpatient Services for Older People' (5 subthemes).	*Barriers:*<br>• Perception that participants were not doing the designing<br>• Perception that too many people were involved<br>• Ageing physical infrastructure Bureaucratic nature of trust procedures<br>• Low expectations of extent of change<br>• Availability of financial resources<br>• Staff workload<br>*Facilitators*:<br>• Sharing stories to gain perspectives of others<br>• Emotional mapping to highlight touchpoints (experiences and areas for improvement)<br>• Involvement in co-designing, being listened to by hospital staff	Not evident or reported.	++
Boyd. H. 2011 et al, Improving healthcare through the use of co-design. The New Zealand Medical Journal, 125,76–87	Experience Based Design (EBD) (Adapted)	Breast Services (inpatient & outpatient) in two hospitals in one District Health Board.	Patient journey mapping, experience surveys and co-design workshops.	• Patient journey map for staff review<br>• Patient Information folder to help navigate the service<br>• 7 new information leaflets,<br>• Patient held record to track appointments	*Barriers:*<br>• Staff workloads; EBD in addition to usual roles<br>• Financial restraints for hospitals and services<br>• Evaluation and planning for sustainability not factored into the	Not evident or reported.	+

For peer review only

Publication/ country	Form of co-production	Setting	Methods	Reported Outcomes	Barriers and facilitators	Health economic evaluation	Quality rating
New Zealand				• Patient focused visual patient journey guide<br>• Poster providing (better) communication guidance for staff and patients<br>• New design mammography gown,<br>• Co-design toolkit and website for other NZ services	EBD project<br>*Facilitators:*<br>• Engaging with patients at the outset of the co-design process before decisions are made<br>• Adopting a flexible approach to enable patients still receiving treatment to participate over time<br>• Working to get key staff buy in (clinical, managerial, administrative) & continued engagement<br>• Find & use ways to challenges participants' thinking, to work outside of their 'comfort zone'		
Golden.B.R. et al, 2011 Improving the patient experience through design. Healthcare Quarterly. 14(3)32-41<br><br>Canada	Rotman Creative Design Process<br>(co-design approach)	Cancer research and treatment centre.<br>Systemic therapy:<br>chemotherapy and transfusion services.	Ethnographic observations. Photojournalling & follow up interviews.<br>Staff discussion group: patient vignettes/ story-boarding ideal patient journey<br>Patient discussion group & storyboarding ideal patient journey.	• Prototypes and a jointly designed ideal patent journey	Not specifically reported.<br><br>Indicates staff workload and patient treatment scheduling pressures precluded bringing the two groups together to co-design service improvements.	Not evident or reported.<br><br>Project funded by charitable & corporate donations to the hospital & the University.	+
Larkin, M et al, 2015 On the Brink of Genuinely Collaborative Care: Experience-	Experience-based Co-Design<br>(EBCD)	Early Intervention Services and Inpatient units (n =7)	In depth interviews with service users (n=6), parents (n=6), inpatient staff (n=9).	Six priorities for service improvement identified from interviews and feedback group work:	*Facilitators:*<br>• Support from senior NHS staff<br>• Researchers leading the processes	Not evident or reported	+

For peer review only

Publication/ country	Form of co-production	Setting	Methods	Reported Outcomes	Barriers and facilitators	Health economic evaluation	Quality rating
Based Co-Design in Mental Health.Qualitative Health Research2015, Vol. 25(11) 1463–1476<br><br>UK	(adapted)	at two hospitals in the Midlands.<br><br>Mental health services for young people and families.	Feedback groups (20) involving 150 stakeholders.<br><br>Co-design event (50 participants).	• pathways in and out of hospital<br>• providing staff with a rewarding and well-supported role<br>• communicating with families and service-users<br>• recovery-focused practice<br>• creating a positive environment for everyone<br>• recognizing and sharing good practice across professions and services<br>• Co-design groups at the co-design event produced 13 action plans to address the 6 priorities	• Involvement of people internal and external to the service in the EBCD team aided project management<br>• Sensitivity to the needs and experiences of services uses in this setting in identifying touchpoints and developing trigger films to share with staff<br><br>*Barriers:*<br>• Challenging for some staff to hear strong service-users and carer views on services<br>• Lack of time and high level organisational support for Steering Group to complete implementation plans<br>• Steering group lacking the power to bring about change in organisations<br>• Organizational restructuring resulting in some study collaborators and "champions" leaving or changing role,<br>• Improvements requiring input from other departments or requiring strategic, budgetary, or staffing commitment<br>• Lack of organizational capacity to respond flexibly or directly to "bottom-up" service development		
Locock, L et al, (2014) Using a	Accelerated Experience-	In intensive care and	Process evaluation using ethnographic	*Re: AEBCD*	*Anticipated Barriers:*	In AEBCD the cost of	++

For peer review only

Publication/ country	Form of co-production	Setting	Methods	Reported Outcomes	Barriers and facilitators	Health economic evaluation	Quality rating
national archive of patient experience narratives to promote local patient-centered quality improvement: an ethnographic process evaluation of 'accelerated' experience-based co-design. Journal of Health Services Research &Policy2014, Vol. 19(4) 200–207<br><br>UK	based Co-Design (AEBCD)	lung cancer services in two English NHS hospitals	methods:<br>• observations of AEBCD processes and events (155 hours)<br>• interviews (n=30)<br>• repeated group interviews with patient participants<br>• reflective diaries (n=22)<br>• service improvement logs (n=4)<br>• evaluation questionnaires (post AEBCD events)	Trigger films developed through secondary analysis if existing films from the Oxford University collections of patient experience interviews were acceptable to patients and staff and were used successfully in subsequent co-design work.<br><br>*Re: Service improvements*<br>48 co-design activities across the four patient pathways. Examples reported were:<br>• Improved cross-site information booklet for patients transferring to another hospital for surgery<br>• A redesigned discharge summary with input from all professions<br>• More comfortable V-shaped pillows for postoperative patients<br>• Sourcing clocks to aid patient orientation in ICU<br>• Changed process for porters to remove waste avoiding ICU rest times<br>• New private room identified for receiving support after diagnosis<br>• Introducing mini 'Schwartz rounds' on ICU	Films developed from a national archive would be considered inappropriate or an inaccurate reflection of care in a specific hospital.<br><br>Barrier not evident: 85% of participants (patients and relatives) reported films to be good or excellent.<br><br>No other barriers reported<br><br>*Facilitators:*<br>• Regular face-to-face meetings with patients in co-design groups reminded staff of who change was for and why it mattered<br>• Co-design groups facilitated active and empowered partnerships between patient and staff	developing a trigger film was £8289, compared to £30,485 for EBCD.<br><br>Co-design phase costs, were £20,276<br>(over half of this was facilitator salary).	

Publication/ country	Form of co-production	Setting	Methods	Reported Outcomes	Barriers and facilitators	Health economic evaluation	Quality rating
Piper, D. et al, 2012 Utilizing experience-based co-design to improve the experience of patients accessing emergency departments in New South Wales public hospitals: an evaluation study. Health Services Management Research2012;25: 162 – 172<br><br>Australia	Experience-based Co-Design (EBCD)	Emergency Department (EDs) & related services in two (programme) stages in seven public hospitals in New South Wales, Australia.	Review of EBCD process and evaluation reports provided by participating sites.<br><br>Interviews of patients, clinicians and managers, and also of high-level project managers including executive sponsors, clinical leads, project managers across sites (n=117)	EBCD processes provided opportunities to work together meaningfully and enabled patients, carers and staff to develop a deeper understanding of each other's experience of care processes, and this strengthened relationships among those involved in EBCD.<br><br>Examples of co-designed strategies implemented and sustained at the programme sites included:<br>• Recruitment of a full-time cleaner<br>• Installation of a new air conditioning unit in the waiting room<br>• Installation of a blanket warming cupboard<br>• Installation of food vending machines in the waiting area<br>• Access to free refreshments<br>• Installation of pay phones in the waiting area<br>• Patient access to 'sick-bay' bags in the waiting area<br>• Increased security presence in the emergency room.<br>22 examples are provided in the	*Barriers:*<br>• Staff viewed EBCD as a burden additional to already busy schedule<br>• Limits in implementation officers' & clinical leads' EBCD roles, reportedly reduced impact of programmes & long-term change<br>• Consumer recruitment & retention was difficult due to the transitory nature of the care episode<br>*Facilitators:*<br>• Develop a consumer engagement strategy, using on-going communication including face-to-face bi-monthly re-connect meetings, telephone & email contact/updates<br>• Integrating the EBCD process into everyday work practices rather than presenting EBCD as a separate task<br>• Adequate resources & reporting opportunities to executive meetings<br>• Clear accountability for implementation, allow adequate time to embed solutions into daily practice and cultures of EDs<br>• Appointing a liaison person to: ensure regular participant contact; manage governance of project & processes; communicate & contextualize aims & timeframes of EBCD to staff; develop a communication strategy to inform all stakeholders of the status & impact	Not evident or reported.	+

For peer review only

Publication/ country	Form of co-production	Setting	Methods	Reported Outcomes	Barriers and facilitators	Health economic evaluation	Quality rating
				publication.<br><br>Implementation strategies also identified.	of solutions on an on-going basis		
Thomson,A., Rivas,C., and Giovannoni. G. Multiple sclerosis outpatient future groups: improving the quality of participant interaction and ideation tools within service improvement activities. BMC Health Services Research (2015) 15:105. DOI 10.1186/s12913-015-0773-8<br><br>UK	Participatory approach drawing on techniques from the speculative design field.	An outpatient clinic for patients with multiple sclerosis in London, UK.	3 staged process using focus groups (future groups). In stages 1 and 3 patient participants and staff participants worked separately. In stage 2 the two groups were combined.<br><br>Process completed in 3 months<br><br>Speculative design techniques included: directed patient and staff journey mapping; combining patient and staff maps of an ideal (patient) journey (using analogies and props-a set of travel related documents); developing service improvements (represented on researcher developed separate patient and	• Volunteer in the outpatient clinic, linked to a Patient Advisory Group meeting 3 monthly<br>• A guide to the MS clinic for new outpatients<br>• An MS clinic dictionary (covering medical terms related to MS and frequently used in the clinic)<br>• A walking map with distances to the clinic (from the underground or disabled parking areas or local coffee shops)<br>• Magazine replenishment (as a clinic resource)<br>• Informal staff training programme (developments in MS research and care)	Facilitators:<br>• Adding (speculative) design led techniques to a participatory approach broadly similar to the 6 stage EBCD approach<br>• Adapting the design led props for the specific healthcare context<br>• Involving the design researcher directly in the (future) focus groups<br>• Including senior staff/managers in the stage 3 (future) focus groups to facilitate understanding of proposed service developments and their implementation.<br>Barriers:<br>• Adapting design led methods for health services improvements projects may reduce their creative contribution if used in a procedural manner<br>• Participant engagement with design led metaphor and analogy driven techniques need to be considered carefully and reflect local and disease specific contexts	Not evident or reported.	+

For peer review only

Publication/ country	Form of co-production	Setting	Methods	Reported Outcomes	Barriers and facilitators	Health economic evaluation	Quality rating
			staff conference style posters (prototyping).				
Tollyfield, R. 2014 Facilitating an accelerated experience-based co-design project. British Journal of Nursing, 2014, I23, (3):136-141<br><br>UK	Accelerated Experience-based Co-Design (AEBCD)	Specialist cardiothoracic intensive therapy unit (ITU) in London.<br><br>(One of the four units involved in the Locock et al 2014 study)	Reflection on the role of facilitator in an AEBCD project in one of two ITUs participating in the project.	Reports on the experience of and learning from facilitation of the AEBCD study conducted by Locock et al (2014).<br><br>• Promoting staff engagement a key aspect of the project- via posters, emails, one-to-one discussions and a ITU weekly newsletter<br>• Developing solutions to low patient recruitment levels<br>• Providing facilitation of the main project events and co-design meetings<br>• Prompting and supporting implementation of planned change	*Facilitators:*<br>• Project supported and endorsed by senior managers and clinicians- Director of Nursing, critical care service manager, ITU consultant physician<br>• Formal training in EBCD methods and on-going email and telephone support from staff experienced in using EBCD<br>• Access to and use of the online Kings Fund EBCD toolkit<br>*Barriers:*<br>• facilitator role time consuming and impacted significantly on time for clinical work (externally funded role for experienced ITU clinician at this site)<br>• learning about the AEBCD process whilst undertaking project organisational tasks and activities against a defined timeline	Not reported.<br><br>See Locock et al (above) for costs of facilitator salaries.	+
Tsianakas,V et al, 2012 Implementing patient-centred cancer care: using	Experience-based Co-Design (EBCD)	A large, inner-city cancer centre in	Ethnographic observation (219 hours)<br>Filmed narrative	*16 breast cancer working group outcomes including:*	*Barriers:*<br><br>Not specifically identified in the	Not evident or reported	++

For peer review only - http://bmjopen.bmj.com/site/about/guidelines.xhtml

Publication/ country	Form of co-production	Setting	Methods	Reported Outcomes	Barriers and facilitators	Health economic evaluation	Quality rating
experience-based co-design to improve patient experience in breast and lung cancer services. Supportive Care in Cancer (2012) 20:2639–2647<br><br>UK		England.<br><br>(Breast and lung cancer patients)	patient interviews (n=36)<br><br>Staff interviews (n= 63)<br><br>Post project interviews with<br><br>Staff (n=4)<br><br>and patients (n=5)	• Administrative staff receive customer-care training and are shown patients' DVD<br>• Healthcare assistants' interpersonal skills assessed prior to recruitment<br>• Managers and administrative staff use values-based performance tool which can improve patients' experience<br>• Changes to structure of clinics to reduce waiting times<br>• Patients receiving same chemotherapy treatment given option to receive information in a group<br><br>*11 lung cancer working group outcomes including:*<br><br>• Establishment of second breaking-bad-news room<br>• Guidance on diagnosis procedures included in junior doctors' induction<br>• Promotion of information and support centres at different sites (advertising at hospital entrance)<br>• Patient DVD 'welcome to cancer services' for newly diagnosed and	publication.<br><br>• As EBCD does not explicitly seek to recruit a representative sample of patients but those who are motivated to 'tell their story' and participate in the co-design work. This may lead to the experiences of 'harder to reach' patient groups remaining unheard.<br><br>*Facilitators:*<br><br>• Genuine and direct patient and carer involvement (relative to other service improvement projects in which patients and staff had participated).<br>• Patient participants taking direct responsibility for the work and its outcomes<br>• The EBCD change process itself together with the direct and active participation of staff and patients produced implementation and action, and contributed to spread and sustainability		

For peer review only

Publication/ country	Form of co-production	Setting	Methods	Reported Outcomes	Barriers and facilitators	Health economic evaluation	Quality rating
				referred patients			
Tsianakas,V., Robert ,G., Richardson,A., Verity,R., Oakley,C., Murrells,T., Flynn,M., Ream,E. Enhancing the experience of carers in the chemotherapy outpatient setting: an exploratory randomised controlled trial to test impact, acceptability and feasibility of a complex intervention co-designed by carers and staff . Support Care Cancer. 2015. DOI 10.1007/s00520-015-2677-x<br><br>UK	The study reports an RCT to test the impact and feasibility of a complex intervention (Take Care). The intervention was developed previously in the same hospital using a co-design approach – Experience-Based Co-Design.	Chemo-therapy day unit (breast, lung or colorectal cancer) in a London teaching hospital.	A two phase mixed methods design including i) an exploratory RCT (impact-measured through before and after postal questionnaire completion), ii) focus groups with carers and healthcare professionals (feasibility and acceptability)	• Carers in the intervention group had better understanding of the symptoms and side effects compared with the control group.<br>• Carers in the intervention group had more confidence in coping than the control group.<br>• There were no differences between the 2 groups for emotional well-being<br>• Health care professionals and carers confirmed the feasibility and acceptability of the EBCD derived Take Care intervention<br>• Patients and HCPs reported the intervention was educational, enhanced knowledge of symptoms and side effects, and increased confidence of carers.	Not reported in terms of EBCD<br><br>Reported limitations of the study included:<br>• Small sample size, no power calculation<br>• Exploratory trial in a single centre limits generalisability<br>• Self-report questionnaire responses may be subject to recall bias	Not evident or reported	+
Vennik et al, Co-production in healthcare:	Experience-based Co-Design (EBCD)	One haematolog y and four	Semi-structured interviews (n=27)	Improvement areas identified by patients were similar across hospitals. Apart from some specific	Barriers:<br>• Individual level- some physicians	Not evident or reported	++

Publication/ country	Form of co-production	Setting	Methods	Reported Outcomes	Barriers and facilitators	Health economic evaluation	Quality rating
rhetoric and practice. International Review of Administrative Sciences<br>2016, Vol. 82(1) 150–168<br>Netherlands	in 4 centres (Adapted)<br>Four project teams planned to use EBCD methodology; all adapted the method. Two used focus groups to gather patient experiences instead of filmed interviews. Two did not interview professionals. One did not use co-design workgroups concluding that it would require too much from participants; staff were not immediately convinced of the added value of this	oncology departments in 5 Dutch hospitals	Observations (70 hours) Documentary analysis	experiences with medical treatments, they mostly concerned aspects of quality of care including:<br>• Decoration of waiting rooms,<br>• Waiting times, -Communication, and coordination with general practitioners.<br>• Psychosocial care is now proactively offered during consultations at one hospital,<br>• A dental care in oncology leaflet was introduced at another hospital<br>Improvement issues identified were related to individual professions, hospital wards, the hospital organization and other healthcare providers.	afraid of being criticised by patients,<br>• Organisational barriers-pressure of work and other project commitments, changes in staffing levels, service re-organisations during the 12 months projects<br>• EBCD largely seen as 'additional work'<br>• Local policies and national trends can impact on engagement with co-production, no staff time freed for co-production/co-design, staff expected to deliver treatment and care and then engage in service improvement in their own time.<br>• Fee-for-service systems do not directly reward projects like EBCD/co-production<br>Facilitators:<br>• Motivated people who are willing to work on EBCD/co-production in their own time<br>• Selecting the 'right patients', inviting patients who have a critical point of view, are mentally and physically well enough condition to participate, and able to tell their story.<br>• It is not so much what patients say that is important; it is the process and the way they are given the opportunity to have their say (in EBCD/co-production) The active role of patients creates a sense of urgency		

For peer review only

Publication/ country	Form of co-production	Setting	Methods	Reported Outcomes	Barriers and facilitators	Health economic evaluation	Quality rating
	phase				to act on the improvement issues raised<br>• The process of co-production/EBCD stimulated staffs' thinking about how to realize quality improvements<br>• Keeping projects 'small', not attempting to improve everything at once and focusing<br>• Patients' input can gain legitimacy for improvement plans and facilitate implementation		

For peer review only

PRISMA 2009 Checklist

Section/topic	#	Checklist item	Reported on page #
**TITLE**			
Title	1	Identify the report as a systematic review, meta-analysis, or both.	4
**ABSTRACT**			
Structured summary	2	Provide a structured summary including, as applicable: background; objectives; data sources; study eligibility criteria, participants, and interventions; study appraisal and synthesis methods; results; limitations; conclusions and implications of key findings; systematic review registration number.	2
**INTRODUCTION**			
Rationale	3	Describe the rationale for the review in the context of what is already known.	3-4
Objectives	4	Provide an explicit statement of questions being addressed with reference to participants, interventions, comparisons, outcomes, and study design (PICOS).	4
**METHODS**			
Protocol and registration	5	Indicate if a review protocol exists, if and where it can be accessed (e.g., Web address), and, if available, provide registration information including registration number.	N/A
Eligibility criteria	6	Specify study characteristics (e.g., PICOS, length of follow-up) and report characteristics (e.g., years considered, language, publication status) used as criteria for eligibility, giving rationale.	5-6
Information sources	7	Describe all information sources (e.g., databases with dates of coverage, contact with study authors to identify additional studies) in the search and date last searched.	5
Search	8	Present full electronic search strategy for at least one database, including any limits used, such that it could be repeated.	Supp file1
Study selection	9	State the process for selecting studies (i.e., screening, eligibility, included in systematic review, and, if applicable, included in the meta-analysis).	6
Data collection process	10	Describe method of data extraction from reports (e.g., piloted forms, independently, in duplicate) and any processes for obtaining and confirming data from investigators.	6
Data items	11	List and define all variables for which data were sought (e.g., PICOS, funding sources) and any assumptions and simplifications made.	5-6
Risk of bias in individual studies	12	Describe methods used for assessing risk of bias of individual studies (including specification of whether this was done at the study or outcome level), and how this information is to be used in any data synthesis.	N/A
Summary measures	13	State the principal summary measures (e.g., risk ratio, difference in means).	N/A
Synthesis of results	14	Describe the methods of handling data and combining results of studies, if done, including measures of consistency (e.g., I^2) for each meta-analysis.	6

For peer review only

PRISMA 2009 Checklist

Section/topic	#	Checklist item	Reported on page #
Risk of bias across studies	15	Specify any assessment of risk of bias that may affect the cumulative evidence (e.g., publication bias, selective reporting within studies).	N/A
Additional analyses	16	Describe methods of additional analyses (e.g., sensitivity or subgroup analyses, meta-regression), if done, indicating which were pre-specified.	N/A
**RESULTS**			
Study selection	17	Give numbers of studies screened, assessed for eligibility, and included in the review, with reasons for exclusions at each stage, ideally with a flow diagram.	6 and 7
Study characteristics	18	For each study, present characteristics for which data were extracted (e.g., study size, PICOS, follow-up period) and provide the citations.	Supp file 2
Risk of bias within studies	19	Present data on risk of bias of each study and, if available, any outcome level assessment (see item 12).	N/A
Results of individual studies	20	For all outcomes considered (benefits or harms), present, for each study: (a) simple summary data for each intervention group (b) effect estimates and confidence intervals, ideally with a forest plot.	Supp file 2
Synthesis of results	21	Present results of each meta-analysis done, including confidence intervals and measures of consistency.	N/A
Risk of bias across studies	22	Present results of any assessment of risk of bias across studies (see Item 15).	N/A
Additional analysis	23	Give results of additional analyses, if done (e.g., sensitivity or subgroup analyses, meta-regression [see Item 16]).	N/A
**DISCUSSION**			
Summary of evidence	24	Summarize the main findings including the strength of evidence for each main outcome; consider their relevance to key groups (e.g., healthcare providers, users, and policy makers).	7-10 and Table 3
Limitations	25	Discuss limitations at study and outcome level (e.g., risk of bias), and at review-level (e.g., incomplete retrieval of identified research, reporting bias).	10-11
Conclusions	26	Provide a general interpretation of the results in the context of other evidence, and implications for future research.	
**FUNDING**			
Funding	27	Describe sources of funding for the systematic review and other support (e.g., supply of data); role of funders for the systematic review.	12

From: Moher D, Liberati A, Tetzlaff J, Altman DG, The PRISMA Group (2009). Preferred Reporting Items for Systematic Reviews and Meta-Analyses: The PRISMA Statement. PLoS Med 6(6): e1000097. doi:10.1371/journal.pmed1000097

For more information, visit: **www.prisma-statement.org**.

For peer review only - http://bmjopen.bmj.com/site/about/guidelines.xhtml

BMJ Open

What outcomes are associated with developing and implementing co-produced interventions in acute healthcare settings? A rapid evidence synthesis.

Journal:	*BMJ Open*
Manuscript ID	bmjopen-2016-014650.R2
Article Type:	Research
Date Submitted by the Author:	04-Apr-2017
Complete List of Authors:	Clarke, David; University of Leeds, Academic Unit of Elderly Care and Rehabilitation<br>Jones, Fiona; St Georges University of London,<br>Harris, Ruth; King\'s College London,<br>Robert, Glenn; King's College London,
**Primary Subject Heading**:	Health services research
Secondary Subject Heading:	Evidence based practice, Qualitative research
Keywords:	rapid evidence synthesis, co-production, acute healthcare, systematic review

SCHOLARONE™ Manuscripts

For peer review only

What outcomes are associated with developing and implementing co-produced interventions in acute healthcare settings? A rapid evidence synthesis.

Corresponding author:
Dr David Clarke
Associate Professor in Stroke Care,
Academic Unit of Elderly Care and Rehabilitation
Leeds Institute of Health Sciences
Temple Bank House
Bradford Royal Infirmary
Bradford
BD9 6RJ
Tel: 01274 383441
Mobile: 07503727990
Email: d.j.clarke@leeds.ac.uk

Professor Fiona Jones,
Professor of Rehabilitation Research,
Faculty of Health Social Care and Education
St George's University of London
2nd Floor Grosvenor Wing
Cranmer Terrace
London SW17 0RE
f.jones@sgul.kingston.ac.uk

Professor Ruth Harris,
Professor of Healthcare for Older Adults,
King's College London
Florence Nightingale Faculty of Nursing and Midwifery
Room 2.24, James Clerk Maxwell Building
57 Waterloo Road
SE1 8WA
ruth.harris@kcl.ac.uk
Tel: 020 7848 3708

Professor Glenn Robert,
Professor of Healthcare Quality & Innovation,
Kings College London
Florence Nightingale Faculty of Nursing and Midwifery
James Clerk Maxwell Building
Waterloo
United Kingdom
Telephone: +44 (0)20 7848 3063
E-mail: glenn.robert@kcl.ac.uk

on behalf of the Collaborative Rehabilitation in Acute Stroke (CREATE) team

Word count: 5192 excluding title page, abstract, references, figures and tables.

For peer review only

What outcomes are associated with developing and implementing co-produced interventions in acute healthcare settings? A rapid evidence synthesis.

Abstract

Background: Co-production is defined as the voluntary or involuntary involvement of users in the design, management, delivery and/or evaluation of services. Interest in co-production as an intervention for improving healthcare quality is increasing. In the acute healthcare context co-production is promoted as harnessing the knowledge of patients, carers and staff to make changes about which they care most. However, little is known regarding the impact of co-production on patient, staff or organizational outcomes in these settings.

Aims: to identify and appraise reported outcomes of co-production as an intervention to improve quality of services in acute healthcare settings.

Design: rapid evidence synthesis.

Data Sources: Medline, Cinahl, Web of Science, Embase, HMIC, Cochrane Database of Systematic Reviews, SCIE, Proquest Dissertation and Theses, EThOS, OpenGrey; CoDesign; Design Journal; Design Issues.

Study selection: studies reporting patient, staff or organisational outcomes associated with using co-co-production in an acute healthcare setting

Findings: 712 titles and abstracts were screened, 24 papers underwent full text review; 11 papers were included in the evidence synthesis. One study was a feasibility randomised controlled trial, three were process evaluations and seven used descriptive qualitative approaches. Reported outcomes related to: (a) the value of patient and staff involvement in co-production processes; (b) the generation of ideas for changes to processes, practices and clinical environments; and (c) tangible service changes and impacts on patient experiences. Only one study included cost analysis; none reported an economic evaluation. No studies assessed the sustainability of any changes made..

Conclusions: despite increasing interest in and advocacy for co-production there is a lack of rigorous evaluation in acute healthcare settings. Future studies should evaluate clinical and service outcomes as well as the cost-effectiveness of co-production relative to other forms of quality improvement. Potentially broader impacts on the values and behaviours of participants should also be considered.

Key words: Rapid evidence synthesis, systematic review, co-production, acute healthcare

For peer review only - http://bmjopen.bmj.com/site/about/guidelines.xhtml

Strengths and limitations of this study

This study is the first to systematically review outcomes associated with developing and implementing co-produced interventions in acute healthcare settings.

We have identified a lack of rigorous evaluation of effectiveness and cost effectiveness of co-produced interventions in the acute healthcare sector at both the service and system level.

The study did not access and review the much broader patient and public involvement, service improvement and clinical microsystems literatures unless specific terms such as ‘co-production’ and ‘co-design’ were used.

We only included studies where full-text reports published in English within the last 10 years could be retrieved; co-production studies published in other languages may have been overlooked by our review.

peer review only

What outcomes are associated with developing and implementing co-produced interventions in acute healthcare settings: a rapid evidence synthesis.

BACKGROUND

There is renewed interest in and advocacy for adoption of ‘co-production’ as a means of co-creating value across the public sector. However, as the term increasingly enters mainstream management (and healthcare quality improvement) discourses there is a sense that it may be being misused or misunderstood, thereby running the risk of becoming denuded of meaning or losing any association with its radical roots.

Co-production was first conceptualised in the United States in the 1970s, originally as a response to the lack of recognition of service users in service delivery [1]. The creation of time banks, a system reliant on the participation of volunteers who are also service users [2,3] showed how collaborative interventions that involve people with long-term psychosocial needs could contribute to improved community links [4]. Since this early work a variety of terms have become evident in the growing literature on co-production, and a range of related approaches are being enacted in different ways and at various levels throughout public and health sector services [5-11]. For the purposes of this paper we follow Osborne et al recent definition of co-production as ‘the voluntary or involuntary involvement of public service users in any of the design, management, delivery and/or evaluation of public services’ [8].

In the healthcare context co-production is promoted as harnessing the knowledge of patients, carers and staff to make changes about which they care most [12-13]. A recent example of using co-production in this way is a project to deliver and evaluate a mental health service to meet the needs of black and minority ethnic communities [14]. Berwick has recently argued for a greater focus on such co-production as part of a proposed ‘third era’ of medicine [15]. Potential benefits might include from a patient perspective improved satisfaction with the experience of care, reduction in complications associated with treatment and improved health outcomes [12-14]. From a staff and organizational perspective, the case for co-production may include improvements in staff wellbeing, increased recognition of the need for a better understanding of patient perspectives and changes in attitudes towards working with patients as partners in quality improvement [12-15].

For peer review only - http://bmjopen.bmj.com/site/about/guidelines.xhtml

In a recent commentary Batalden et al, whilst arguing that where healthcare activities are co-produced, services, providers and service users become far more effective agents of change, note that current systems can both support and constrain partnerships between patients and professionals (and that historically this kind of partnership has been unequal) [12]. The evidence base in terms of robust evaluation of the impact of co-production on patient outcomes appeared limited. Batalden et al argue this raises a series of questions for proponents of co-production including the need to determine '[m]easures that accurately reflect co-produced service and its results [and] measures which will help us understand co-productive processes' [12: 7].

Osborne et al, starting from the premise that co-production is (or has become over time) a 'woolly word', helpfully critiqued its conceptualization within the policy and practice literature, linked it directly to the co-creation of value in public service delivery and proposed a framework for differentiating 'the cluster of related concepts that are contained within the term 'co-production' [8]. This framework comprises 'four ideal types of value that are co-created in public service delivery by the iterative interactions of service users and service professionals with public service delivery systems'; namely co-production, co-design, co-construction and co-innovation. Voorberg et al conducted a systematic review of the literature (1987-2013) relating to co-creation/co-production with citizens in public innovation across all sectors, concluding that most studies focus on the identification of influential factors, while hardly any attention was paid to outcomes [16]. Greenhalgh et al report a narrative review of different models of co-creation relevant to community-based health services; they identify key success principles but note that 'impact is by no means guaranteed' [5].

For the purposes of this rapid evidence synthesis (RES) we identify co-production and co-design as specific approaches to co-creating value at the service (or clinical microsystem [13]) level within an acute healthcare organization. Co-design is an approach to participatory design (albeit traditionally of a new product) that seeks to actively involve all stakeholders (e.g. staff, patients, citizens) in a process to help ensure the result meets their needs and is usable. Such approaches are increasingly being used, often situated in the emerging Service Design field [17-18] as part of co-production projects. Specific forms of co-design such as Experience Based Design (EBD) [19-20] and Experience-based Co-design (EBCD) [21] have been developed and applied in the healthcare sector. We also follow Osborne et al conceptualisation of their final two 'types of value' (co-construction and co-innovation) as being concerned with service systems (rather than individual services) [8]; we return to this important distinction at the end of this paper.

We undertook this RES partly to inform an ongoing study (Collaborative Rehabilitation Environments in Acute Stroke- CREATE) which is bringing together staff, former patients and carers to review and jointly co-produce the way in which therapeutic rehabilitation-related activity is provided in stroke units in the early days and weeks after stroke [22]. In the absence of existing published evidence, this RES aims to identify and evaluate reported outcomes of co-produced interventions designed to achieve patient focused quality improvements in acute healthcare settings. To inform the empirical study our secondary objectives were to identify in such settings:

- those elements of co-production processes that lead to patient focused quality improvements;
- any evidence of cost-benefit analysis, cost reduction or other economic evaluation associated with co-produced interventions;
- barriers and facilitators reported to influence the implementation and use of co-production methods and implementation of co-produced interventions; and
- issues which impact sustainability of quality improvements following the use of co-production.

For peer review only - http://bmjopen.bmj.com/site/about/guidelines.xhtml

REVIEW METHODS

Whilst there are no agreed international guidelines for the design and conduct of RES [23] there is overall agreement the process involves providing an overview of existing research on a defined topic area, together with a synthesis of the evidence provided by these studies to address specific review questions. RES are typically completed in 2-6 six months; this does not normally allow for all stages of traditional effectiveness reviews [23-25]. However, RES are increasingly valued by policy makers who require rapid answers to specific questions to inform policy revision or service development [26-27].

Limitations of RES include the potential for introduction of bias. Sources of bias can include omission of critical appraisal of methodological quality of included papers. Similarly, limiting the scope of the search to published literature or specific databases, to a specific focus within a larger topic, or to a reduced timespan of publication can reduce the applicability of RES findings for some audiences. In RES of effectiveness, meta-analyses may be omitted. It is important therefore that RES are conducted systematically; methods used and limitations introduced by these should be reported transparently [23-26].

This RES was conducted between January and June 2016. We used a systematic approach and included full-text evidence published in English within the last 10 years. Search terms and databases searched were agreed by researchers and Information Specialists at the University of Leeds. Search terms used were specific to the use of co-production in acute healthcare settings (see supplementary file 1). To keep the search focused, we omitted broader search terms including cooperative behaviour, patient participation, collaborative approach, and service improvement.

Box 1: Database and hand searches

Medline, Cinahl, Web of Science, Embase, HMIC, Cochrane Database of Systematic Reviews, SCIE, Proquest Dissertation and Theses, EThOS and OpenGrey.

The following journals were hand searched:
CoDesign; The Design Journal; and Design Issues.

Database searches were conducted for the period January 1st 2005 to 31st January 2016. Given existing reviews [5, 16] and the CREATE study focus, we reviewed post 2005 evidence and only that reporting on studies in acute healthcare settings. We completed citation tracking of 5 seminal papers; five experts in co-production were requested to nominate 3-5 seminal papers relevant to our review. Inclusion criteria are shown in table 1:

Table 1: inclusion criteria

• Reports research using a co-creation or co-production, or co-design or Experience-Based Co-Design approach in an acute healthcare setting
• Reports patient or staff or organisational outcomes resulting from research using a co-creation, co-production, co-design, Experience-Based Co-Design approach in an acute healthcare setting<br><br>Outcomes of interest include:

For peer review only - http://bmjopen.bmj.com/site/about/guidelines.xhtml

- any measure of the outcome of co-produced interventions on patient focused quality improvements in acute healthcare settings as reported by patients or families or caregivers, or health service providers<br>- including patient reported outcome measures and patient or staff reported experience measures<br>- using qualitative or quantitative data
• Acute healthcare settings include:<br>- emergency departments/accident and emergency departments<br>- adult inpatient facilities including: acute medical or surgical admission units (often termed MAUs or SAUs), acute medical or surgical units, acute trauma units, acute neurological units, intensive or critical care units, acute care of the elderly or geriatric units, medical oncology or cancer services<br>- adult outpatient facilities including medical, surgical, trauma, neurology, care of the elderly or geriatrics, medical oncology or cancer services

Two reviewers independently read all titles and abstracts. Discrepancies in retain or reject decisions were addressed by discussion between reviewers with recourse to a third where consensus could not be reached. Three reviewers independently read the retrieved full text papers; initial decisions to retain or reject were made independently based on the inclusion criteria. All three reviewers then discussed retain and reject recommendations and reached a consensus agreement.

Three reviewers completed data extraction. We used quality appraisal checklists developed for quantitative and qualitative studies [28] (Table 2). These were selected as they were developed and used by the National Institute for Health and Care Excellence (NICE) in the appraisal of the quality of evidence underpinning NICE public health guidance. The checklists address fourteen areas of study quality ranging from theoretical approach through study design, data collection and analysis methods and also ethical review (Supplementary file 3 -quality assessment records). For each study two reviewers undertook data extraction and quality appraisal independently. Studies were not excluded on the basis of quality appraisal; this was used to inform the discussion of the evidence identified in the review. We adopted a mixed research synthesis approach [29] drawing on methods reported by Boger et al [30]. We used an integrated design which entailed grouping studies for synthesis not by methods (i.e., qualitative and quantitative) but rather by findings viewed as answering the same research questions, or addressing the same aspects of a target phenomenon.

RESULTS:

A total of 712 titles and abstracts were identified for screening from which 24 papers were identified for full text review. Following review, eleven publications were included in the final evidence synthesis (figure 1); full details of each study can be found in supplementary file 2.

Table 2: Quality Assessment study rating criteria [28]

++ All or most of the checklist criteria have been fulfilled, where they have not been fulfilled the conclusions are very unlikely to alter.
+ Some of the checklist criteria have been fulfilled, where they have not been fulfilled, or not adequately described, the conclusions are unlikely to alter.

For peer review only - http://bmjopen.bmj.com/site/about/guidelines.xhtml

– Few or no checklist criteria have been fulfilled and the conclusions are likely or very likely to alter.

Papers were published between 2008 and January 2016, with studies conducted in five countries (Canada, England, New Zealand, Australia, Netherlands). Settings included intensive care units (n=1), inpatient and outpatient oncology services, including breast, lung, colorectal and haematology services (n=5), mental health services (n=1), emergency departments (n=1), an outpatient clinic for people with multiple sclerosis (n=1), and older peoples outpatient services (n=1). A further study evaluated co-design projects in five Dutch hospitals; the projects were conducted in four oncology departments and a haematology department.

One study was a feasibility randomised controlled trial (RCT) of a co-designed intervention [31]. The study design was explicitly stated in only one of the remaining papers [32]; the remaining studies utilised descriptive qualitative approaches to evaluate changes in services or explore participant's experiences or views, although these were rarely described in detail. Data collection methods included self-report postal questionnaires; ethnographic observations of patient journeys through services and of staff working practices, semi-structured interviews with staff and patients (sometimes filmed), focus groups and emotional mapping exercises. Data analysis techniques were described in any detail in only six of the eleven publications [32-38]. Most papers focused on the processes used to understand and co-design within services rather than evaluating outcomes; the exception being the feasibility RCT [31].

Quality assessment ratings are shown in the final column of the table in supplementary file 2. Four studies were rated at ++ with the remaining seven at +. Ratings largely reflect the omission of detail on research methods, particularly in relation to stating research aims, questions, sampling decisions and discussion of data analysis and findings.

Figure 1: Adapted Prisma diagram insert here

Use of co-production methods

Methods reported included: a feasibility RCT of the impact of a co-designed intervention [31], participatory design [38], creative design [39], co-production [37], experienced-based design (EBD)[33,42], experienced-based co-design (EBCD) [34-37] and accelerated experienced-based co-design (AEBCD) [32,41]. EBD or variants of this approach was reported in eight of eleven included studies, with a further study [31] testing an intervention previously designed using EBCD [40]. Projects ran for 3 months to over one year. Whilst local tailoring of approaches was clear, the use of a staged process for co-production was evident across all studies. This indicated that whilst the classification of approaches varied, the majority demonstrated patient, carer and staff reflecting and working together using participative methods in joint review and co-design activities focused on improving patient and/or carer experiences.

There was variation in levels of participant engagement. In an oncology service, whilst patients and a wide range of staff were included in the creative design project, they participated separately in the three stages of the study and did not come together to share and discuss their respective experiences [39]. In the Thomson et al study a designer led, three-stage process was used in which carers and staff came together only once; this variation in participant engagement did not appear to have any impact of the range of practical service improvement outcomes [38]. However in the case of the Golden et al study [39] the lack of joint working between the patients, carers and staff may

For peer review only - http://bmjopen.bmj.com/site/about/guidelines.xhtml

have been a key reason why no specific outcomes or agreed plans to introduce the changes identified in the creative design process were reported.

Reported outcomes of co-produced interventions

Our primary aim was to identify and evaluate the reported outcomes of co-produced interventions designed to achieve patient-focused quality improvements in acute healthcare settings. The methods used to understand participants' experiences and obtain their views included observation, process mapping, interviews, focus groups and postal survey, more detail is reported in supplementary file 2. Overall, patients reported positive experiences of participation, consistent with those associated with a wide range of co-production in the public and healthcare sector [5,7,10,12, 16,]. Co-production or co-design projects were effective in generating a wide range of ideas and specific suggestions related to improving patients' experiences across the different settings. Reported service changes and quality improvements are summarised in table 3. However, clarity regarding implementation of improvements to services and/or evidence of resulting impact on patient outcomes was one of the main limitations of the reporting in the majority of the studies.

The feasibility RCT [31]evaluating the impact of a co-designed intervention to improve the knowledge, experience and emotional well-being of carers of people with breast, lung or colorectal cancer was the only study reporting use of a validated outcome measure, the GHQ-12 [31]. The other quantitative measures used in this study were adapted from existing measures (n=2, for example, supportive care needs) or developed by the study team (n=2, for example, experience of care and knowledge of chemotherapy). Carers who received the intervention reported significantly better understanding of symptoms and side effects and that their information needs were more often met compared with the control group [31]. Confidence in coping improved between baseline and follow-up for the intervention group and declined for the control (although differences were not statistically significant). Accepting the limitations of the single centre and small sample size, this study provided evidence for the effectiveness of the carer focused intervention developed in an earlier study by carers and staff using EBCD methods [40]. In the remaining 10 studies there were no reported instances of use of validated outcome measures, such as quality of life measures, nor any instances of validated patient reported experience (PREM) or outcome measures (PROM).

Four studies [33, 35, 37, 39], did not clearly differentiate between the findings from the co-production or co-design processes used and patient focused quality improvement outcomes. In these studies it was difficult to distinguish between ideas for improvement generated by co-design activities and tangible service improvements that had either been introduced or were planned. However, the reported 'outcomes' of the studies can be categorised in three main ways and are summarised in table 3 below:

1) patient and staff involvement in the co-production or co-design processes;

2) generating ideas and suggestions for changes to processes, practices and clinical environments impacting on patients' and/or carers' experiences of a service, (and often indirectly on staffs');

3) tangible change in services and impact on patient or carer experiences.

Elements of co-production that led to quality improvement

Not all authors commented directly on the process of co-production. Those that did identified that: co-production methods provided the means to ensure patients' experiences, concerns and ideas for change were captured, presented to and discussed with staff in services. Active engagement of patients in this way gave both legitimacy and urgency to service improvement plans [36,37], particularly where managers were involved in or actively supported co-production projects

For peer review only - http://bmjopen.bmj.com/site/about/guidelines.xhtml

[35,37,41,42]. Tsianakas et al [36] identified genuine and direct patient and carer involvement (relative to other service improvement projects in which patients and staff had participated) and linked this to patient participants taking direct responsibility for the work and its outcomes as being success factors linked to the EBCD process itself. Similar claims were evident in other EBD and EBCD projects [32, 33,37, 42], where patients perceived there to be a partnership with staff in the co-production process[32,33] and where staff reported facilitated co-design groups directly contributed to active and empowered partnerships between staff and patients in which provided genuine opportunities for patients to 'have their say'[32,33,37]. There appeared to be an equally powerful impact on some staff participating in co-production in that the methods required them to engage with and listen to patients in ways most had not experienced previously [34,36,37,42].

However, despite clear evidence of the contribution of co-design activities in generating ideas for patient focused service improvements (see table 3), in some projects staff and patients were frustrated by the lack of progress from problem and solution identification to actual quality improvements [33-35, 37]. In one case, the funded project appeared to end before concrete action planning to effect change, based on the ideas generated, had occurred [39]. Researchers ascribed this to a combination of the time taken to undertake co-design activities and the often slow processes of change in complex health service organisations.

Economic evaluation

There were no cost-benefit analyses related to reported outcomes for any of the studies. Only one study reported a cost analysis of co-production methods by comparing the cost of an accelerated EBCD approach (AEBCD) to standard EBCD [32]. A significant reduction in costs was reported for AEBCD. The cost of developing patient experience (trigger) films fell from £30,485 to £8,289 and, as the film developed could be used in future projects, this was a one-off cost. This study was also the only one to quantify costs of the co-design stages which were £20,276; over half of this sum was for the salary of local facilitators.

Other authors alluded to the costs associated with participatory or co-design methods in terms of the largely time-related costs involved in freeing staff to take part in or run the projects, or costs associated with actual or planned service improvements. These include, for example, improved signage [33], provision of personal care equipment [34], information booklets/leaflets, DVDs [32,36,37,38,42], a website and web based toolkit [42]or costs associated with having blood tests conducted off site [39]. However, no actual or estimated costs were reported for these elements. Similarly, nine of the eleven publications reported on researcher led, designer led or researcher supported projects but in only two cases were actual research costs traceable [32, 39].

Barriers and facilitators to implementation

The eleven studies encompassed nine projects conducted in diverse acute healthcare settings, although few of the reported barriers or facilitators were specific to project or setting. The most commonly reported barriers encountered in using co-production approaches in acute healthcare settings included a lack of support, resources or managerial authority to bring about structural or environmental changes [33,34,35,37,42]. In addition, practical or logistical problems were identified, for example, ensuring frail elderly people could attend regular co-design meetings [33]. Recruiting patients and carers and retaining them through the different stages of projects were highlighted by a number of researchers [32, 34-35, 37, 41]. In at least one project [34] some patients participated only in discovery or exploratory phases, then having 'shared' their experiences with staff, declined further participation arguing that they were no longer using the service. Researchers also identified the need to plan for and manage patients' understanding of what may be a radically different form of engagement with hospital staff, often quite unlike that experienced previously by users of health services[32,33,36]. In the studies reviewed, the frequency and duration of their involvement and

For peer review only - http://bmjopen.bmj.com/site/about/guidelines.xhtml

also the time it may take to bring about changes in the structures, processes and sometimes the physical environments of services were highlighted as factors to be addressed with participants [33,39,42].

In the majority of projects staff engagement was in addition to usual clinical or managerial roles; nonetheless, a high level of interest in and commitment to co-production activity was identified in almost all projects. However, this was impacted on in at least five projects by staffs' frustration at the expectation that they would undertake co-production/co-design work in their own time, that they could not allocate time out of their routine work and that additional support was often not provided by more senior staff [33-35,37, 42]. For some staff this made participation almost impossible [37]; for others it meant projects did not progress as expected [35] or contributed to tensions in co-design groups or between researchers and participants [33]. The duration of projects also increased the likelihood that staff turnover would impact on project leadership or involvement [34-35]. The need for structured and on-going managerial and organisational support was highlighted [32-34, 36, 38, 41,42] but only two studies expressly refer to governance or oversight groups set up to support co-design projects [33-34].

In projects where facilitators were engaged formally and funded to manage or oversee projects, it was more likely that projects (a) maintained momentum and were delivered as planned, (b) engaged and retained patients, carers and staff and (c) generated concrete examples of areas where patients' or carers' experiences could be improved [32,35-36,41]. In some studies, researchers (some of whom were designers) facilitated staff and patient engagement in the projects. Where designers were directly involved, our review suggests that they introduced ways of thinking and working which successfully challenged staff and patients to reconceptualise everyday processes and activities. This was achieved using metaphor games, design experiments, visual storyboards, prototyping, future focus groups, and emotional mapping; approaches not familiar to most health service staff [32-33, 38, 39]. However, with the exception of Locock et al [32] the costs associated with facilitator or researcher involvement were not identified.

Despite the barriers identified above, the studies suggest researchers and participants across settings viewed the benefits of this level of patient and staff involvement in structured co-production/co-design projects as outweighing the challenges.

Sustainability

None of the eleven studies formally evaluated whether co-production or co-design as a way of working had been sustained, or whether improvements made as a result of such approaches were sustained over any length of time. However, a number of concrete changes in service organisation, care environments and patient and in carer experiences occurring either during or closely related to co-production activities were reported, see table 3 [32,33-38,42]. Bowen et al [33] indicate that some changes may take two years or more to effect (for example, changes in hospital signage and routing), a period of time long after staff and patients had ceased to be actively involved in specific projects; another study reported that planned changes may not be achieved at all despite extending the life of projects [35].

[**Insert table 3 here**]

DISCUSSION

This rapid evidence synthesis has summarised peer reviewed evidence relating to the use of co-production approaches in focused quality improvement projects in the acute healthcare sector.

For peer review only - http://bmjopen.bmj.com/site/about/guidelines.xhtml

Different forms of co-production/co-design were most commonly reported and few criticisms of these methods were identified, in part reflecting an almost certain positive publication bias. However, critical reflections are emerging [37, 43,44]. These, in part explore the so-called 'dark side' of co-production recognising, for example: the potential for reproducing inequality as those who are most able to are those that co-produce; the risks of 'captured' co-production where participants are compelled, sometimes unknowingly, to co-produce; co-production as a substitution of labour (what Fotaki terms 'a race to the bottom in times of austerity' [43]); and co-production being appropriated as cover for inherently political decisions.

The kind of patient focused quality improvements reported in the identified studies can largely be considered examples of simple practical changes or 'sweating the small things' [45]. However, from the perspective of patients' experiences of and journeys through a service, these issues were likely to make care and treatment more humane and more person centred, as well as making journeys easier to engage with and negotiate. However, both Bowen et al [33] and Thomson et al [38] argue that the ideation tools in EBD and EBCD were limited and that incorporating 'designerly' thinking into co-production projects could stimulate greater creativity and alternative solutions in sometimes rigid health services.

The relative lack of robust evaluations we found may also reflect the stage of adoption of these types of methods in acute healthcare settings as we report, there is currently only one published feasibility RCT. However, there is a large-scale stepped wedge randomised controlled trial under way in nine Australian community mental health teams which is evaluating the effect of co-design on psychosocial recovery outcomes (such as willingness to ask for help and personal confidence and hope) [46]. In future studies, robust and routine evaluation of the costs and impacts of using co-production methods are required so that those leading and participating in quality improvement efforts in acute healthcare organizations can make informed decisions regarding their adoption in addition to (or instead of) other quality improvement methods. However, reliance on relatively broad and insensitive measures - such as patient satisfaction surveys- may overlook the real value placed by patients and staff on changes in the personal behaviour, attitudes, and culture of healthcare teams. The studies reviewed here consistently referred to these service level benefits of co-production for participants and services, as well as pointing to potential service system level effects such as those identified by Osborne et al [8].

As others have highlighted, the use of similar approaches in the community health care sector have not always 'follow[ed] through to significant and sustainable redesign' [5]. A recent narrative review of four different models of co-creation relevant to community-based health services proposed three 'key success principles', namely (1) a systems perspective (assuming emergence, local adaptation, and nonlinearity); (2) the framing of research as a creative enterprise with human experience at its core; and (3) an emphasis on process (the framing of the programme, the nature of relationships, and governance and facilitation arrangements, especially the style of leadership and how conflict is managed). The review authors proposed that co-creation 'failures' could often be tracked back to abandoning (or never adopting) these principles [5]. The publications in our RES do not specifically identify these principles and provide no clear evidence that such principles underpinned the planned research and service improvements noted. However, individually and collectively, included studies highlighted problems which arose when explicit adoption of such principles was not evident in the set up and delivery of the reported co-production projects in acute healthcare settings. Although the acute healthcare sector faces challenges associated with rapid patient turnover and with patients sometimes critically or terminally ill, the majority of studies in this RES demonstrate that co-production is possible and was valued by participants. These findings have helped in planning for the introduction of the EBCD approach in the CREATE study. Although none of the studies reviewed were conducted in stroke services, the facilitators and barriers identified in the RES, together with the principles identified in the Greenhalgh et al review [5] indicate key areas for research or service improvement teams to address with managers, staff, patients and relatives participating in the

For peer review only - http://bmjopen.bmj.com/site/about/guidelines.xhtml

EBCD project activity. Specifically, future studies should evaluate clinically related outcomes, for example through validated patient reported experience and outcome measures. Cost-effectiveness of co-production projects can and should be evaluated using standard health economic approaches. Equally important is the impact of service change on factors such as patient access to and flow through services and evaluating whether changes are sustained overtime. Potentially broader impacts on the values and behaviours of participants, including staff, are more difficult to measure but could be evaluated qualitatively and quantitatively and incorporated in the evaluation of co-production projects in acute healthcare settings.

Co-production is an emerging focus for research and evaluation in the health sector. Currently there is no international agreement on or consistent use of terminology to capture the range and diversity of participative approaches increasingly employed in health services worldwide. However, we acknowledge that the exclusion of broader search terms relating to patient participation, patient centredness, service improvement and clinical microsystems is a limitation of this rapid evidence synthesis. We limited search terms to those we anticipated were likely to identify research studies reporting on use of recognisable and structured co-production or co-design approaches. In so doing, we did not access the more diverse and extensive literature reporting on projects including patient or service user participation or projects which may be termed 'patient or user centred'. This literature commonly includes descriptive summaries, reports and commentaries; whilst these descriptions can be rich and informative in terms of service review and development, the publications often do not report on research or structured service improvement methods. We did search for evidence in a database of grey literature from Europe (Open-Grey) but identified no publications meeting our inclusion criteria. Our strategy of asking five experts in the field to nominate key publications was designed to reduce the likelihood we did not identify key research publications. Nonetheless, it is possible that the narrow focus of the search terms used in our rapid evidence synthesis means we did not identify research and evaluation of co-production projects in the health sector reported using some of the alternative terms described above. Such literature may have been consistent with or challenged our findings. However, this evidence synthesis drew on good practice guidelines for such reviews and was rigorously conducted [23, 25-26]. This study is the first to systematically review outcomes associated with developing and implementing co-produced interventions in acute healthcare settings.

Whilst, the potential benefits of using co-production in acute healthcare settings have been identified these must be set against the concerns identified by participants in the studies we reviewed that such approaches can be challenging to set up and implement in busy clinical environments where no formal, practical and financial provision is made for staff, patient and carer involvement on a sustained basis. Accelerated approaches may offer a partial solution but organisation level understanding of and commitment to these kinds of service improvement methods - as also identified by Greenhalgh et al [5] - remains key to their likely effectiveness.

CONCLUSIONS

We have identified a lack of rigorous evaluation of effectiveness and cost effectiveness of co-produced interventions in the acute healthcare sector at both the service and system level. Traditionally, co-production has been applied in community settings but there is growing recognition of the value of a more collective contribution to quality improvement work from those who are both delivering and receiving any form of public service [12,47]. At the clinical microsystem level within the healthcare sector there has been increasing adoption of co-production involving patients and staff, typically drawing on co-design approaches, as a means of improving the quality of frontline services [12-13]. Health services, whether public or private, operate within increasingly severe financial restraints where funding for quality improvement work is limited. Without robust critique, evaluation and evidence of the co-creation of value there is a danger of co-production becoming another management fad or fashion [48], with the meaning of the term itself appropriated and co-opted but no longer 'co-produced'.

For peer review only - http://bmjopen.bmj.com/site/about/guidelines.xhtml

Funding: This project is funded by the National Institute for Health Research Health Services and Delivery Research Programme (project number 13/114/95). The views and opinions expressed therein are those of the authors and do not necessarily reflect those of the Health Services and Delivery Research Programme, NIHR, NHS or the Department of Health.

Acknowledgements:

The arguments presented in this paper were partly informed by discussions during a workshop at the University of Nottingham on 7-8 July 2016 organised by Professor Andrew Gray and Professor Bruce Stafford: 'The Individual and the State: co-production and interdependency in the mixed economy of public service consumption'.

Our thanks go to Deirdre Andre and Sally Dalton, Information Specialists at the University of Leeds who helped refine the search terms and strategy and conducted the database searches. Tino Kulnic assisted with the initial screening of abstracts. Stephanie Honey and Parminder Dhiman, research fellows, also assisted with some of the data extraction and quality assessments.

Declaration of competing interests:

We have read and understood BMJ policy on declaration of interests and declare that we have no competing interests.

Author contributions:

DJC, FJ, RH and GR designed the rapid evidence synthesis and developed the search terms. GR conducted hand searches of the journals: CoDesign, The Design Journal, and Design Issues. DJC and FJ screened all abstracts and agreed on full text for review. DJC, RH and FJ reviewed all full texts and agreed inclusions and exclusions. DJC, FJ and RH independently completed data extraction and quality assessment for all included papers. DJC prepared the manuscript and GH, RH and FJ reviewed and revised the manuscript. All authors agreed the final version of the manuscript.

Data sharing: Not applicable

Figure legends:

Figure 1: Adapted PRISMA Flow Diagram

References

1. Realpe A and Wallace LM. What is co-production? London; The Health Foundation, 2016 http://personcentredcare.health.org.uk/sites/default/files/resources/what_is_co-production.pdf, accessed 19th August 2016
2. Boyle D, Clarke S and Burns S. Aspects of co-production: the implications for work, health and volunteering. London: New Economics Foundation 2006.
3. Needham C and Carr S (2009). SCIE Research briefing 31: co-production: an emerging evidence base for adult social care transformation. London: Social Care Institute for Excellence. http://www.scie.org.uk/publications/briefings/briefing31/ Accessed 22nd September 2016
4. Boyle D, Clarke S and Burns S (2006). Hidden work: co-production by people outside paid employment. London: Joseph Rowntree Foundation 2006.
5. Greenhalgh T, Jackson C, Shaw S. et al Achieving Research Impact Through Co-creation in Community-Based Health Services: Literature Review and Case Study. *The Milbank Quarterly*, 2016 94: 392–429.
6. Donetto S, Pierri P, Tsianakas V et al . 'Experience-based Co-design and healthcare improvement: realising participatory design in the public sector', *The Design Journal*, 2015 18(2): 227-248

For peer review only - http://bmjopen.bmj.com/site/about/guidelines.xhtml

7. Bovaird T (2007). 'Beyond engagement and participation: user and community co-production of public services'. *Public Administration Review*, 2007; 67: 846–860

8. Osborne SP, Radnor Z and Strokosch K Co-production and the co-creation of value in public services: a suitable case for treatment? *Public Management Review*, 2016 18(5): 639-653

9. Boyle D, Coote A, Sherwood S et al Right here, right now. Taking co-production into the mainstream. Discussion Paper. London, NEF/NESTA 2010.

10. Scottish Co-production Network. Co-production – how we make a difference together, http://www.coproductionscotland.org.uk/resources/, accessed 19th August 2016

11. Fugini M, Bracci E and Sicilia M. Co-production in the Public Sector. Experiences and Challenges. London: Springer 2016.

12. Batalden M, Batalden P, Margolis P et al. Co-production of healthcare service. *BMJ Qual Saf* BMJ Qual Saf 2016;25:509-517.

13. Nelson, E.C. Batalden,P. B., Godfrey,M.M. Lazar,J.S. (Eds) Value by Design: Developing Clinical Microsystems to Achieve Organizational Excellence, 2011. New-Jersey, Jossey-Bass

14. Lwembe S, Green SA, Chigwende J, Ojwang T and Dennis R. (2017) Co-production as an approach to developing stakeholder partnerships to reduce mental health inequalities: an evaluation of a pilot service. *Primary Health Care Research & Development* **18**: 14–23, doi:10.1017/S1463423616000141

15. Berwick DM. Era 3 for Medicine and Health Care. *JAMA*. 2016;315(13):1329-1330. doi:10.1001/jama.2016.1509

16. Voorberg, W.H. Bekkers, V. J. J. M. Tummers, L. G. (2014): A Systematic Review of Co-Creation and Co-Production: Embarking on the social innovation journey, *Public Management Review*, 2015; 17(9): 13335-1357

17. Sangiorgi, D. (2011). 'Transformative services and transformation design'. *Int Journal of Design*, 2011; 5: 29–40.

18. Sangiorgi D and Prendiville A (eds.). (In press) Designing for Service: Key issues and new directions. Bloomsbury Publishing; London

19. Bate SP and Robert G. Bringing user experience to health care improvement: the concepts, methods and practices of experience-based design. Oxford; Radcliffe Publishing 2007

20. Bate SP and Robert G. 'Experience-based design: from redesigning the system around the patient to co-designing services with the patient', *Qual & Safin Health Care*, 2006; 15(5): 307-310

21. Robert G, Cornwell J, Locock L, et al Patients and staff as co-designers of healthcare services. *BMJ* 2015; 350:g7714

22. Jones F, Clarke D, Robert G et al. Using co-production to improve patient carer and staff experiences in health care organizations: a multi-centre, mixed methods evaluation in inpatient stroke units. NIHR- HS&DR Project 2016 13/114/95, http://www.nets.nihr.ac.uk/__data/assets/pdf_file/0010/162469/PRO-13-114-95.pdf

23. Featherstone RM, Dryden, D.M., Foisy, M. et al. Advancing knowledge of rapid reviews: an analysis of results, conclusions and recommendations from published review articles examining rapid reviews. *Syst Rev* 2015 4(1):50. DOI 10.1186/s13643-015-0040-4

24. Khangura, S., Konnyu, K., Cushman, R., et al. Evidence summaries: the evolution of a rapid review approach. *Syst Rev* 2012; 1(1), DOI:10.1186/2046-4053-1-10

For peer review only - http://bmjopen.bmj.com/site/about/guidelines.xhtml

25. Ganann, R., Ciliska, D., & Thomas, H. Expediting systematic reviews: methods and implications of rapid reviews. *Implementation Science*, 2010 5: 56. DOI: 10.1186/1748-5908-5-56

26. Bevan, N. Connelly,N., Francis,J., et al Rapid Evidence Assessment Toolkit. Civil Service. http://webarchive.nationalarchives.gov.uk/20140509095856/http:/www.civilservice.gov.uk/networks/gsr/resources-and-guidance/rapid-evidence-assessment 2010 Accessed 5th February 2016

27. Peterson, K., Floyd, N., Ferguson, L., Christensen, V., & Helfand, M. (2016). User survey finds rapid evidence reviews increased uptake of evidence by Veterans Health Administration leadership to inform fast-paced health-system decision-making. Systematic Reviews, 5(1), 132.

28. NICE Methods for the development of NICE public health guidance (3rd edition). https://www.nice.org.uk/article/pmg4/chapter/1%20introduction 2010 Accessed 5th February 2016

29. Sandelowski M, Voils CI, Barrosso J Defining and Designing Mixed Research Synthesis Studies. *Research in the Schools* 2006 13 (1): 29

30. Boger E, Ellis J, Latter S, et al. (2015) Self-Management and Self-Management Support Outcomes: A Systematic Review and Mixed Research Synthesis of Stakeholder Views. *PLoS ONE* 2015 10(7): e0130990.

31. Tsianakas V, Robert G, Richardson A, et al. (2015) 'Enhancing the experience of carers in the chemotherapy outpatient setting: an exploratory randomised controlled trial to test the impact, acceptability and feasibility of a complex intervention co-designed by carers and staff', *Supportive Care in Cancer* 2015 23(10):3069-80

32. Locock, L Robert G, Boaz A, et al, Using a national archive of patient experience narratives to promote local patient-centered quality improvement: an ethnographic process evaluation of 'accelerated' experience-based co-design. *Journal of Health Services Research &Policy* 2014, 19(4) 200–207

33. Bowen, S. McSeveny, K, Lockley, E et al, How was it for you? Experiences of participatory design in the UK health service. *CoDesign* 2013 9:4, 230-246

34. Piper, D. Ledema R, Gray J, et al, Utilizing experience-based co-design to improve the experience of patients accessing emergency departments in New South Wales public hospitals: an evaluation study. *Health Services Management Research* 2012;25: 162 – 172

35. Larkin, M Boden, ZVR, Newton, E et al, On the Brink of Genuinely Collaborative Care: Experience-Based Co-Design in Mental Health. *Qualitative Health Research* 2015, 25(11) 1463–1476

36. Tsianakas,V Robert G, Maben J, et al, Implementing patient-centred cancer care: using experience-based co-design to improve patient experience in breast and lung cancer services. *Supportive Care in Cancer* 2012 20:2639–2647

37. Vennik,D van de Bovenkamp, H.M. Putters, K et al, Co-production in healthcare: rhetoric and practice. *Int Rev Administrative Sciences* 2016; 82(1) 150–168

38. Thomson A, Rivas C, Giovannoni G. Improving the quality of participant interaction and ideation tools within service improvement activities. *BMC Health Services Res*, 15:105

39. Golden.B.R. Hannam, R., Fraser H. et al, Improving the patient experience through design. Healthcare Quarterly 2011. 14(3)32-41

40. Ream E, Tsianakas V, Verity R et al Enhancing the role of carers in the outpatient chemotherapy setting: a participatory action research project. Final report. London; King's College London 2013 http://www.dimblebycancercare.org/wp-content/uploads/2013/09/Dimbleby-report-executive-summary-pdf-3rd-Sept.pdf, accessed 20th August 2016

For peer review only - http://bmjopen.bmj.com/site/about/guidelines.xhtml

41. Tollyfield, R. Facilitating an accelerated experience-based co-design project. *Brit Journal of Nurs*, 2014, 23, (3):136-141

42. Boyd. H. McKernon, S, Mullin, B et al, Improving healthcare through the use of co-design. The *New Zealand Medical Journal* 2011 125,76–87

43. Fotaki, M. Co-Production under the financial crisis and austerity. A means of democratizing public services or a race to the bottom? *Journal of Management Inquiry* 2015: 24(4): 433-438.

44. Meijer A. Coproduction as a structural transformation of the public sector. *Int Journal of Public Sector Management* 2016 29(6): 596-611

45. Moore C and Buchanan D. Sweat the small stuff: a case study of small-scale change processes and consequences in acute care. *Health Serv Manage Res* 2013; 26: 9–9.

46. Palmer V, Chondros P, Piper D, et al. The CORE Study protocol: a stepped wedge cluster randomized controlled trial to test a co-design technique to optimize psychosocial recovery outcomes for people affected by mental illness. *BMJ Open* 2015 5:e006688 DOI:10.1136

47. Robert G and MacDonald A. (2017) 'Infrastructuring for organisational creativity and quality improvement in the healthcare sector: 'designerly' or 'design-like?'. In: D Sangiorgi and A Prendiville (eds). Designing for Service. Contemporary Issues and Novel Spaces, London: Bloomsbury

48. Walshe K. Pseudoinnovation: the development and spread of healthcare quality improvement methodologies, *International Journal for Quality in Health Care* 2009, 21 (3) 153-159

For peer review only

Table 3: Reported outcomes of co-production in acute healthcare settings

Type of outcomes	Evidence synthesis	Studies	Example
**Patient & staff involvement**	Patients perceived being really listened to by staff, having the sense that involvement was genuinely collaborative and that staff were committed to working with patients to improve services.	32,33, 34,36,41	Key to success was thought to be the strong relationship between patients and staff that has been built over time. (36)<br>Co-production' approach was seen to give patients, carers and staff a deeper understanding of each others' experience of the processes of care. It provided an opportunity to work meaningfully together, and this strengthened relationships among those involved in EBCD (34)<br>*'It was good, it felt as though [the hospital] and the professional staff were really interested … There was a comradeship if that's the right word …' (Older Patient- 33p)*
	Patients and staff recognised levels of participation and collaboration were distinct from any quality improvement activity they had experienced previously.	33,34,36,37	EBD's techniques facilitated building trust and rapport (33)<br>EBCD was perceived as a significantly higher level of genuine direct patient and carer involvement (relative to other service improvement projects in which patients and staff had participated) - *'the constant feedback [being] really, really useful', and '[needing] the eyeballing of each other to make it work'.* (36)<br>The EBCD process was thought to have given patients a greater sense of direct responsibility for the work and its outcomes (36, 37) and led to an increased understanding of the 'other' perspective, resulting in a potentially broader cultural change in mind-sets and behaviour. Staff participating in the co-design groups reported a greater sense of empowerment to make changes to their service (36).<br>Allowed nurses to reconnect with their core values of caring and compassion; development of a sense of empathy with patients was also evident.
	Staff realised that they often really did not have a good understanding and insight into patients' experiences of the services they provided.	36,37, 41, 42	Letting their 'own' patients talk about their experiences in a common 'action' setting affects professional staff, as patient stories make visible what the impact of (already known) problems really is on patients. The active role of patients and the way they express their experiences creates a sense of urgency to act on the improvement issues raised. (37)<br>Staff identified that working with patients in this honest and open sharing process was moving and at times humbling (36).
	There were also negative perceptions reported by a small number of patients and staff. These related to questioning the value of the amount of staff and patient time invested, and to not being able to see or experience actual	33,34	*'[The project] Seemed to go on for a long time . . . 'and I don't think we were properly prepared for that . . . my initial impression was that it would be perhaps a couple of interviews, and a discussion [Older patient-33:p]*<br>Both patients and staff identified that some service changes did not happen e.g. 'not sure if it has had any effect' (patient), 'in some areas, the area has not been improved' (staff), 'there's a few changes … [but] … there was some really serious things … they were highlighted, there was no resolution' (staff) (33)<br>Authors noted that although consensus reached quite easily, staff groups had tendency to focus on more positive

For peer review only - http://bmjopen.bmj.com/site/about/guidelines.xhtml

	service changes.		aspects of care and recent developments/improvements whereas service users and carers were more openly dissatisfied(34).
	Negative staff experiences and perceptions were also evident; these all related to challenges in conducting co-production or co-design projects.	33,34, 35, 37,42	Staff (and patients) reported little or no change had occurred. *'there's got to be something at the end of it'* (Staff Member-33p).<br>Staff viewed EBCD as a burden, additional to an already busy schedule (34, 37).<br>Staff workloads increased where EBCD was additional to usual roles (33, 35, 37, 42).<br>Lack of time and high level organisational support for Steering Group to complete implementation plans (35).<br>Lack of organisational capacity to respond to 'bottom up' service development (35).<br>Fee for service systems do not directly reward (or encourage) EBCD/co-production projects (37).
**Generating ideas & suggestions for changes to processes, practices & clinical environments**	The number of ideas generated or issues highlighted ranged from 5-11 core issues, through 48 co-design activities to 400 ideas generated by staff in an oncology service project.		
	A significant number of factors affecting patient experience from across the range of acute healthcare settings related to the structure and process of care and service provision.<br>Changes spanned:<br>-information and communication processes,<br>-environmental and organisational changes (and to a lesser extent)<br>-staff training and development.	33,36,40,42, 37, 42, 35	New template for patient letters (33)<br>A patient journey map and accompanying journey guide was developed linked to a patient held record (42).<br>A clinic guide and clinic (terms) dictionary were developed for new patients (38).<br>Redesigned discharge summary, improved cross site information booklet for patients transferring to another hospital for surgery (32)<br>Patient focused visual journey guide (42),<br>Proposals for layout of roads surrounding the outpatient building, design proposals for new way-finding materials (signage and maps) (33).<br>Guidance on diagnosis procedures included in junior doctors' induction (36).<br>Informal staff training programme-'recent developments in' (38).<br>A co-design toolkit and website for other services in New Zealand(42).

For peer review only - http://bmjopen.bmj.com/site/about/guidelines.xhtml

	Acute hospital admission and care have impacts beyond individual patients; involvement of families and carers - evident in five of the studies - appears to have helped ensure that those involved in the patients journey were also able to comment on and shape that experience.	31, 35, 36,	Administrative staff receive customer care training watch the patient experiences) DVD (33).<br>Ensuring that when carers came to visit, they were greeted appropriately at the door of the unit (32)<br>Clinic structures changed to reduce waiting times (33).<br>Carers information leaflet increases knowledge and understanding of symptoms and side effects and carers confidence (28).
	The structured co-production and co-design methods employed empowered both patients and staff to collaboratively review services and to plan and undertake changes in their workplaces.	32, 33, 34,36, 38,42,	Despite concerns about activities being '*a bit daunting at first*', participants supported researchers' perceptions that sharing experiences via storytelling and emotional mapping helped to build empathy and cohesion in the project group (30).<br>The opportunity to hear directly from patients and carers reportedly had a transforming effect on some staff. *'Our data suggest face-to-face encounters with patients in co-design groups often had a profound effect on staffing making them think differently about their practice and reconnect with their core professional values, resulting in renewed motivation* (29p).
	Involving designers/design researchers in co-production projects added value though challenging staff and patients way of thinking about their healthcare journey and experiences and how these could be improved within existing service settings. However, as design led techniques may be unfamiliar to staff and patients these and their purpose need to be carefully explained if they are not be seen as time wasting or games.	33,38,39	Forum theatre training to improve staff awareness of 'customer care' (33).<br>Use of metaphor, analogy and prototyping techniques (travel documents linked to an ideal patient journey). The props and the analogy facilitated the co-design process by enabling participants to not just suggest solutions to problems but create and share more radical suggestions for change (such as initiating a Patient Advisory Group) (38).<br>Photojournalling and storyboarding (39)
**Tangible change in services & impact on patient or carer experiences**	Patient and carer experience: Overall patients reported positive experiences of participation. Despite the barriers identified participants across settings viewed the benefits of patient and staff involvement in structured co-production/co-design projects as outweighing the challenges.	31, 32,34,36,42	Carers who received the co-designed intervention reported significantly better understanding of symptoms and side effects and that their information needs were more often met than the control group. Confidence in coping improved between baseline and follow-up for the intervention group and declined for the control)(31)<br>Patients can opt to receive chemotherapy treatment information in a group with others having the same treatment (36).<br>More comfortable V shaped pillows for post-operative patients (32).<br>Changed process for porters to remove waste avoiding ICU rest times (32)

For peer review only - http://bmjopen.bmj.com/site/about/guidelines.xhtml

			Provision of free refreshments (patients and families) (34).<br>New design for mammography gown (42).
	Service changes (processes and environmental change): It was not always possible to distinguish changes that had been implemented from those which were planned. Reporting of service improvement outcomes per-se was a limitation of the included studies, but the projects reported largely indicated that the structured co-production and co-design methods employed empowered both patients and staff to collaboratively review services and to plan and undertake changes in their workplaces.	32,34,36,37,38,42	Introduction of mini-Schwartz rounds on ICU (32).<br>New private room identified for receiving support after diagnosis (32).<br>Installation of a new air-conditioning unit in the waiting room, installation of a blanket warming cupboard, installation of payphones in waiting areas (34)<br>Changes to structure of clinics to reduce waiting times (36).<br>Decoration of waiting rooms (37)<br>Volunteer in the outpatient clinic linked to a patient advisory group (38).<br>Psychosocial care offered proactively during consultations (one hospital) (37).<br>Patient held record to track appointments (42)

For peer review only - http://bmjopen.bmj.com/site/about/guidelines.xhtml

For peer review only

For peer review only

For peer review only

For peer review only - http://bmjopen.bmj.com/site/about/guidelines.xhtml

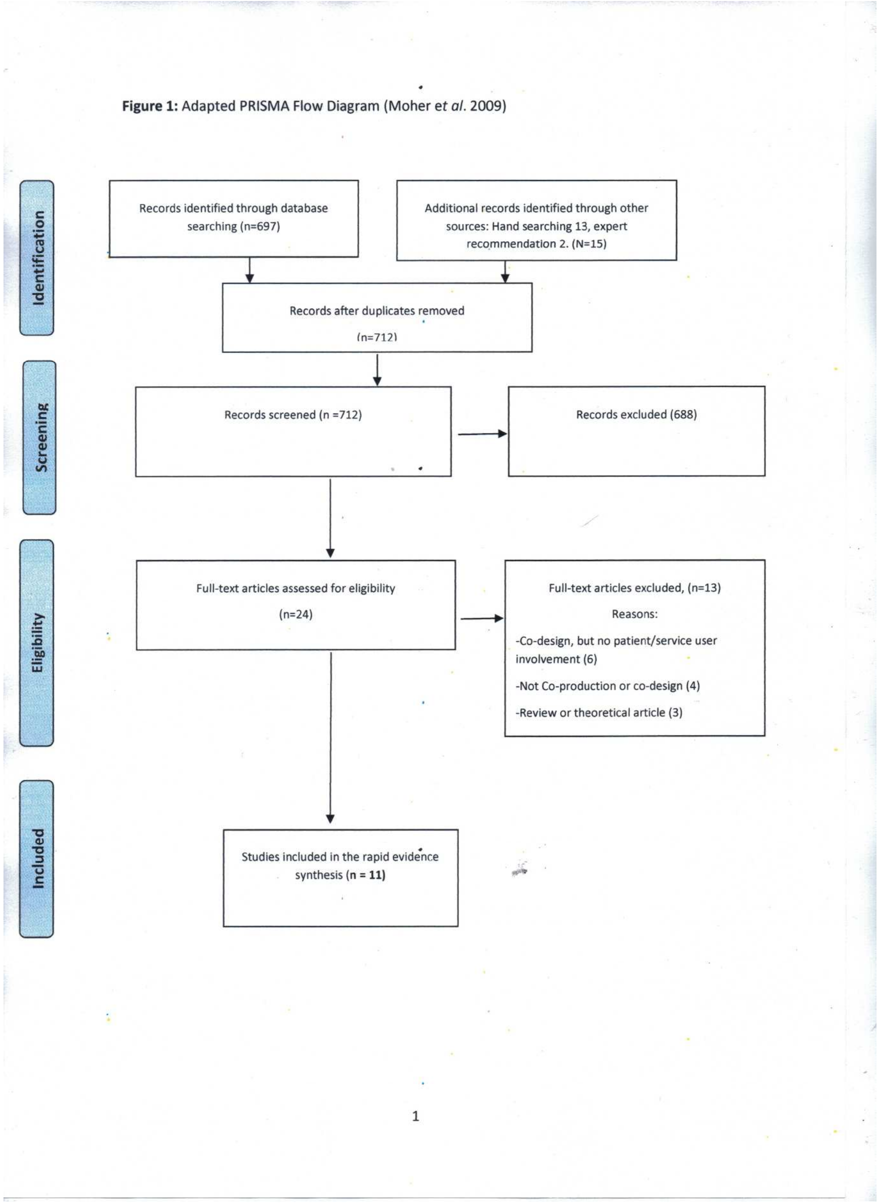

Figure 1 Adapted Prisma Flow Diagram

215x297mm (300 x 300 DPI)

Supplementary file 1 Search strategy (MEDLINE example)

Database: Ovid MEDLINE(R) <1996 to November Week 3 2015>

----

1 co-produc*.tw. (1149)

2 coproduc*.tw. (620)

3 co design*.tw. (53)

4 codesign*.tw. (16)

5 co creat*.tw. (171)

6 cocreat*.tw. (52)

7 (co research and design).tw. (1)

8 experience based design*.tw. (7)

9 participatory design*.tw. (149)

10 (experience adj3 design).tw. (252)

11 (evidence* adj2 design*).tw. (550)

12 EBC?.tw. (1604)

13 collaborative design.tw. (44)

14 human centred design.tw. (10)

15 human centered design.tw. (38)

16 people centred design.tw. (0)

17 people centered design.tw. (0)

18 inclusive design.tw. (32)

19 practice led design.tw. (1)

20 practice based design.tw. (2)

21 interactive design.tw. (31)

22 open design.tw. (147)

23 user centred design.tw. (51)

24 user centered design.tw. (177)

25 or/1-24 [co-design] (5093)

26 (acute adj (setting* or hospital* or care or healthcare)).tw. (15023)

27 ((secondary or speciali?ed) adj care).tw. (4410)

28 hospital care.tw. (4817)

29 exp Hospitals/ (117870)

30 Hospitalization/ (52132)

31 (hospitali?ation* or hospitali?ed).tw. (119506)

32 Inpatients/ (12602)

33 inpatient*.tw. (52413)

For peer review only

34 emergency medicine/ (8188)

35 Emergency treatment/ (8750)

36 emergency hospital admission*.tw. (240)

37 emergency hospitali#ation.tw. (116)

38 critical care/ (14911)

39 ambulatory care/ (20495)

40 urgent care.tw. (960)

41 or/26-40 [acute terms] (343100)

42 Delivery of Health Care/ (45257)

43 Efficiency, Organizational/ (17793)

44 Efficiency/ (4714)

45 Health Services Research/ (23584)

46 "Outcome Assessment (Health Care)"/ (50681)

47 patient outcome assessment/ (1713)

48 exp Program Evaluation/ (54529)

49 Quality Assurance, Health Care/ (38609)

50 Quality Improvement/ (9440)

51 Quality Indicators, Health Care/ (11744)

52 Quality of Health Care/ (43880)

53 (bench mark* or benchmark*).tw,kf. (17315)

54 (delivery adj2 health*).tw,kf. (12585)

55 (efficien* adj2 (assess* or assurance* or evaluat* or improv* or indicat* or measur* or test*)).tw,kf. (23714)

56 (performance adj2 (assess* or assurance* or evaluat* or improv* or indicat* or measur* or test*)).tw,kf. (74378)

57 (outcome* adj2 (assess* or assurance* or evaluat* or improv* or indicat* or measur* or test*)).tw,kf. (256084)

58 (program* adj2 (assess* or assurance* or evaluat* or improv* or indicat* or measur* or test*)).tw,kf. (27836)

59 or/42-58 [outcome or evaluation terms] (611767)

60 25 and 41 and 59 [co-design and acute care and outcome or evaluation terms] (45)

61 exp animals/ not humans.sh. (2031513)

62 60 not 61 [human only studies] (45)

63 limit 62 to (english language and yr="2005 - 2015") (40

For peer review only

Supplementary file 2 Empirical studies included in review

Publication/ country	Form of co-production	Setting	Methods	Reported Outcomes	Barriers and facilitators	Health economic evaluation	Quality rating
Bowen, S. et al, 2013 How was it for you? Experiences of participatory design in the UK health service. CoDesign, 9:4, 230-246<br><br>UK	Experience Based Design (EBD)	Medical outpatient service for older people. In a Teaching Hospital NHS Trust	Face to face (2) or telephone (9), semi-structured interviews (n= 11)	*Reported EBD Outcomes:*<br>• New template for patient appointment letters<br>• Design proposals for new way-finding materials (signage and maps)<br>• Proposal for layout of roads surrounding the outpatient building<br>• Video of patient stories distributed to all staff in the Trust to highlight older people's experiences,<br>• Forum theatre training event to improve outpatient staff awareness of customer care.<br>*Interview Findings*:<br>• Doing EBD (4 subthemes).<br>• Reflecting on EBD & Better Outpatient Services for Older People' (5 subthemes).	*Barriers:*<br>• Perception that participants were not doing the designing<br>• Perception that too many people were involved<br>• Ageing physical infrastructure Bureaucratic nature of trust procedures<br>• Low expectations of extent of change<br>• Availability of financial resources<br>• Staff workload<br>*Facilitators*:<br>• Sharing stories to gain perspectives of others<br>• Emotional mapping to highlight touchpoints (experiences and areas for improvement)<br>• Involvement in co-designing, being listened to by hospital staff	Not evident or reported.	++
Boyd. H. 2011 et al, Improving healthcare through the use of co-design. The New Zealand Medical Journal, 125,76–87	Experience Based Design (EBD) (Adapted)	Breast Services (inpatient & outpatient) in two hospitals in one District Health Board.	Patient journey mapping, experience surveys and co-design workshops.	• Patient journey map for staff review<br>• Patient Information folder to help navigate the service<br>• 7 new information leaflets,<br>• Patient held record to track appointments	*Barriers:*<br>• Staff workloads; EBD in addition to usual roles<br>• Financial restraints for hospitals and services<br>• Evaluation and planning for sustainability not factored into the	Not evident or reported.	+

For peer review only

Publication/ country	Form of co-production	Setting	Methods	Reported Outcomes	Barriers and facilitators	Health economic evaluation	Quality rating
New Zealand				• Patient focused visual patient journey guide<br>• Poster providing (better) communication guidance for staff and patients<br>• New design mammography gown,<br>• Co-design toolkit and website for other NZ services	EBD project<br>*Facilitators:*<br>• Engaging with patients at the outset of the co-design process before decisions are made<br>• Adopting a flexible approach to enable patients still receiving treatment to participate over time<br>• Working to get key staff buy in (clinical, managerial, administrative) & continued engagement<br>• Find & use ways to challenges participants' thinking, to work outside of their 'comfort zone'		
Golden.B.R. et al, 2011 Improving the patient experience through design. Healthcare Quarterly. 14(3)32-41<br><br>Canada	Rotman Creative Design Process<br>(co-design approach)	Cancer research and treatment centre.<br>Systemic therapy:<br>chemotherapy and transfusion services.	Ethnographic observations. Photojournalling & follow up interviews.<br>Staff discussion group: patient vignettes/ story-boarding ideal patient journey<br>Patient discussion group & storyboarding ideal patient journey.	• Prototypes and a jointly designed ideal patent journey	Not specifically reported.<br><br>Indicates staff workload and patient treatment scheduling pressures precluded bringing the two groups together to co-design service improvements.	Not evident or reported.<br><br>Project funded by charitable & corporate donations to the hospital & the University.	+
Larkin, M et al, 2015 On the Brink of Genuinely Collaborative Care: Experience-	Experience-based Co-Design<br>(EBCD)	Early Intervention Services and Inpatient units (n =7)	In depth interviews with service users (n=6), parents (n=6), inpatient staff (n=9).	Six priorities for service improvement identified from interviews and feedback group work:	*Facilitators:*<br>• Support from senior NHS staff<br>• Researchers leading the processes	Not evident or reported	+

For peer review only

Publication/ country	Form of co-production	Setting	Methods	Reported Outcomes	Barriers and facilitators	Health economic evaluation	Quality rating
Based Co-Design in Mental Health.Qualitative Health Research2015, Vol. 25(11) 1463–1476<br><br>UK	(adapted)	at two hospitals in the Midlands.<br><br>Mental health services for young people and families.	Feedback groups (20) involving 150 stakeholders.<br><br>Co-design event (50 participants).	• pathways in and out of hospital<br>• providing staff with a rewarding and well-supported role<br>• communicating with families and service-users<br>• recovery-focused practice<br>• creating a positive environment for everyone<br>• recognizing and sharing good practice across professions and services<br>• Co-design groups at the co-design event produced 13 action plans to address the 6 priorities	• Involvement of people internal and external to the service in the EBCD team aided project management<br>• Sensitivity to the needs and experiences of services uses in this setting in identifying touchpoints and developing trigger films to share with staff<br><br>*Barriers:*<br>• Challenging for some staff to hear strong service-users and carer views on services<br>• Lack of time and high level organisational support for Steering Group to complete implementation plans<br>• Steering group lacking the power to bring about change in organisations<br>• Organizational restructuring resulting in some study collaborators and “champions” leaving or changing role,<br>• Improvements requiring input from other departments or requiring strategic, budgetary, or staffing commitment<br>• Lack of organizational capacity to respond flexibly or directly to “bottom-up” service development		
Locock, L et al, (2014) Using a	Accelerated Experience-	In intensive care and	Process evaluation using ethnographic	*Re: AEBCD*	*Anticipated Barriers:*	In AEBCD the cost of	++

For peer review only - http://bmjopen.bmj.com/site/about/guidelines.xhtml

Publication/ country	Form of co-production	Setting	Methods	Reported Outcomes	Barriers and facilitators	Health economic evaluation	Quality rating
national archive of patient experience narratives to promote local patient-centered quality improvement: an ethnographic process evaluation of 'accelerated' experience-based co-design. Journal of Health Services Research &Policy2014, Vol. 19(4) 200–207<br><br>UK	based Co-Design (AEBCD)	lung cancer services in two English NHS hospitals	methods:<br>• observations of AEBCD processes and events (155 hours)<br>• interviews (n=30)<br>• repeated group interviews with patient participants<br>• reflective diaries (n=22)<br>• service improvement logs (n=4)<br>• evaluation questionnaires (post AEBCD events)	Trigger films developed through secondary analysis if existing films from the Oxford University collections of patient experience interviews were acceptable to patients and staff and were used successfully in subsequent co-design work.<br><br>*Re: Service improvements*<br>48 co-design activities across the four patient pathways. Examples reported were:<br>• Improved cross-site information booklet for patients transferring to another hospital for surgery<br>• A redesigned discharge summary with input from all professions<br>• More comfortable V-shaped pillows for postoperative patients<br>• Sourcing clocks to aid patient orientation in ICU<br>• Changed process for porters to remove waste avoiding ICU rest times<br>• New private room identified for receiving support after diagnosis<br>• Introducing mini 'Schwartz rounds' on ICU	Films developed from a national archive would be considered inappropriate or an inaccurate reflection of care in a specific hospital.<br><br>Barrier not evident: 85% of participants (patients and relatives) reported films to be good or excellent.<br><br>No other barriers reported<br><br>*Facilitators:*<br>• Regular face-to-face meetings with patients in co-design groups reminded staff of who change was for and why it mattered<br>• Co-design groups facilitated active and empowered partnerships between patient and staff	developing a trigger film was £8289, compared to £30,485 for EBCD.<br><br>Co-design<br>phase costs, were £20,276<br>(over half of this was facilitator salary).	

For peer review only

Publication/ country	Form of co-production	Setting	Methods	Reported Outcomes	Barriers and facilitators	Health economic evaluation	Quality rating
Piper, D. et al, 2012 Utilizing experience-based co-design to improve the experience of patients accessing emergency departments in New South Wales public hospitals: an evaluation study. Health Services Management Research2012;25: 162 – 172<br><br>Australia	Experience-based Co-Design (EBCD)	Emergency Department (EDs) & related services in two (programme) stages in seven public hospitals in New South Wales, Australia.	Review of EBCD process and evaluation reports provided by participating sites.<br><br>Interviews of patients, clinicians and managers, and also of high-level project managers including executive sponsors, clinical leads, project managers across sites (n=117)	EBCD processes provided opportunities to work together meaningfully and enabled patients, carers and staff to develop a deeper understanding of each other's experience of care processes, and this strengthened relationships among those involved in EBCD.<br><br>Examples of co-designed strategies implemented and sustained at the programme sites included:<br>• Recruitment of a full-time cleaner<br>• Installation of a new air conditioning unit in the waiting room<br>• Installation of a blanket warming cupboard<br>• Installation of food vending machines in the waiting area<br>• Access to free refreshments<br>• Installation of pay phones in the waiting area<br>• Patient access to 'sick-bay' bags in the waiting area<br>• Increased security presence in the emergency room.<br>22 examples are provided in the	*Barriers:*<br>• Staff viewed EBCD as a burden additional to already busy schedule<br>• Limits in implementation officers' & clinical leads' EBCD roles, reportedly reduced impact of programmes & long-term change<br>• Consumer recruitment & retention was difficult due to the transitory nature of the care episode<br>*Facilitators:*<br>• Develop a consumer engagement strategy, using on-going communication including face-to-face bi-monthly re-connect meetings, telephone & email contact/updates<br>• Integrating the EBCD process into everyday work practices rather than presenting EBCD as a separate task<br>• Adequate resources & reporting opportunities to executive meetings<br>• Clear accountability for implementation, allow adequate time to embed solutions into daily practice and cultures of EDs<br>• Appointing a liaison person to: ensure regular participant contact; manage governance of project & processes; communicate & contextualize aims & timeframes of EBCD to staff; develop a communication strategy to inform all stakeholders of the status & impact	Not evident or reported.	+

For peer review only

Publication/ country	Form of co-production	Setting	Methods	Reported Outcomes	Barriers and facilitators	Health economic evaluation	Quality rating
				publication.<br><br>Implementation strategies also identified.	of solutions on an on-going basis		
Thomson,A., Rivas,C., and Giovannoni. G. Multiple sclerosis outpatient future groups: improving the quality of participant interaction and ideation tools within service improvement activities. BMC Health Services Research (2015) 15:105. DOI 10.1186/s12913-015-0773-8<br><br>UK	Participatory approach drawing on techniques from the speculative design field.	An outpatient clinic for patients with multiple sclerosis in London, UK.	3 staged process using focus groups (future groups). In stages 1 and 3 patient participants and staff participants worked separately. In stage 2 the two groups were combined.<br><br>Process completed in 3 months<br><br>Speculative design techniques included: directed patient and staff journey mapping; combining patient and staff maps of an ideal (patient) journey (using analogies and props-a set of travel related documents); developing service improvements (represented on researcher developed separate patient and	• Volunteer in the outpatient clinic, linked to a Patient Advisory Group meeting 3 monthly<br>• A guide to the MS clinic for new outpatients<br>• An MS clinic dictionary (covering medical terms related to MS and frequently used in the clinic)<br>• A walking map with distances to the clinic (from the underground or disabled parking areas or local coffee shops)<br>• Magazine replenishment (as a clinic resource)<br>• Informal staff training programme (developments in MS research and care)	Facilitators:<br>• Adding (speculative) design led techniques to a participatory approach broadly similar to the 6 stage EBCD approach<br>• Adapting the design led props for the specific healthcare context<br>• Involving the design researcher directly in the (future) focus groups<br>• Including senior staff/managers in the stage 3 (future) focus groups to facilitate understanding of proposed service developments and their implementation.<br>Barriers:<br>• Adapting design led methods for health services improvements projects may reduce their creative contribution if used in a procedural manner<br>• Participant engagement with design led metaphor and analogy driven techniques need to be considered carefully and reflect local and disease specific contexts	Not evident or reported.	+

For peer review only

Publication/ country	Form of co-production	Setting	Methods	Reported Outcomes	Barriers and facilitators	Health economic evaluation	Quality rating
			staff conference style posters (prototyping).				
Tollyfield, R. 2014 Facilitating an accelerated experience-based co-design project. British Journal of Nursing, 2014, l23, (3):136-141<br><br>UK	Accelerated Experience-based Co-Design (AEBCD)	Specialist cardiothoracic intensive therapy unit (ITU) in London.<br><br>(One of the four units involved in the Locock et al 2014 study)	Reflection on the role of facilitator in an AEBCD project in one of two ITUs participating in the project.	Reports on the experience of and learning from facilitation of the AEBCD study conducted by Locock et al (2014).<br><br>• Promoting staff engagement a key aspect of the project- via posters, emails, one-to-one discussions and a ITU weekly newsletter<br>• Developing solutions to low patient recruitment levels<br>• Providing facilitation of the main project events and co-design meetings<br>• Prompting and supporting implementation of planned change	*Facilitators:*<br>• Project supported and endorsed by senior managers and clinicians- Director of Nursing, critical care service manager, ITU consultant physician<br>• Formal training in EBCD methods and on-going email and telephone support from staff experienced in using EBCD<br>• Access to and use of the online Kings Fund EBCD toolkit<br>*Barriers:*<br>• facilitator role time consuming and impacted significantly on time for clinical work (externally funded role for experienced ITU clinician at this site)<br>• learning about the AEBCD process whilst undertaking project organisational tasks and activities against a defined timeline	Not reported.<br><br>See Locock et al (above) for costs of facilitator salaries.	+
Tsianakas,V et al, 2012 Implementing patient-centred cancer care: using	Experience-based Co-Design (EBCD)	A large, inner-city cancer centre in	Ethnographic observation (219 hours)<br>Filmed narrative	*16 breast cancer working group outcomes including:*	*Barriers:*<br><br>Not specifically identified in the	Not evident or reported	++

Publication/ country	Form of co-production	Setting	Methods	Reported Outcomes	Barriers and facilitators	Health economic evaluation	Quality rating
experience-based co-design to improve patient experience in breast and lung cancer services. Supportive Care in Cancer (2012) 20:2639–2647<br><br>UK		England.<br><br>(Breast and lung cancer patients)	patient interviews (n=36)<br><br>Staff interviews (n= 63)<br><br>Post project interviews with<br><br>Staff (n=4)<br><br>and patients (n=5)	• Administrative staff receive customer-care training and are shown patients' DVD<br>• Healthcare assistants' interpersonal skills assessed prior to recruitment<br>• Managers and administrative staff use values-based performance tool which can improve patients' experience<br>• Changes to structure of clinics to reduce waiting times<br>• Patients receiving same chemotherapy treatment given option to receive information in a group<br><br>*11 lung cancer working group outcomes including:*<br><br>• Establishment of second breaking-bad-news room<br>• Guidance on diagnosis procedures included in junior doctors' induction<br>• Promotion of information and support centres at different sites (advertising at hospital entrance)<br>• Patient DVD 'welcome to cancer services' for newly diagnosed and	publication.<br><br>• As EBCD does not explicitly seek to recruit a representative sample of patients but those who are motivated to 'tell their story' and participate in the co-design work. This may lead to the experiences of 'harder to reach' patient groups remaining unheard.<br><br>*Facilitators:*<br><br>• Genuine and direct patient and carer involvement (relative to other service improvement projects in which patients and staff had participated).<br>• Patient participants taking direct responsibility for the work and its outcomes<br>• The EBCD change process itself together with the direct and active participation of staff and patients produced implementation and action, and contributed to spread and sustainability		

Publication/ country	Form of co-production	Setting	Methods	Reported Outcomes	Barriers and facilitators	Health economic evaluation	Quality rating
				referred patients			
Tsianakas,V., Robert ,G., Richardson,A., Verity,R., Oakley,C., Murrells,T., Flynn,M., Ream,E. Enhancing the experience of carers in the chemotherapy outpatient setting: an exploratory randomised controlled trial to test impact, acceptability and feasibility of a complex intervention co-designed by carers and staff . Support Care Cancer. 2015. DOI 10.1007/s00520-015-2677-x<br><br>UK	The study reports an RCT to test the impact and feasibility of a complex intervention (Take Care). The intervention was developed previously in the same hospital using a co-design approach – Experience-Based Co-Design.	Chemo-therapy day unit (breast, lung or colorectal cancer) in a London teaching hospital.	A two phase mixed methods design including i) an exploratory RCT (impact-measured through before and after postal questionnaire completion), ii) focus groups with carers and healthcare professionals (feasibility and acceptability)	• Carers in the intervention group had better understanding of the symptoms and side effects compared with the control group.<br>• Carers in the intervention group had more confidence in coping than the control group.<br>• There were no differences between the 2 groups for emotional well-being<br>• Health care professionals and carers confirmed the feasibility and acceptability of the EBCD derived Take Care intervention<br>• Patients and HCPs reported the intervention was educational, enhanced knowledge of symptoms and side effects, and increased confidence of carers.	Not reported in terms of EBCD<br><br>Reported limitations of the study included:<br>• Small sample size, no power calculation<br>• Exploratory trial in a single centre limits generalisability<br>• Self-report questionnaire responses may be subject to recall bias	Not evident or reported	+
Vennik et al, Co-production in healthcare:	Experience-based Co-Design (EBCD)	One haematology and four	Semi-structured interviews (n=27)	Improvement areas identified by patients were similar across hospitals. Apart from some specific	Barriers:<br>• Individual level- some physicians	Not evident or reported	++

For peer review only

Publication/ country	Form of co-production	Setting	Methods	Reported Outcomes	Barriers and facilitators	Health economic evaluation	Quality rating
rhetoric and practice. International Review of Administrative Sciences<br>2016, Vol. 82(1) 150–168<br>Netherlands	in 4 centres (Adapted)<br>Four project teams planned to use EBCD methodology; all adapted the method. Two used focus groups to gather patient experiences instead of filmed interviews. Two did not interview professionals. One did not use co-design workgroups concluding that it would require too much from participants; staff were not immediately convinced of the added value of this	oncology departments in 5 Dutch hospitals	Observations (70 hours) Documentary analysis	experiences with medical treatments, they mostly concerned aspects of quality of care including:<br>• Decoration of waiting rooms,<br>• Waiting times, -Communication, and coordination with general practitioners.<br>• Psychosocial care is now proactively offered during consultations at one hospital,<br>• A dental care in oncology leaflet was introduced at another hospital<br>Improvement issues identified were related to individual professions, hospital wards, the hospital organization and other healthcare providers.	afraid of being criticised by patients,<br>• Organisational barriers-pressure of work and other project commitments, changes in staffing levels, service re-organisations during the 12 months projects<br>• EBCD largely seen as 'additional work'<br>• Local policies and national trends can impact on engagement with co-production, no staff time freed for co-production/co-design, staff expected to deliver treatment and care and then engage in service improvement in their own time.<br>• Fee-for-service systems do not directly reward projects like EBCD/co-production<br>Facilitators:<br>• Motivated people who are willing to work on EBCD/co-production in their own time<br>• Selecting the 'right patients', inviting patients who have a critical point of view, are mentally and physically well enough condition to participate, and able to tell their story.<br>• It is not so much what patients say that is important; it is the process and the way they are given the opportunity to have their say (in EBCD/co-production) The active role of patients creates a sense of urgency		

Publication/ country	Form of co-production	Setting	Methods	Reported Outcomes	Barriers and facilitators	Health economic evaluation	Quality rating
	phase				to act on the improvement issues raised<br>• The process of co-production/EBCD stimulated staffs' thinking about how to realize quality improvements<br>• Keeping projects 'small', not attempting to improve everything at once and focusing<br>• Patients' input can gain legitimacy for improvement plans and facilitate implementation		

For peer review only

Supplementary file

Quality assessment records: What outcomes are associated with developing and implementing co-produced interventions in acute healthcare settings? A rapid evidence synthesis. (Clarke,D.J., Jones, F., Harris, R., Robert, G. 2017)

Checklist CREATE Rapid evidence synthesis. Quality appraisal tool. The full guidance can be accessed at:

https://www.nice.org.uk/article/pmg4/chapter/1%20introduction

**Study identification:** Include author, title, reference, year of publication	Bowen et al, How was it for you? Experiences of participatory design in the UK health service. CoDesign, 9:4, 230-246,	
**Guidance topic:**	**Key research question/aim:**<br>The aim of the project being evaluated was: ' a 12-month service improvement project focused on the medical outpatient service for older people,.<br>The aim (of this publication) was to explore how the project reveals issues of wider relevance to participatory health service design and to suggest general tactics for dealing with them.	
**Checklist completed by:**	DJC	
**Theoretical approach** Co-design using EBD located within a wider discussion of participatory approaches in healthcare and the NHS.		
**1. Is a qualitative approach appropriate?**<br>For example:<br>• Does the research question seek to understand processes or structures, or illuminate subjective experiences or meanings?<br>• Could a quantitative approach better have addressed the research question?	**Appropriate √**<br>Inappropriate<br>Not sure	Comments:<br>Appropriate method to address the main focus of the study which was on 'Experiences of participatory design in the UK health service' and used semi-structured interviews and a thematic approach to the analysis of the interview data.

For peer review only

<table>
<tr><td>2. Is the study clear in what it seeks to do?

For example:

• Is the purpose of the study discussed – aims/objectives/research question/s?

• Is there adequate/appropriate reference to the literature?

• Are underpinning values/assumptions/theory discussed?</td><td>Clear √

Unclear

Mixed</td><td>Comments:

Although lacks specific aims and research questions the purpose and focus of the evaluation work is clear.

`the work is located in the wider literature on participatory health service design in general and on the use of the EBD method specifically’. This refers to underlying assumption related to participation, i.e. that it should be more than consultation and go beyond surveys and directly involve users’ experiences in face to face co-design activity with staff.</td></tr>
<tr><td colspan="3">Study design</td></tr>
<tr><td>3. How defensible/rigorous is the research design/methodology?

For example:

• Is the design appropriate to the research question?

• Is a rationale given for using a qualitative approach?

• Are there clear accounts of the rationale/justification for the sampling, data collection and data analysis techniques used?

• Is the selection of cases/sampling strategy theoretically justified?</td><td>Defensible √

Indefensible

Not sure</td><td>Comments:

Appropriate means of data collection and analysis. Small sample size and no specific justification for the qualitative design.

The sample reflects the participants in the main EBD project but is not otherwise discussed or justified.</td></tr>
<tr><td colspan="3">Data collection</td></tr>
</table>

For peer review only

**4. How well was the data collection carried out?**<br><br>For example:<br>• Are the data collection methods clearly described?<br>• Were the appropriate data collected to address the research question?<br>• Was the data collection and record keeping systematic?	**Appropriately √**<br><br>Inappropriately<br><br>Not sure/inadequately reported	Comments:<br><br>Methods were clearly described, it was clear who undertook data collection and how. Some indication is provided of the focus of the questions asked of participants but no actual questions are presented in the paper.<br><br>No specific comments are made re record keeping although the description of data management as part of analysis is clear and appropriate.
**Trustworthiness**		
**5. Is the role of the researcher clearly described?**<br><br>For example:<br>• Has the relationship between the researcher and the participants been adequately considered?<br>• Does the paper describe how the research was explained and presented to the participants?	Clearly described<br><br>Unclear<br><br>**Not described √**	Comments:<br><br>Two researchers, who were not members of the original BOSOP project team, conducted the interviews.<br><br>The relationship between the researcher and the participants was not discussed.<br><br>How the research was explained and presented to the participants was not discussed.
**6. Is the context clearly described?**<br><br>For example:<br>• Are the characteristics of the participants and settings clearly defined?<br>• Were observations made in a sufficient variety of circumstances	**Clear √**<br><br>Unclear<br><br>Not sure	Comments:<br><br>Context bias was not considered. No observations were undertake, this was a small scale interview study.<br><br>The overall characteristics of the interview participants are outlined (e.g. patient or staff, if staff role type is stated) Notes who declined to participate and that a £10 voucher was given to participants.

For peer review only

• Was context bias considered		
**7. Were the methods reliable?**<br><br>For example:<br><br>• Was data collected by more than 1 method?<br><br>• Is there justification for triangulation, or for not triangulating?<br><br>• Do the methods investigate what they claim to?	**Reliable √**<br><br>Unreliable<br><br>Not sure	Comments:<br><br>Only interviews were used in this study (n=11). Data to evaluate the original project is described, there is no specific consideration of data triangulation. The interviews are clearly focused on participants' experiences and address the overall research aim.
**Analysis**		
**8. Is the data analysis sufficiently rigorous?**<br><br>For example:<br><br>• Is the procedure explicit – i.e. is it clear how the data was analysed to arrive at the results? (Yes)<br><br>• How systematic is the analysis, is the procedure reliable/dependable? (Yes)<br><br>• Is it clear how the themes and concepts were derived from the data? (yes)	**Rigorous √**<br><br>Not rigorous<br><br>Not sure/not reported	Comments: This is clearly outlined, is consistent with good practice in interview based qualitative research. The thematic analysis approach is clearly described, see across.
**9. Is the data 'rich'?**<br><br>For example:<br><br>• How well are the contexts of the data described?	**Rich √**<br><br>Poor<br><br>Not sure/not reported	Comments: A number of extracts from participants' interviews are reported and commented on in the discussion of the main themes emerging from the interview data analysis. Different views are explored although the sample size is limited so meaningful comparisons between participant views are vert limited.

For peer review only

• Has the diversity of perspective and content been explored?<br>• How well has the detail and depth been demonstrated?<br>• Are responses compared and contrasted across groups/sites?		
**10. Is the analysis reliable?**<br>For example:<br>• Did more than 1 researcher theme and code transcripts/data?<br>• If so, how were differences resolved?<br>• Did participants feed back on the transcripts/data if possible and relevant?<br>• Were negative/discrepant results addressed or ignored?	**Reliable √**<br>Unreliable<br>Not sure/not reported	Comments:<br>There appears to have been independent and joint researcher involvement in coding and thematic development. No participant feedback was sought on transcripts. As stated above the sample size was small so there appeared to be broad consensus amongst participants rather than negative/discrepant results, but 'negative' views were reported where they were identified.
**11. Are the findings convincing?**<br>For example:<br>• Are the findings clearly presented?<br>• Are the findings internally coherent?<br>• Are extracts from the original data included?<br>• Are the data appropriately referenced?<br>• Is the reporting clear and coherent?	**Convincing √**<br>Not convincing<br>Not sure	Comments:<br>Five themes were identified and discussed clearly and coherently. Original data extracts were included. The discussion section refers to linked and relevant literature in the field.

For peer review only

**12. Are the findings relevant to the aims of the study?**	**Relevant √**<br><br>Irrelevant<br><br>Partially relevant	Comments: Focus directly on participants' experiences of the involvement in the EBD project.
**13. Conclusions**<br><br>For example:<br><br>• How clear are the links between data, interpretation and conclusions?<br><br>• Are the conclusions plausible and coherent?<br><br>• Have alternative explanations been explored and discounted?<br><br>• Does this enhance understanding of the research topic?<br><br>• Are the implications of the research clearly defined?<br><br>**Is there adequate discussion of any limitations encountered?**	**Adequate √**<br><br>Inadequate<br><br>Not sure	Comments:<br><br>Conclusions are clear, plausible and directly related to evaluation of the experience of the EBD method. The findings appear to have been contributory to further development of the EBD method which became EBCD though informing the Kings Fund resources/tool kit.<br><br>Limitations of the methods and the study were clearly identified in the discussion section and their impact of the findings considered.
**Ethics**		
**14. How clear and coherent is the reporting of ethics?**<br><br>For example:<br><br>• Have ethical issues been taken into consideration?<br><br>• Are they adequately discussed e.g. do they	**Appropriate √**<br><br>Inappropriate<br><br>Not sure/not reported	Comments: Clear that ethical approval was obtained nut ethical issues were not discussed in the report other than that consent was obtained.

For peer review only

address consent and anonymity?<br>• Have the consequences of the research been considered i.e. raising expectations, changing behaviour?<br>• Was the study approved by an ethics committee?		
**Overall assessment**		
**As far as can be ascertained from the paper, how well was the study conducted? (see guidance notes)**	**++**<br>+<br>−	Comments: Small descriptive study but clearly presented overall with core areas of the research e.g. data collection and analysis clearly reported and appropriate to the study focus. This statement applies: *'All or most of the checklist criteria have been fulfilled, where they have not been fulfilled the conclusions are very unlikely to alter'*.

**Study identification:** Include author, title, reference, year of publication	Bowen et al, How was it for you? Experiences of participatory design in the UK health service. CoDesign, 9:4, 230-246,
**Guidance topic:** Co-production in acute healthcare settings.	**Key research question/aim:** The authors were concerned that there had been only limited tangible service improvement at the end of the project BOSOP project so they designed a post-project evaluation study to test their previous hypotheses about why this might be the case against the viewpoints of other stakeholders within the EBD project and to refine understanding.
**Checklist completed by:**	RH
**Theoretical approach:** No specific theoretical framework was reported	

For peer review only

**1. Is a qualitative approach appropriate?**<br>For example:<br>• Does the research question seek to understand processes or structures, or illuminate subjective experiences or meanings?<br>• Could a quantitative approach better have addressed the research question?	Appropriate ✓<br>Inappropriate<br>Not sure	Comments:
**2. Is the study clear in what it seeks to do?**<br>For example:<br>• Is the purpose of the study discussed – aims/objectives/research question/s?<br>• Is there adequate/appropriate reference to the literature?<br>• Are underpinning values/assumptions/theory discussed?	Clear ✓<br>Unclear<br>Mixed	Comments:
**Study design**		
**3. How defensible/rigorous is the research design/methodology?**<br>For example:<br>• Is the design appropriate to the research question?<br>• Is a rationale given for using a qualitative approach?<br>• Are there clear accounts of the rationale/justification for the sampling, data collection and data analysis techniques	Defensible ✓<br>Indefensible<br>Not sure	Comments:

For peer review only

used?<br>• Is the selection of cases/sampling strategy theoretically justified?		
**Data collection**		
**4. How well was the data collection carried out?**<br>For example:<br>• Are the data collection methods clearly described?<br>• Were the appropriate data collected to address the research question?<br>• Was the data collection and record keeping systematic?	Appropriately ✓<br>Inappropriately<br>Not sure/inadequately reported	Comments:
**Trustworthiness**		

For peer review only

**5. Is the role of the researcher clearly described?**<br>For example:<br>• Has the relationship between the researcher and the participants been adequately considered?<br>• Does the paper describe how the research was explained and presented to the participants?	Clearly described ✓<br>Unclear<br>Not described	Comments:
**6. Is the context clearly described?**<br>For example:<br>• Are the characteristics of the participants and settings clearly defined?<br>• Were observations made in a sufficient variety of circumstances<br>• Was context bias considered	Clear ✓<br>Unclear<br>Not sure	Comments:
**7. Were the methods reliable?**<br>For example:<br>• Was data collected by more than 1 method?<br>• Is there justification for triangulation, or for not triangulating?	Reliable ✓<br>Unreliable<br>Not sure	Comments:

For peer review only

• Do the methods investigate what they claim to?		
**Analysis**		
**8. Is the data analysis sufficiently rigorous?**<br>For example:<br>• Is the procedure explicit – i.e. is it clear how the data was analysed to arrive at the results?<br>• How systematic is the analysis, is the procedure reliable/dependable?<br>• Is it clear how the themes and concepts were derived from the data?	Rigorous<br>Not rigorous ✓<br>Not sure/not reported	Comments:
**9. Is the data 'rich'?**<br>For example:<br>• How well are the contexts of the data described?<br>• Has the diversity of perspective and content been explored?<br>• How well has the detail and depth been demonstrated?<br>• Are responses compared and contrasted across groups/sites?	Rich ✓<br>Poor<br>Not sure/not reported	Comments:
**10. Is the analysis reliable?**	Reliable ✓	Comments:

For peer review only

For example:<br><br>• Did more than 1 researcher theme and code transcripts/data?<br><br>• If so, how were differences resolved?<br><br>• Did participants feed back on the transcripts/data if possible and relevant?<br><br>• Were negative/discrepant results addressed or ignored?	Unreliable<br><br>Not sure/not reported	
**11. Are the findings convincing?**<br><br>For example:<br><br>• Are the findings clearly presented?<br><br>• Are the findings internally coherent?<br><br>• Are extracts from the original data included?<br><br>• Are the data appropriately referenced?<br><br>• Is the reporting clear and coherent?	Convincing ✓<br><br>Not convincing<br><br>Not sure	Comments:
**12. Are the findings relevant to the aims of the study?**	Relevant ✓<br><br>Irrelevant<br><br>Partially relevant	Comments:
**13. Conclusions**<br><br>For example:<br><br>• How clear are the links between data, interpretation and	Adequate ✓<br><br>Inadequate<br><br>Not sure	Comments:

For peer review only

conclusions?<br>• Are the conclusions plausible and coherent?<br>• Have alternative explanations been explored and discounted?<br>• Does this enhance understanding of the research topic?<br>• Are the implications of the research clearly defined?<br>**Is there adequate discussion of any limitations encountered?**		
**Ethics**		
**14. How clear and coherent is the reporting of ethics?**<br>For example:<br>• Have ethical issues been taken into consideration?<br>• Are they adequately discussed e.g. do they address consent and anonymity?<br>• Have the consequences of the research been considered i.e. raising expectations, changing behaviour?<br>• Was the study approved by an ethics committee?	Appropriate ✓<br>Inappropriate<br>Not sure/not reported	Comments:
**Overall assessment**		
**As far as can be ascertained from the paper, how well was the study conducted? (see guidance notes)**	++ ✓	Comments:

For peer review only

	+<br>-	

**Study identification:** Include author, title, reference, year of publication	Boyd et al, Improving healthcare through the use of co-design. The New Zealand Medical Journal, 125,76–87.
**Guidance topic:**	**Key research question/aim:** No research questions. Aims stated only as follows: A Patient Co-Design of Breast Service Project was set up to improve referral processes and develop clinical guidelines for patients with breast cancer. It aimed to use co-design methods to understand patients experiences, make small services changes (not defined), make recommendations for further service change and develop a model for working with patients that could be used in other services. It also sought to work with patients in a new and more participatory way .
**Checklist completed by:**	DJC

Theoretical approach Locates the service improvement project in the broad area of co-design (with reference to co-production) and describes a project using EBD based methods but with six elements as opposed to the four seen for example in Bowen et al. Describes a clear commitment to the equity (in participating in service improvement based on experiences of patients), and understanding and improving patient experiences.

**1. Is a qualitative approach appropriate?**<br><br>For example:<br><br>• Does the research question seek to understand processes or structures, or illuminate subjective experiences or meanings?<br><br>• Could a quantitative approach better have	**Appropriate √**<br><br>Inappropriate<br><br>Not sure	Comments: Qualitative methods are used as part of the service improvement approach, rather than as research methods.<br><br>A simple quantitative survey tool was also employed.

For peer review only

addressed the research question?		
**2. Is the study clear in what it seeks to do?**<br>For example:<br>• Is the purpose of the study discussed – aims/objectives/research question/s?<br>• Is there adequate/appropriate reference to the literature?<br>• Are underpinning values/assumptions/theory discussed?	**Clear √**<br>Unclear<br>Mixed	Comments: Aims are only broadly outlined but the intentions of the project and desired impact on services to improve patient experiences were clear.
**Study design**		
**3. How defensible/rigorous is the research design/methodology?**<br>For example:<br>• Is the design appropriate to the research question?<br>• Is a rationale given for using a qualitative approach?<br>• Are there clear accounts of the rationale/justification for the sampling, data collection and data analysis techniques used?<br>• Is the selection of cases/sampling strategy theoretically justified?	**Defensible √**<br>Indefensible<br>Not sure	Comments: In the sense that this was a structured service improvement approach based on an established approach EBD. No research questions were developed as such but there were questions driving the co-design workshops focused on patient experience.<br>There is no justification for sampling as such but there is for the patient journey mapping and the co-design workshops.

For peer review only

Data collection		
**4. How well was the data collection carried out?**<br><br>For example:<br><br>• Are the data collection methods clearly described?<br><br>• Were the appropriate data collected to address the research question?<br><br>• Was the data collection and record keeping systematic?	Appropriately<br><br>Inappropriately<br><br>Not sure/**inadequately reported √**	Comments: The methods were appropriate and relevant to the intended project outcomes but were reported in only a limited way.
**Trustworthiness**		
**5. Is the role of the researcher clearly described?**<br><br>For example:<br><br>• Has the relationship between the researcher and the participants been adequately considered?<br><br>• Does the paper describe how the research was explained and presented to the participants?	Clearly described<br><br>Unclear<br><br>**Not described √**	Comments: The project as a whole is described but this refers to a number of facilitators and not to a single or small group of researchers as such.
**6. Is the context clearly described?**<br><br>For example:<br><br>• Are the characteristics of the participants and	Clear<br><br>Unclear<br><br>**Not sure √**	Comments: There is again, broad overview of the services in a particular health district. The ages of women attending, the backgrounds, referral systems, treatment times etc are not referred to.

For peer review only

settings clearly defined?<br>• Were observations made in a sufficient variety of circumstances<br>• Was context bias considered		
**7. Were the methods reliable?**<br>For example:<br>• Was data collected by more than 1 method?<br>• Is there justification for triangulation, or for not triangulating?<br>• Do the methods investigate what they claim to?	**Reliable √**<br>Unreliable<br>Not sure	Comments: Adequate description of methods appropriate to address the aims of the project.
**Analysis**		
**8. Is the data analysis sufficiently rigorous?**<br>For example:<br>• Is the procedure explicit – i.e. is it clear how the data was analysed to arrive at the results?<br>• How systematic is the analysis, is the procedure reliable/dependable?<br>• Is it clear how the themes and concepts were derived from the data?	Rigorous<br>Not rigorous<br>Not sure**/not reported √**	Comments: no comment on this important issue.

For peer review only

**9. Is the data 'rich'?**<br><br>For example:<br><br>• How well are the contexts of the data described?<br><br>• Has the diversity of perspective and content been explored?<br><br>• How well has the detail and depth been demonstrated?<br><br>• Are responses compared and contrasted across groups/sites?	Rich<br><br>Poor<br><br>Not sure/**not reported fully √**	Comments: There is descriptive summary in each of the three areas of data collected but no illustrative direct quotations from participants or staff.
**10. Is the analysis reliable?**<br><br>For example:<br><br>• Did more than 1 researcher theme and code transcripts/data?<br><br>• If so, how were differences resolved?<br><br>• Did participants feed back on the transcripts/data if possible and relevant?<br><br>• Were negative/discrepant results addressed or ignored?	Reliable<br><br>Unreliable<br><br>Not sure/**not reported √**	Comments: As above, no comment.
**11. Are the findings convincing?**<br><br>For example:<br><br>• Are the findings clearly presented?	**Convincing √**<br><br>Not convincing<br><br>Not sure	Comments: Accepting the limitations in reporting, the findings are convincing.

• Are the findings internally coherent?<br>• Are extracts from the original data included?<br>• Are the data appropriately referenced?<br>• Is the reporting clear and coherent?		
**12. Are the findings relevant to the aims of the study?**	**Relevant √**<br>Irrelevant<br>Partially relevant	Comments:
**13. Conclusions**<br>For example:<br>• How clear are the links between data, interpretation and conclusions?<br>• Are the conclusions plausible and coherent?<br>• Have alternative explanations been explored and discounted?<br>• Does this enhance understanding of the research topic?<br>• Are the implications of the research clearly defined?<br>**Is there adequate discussion of any limitations encountered?**	Adequate<br>**Inadequate √**<br>Not sure	Comments: There is a discussion section with recommendations but no conclusion section as such. The discussion does add to this specific literature (limited as it is) and there are indications for further research. The limitations of the study itself are acknowledged, i.e no evaluation and no follow up to determine whether planned improvements had been implemented and sustained.
**Ethics**		

For peer review only

**14. How clear and coherent is the reporting of ethics?**<br><br>For example:<br><br>• Have ethical issues been taken into consideration?<br><br>• Are they adequately discussed e.g. do they address consent and anonymity?<br><br>• Have the consequences of the research been considered i.e. raising expectations, changing behaviour?<br><br>• Was the study approved by an ethics committee?	Appropriate<br><br>Inappropriate<br><br>Not sure/**not reported √**	Comments: No indication that this was required if this was regarded as a service improvement project.
**Overall assessment**		
**As far as can be ascertained from the paper, how well was the study conducted? (see guidance notes)**	++<br><br>**+ √**<br><br>−	Comments: A very limited report; largely a service improvement report.

For peer review only

**Study identification:** Include author, title, reference, year of publication	Boyd et al, Improving healthcare through the use of co-design. The New Zealand Medical Journal, 125,76–87.
**Guidance topic:**	**Key research question/aim:** No research questions. Aims: A Patient Co-Design of Breast Service Project was set up to improve referral processes and develop clinical guidelines for patients with breast cancer. It aimed to use co-design methods to understand patients' experiences, make small services changes, make recommendations for further service change and develop a model for working with patients that could be

	used in other services. It also sought to work with patients in a new and more participatory way.	
**Checklist completed by:**	FJ	
**Theoretical approach** Locates the service improvement project in the broad area of co-design (with reference to co-production) and describes a project using EBD based methods but with six elements as opposed to the four seen for example in Bowen et al.		
**1. Is a qualitative approach appropriate?**<br>For example:<br>• Does the research question seek to understand processes or structures, or illuminate subjective experiences or meanings?<br>• Could a quantitative approach better have addressed the research question?	Appropriate √<br>Inappropriate<br>Not sure	Comments:
**2. Is the study clear in what it seeks to do?**<br>For example:<br>• Is the purpose of the study discussed – aims/objectives/research question/s?<br>• Is there adequate/appropriate reference to the literature?<br>• Are underpinning values/assumptions/theory discussed?	Clear √<br>Unclear<br>Mixed	Comments:

For peer review only

**Study design**		
**3. How defensible/rigorous is the research design/methodology?**<br><br>For example:<br><br>• Is the design appropriate to the research question?<br><br>• Is a rationale given for using a qualitative approach?<br><br>• Are there clear accounts of the rationale/justification for the sampling, data collection and data analysis techniques used?<br><br>• Is the selection of cases/sampling strategy theoretically justified?	Defensible **√**<br><br>Indefensible<br><br>Not sure	Comments:
**Data collection**		
**4. How well was the data collection carried out?**<br><br>For example:<br><br>• Are the data collection methods clearly described?<br><br>• Were the appropriate data collected to address the research question?<br><br>• Was the data collection and record keeping systematic?	Appropriately<br><br>Inappropriately<br><br>Not sure/inadequately reported **√**	Comments:

For peer review only

**Trustworthiness**		
**5. Is the role of the researcher clearly described?**<br><br>For example:<br><br>• Has the relationship between the researcher and the participants been adequately considered?<br><br>• Does the paper describe how the research was explained and presented to the participants?	Clearly described<br><br>Unclear<br><br>Not described **√**	Comments:
**6. Is the context clearly described?**<br><br>For example:<br><br>• Are the characteristics of the participants and settings clearly defined?<br><br>• Were observations made in a sufficient variety of circumstances<br><br>• Was context bias considered	Clear<br><br>Unclear<br><br>Not sure **√**	Comments:
**7. Were the methods reliable?**<br><br>For example:<br><br>• Was data collected by more than 1 method?<br><br>• Is there justification for triangulation, or for not triangulating?<br><br>• Do the methods investigate what they claim	Reliable **√**<br><br>Unreliable<br><br>Not sure	Comments:

For peer review only

to?		
**Analysis**		
**8. Is the data analysis sufficiently rigorous?**<br><br>For example:<br><br>• Is the procedure explicit – i.e. is it clear how the data was analysed to arrive at the results?<br><br>• How systematic is the analysis, is the procedure reliable/dependable?<br><br>• Is it clear how the themes and concepts were derived from the data?	Rigorous<br><br>Not rigorous<br><br>Not sure/not reported √	Comments:
**9. Is the data 'rich'?**<br><br>For example:<br><br>• How well are the contexts of the data described?<br><br>• Has the diversity of perspective and content been explored?<br><br>• How well has the detail and depth been demonstrated?<br><br>• Are responses compared and contrasted across groups/sites?	Rich<br><br>Poor<br><br>Not sure/not reported fully √	Comments:
**10. Is the analysis reliable?**	Reliable	Comments:

For peer review only

For example:<br>• Did more than 1 researcher theme and code transcripts/data?<br>• If so, how were differences resolved?<br>• Did participants feed back on the transcripts/data if possible and relevant?<br>• Were negative/discrepant results addressed or ignored?	Unreliable<br>Not sure/not reported √	
**11. Are the findings convincing?**<br>For example:<br>• Are the findings clearly presented?<br>• Are the findings internally coherent?<br>• Are extracts from the original data included?<br>• Are the data appropriately referenced?<br>• Is the reporting clear and coherent?	Convincing √<br>Not convincing<br>Not sure	Comments:
**12. Are the findings relevant to the aims of the study?**	Relevant √<br>Irrelevant<br>Partially relevant	Comments:
**13. Conclusions**<br>For example:	Adequate<br>Inadequate √	Comments:

For peer review only

• How clear are the links between data, interpretation and conclusions?<br>• Are the conclusions plausible and coherent?<br>• Have alternative explanations been explored and discounted?<br>• Does this enhance understanding of the research topic?<br>• Are the implications of the research clearly defined?<br>**Is there adequate discussion of any limitations encountered?**	Not sure	
**Ethics**		
**14. How clear and coherent is the reporting of ethics?**<br>For example:<br>• Have ethical issues been taken into consideration?<br>• Are they adequately discussed e.g. do they address consent and anonymity?<br>• Have the consequences of the research been considered i.e. raising expectations, changing behaviour?<br>• Was the study approved by an ethics committee?	Appropriate<br>Inappropriate<br>Not sure/not reported **√**	Comments:

For peer review only

**Overall assessment**		
**As far as can be ascertained from the paper, how well was the study conducted? (see guidance notes)**	++<br>**+ √**<br>−	Comments: The methods were appropriate and relevant to the intended project outcomes. The paper is limited by omissions in a number of the areas indicated above, principally, little information on data analysis and the reporting of findings.

**Study identification:** Include author, title, reference, year of publication	Golden.B.R. et al, 2011 Improving the patient experience through design. Healthcare Quarterly. 14(3)32-41
**Guidance topic:** Co-production in acute healthcare settings.	**Key research question/aim:** PMH's vision was to create a new space and experience that was truly patient centric and world class. (P32) No specific aims and objectives were stated.
**Checklist completed by:**	FJ
**Theoretical approach:**<br><br>Reference to ethnography and 'Rotman Creative design process	

**1. Is a qualitative approach appropriate?**<br><br>For example:<br><br>• Does the research question seek to understand processes or structures, or illuminate subjective experiences or meanings?<br><br>• Could a quantitative approach better have	Appropriate ✓<br><br>Inappropriate<br><br>Not sure	Comments:<br><br>The study report different qualitative methods used to inform, develop and explore service experience in an outpatient cancer setting. Its is not a qualitative study as such.

For peer review only

addressed the research question?		
**2. Is the study clear in what it seeks to do?**<br>For example:<br>• Is the purpose of the study discussed – aims/objectives/research question/s?<br>• Is there adequate/appropriate reference to the literature?<br>• Are underpinning values/assumptions/theory discussed?	Clear<br>Unclear<br>Mixed ✓	Comments:<br>There are no aims but the purpose is clear to improve the experience of patients. Minimal reference to relevant literature, reads more like a commentary/report than reporting the findings from a distinct study.
**Study design**		
**3. How defensible/rigorous is the research design/methodology?**<br>For example:<br>• Is the design appropriate to the research question?<br>• Is a rationale given for using a qualitative approach?<br>• Are there clear accounts of the rationale/justification for the sampling, data collection and data analysis techniques used?<br>• Is the selection of cases/sampling strategy	Defensible<br>Indefensible<br>Not sure ✓	Comments:<br>The paper reports the 3 stages of Rotman Creative design, the first 2 stages include multiple methods. But these are not clearly linked to any research question. Many of the data collection methods involved researcher led activities, which followed a series of stages without full rationale and justification.<br>There was no evidence of sampling and inadequate detail about cases included.

For peer review only

theoretically justified?		
**Data collection**		
**4. How well was the data collection carried out?**<br>For example:<br>• Are the data collection methods clearly described?<br>• Were the appropriate data collected to address the research question?<br>• Was the data collection and record keeping systematic?	Appropriately<br>Inappropriately<br>Not sure/<br>inadequately reported ✓	Comments:<br>Data collection methods briefly described but not in a systematic or logical way. No detail about how data was extracted, recorded etc
**Trustworthiness**		

For peer review only

**5. Is the role of the researcher clearly described?**<br>For example:<br>• Has the relationship between the researcher and the participants been adequately considered?<br>• Does the paper describe how the research was explained and presented to the participants?	Clearly described<br>Unclear<br>Not described ✓	Comments:
**6. Is the context clearly described?**<br>For example:<br>• Are the characteristics of the participants and settings clearly defined?<br>• Were observations made in a sufficient variety of circumstances<br>• Was context bias considered	Clear ✓<br>Unclear<br>Not sure	Comments:<br>There were 'patient personas' described which were supposed to represent patients using the services, no other details<br>The setting was described but not in any great detail, more to illustrate particular problems, issues.
**7. Were the methods reliable?**<br>For example:<br>• Was data collected by more than 1 method?<br>• Is there justification for triangulation, or for not triangulating?	Reliable<br>Unreliable<br>Not sure ✓	Comments:<br>Not reported; could be triangulation as more than one method was used but this was not reported as such.

For peer review only

• Do the methods investigate what they claim to?		
**Analysis**		
**8. Is the data analysis sufficiently rigorous?**<br>For example:<br>• Is the procedure explicit – i.e. is it clear how the data was analysed to arrive at the results?<br>• How systematic is the analysis, is the procedure reliable/dependable?<br>• Is it clear how the themes and concepts were derived from the data?	Rigorous<br>Not rigorous<br>Not sure/ ✓	Comments:<br>Inconsistent and inadequate reporting of data analysis methods<br>For example the interview data was subjected to line by line coding and then synthesised into a mind map. The method of analysis and approach used was not stated.
**9. Is the data 'rich'?**<br>For example:<br>• How well are the contexts of the data described?<br>• Has the diversity of perspective and content been explored?<br>• How well has the detail and depth been demonstrated?<br>• Are responses compared and contrasted	Rich<br>Poor<br>Not sure✓	Comments:<br>Quotes were given from patients involved in photo journaling; need mind map illustrated the diversity and context of the project. But the findings weren't presented systematically and preferential reporting of certain experiences/comments couldn't be ruled out. The diversity of perspective presented by authors is hard to appraise.

For peer review only

across groups/sites?		
**10. Is the analysis reliable?**<br>For example:<br>• Did more than 1 researcher theme and code transcripts/data?<br>• If so, how were differences resolved?<br>• Did participants feed back on the transcripts/data if possible and relevant?<br>• Were negative/discrepant results addressed or ignored?	Reliable<br>Unreliable<br>Not sure/not reported ✓	Comments:
**11. Are the findings convincing?**<br>For example:<br>• Are the findings clearly presented?<br>• Are the findings internally coherent?<br>• Are extracts from the original data included?<br>• Are the data appropriately referenced?<br>• Is the reporting clear and coherent?	Convincing<br>Not convincing<br>Not sure ✓	Comments: The data findings were not consistently reported. There was more detail about some stages than other in sections. Some extracts from original data included, but this was also inconsistent.
**12. Are the findings relevant to the aims of the study?**	Relevant ✓<br>Irrelevant	Comments:

For peer review only

	Partially relevant	
**13. Conclusions**<br><br>For example:<br><br>• How clear are the links between data, interpretation and conclusions?<br><br>• Are the conclusions plausible and coherent?<br><br>• Have alternative explanations been explored and discounted?<br><br>• Does this enhance understanding of the research topic?<br><br>• Are the implications of the research clearly defined?<br><br>**Is there adequate discussion of any limitations encountered?**	Adequate<br><br>Inadequate<br><br>Not sure ✓	Comments:<br><br>The aims were not fully stated therefore it is not clear if the conclusions are accurate.<br><br>The paper provides an in-depth description of a staged design approach but the lack of clarity and way in which the findings are reported makes it difficult to confirm if the findings are plausible
**Ethics**		
**14. How clear and coherent is the reporting of ethics?**<br><br>For example:<br><br>• Have ethical issues been taken into consideration?<br><br>• Are they adequately discussed e.g. do they	Appropriate<br><br>Inappropriate<br><br>Not sure/not reported ✓	Comments:<br><br>Not reported

For peer review only

address consent and anonymity?<br>• Have the consequences of the research been considered i.e. raising expectations, changing behaviour?<br>• Was the study approved by an ethics committee?		
**Overall assessment**		
**As far as can be ascertained from the paper, how well was the study conducted? (see guidance notes)**	++<br>+ ✓<br>-	Comments:

**Study identification:** Include author, title, reference, year of publication	Golden.B.R. et al, 2011 Improving the patient experience through design. Healthcare Quarterly. 14(3)32-41
**Guidance topic:** Co-production in acute healthcare settings.	**Key research question/aim:** PMH's vision was to create a new space and experience that was truly patient centric and world class. (P32) No specific aims and objectives were stated.
**Checklist completed by:**	RH
**Theoretical approach:** No specific theoretical framework was reportedly used although the paper does refer to ethnography. The Rotman Creative Design process was used. This involved 3 main activities: Gear 1. empathy and deep human understanding; Gear 2. Concept visualisation; Gear 3. Strategic business design.	

For peer review only

**1. Is a qualitative approach appropriate?**<br><br>For example:<br><br>• Does the research question seek to understand processes or structures, or illuminate subjective experiences or meanings?<br><br>• Could a quantitative approach better have addressed the research question?	Appropriate ✓<br><br>Inappropriate<br><br>Not sure	Comments: The paper does not report a research study as such. It reports the first stages of a co-design process but the later stages where a specific intervention is designed and evaluated is not reported.
**2. Is the study clear in what it seeks to do?**<br><br>For example:<br><br>• Is the purpose of the study discussed – aims/objectives/research question/s?<br><br>• Is there adequate/appropriate reference to the literature?<br><br>• Are underpinning values/assumptions/theory discussed?	Clear<br><br>Unclear<br><br>Mixed ✓	Comments: The aims are not specifically stated although from reading the paper it is clear what they are attempting to do. The researchers do cite literature about the process of co-design they are using but the study feels half finished!
**Study design**		
**3. How defensible/rigorous is the research design/methodology?**<br><br>For example:<br><br>• Is the design appropriate to the research question?	Defensible<br><br>Indefensible<br><br>Not sure ✓	Comments: The approach used is defensible however, there is a lack of details about how participants were selected, no. of participants / observation sessions etc.

For peer review only

• Is a rationale given for using a qualitative approach?<br>• Are there clear accounts of the rationale/justification for the sampling, data collection and data analysis techniques used?<br>• Is the selection of cases/sampling strategy theoretically justified?		
**Data collection**		
**4. How well was the data collection carried out?**<br>For example:<br>• Are the data collection methods clearly described?<br>• Were the appropriate data collected to address the research question?<br>• Was the data collection and record keeping systematic?	Appropriately<br>Inappropriately<br>Not sure/**inadequately reported** ✓	Comments: There was quite a few methods used and how these worked together is not reported. There is a lack of detail about how individual methods as well.
**Trustworthiness**		

For peer review only

**5. Is the role of the researcher clearly described?**<br>For example:<br>• Has the relationship between the researcher and the participants been adequately considered?<br>• Does the paper describe how the research was explained and presented to the participants?	Clearly described<br>Unclear<br>Not described ✓	Comments:
**6. Is the context clearly described?**<br>For example:<br>• Are the characteristics of the participants and settings clearly defined?<br>• Were observations made in a sufficient variety of circumstances<br>• Was context bias considered	Clear ✓<br>Unclear<br>Not sure	Comments: Characteristics of some patients were reported in detail but not all, particularly carers. Observations were made in a variety of circumstances although context bias was not considered in the paper.
**7. Were the methods reliable?**<br>For example:<br>• Was data collected by more than 1 method?<br>• Is there justification for triangulation, or for not triangulating?	Reliable ✓<br>Unreliable<br>Not sure	Comments: Not reported as such although there were data from a range of methods.

For peer review only

• Do the methods investigate what they claim to?		
**Analysis**		
**8. Is the data analysis sufficiently rigorous?**<br>For example:<br>• Is the procedure explicit – i.e. is it clear how the data was analysed to arrive at the results?<br>• How systematic is the analysis, is the procedure reliable/dependable?<br>• Is it clear how the themes and concepts were derived from the data?	Rigorous<br>Not rigorous<br>Not sure/**not reported** ✓	Comments: Analysis not reported in sufficient detail
**9. Is the data 'rich'?**<br>For example:<br>• How well are the contexts of the data described?<br>• Has the diversity of perspective and content been explored?<br>• How well has the detail and depth been demonstrated?<br>• Are responses compared and contrasted across groups/sites?	Rich ✓<br>Poor<br>Not sure/not reported	Comments: The data as reported is rich although whether data analysis is systematic and comprehensive is not reported.

For peer review only

**10. Is the analysis reliable?**<br>For example:<br>• Did more than 1 researcher theme and code transcripts/data?<br>• If so, how were differences resolved?<br>• Did participants feed back on the transcripts/data if possible and relevant?<br>• Were negative/discrepant results addressed or ignored?	Reliable<br>Unreliable<br>Not sure/not reported ✓	Comments: How data were analysed is not reported.
**11. Are the findings convincing?**<br>For example:<br>• Are the findings clearly presented?<br>• Are the findings internally coherent?<br>• Are extracts from the original data included?<br>• Are the data appropriately referenced?<br>• Is the reporting clear and coherent?	Convincing ✓<br>Not convincing<br>Not sure	Comments: This findings were quite compelling although not presented in a form that was easy to follow.
**12. Are the findings relevant to the aims of the study?**	Relevant ✓<br>Irrelevant<br>Partially relevant	Comments: although the study is not complete – the design and implementation of an intervention is not reported.

For peer review only

**13. Conclusions**<br>For example:<br>• How clear are the links between data, interpretation and conclusions?<br>• Are the conclusions plausible and coherent?<br>• Have alternative explanations been explored and discounted?<br>• Does this enhance understanding of the research topic?<br>• Are the implications of the research clearly defined?<br>**Is there adequate discussion of any limitations encountered?**	Adequate<br>Inadequate<br>Not sure ✓	Comments: The aims are not fulfilled as reported in this paper as the intervention developed is not reported. Feels unfinished. There is useful, interesting findings though. And the value of the co-design approach is demonstrated.
**Ethics**		
**14. How clear and coherent is the reporting of ethics?**<br>For example:<br>• Have ethical issues been taken into consideration?<br>• Are they adequately discussed e.g. do they address consent and anonymity?<br>• Have the consequences of the research been	Appropriate<br>Inappropriate<br>Not sure/not reported ✓	Comments: Not addressed in the paper

For peer review only

considered i.e. raising expectations, changing behaviour?<br>• Was the study approved by an ethics committee?		
**Overall assessment**		
**As far as can be ascertained from the paper, how well was the study conducted? (see guidance notes)**	++<br>+ ✓<br>–	Comments:

**Study identification:** Include author, title, reference, year of publication	Larkin, M et al, 2015 On the Brink of Genuinely Collaborative Care: Experience-Based Co-Design in Mental Health. Qualitative Health Research2015, Vol. 25(11) 1463–1476
**Guidance topic:** Co-production in acute healthcare settings.	**Key research question/aim:**<br>Can 3 qualitative studies and an adapted form of EBCD translate into service improvements in acute mental health services
**Checklist completed by:**	FJ
**Theoretical approach: evaluation of existing EBCD improvement projects; no other details about theoretical approach**	

**1. Is a qualitative approach appropriate?**	Appropriate ✓	Comments: interviews used to inform co-design process

For peer review only

For example:<br>• Does the research question seek to understand processes or structures, or illuminate subjective experiences or meanings?<br>• Could a quantitative approach better have addressed the research question?	Inappropriate<br>Not sure	
**2. Is the study clear in what it seeks to do?**<br>For example:<br>• Is the purpose of the study discussed – aims/objectives/research question/s?<br>• Is there adequate/appropriate reference to the literature?<br>• Are underpinning values/assumptions/theory discussed?	Clear ✓<br>Unclear<br>Mixed	Comments:
**Study design**		
**3. How defensible/rigorous is the research design/methodology?**<br>For example:<br>• Is the design appropriate to the research question?<br>• Is a rationale given for using a qualitative	Defensible ✓<br>Indefensible<br>Not sure	Comments:

For peer review only

approach?<br>• Are there clear accounts of the rationale/justification for the sampling, data collection and data analysis techniques used?<br>• Is the selection of cases/sampling strategy theoretically justified?		
**Data collection**		
**4. How well was the data collection carried out?**<br>For example:<br>• Are the data collection methods clearly described?<br>• Were the appropriate data collected to address the research question?<br>• Was the data collection and record keeping systematic?	Appropriately ✓<br>Inappropriately<br>Not sure/inadequately reported	Comments: full findings of interview analyses are reported elsewhere
**Trustworthiness**		

For peer review only

**5. Is the role of the researcher clearly described?**<br>For example:<br>• Has the relationship between the researcher and the participants been adequately considered?<br>• Does the paper describe how the research was explained and presented to the participants?	Clearly described<br>Unclear ✓<br>Not described	Comments: role and activities of researcher not explicitly stated but inferred from descriptions of researchers involvement in co-design process
**6. Is the context clearly described?**<br>For example:<br>• Are the characteristics of the participants and settings clearly defined?<br>• Were observations made in a sufficient variety of circumstances<br>• Was context bias considered	Clear<br>Unclear ✓<br>Not sure	Comments: no comment on content bias, no observations done; characteristics of participants not described (? this is due to the fact that they are reported in another publication)
**7. Were the methods reliable?**<br>For example:<br>• Was data collected by more than 1 method?<br>• Is there justification for triangulation, or for not triangulating?	Reliable ✓<br>Unreliable<br>Not sure	Comments: interviews only but triangulated through feedback groups and co-design groups

For peer review only - http://bmjopen.bmj.com/site/about/guidelines.xhtml

• Do the methods investigate what they claim to?		
**Analysis**		
**8. Is the data analysis sufficiently rigorous?**<br>For example:<br>• Is the procedure explicit – i.e. is it clear how the data was analysed to arrive at the results?<br>• How systematic is the analysis, is the procedure reliable/dependable?<br>• Is it clear how the themes and concepts were derived from the data?	Rigorous<br><br>Not rigorous<br><br>Not sure/not reported<br>✓	Comments: little detail about how themes derived but ? this is due to the fact that they are reported in another publication
**9. Is the data 'rich'?**<br>For example:<br>• How well are the contexts of the data described?<br>• Has the diversity of perspective and content been explored?<br>• How well has the detail and depth been demonstrated?<br>• Are responses compared and contrasted	Rich<br><br>Poor<br><br>Not sure/not reported<br>✓	Comments: little detail about how themes derived but ? this is due to the fact that they are reported in another publication. But findings are relevant to reflecting on the context and experience of doing the project using adapted EBCD

For peer review only

across groups/sites?		
**10. Is the analysis reliable?**<br>For example:<br>• Did more than 1 researcher theme and code transcripts/data?<br>• If so, how were differences resolved?<br>• Did participants feed back on the transcripts/data if possible and relevant?<br>• Were negative/discrepant results addressed or ignored?	Reliable<br>Unreliable<br>Not sure/not reported ✓	Comments: a/above
**11. Are the findings convincing?**<br>For example:<br>• Are the findings clearly presented?<br>• Are the findings internally coherent?<br>• Are extracts from the original data included?<br>• Are the data appropriately referenced?<br>• Is the reporting clear and coherent?	Convincing ✓<br>Not convincing<br>Not sure	Comments:<br>a/above
**12. Are the findings relevant to the aims of the study?**	Relevant ✓	Comments:

For peer review only

	Irrelevant<br><br>Partially relevant	
**13. Conclusions**<br><br>For example:<br><br>• How clear are the links between data, interpretation and conclusions?<br><br>• Are the conclusions plausible and coherent?<br><br>• Have alternative explanations been explored and discounted?<br><br>• Does this enhance understanding of the research topic?<br><br>• Are the implications of the research clearly defined?<br><br>**Is there adequate discussion of any limitations encountered?**	Adequate ✓<br><br>Inadequate<br><br>Not sure	Comments:
**Ethics**		
**14. How clear and coherent is the reporting of ethics?**<br><br>For example:<br><br>• Have ethical issues been taken into consideration?<br><br>• Are they adequately discussed e.g. do they	Appropriate ✓<br><br>Inappropriate<br><br>Not sure/not reported	Comments:

For peer review only

address consent and anonymity?<br>• Have the consequences of the research been considered i.e. raising expectations, changing behaviour?<br>• Was the study approved by an ethics committee?		
**Overall assessment**		
**As far as can be ascertained from the paper, how well was the study conducted? (see guidance notes)**	++ ✓<br>Revised to + after independent third review.	Comments: assessment given relevant to the type of reporting (ie experience of adapted EBCD- primary interview data referred to only)

**Study identification:** Include author, title, reference, year of publication	Larkin, M et al, 2015 On the Brink of Genuinely Collaborative Care: Experience-Based Co-Design in Mental Health. Qualitative Health Research2015, Vol. 25(11) 1463–1476
**Guidance topic:** Co-production in acute healthcare settings.	Key research question/aim: Not clearly stated however the researchers aimed to use their existing research to develop patient care to better meet the needs of young people with psychosis. EBCD was considered to be a good way to translate research findings into service improvements.
**Checklist completed by:**	RH

For peer review only

**Theoretical approach:** No specific theoretical framework was reported		
**1. Is a qualitative approach appropriate?**<br><br>For example:<br><br>• Does the research question seek to understand processes or structures, or illuminate subjective experiences or meanings?<br><br>• Could a quantitative approach better have addressed the research question?	Appropriate ✓<br><br>Inappropriate<br><br>Not sure	Comments:
**2. Is the study clear in what it seeks to do?**<br><br>For example:<br><br>• Is the purpose of the study discussed – aims/objectives/research question/s?<br><br>• Is there adequate/appropriate reference to the literature?<br><br>• Are underpinning values/assumptions/theory discussed?	Clear ✓<br><br>Unclear<br><br>Mixed	Comments:
**Study design**		
**3. How defensible/rigorous is the research design/methodology?**<br><br>For example:	Defensible<br><br>Indefensible	Comments: the qualitative study does appear to be rigorous however, the audit after the co-design aspects of the paper is much less clear.

For peer review only

• Is the design appropriate to the research question?<br>• Is a rationale given for using a qualitative approach?<br>• Are there clear accounts of the rationale/justification for the sampling, data collection and data analysis techniques used?<br>• Is the selection of cases/sampling strategy theoretically justified?	Not sure ✓	
**Data collection**		
**4. How well was the data collection carried out?**<br>For example:<br>• Are the data collection methods clearly described?<br>• Were the appropriate data collected to address the research question?<br>• Was the data collection and record keeping systematic?	Appropriately ✓<br>Inappropriately<br>Not sure/inadequately reported	Comments: the qualitative study does appear to be rigorous however, the audit after the co-design aspects of the paper is much less clear
**Trustworthiness**		

For peer review only

**5. Is the role of the researcher clearly described?**<br>For example:<br>• Has the relationship between the researcher and the participants been adequately considered?<br>• Does the paper describe how the research was explained and presented to the participants?	Clearly described<br>Unclear ✓<br>Not described	Comments:
**6. Is the context clearly described?**<br>For example:<br>• Are the characteristics of the participants and settings clearly defined?<br>• Were observations made in a sufficient variety of circumstances<br>• Was context bias considered	Clear ✓<br>Unclear<br>Not sure	Comments:
**7. Were the methods reliable?**<br>For example:<br>• Was data collected by more than 1 method?<br>• Is there justification for triangulation, or for not triangulating?	Reliable<br>Unreliable<br>Not sure ✓	Comments: not reported

For peer review only

• Do the methods investigate what they claim to?		
**Analysis**		
**8. Is the data analysis sufficiently rigorous?**<br>For example:<br>• Is the procedure explicit – i.e. is it clear how the data was analysed to arrive at the results?<br>• How systematic is the analysis, is the procedure reliable/dependable?<br>• Is it clear how the themes and concepts were derived from the data?	Rigorous<br>Not rigorous<br>Not sure/not reported ✓	Comments: not reported
**9. Is the data 'rich'?**<br>For example:<br>• How well are the contexts of the data described?<br>• Has the diversity of perspective and content been explored?<br>• How well has the detail and depth been demonstrated?<br>• Are responses compared and contrasted	Rich ✓<br>Poor<br>Not sure/not reported	Comments: I think there is a lot of information about conducting EBCD but it doesn't fit easily in the usual quality assessments. However, I think the complexity of what we are looking at makes it difficult.

across groups/sites?		
**10. Is the analysis reliable?**<br>For example:<br>• Did more than 1 researcher theme and code transcripts/data?<br>• If so, how were differences resolved?<br>• Did participants feed back on the transcripts/data if possible and relevant?<br>• Were negative/discrepant results addressed or ignored?	Reliable<br>Unreliable<br>Not sure/not reported ✓	Comments: not much detail given
**11. Are the findings convincing?**<br>For example:<br>• Are the findings clearly presented?<br>• Are the findings internally coherent?<br>• Are extracts from the original data included?<br>• Are the data appropriately referenced?<br>• Is the reporting clear and coherent?	Convincing ✓<br>Not convincing<br>Not sure	Comments: The findings aren't really 'findings' as such. It is a discussion of implementing EBCD. The information is convincing in terms of what we learn about the challenges and facilitators of using EBCD
**12. Are the findings relevant to the aims of the study?**	Relevant ✓<br>Irrelevant	Comments:

For peer review only

	Partially relevant	
**13. Conclusions**<br><br>For example:<br><br>• How clear are the links between data, interpretation and conclusions?<br><br>• Are the conclusions plausible and coherent?<br><br>• Have alternative explanations been explored and discounted?<br><br>• Does this enhance understanding of the research topic?<br><br>• Are the implications of the research clearly defined?<br><br>**Is there adequate discussion of any limitations encountered?**	Adequate ✓<br><br>Inadequate<br><br>Not sure	Comments:
**Ethics**		
**14. How clear and coherent is the reporting of ethics?**<br><br>For example:<br><br>• Have ethical issues been taken into consideration?<br><br>• Are they adequately discussed e.g. do they	Appropriate✓<br><br>Inappropriate<br><br>Not sure/not reported	Comments:

For peer review only

address consent and anonymity?<br>• Have the consequences of the research been considered i.e. raising expectations, changing behaviour?<br>• Was the study approved by an ethics committee?		
**Overall assessment**		
**As far as can be ascertained from the paper, how well was the study conducted? (see guidance notes)**	++<br>+ ✓<br>−	Comments:

**Study identification:** Include author, title, reference, year of publication	Locock, L et al, (2014) Using a national archive of patient experience narratives to promote local patient-centered quality improvement: an ethnographic process evaluation of 'accelerated' experience-based co-design. Journal of Health Services Research &Policy2014, Vol. 19(4) 200–207
**Guidance topic:** Co-production in acute healthcare settings.	**Key research question/aim:**
**Checklist completed by:**	FJ
**Theoretical approach: A-EBCD v EBCD (12 existing studies) Ethnographic process evaluation using multiple methods**	

For peer review only

**1. Is a qualitative approach appropriate?**<br>For example:<br>• Does the research question seek to understand processes or structures, or illuminate subjective experiences or meanings?<br>• Could a quantitative approach better have addressed the research question?	Appropriate ✓<br>Inappropriate<br>Not sure	Comments:
**2. Is the study clear in what it seeks to do?**<br>For example:<br>• Is the purpose of the study discussed – aims/objectives/research question/s?<br>• Is there adequate/appropriate reference to the literature?<br>• Are underpinning values/assumptions/theory discussed?	Clear ✓<br>Unclear<br>Mixed	Comments:
**Study design**		
**3. How defensible/rigorous is the research design/methodology?**<br>For example:<br>• Is the design appropriate to the research question?<br>• Is a rationale given for using a qualitative approach?<br>• Are there clear accounts of the rationale/justification for	Defensible ✓<br>Indefensible<br>Not sure	Comments:

For peer review only

the sampling, data collection and data analysis techniques used?<br>• Is the selection of cases/sampling strategy theoretically justified?		
**Data collection**		
**4. How well was the data collection carried out?**<br>For example:<br>• Are the data collection methods clearly described?<br>• Were the appropriate data collected to address the research question?<br>• Was the data collection and record keeping systematic?	Appropriately ✓<br>Inappropriately<br>Not sure/inadequately reported	Comments:<br>Limited reporting of findings – no detail about pathway and impact of improvement activities
**Trustworthiness**		

For peer review only

**5. Is the role of the researcher clearly described?**<br>For example:<br>• Has the relationship between the researcher and the participants been adequately considered?<br>• Does the paper describe how the research was explained and presented to the participants?	Clearly described<br>Unclear<br>Not described ✓	Comments:
**6. Is the context clearly described?**<br>For example:<br>• Are the characteristics of the participants and settings clearly defined?<br>• Were observations made in a sufficient variety of circumstances<br>• Was context bias considered	Clear<br>Unclear ✓<br>Not sure	Comments:<br>Very little detail about context- Lung cancer and ITU; context bas not considered
**7. Were the methods reliable?**<br>For example:<br>• Was data collected by more than 1 method?<br>• Is there justification for triangulation, or for not triangulating?	Reliable ✓<br>Unreliable<br>Not sure	Comments:

For peer review only

• Do the methods investigate what they claim to?		
**Analysis**		
**8. Is the data analysis sufficiently rigorous?**<br>For example:<br>• Is the procedure explicit – i.e. is it clear how the data was analysed to arrive at the results?<br>• How systematic is the analysis, is the procedure reliable/dependable?<br>• Is it clear how the themes and concepts were derived from the data?	Rigorous ✓<br>Not rigorous<br>Not sure/not reported	Comments:
**9. Is the data 'rich'?**<br>For example:<br>• How well are the contexts of the data described?<br>• Has the diversity of perspective and content been explored?<br>• How well has the detail and depth been demonstrated?<br>• Are responses compared and contrasted across groups/sites?	Rich<br>Poor<br>Not sure/not reported ✓	Comments: minimal reporting of context, and whether responses were compared/contrasted
**10. Is the analysis reliable?**	Reliable	Comments:

For peer review only

For example:<br>• Did more than 1 researcher theme and code transcripts/data?<br>• If so, how were differences resolved?<br>• Did participants feed back on the transcripts/data if possible and relevant?<br>• Were negative/discrepant results addressed or ignored?	Unreliable<br>Not sure/not reported ✓	
**11. Are the findings convincing?**<br>For example:<br>• Are the findings clearly presented?<br>• Are the findings internally coherent?<br>• Are extracts from the original data included?<br>• Are the data appropriately referenced?<br>• Is the reporting clear and coherent?	Convincing ✓<br>Not convincing<br>Not sure	Comments:
**12. Are the findings relevant to the aims of the study?**	Relevant ✓<br>Irrelevant<br>Partially relevant	Comments:
**13. Conclusions**<br>For example:	Adequate ✓<br>Inadequate	Comments:

For peer review only

• How clear are the links between data, interpretation and conclusions?<br>• Are the conclusions plausible and coherent?<br>• Have alternative explanations been explored and discounted?<br>• Does this enhance understanding of the research topic?<br>• Are the implications of the research clearly defined?<br>**Is there adequate discussion of any limitations encountered?**	Not sure	
**Ethics**		
**14. How clear and coherent is the reporting of ethics?**<br>For example:<br>• Have ethical issues been taken into consideration?<br>• Are they adequately discussed e.g. do they address consent and anonymity?<br>• Have the consequences of the research been considered i.e. raising expectations, changing behaviour?<br>• Was the study approved by an ethics committee?	Appropriate ✓<br>Inappropriate<br>Not sure/not reported	Comments:
**Overall assessment**		
**As far as can be ascertained from the paper, how well was**	++ ✓	Comments:

For peer review only

**the study conducted? (see guidance notes)**		

**Study identification:** Include author, title, reference, year of publication	Locock, L et al, (2014) Using a national archive of patient experience narratives to promote local patient-centered quality improvement: an ethnographic process evaluation of 'accelerated' experience-based co-design. Journal of Health Services Research &Policy2014, Vol. 19(4) 200–207.
**Guidance topic:** Co-production in acute healthcare settings.	**Key research question/aim: 3 questions identified.**<br><br>1. What improvement activities does the approach stimulate (for what cost) and how do these compare with traditional EBCD?<br>2. How does using films of national rather than local narratives affect the level and quality of engagement with service improvement by local NHS staff?<br>3. How well do local patients feel national narratives represent themes important to their own experience?
**Checklist completed by:**	DJC

Theoretical approach: A-EBCD v EBCD (12 existing studies) Ethnographic process evaluation using multiple methods

**1. Is a qualitative approach appropriate?**<br><br>For example:<br><br>• Does the research question seek to understand processes or structures, or illuminate subjective experiences or meanings?<br><br>• Could a quantitative approach better have addressed the research question?	Appropriate ✓<br><br>Inappropriate<br><br>Not sure	Comments: Important to understand factors influencing the implementation of the accelerated approach and to observe the process 'first hand', same ethnographer involved though out all stages of data collection.<br><br>Also compared this study's survey data with that from evaluations of 12 previous EBCD studies.
**2. Is the study clear in what it seeks to do?**	Clear ✓	Comments: Comparing AEBCD with EBCD by investigated whether archive film could replace the need for local

For peer review only - http://bmjopen.bmj.com/site/about/guidelines.xhtml

For example:<br>• Is the purpose of the study discussed – aims/objectives/research question/s?<br>• Is there adequate/appropriate reference to the literature?<br>• Are underpinning values/assumptions/theory discussed?	Unclear<br>Mixed	discovery interviews (and film) so making EBCD faster and cheaper; aimed to halve the standard EBCD cycle from 12 to 6 months.
**Study design**		
**3. How defensible/rigorous is the research design/methodology?**<br>For example:<br>• Is the design appropriate to the research question?<br>• Is a rationale given for using a qualitative approach?<br>• Are there clear accounts of the rationale/justification for the sampling, data collection and data analysis techniques used?<br>• Is the selection of cases/sampling strategy theoretically justified?	Defensible ✓<br>Indefensible<br>Not sure	Comments: Clear justification for the methods adopted, consistent with good practice in ethnographic process evaluation.
**Data collection**		
**4. How well was the data collection carried out?**<br>For example:	Appropriately ✓<br>Inappropriately	Comments: Clear description of data collection methods, more limited reporting of findings other than for improvement activities.

For peer review only

• Are the data collection methods clearly described?<br>• Were the appropriate data collected to address the research question?<br>• Was the data collection and record keeping systematic?	Not sure/inadequately reported	
**Trustworthiness**		
**5. Is the role of the researcher clearly described?**<br>For example:<br>• Has the relationship between the researcher and the participants been adequately considered?<br>• Does the paper describe how the research was explained and presented to the participants?	Clearly described<br>Unclear<br>Not described ✓	Comments:
**6. Is the context clearly described?**<br>For example:<br>• Are the characteristics of the participants and settings clearly defined?<br>• Were observations made in a sufficient variety of circumstances	Clear<br>Unclear ✓<br>Not sure	Comments:<br>Little detail about context, focus specifically on impact of using a national versus us locally derived film, disease specific condition less relevant in this publication.

For peer review only

• Was context bias considered		
**7. Were the methods reliable?**<br>For example:<br>• Was data collected by more than 1 method?<br>• Is there justification for triangulation, or for not triangulating?<br>• Do the methods investigate what they claim to?	Reliable ✓<br>Unreliable<br>Not sure	Comments: Appropriate and clearly articulated.
**Analysis**		
**8. Is the data analysis sufficiently rigorous?**<br>For example:<br>• Is the procedure explicit – i.e. is it clear how the data was analysed to arrive at the results?<br>• How systematic is the analysis, is the procedure reliable/dependable?<br>• Is it clear how the themes and concepts were derived from the data?	Rigorous ✓<br>Not rigorous<br>Not sure/not reported	Comments: Brief summary of approaches used, all consistent with the ethnographic process evaluation and the survey data generated.
**9. Is the data 'rich'?**<br>For example:<br>• How well are the contexts of the data described?	Rich<br>Poor<br>Not sure/not reported ✓	Comments: minimal reporting of context focus specifically on impact of using a national versus us locally derived film, disease specific condition and location of the study less relevant in this publication.

For peer review only

• Has the diversity of perspective and content been explored?<br>• How well has the detail and depth been demonstrated?<br>• Are responses compared and contrasted across groups/sites?		
**10. Is the analysis reliable?**<br>For example:<br>• Did more than 1 researcher theme and code transcripts/data?<br>• If so, how were differences resolved?<br>• Did participants feed back on the transcripts/data if possible and relevant?<br>• Were negative/discrepant results addressed or ignored?	Reliable<br>Unreliable<br>Not sure/not reported ✓	Comments: Reports a two day workshop with the study team but no other details.
**11. Are the findings convincing?**<br>For example:<br>• Are the findings clearly presented?<br>• Are the findings internally coherent?<br>• Are extracts from the original data included?<br>• Are the data appropriately referenced?<br>• Is the reporting clear and coherent?	Convincing ✓<br>Not convincing<br>Not sure	Comments:

For peer review only

**12. Are the findings relevant to the aims of the study?**	Relevant ✓<br>Irrelevant<br>Partially relevant	Comments:
**13. Conclusions**<br>For example:<br>• How clear are the links between data, interpretation and conclusions?<br>• Are the conclusions plausible and coherent?<br>• Have alternative explanations been explored and discounted?<br>• Does this enhance understanding of the research topic?<br>• Are the implications of the research clearly defined?<br>**Is there adequate discussion of any limitations encountered?**	Adequate ✓<br>Inadequate<br>Not sure	Comments:
**Ethics**		
**14. How clear and coherent is the reporting of ethics?**<br>For example:<br>• Have ethical issues been taken into consideration?<br>• Are they adequately discussed e.g. do they address consent and anonymity?<br>• Have the consequences of the research been considered	Appropriate ✓<br>Inappropriate<br>Not sure/not reported	Comments:

For peer review only

i.e. raising expectations, changing behaviour?<br>• Was the study approved by an ethics committee?		
**Overall assessment**		
**As far as can be ascertained from the paper, how well was the study conducted? (see guidance notes)**	++ ✓	Comments:

**Study identification:** Include author, title, reference, year of publication	Piper, D. et al, 2012 Utilizing experience-based co-design to improve the experience of patients accessing emergency departments in New South Wales public hospitals: an evaluation study. Health Services Management Research2012;25: 162 – 172
**Guidance topic:** Co-production in acute healthcare settings.	**Key research question/aim:**<br>What specific improvements did EBCD deliver for patients, carers and staff in the Emergency Departments involved in the project?<br>What did it feel like to take part in EBCD as compared to other redesign approaches? What did participants identify as the 'must do' or key success factors in EBCD?<br>What can the pilot tell us about the likely sustainability and spread of improvements brought about by EBCD?<br>What lessons can be drawn from the pilot about future EBCD projects in New South Wales?

For peer review only

**Checklist completed by:**	FJ	
**Theoretical approach:** evaluation of existing EBCD improvement projects; no other details about theoretical approach		
**1. Is a qualitative approach appropriate?**<br>For example:<br>• Does the research question seek to understand processes or structures, or illuminate subjective experiences or meanings?<br>• Could a quantitative approach better have addressed the research question?	Appropriate ✓<br>Inappropriate<br>Not sure	Comments:
**2. Is the study clear in what it seeks to do?**<br>For example:<br>• Is the purpose of the study discussed – aims/objectives/research question/s?<br>• Is there adequate/appropriate reference to the literature?<br>• Are underpinning values/assumptions/theory discussed?	Clear ✓<br>Unclear<br>Mixed	Comments:
**Study design**		
**3. How defensible/rigorous is the research design/methodology?**	Defensible	Comments:

For peer review only - http://bmjopen.bmj.com/site/about/guidelines.xhtml

For example:<br>• Is the design appropriate to the research question?<br>• Is a rationale given for using a qualitative approach?<br>• Are there clear accounts of the rationale/justification for the sampling, data collection and data analysis techniques used?<br>• Is the selection of cases/sampling strategy theoretically justified?	Indefensible<br>Not sure ✓	Not much detail about sampling. Selection of cases not justified.
**Data collection**		
**4. How well was the data collection carried out?**<br>For example:<br>• Are the data collection methods clearly described?<br>• Were the appropriate data collected to address the research question?<br>• Was the data collection and record keeping systematic?	Appropriately ✓<br>Inappropriately<br>Not sure/inadequately reported	Comments:
**Trustworthiness**		

For peer review only

**5. Is the role of the researcher clearly described?**<br>For example:<br>• Has the relationship between the researcher and the participants been adequately considered?<br>• Does the paper describe how the research was explained and presented to the participants?	Clearly described<br>Unclear ✓<br>Not described	Comments: interview and document data analysed by an independent analyst
**6. Is the context clearly described?**<br>For example:<br>• Are the characteristics of the participants and settings clearly defined?<br>• Were observations made in a sufficient variety of circumstances<br>• Was context bias considered	Clear ✓<br>Unclear<br>Not sure	Comments: no comment on content bias
**7. Were the methods reliable?**<br>For example:<br>• Was data collected by more than 1 method?<br>• Is there justification for triangulation, or for not triangulating?	Reliable ✓<br>Unreliable<br>Not sure	Comments:<br>Interviews with participants, analysis of project reports, interviews senior managers

For peer review only

• Do the methods investigate what they claim to?		
**Analysis**		
**8. Is the data analysis sufficiently rigorous?**<br>For example:<br>• Is the procedure explicit – i.e. is it clear how the data was analysed to arrive at the results?<br>• How systematic is the analysis, is the procedure reliable/dependable?<br>• Is it clear how the themes and concepts were derived from the data?	Rigorous<br>Not rigorous<br>Not sure/not ✓reported	Comments:<br>Not clear how findings were arrived at
**9. Is the data 'rich'?**<br>For example:<br>• How well are the contexts of the data described?<br>• Has the diversity of perspective and content been explored?<br>• How well has the detail and depth been demonstrated?<br>• Are responses compared and contrasted across groups/sites?	Rich<br>Poor<br>Not sure/not reported ✓	Comments: no illustrative quotes. Data structured into main categories (barriers and achievements) but no themes/sub-themes

For peer review only

**10. Is the analysis reliable?**<br><br>For example:<br><br>• Did more than 1 researcher theme and code transcripts/data?<br><br>• If so, how were differences resolved?<br><br>• Did participants feed back on the transcripts/data if possible and relevant?<br><br>• Were negative/discrepant results addressed or ignored?	Reliable<br><br>Unreliable<br><br>Not sure/not reported ✓	Comments:
**11. Are the findings convincing?**<br><br>For example:<br><br>• Are the findings clearly presented?<br><br>• Are the findings internally coherent?<br><br>• Are extracts from the original data included?<br><br>• Are the data appropriately referenced?<br><br>• Is the reporting clear and coherent?	Convincing ✓<br><br>Not convincing<br><br>Not sure	Comments:<br><br>Finding reported clearly but as a commentary rather than using a traditional way with themes/sub-themes –illustrative quotes etc.
**12. Are the findings relevant to the aims of the study?**	Relevant ✓<br><br>Irrelevant<br><br>Partially relevant	Comments:

For peer review only

**13. Conclusions**<br>For example:<br>• How clear are the links between data, interpretation and conclusions?<br>• Are the conclusions plausible and coherent?<br>• Have alternative explanations been explored and discounted?<br>• Does this enhance understanding of the research topic?<br>• Are the implications of the research clearly defined?<br>**Is there adequate discussion of any limitations encountered?**	Adequate ✓<br>Inadequate<br>Not sure	Comments:
**Ethics**		
**14. How clear and coherent is the reporting of ethics?**<br>For example:<br>• Have ethical issues been taken into consideration?<br>• Are they adequately discussed e.g. do they address consent and anonymity?<br>• Have the consequences of the research been considered i.e. raising expectations, changing behaviour?	Appropriate<br>Inappropriate<br>Not sure/not reported ✓	Comments:<br>No mention of ethical approval

For peer review only

• Was the study approved by an ethics committee?		
**Overall assessment**		
**As far as can be ascertained from the paper, how well was the study conducted? (see guidance notes)**	+ ✓	Comments:

**Study identification:** Include author, title, reference, year of publication	Piper, D. et al, 2012 Utilizing experience-based co-design to improve the experience of patients accessing emergency departments in New South Wales public hospitals: an evaluation study. Health Services Management Research2012;25: 162 – 172
**Guidance topic:** Co-production in acute healthcare settings.	**Key research question/aim:** Not clearly stated - This paper presents the findings from three evaluations of two subsequent multisite EBCD programmes conducted by New South Wales Health (Australia) over two years involving seven hospital sites to take stock of the impact of EBCD on patients' experience of ED care.
**Checklist completed by:**	RH
**Theoretical approach:** No specific theoretical framework was reported	

**1. Is a qualitative approach appropriate?**<br>For example:<br>• Does the research question seek to understand processes or structures, or illuminate subjective experiences or meanings?	Appropriate ✓<br>Inappropriate<br>Not sure	Comments:

For peer review only

• Could a quantitative approach better have addressed the research question?		
**2. Is the study clear in what it seeks to do?**<br>For example:<br>• Is the purpose of the study discussed – aims/objectives/research question/s?<br>• Is there adequate/appropriate reference to the literature?<br>• Are underpinning values/assumptions/theory discussed?	Clear✓<br>Unclear<br>Mixed	Comments:
**Study design**		
**3. How defensible/rigorous is the research design/methodology?**<br>For example:<br>• Is the design appropriate to the research question?<br>• Is a rationale given for using a qualitative approach?<br>• Are there clear accounts of the rationale/justification for the sampling, data collection and data analysis techniques used?<br>• Is the selection of cases/sampling strategy theoretically justified?	Defensible ✓<br>Indefensible<br>Not sure	Comments:

For peer review only

**Data collection**		
**4. How well was the data collection carried out?**<br>For example:<br>• Are the data collection methods clearly described?<br>• Were the appropriate data collected to address the research question?<br>• Was the data collection and record keeping systematic?	Appropriately ✓<br>Inappropriately<br>Not sure/inadequately reported	Comments: not a great deal of detail is given
**Trustworthiness**		
**5. Is the role of the researcher clearly described?**<br>For example:<br>• Has the relationship between the researcher and the participants been adequately considered?<br>• Does the paper describe how the research was explained and presented to the participants?	Clearly described<br>Unclear ✓<br>Not described	Comments:
**6. Is the context clearly described?**	Clear ✓	Comments:

For peer review only

For example:<br>• Are the characteristics of the participants and settings clearly defined?<br>• Were observations made in a sufficient variety of circumstances<br>• Was context bias considered	Unclear<br>Not sure	
**7. Were the methods reliable?**<br>For example:<br>• Was data collected by more than 1 method?<br>• Is there justification for triangulation, or for not triangulating?<br>• Do the methods investigate what they claim to?	Reliable<br>Unreliable<br>Not sure ✓	Comments: varied across sites – not fully reported in an auditable way
**Analysis**		
**8. Is the data analysis sufficiently rigorous?**<br>For example:<br>• Is the procedure explicit – i.e. is it clear how the data was analysed to arrive at the results?<br>• How systematic is the analysis, is the procedure reliable/dependable?<br>• Is it clear how the themes and concepts were	Rigorous<br>Not rigorous<br>Not sure/not reported ✓	Comments: one researcher did analysis, not much further detail given.

For peer review only

derived from the data?		
**9. Is the data 'rich'?**<br>For example:<br>• How well are the contexts of the data described?<br>• Has the diversity of perspective and content been explored?<br>• How well has the detail and depth been demonstrated?<br>• Are responses compared and contrasted across groups/sites?	Rich<br>Poor<br>Not sure/not reported ✓	Comments: not a lot of detail
**10. Is the analysis reliable?**<br>For example:<br>• Did more than 1 researcher theme and code transcripts/data?<br>• If so, how were differences resolved?<br>• Did participants feed back on the transcripts/data if possible and relevant?<br>• Were negative/discrepant results addressed or ignored?	Reliable<br>Unreliable<br>Not sure/not reported ✓	Comments: not much detail given, one researcher
**11. Are the findings convincing?**	Convincing ✓	Comments:

For peer review only

For example:<br>• Are the findings clearly presented?<br>• Are the findings internally coherent?<br>• Are extracts from the original data included?<br>• Are the data appropriately referenced?<br>• Is the reporting clear and coherent?	Not convincing<br>Not sure	
**12. Are the findings relevant to the aims of the study?**	Relevant ✓<br>Irrelevant<br>Partially relevant	Comments:
**13. Conclusions**<br>For example:<br>• How clear are the links between data, interpretation and conclusions?<br>• Are the conclusions plausible and coherent?<br>• Have alternative explanations been explored and discounted?<br>• Does this enhance understanding of the research topic?<br>• Are the implications of the research clearly defined?<br>**Is there adequate discussion of any limitations**	Adequate ✓<br>Inadequate<br>Not sure	Comments:

For peer review only

**encountered?**		
**Ethics**		
**14. How clear and coherent is the reporting of ethics?**<br><br>For example:<br><br>• Have ethical issues been taken into consideration?<br><br>• Are they adequately discussed e.g. do they address consent and anonymity?<br><br>• Have the consequences of the research been considered i.e. raising expectations, changing behaviour?<br><br>• Was the study approved by an ethics committee?	Appropriate<br><br>Inappropriate<br><br>Not sure/not reported ✓	Comments: Not addressed in the paper
**Overall assessment**		
**As far as can be ascertained from the paper, how well was the study conducted? (see guidance notes)**	++<br><br>+ ✓<br><br>−	Comments:

For peer review only

**Study identification:** Include author, title, reference, year of publication	Multiple sclerosis outpatient future groups: improving the quality of participant interaction and ideation tools within service improvement activities. Alison Thomson, Carol Rivas and Gavin Giovannoni. BMC Health Services Research (2015) 15:105. DOI 10.1186/s12913-015-0773-8	
**Guidance topic:**	**Key research question/aim:**<br><br>The aim of the study is to consider how the role of a professional designer as the facilitator can develop more radical ideas, beyond solving simple problems, from patients and staff in a service improvement activity.	
**Checklist completed by:**	SH	
**Theoretical approach**		
**1. Is a qualitative approach appropriate?**<br><br>For example:<br><br>• Does the research question seek to understand processes or structures, or illuminate subjective experiences or meanings?<br><br>• Could a quantitative approach better have addressed the research question?	Appropriate ✓<br><br>Inappropriate<br><br>Not sure	Comments: Appropriate - Illuminates subjective experiences which could not have been carried out by quantitative approaches.
**2. Is the study clear in what it seeks to do?**<br><br>For example:<br><br>• Is the purpose of the study discussed –	Clear ✓<br><br>Unclear<br><br>Mixed	Comments: The aim and the EBCD approach is explained and discussed. The references are appropriate.

For peer review only

aims/objectives/research question/s?<br>• Is there adequate/appropriate reference to the literature?<br>• Are underpinning values/assumptions/theory discussed?		
**Study design**		
**3. How defensible/rigorous is the research design/methodology?**<br>For example:<br>• Is the design appropriate to the research question?<br>• Is a rationale given for using a qualitative approach?<br>• Are there clear accounts of the rationale/justification for the sampling, data collection and data analysis techniques used?<br>• Is the selection of cases/sampling strategy theoretically justified?	Defensible ✓<br>Indefensible<br>Not sure	Comments: The focus group approach is appropriate for eliciting the views of staff and patients. The rationale for the sampling of the staff and patient participant groups are justified.
**Data collection**		
**4. How well was the data collection carried out?**<br>For example:<br>• Are the data collection methods clearly	Appropriately ✓<br>Inappropriately	Comments: The mapping of the staff and patient journey and the development of service improvement are clearly described. The data collection is systematic and includes verbatim quotations, photographs

For peer review only

described?<br>• Were the appropriate data collected to address the research question?<br>• Was the data collection and record keeping systematic?	Not sure/inadequately reported	and diagrams.
**Trustworthiness**		
**5. Is the role of the researcher clearly described?**<br>For example:<br>• Has the relationship between the researcher and the participants been adequately considered?<br>• Does the paper describe how the research was explained and presented to the participants?	Clearly described ✓<br>Unclear<br>Not described	Comments: The relationships are described. The participants were involved with the research on an on-going basis via the design-led process.
**6. Is the context clearly described?**<br>For example:<br>• Are the characteristics of the participants and settings clearly defined?<br>• Were observations made in a sufficient variety of circumstances<br>• Was context bias considered	Clear ✓<br>Unclear<br>Not sure	Comments: The context of the MS outpatient clinic was clearly defined. A range of staff participants were interviewed and male and female patients included. The authors acknowledge that the final context of use should be considered.
**7. Were the methods reliable?**	Reliable ✓	Comments: The methods were reliable.. Analogies and props were used to elicit rich data. Non-participant observation of the focus groups was

For peer review only

For example:<br>• Was data collected by more than 1 method?<br>• Is there justification for triangulation, or for not triangulating?<br>• Do the methods investigate what they claim to?	Unreliable<br>Not sure	also carried out.
**Analysis**		
**8. Is the data analysis sufficiently rigorous?**<br>For example:<br>• Is the procedure explicit – i.e. is it clear how the data was analysed to arrive at the results?<br>• How systematic is the analysis, is the procedure reliable/dependable?<br>• Is it clear how the themes and concepts were derived from the data?	Rigorous<br>Not rigorous ✓<br>Not sure/not reported	Comments: It is unclear as the analysis is only briefly reported but it is stated that thematic analysis was carried out using NVivo v (8) and grounded theory principles were used.
**9. Is the data 'rich'?**<br>For example:<br>• How well are the contexts of the data described?<br>• Has the diversity of perspective and content been explored?<br>• How well has the detail and depth been demonstrated?	Rich ✓<br>Poor<br>Not sure/not reported	Comments: The data includes relevant conversation excerpts and photographs of work completed by the groups.

For peer review only

• Are responses compared and contrasted across groups/sites?		
**10. Is the analysis reliable?**<br><br>For example:<br><br>• Did more than 1 researcher theme and code transcripts/data?<br><br>• If so, how were differences resolved?<br><br>• Did participants feed back on the transcripts/data if possible and relevant?<br><br>• Were negative/discrepant results addressed or ignored?	Reliable<br><br>Unreliable<br><br>Not sure/not reported ✓	Comments:
**11. Are the findings convincing?**<br><br>For example:<br><br>• Are the findings clearly presented?<br><br>• Are the findings internally coherent?<br><br>• Are extracts from the original data included?<br><br>• Are the data appropriately referenced?<br><br>• Is the reporting clear and coherent?	Convincing ✓<br><br>Not convincing<br><br>Not sure	Comments: The findings are clearly presented and include extracts from the original data in then form of quotations and photographs.
**12. Are the findings relevant to the aims of the study?**	Relevant x<br><br>Irrelevant	Comments: The findings are relevant and show that design-led methods can facilitate the development of ideas and enhance service improvement activities.

For peer review only

	Partially relevant	
**13. Conclusions**<br><br>For example:<br><br>• How clear are the links between data, interpretation and conclusions?<br><br>• Are the conclusions plausible and coherent?<br><br>• Have alternative explanations been explored and discounted?<br><br>• Does this enhance understanding of the research topic?<br><br>• Are the implications of the research clearly defined?<br><br>**Is there adequate discussion of any limitations encountered?**	Adequate ✓<br><br>Inadequate<br><br>Not sure	Comments: The conclusions are plausible and enhance the research topic. The implication that social science methods can be used to integrate design-led approaches in service improvement is clearly defined.<br><br>The limitations are discussed adequately.
**Ethics**		
**14. How clear and coherent is the reporting of ethics?**<br><br>For example:<br><br>• Have ethical issues been taken into consideration?<br><br>• Are they adequately discussed e.g. do they address consent and anonymity?<br><br>• Have the consequences of the research been	Appropriate<br><br>Inappropriate<br><br>Not sure/not reported ✓	Comments: Only very brief references to ethical considerations. It is stated that ethical approval was given.

For peer review only

considered i.e. raising expectations, changing behaviour?<br>• Was the study approved by an ethics committee?		
**Overall assessment**		
**As far as can be ascertained from the paper, how well was the study conducted? (see guidance notes)**	++<br>+ ✓<br>−	Comments:

**Study identification:** Include author, title, reference, year of publication	Thompson, A., Rivas, C., Giovannoni, G. Multiple sclerosis outpatient future groups: improving the quality of participant interaction and ideation tools within service improvement activities. BMC Health Services Research (2015) 15:105. DOI 10.1186/s12913-015-0773-8
**Guidance topic:**	**Key research question/aim:**<br>The aim of the study is to consider how the role of a professional designer as the facilitator can develop more radical ideas, beyond solving simple problems, from patients and staff in a service improvement activity.
**Checklist completed by:**	DJC
**Theoretical approach**	

For peer review only

**1. Is a qualitative approach appropriate?**<br><br>For example:<br><br>• Does the research question seek to understand processes or structures, or illuminate subjective experiences or meanings?<br><br>• Could a quantitative approach better have addressed the research question?	Appropriate ✓<br><br>Inappropriate<br><br>Not sure	Comments:
**2. Is the study clear in what it seeks to do?**<br><br>For example:<br><br>• Is the purpose of the study discussed – aims/objectives/research question/s?<br><br>• Is there adequate/appropriate reference to the literature?<br><br>• Are underpinning values/assumptions/theory discussed?	Clear ✓<br><br>Unclear<br><br>Mixed	Comments:
**Study design**		
**3. How defensible/rigorous is the research design/methodology?**<br><br>For example:<br><br>• Is the design appropriate to the research question?<br><br>• Is a rationale given for using a qualitative	Defensible ✓<br><br>Indefensible<br><br>Not sure	Comments:

For peer review only

approach?<br>• Are there clear accounts of the rationale/justification for the sampling, data collection and data analysis techniques used?<br>• Is the selection of cases/sampling strategy theoretically justified?		
**Data collection**		
**4. How well was the data collection carried out?**<br>For example:<br>• Are the data collection methods clearly described?<br>• Were the appropriate data collected to address the research question?<br>• Was the data collection and record keeping systematic?	Appropriately ✓<br>Inappropriately<br>Not sure/inadequately reported	Comments:
**Trustworthiness**		
**5. Is the role of the researcher clearly described?**<br>For example:<br>• Has the relationship between the researcher and the participants been adequately considered?<br>• Does the paper describe how the research was	Clearly described ✓<br>Unclear<br>Not described	Comments:

For peer review only

explained and presented to the participants?		
**6. Is the context clearly described?**<br>For example:<br>• Are the characteristics of the participants and settings clearly defined?<br>• Were observations made in a sufficient variety of circumstances<br>• Was context bias considered	Clear ✓<br>Unclear<br>Not sure	Comments:
**7. Were the methods reliable?**<br>For example:<br>• Was data collected by more than 1 method?<br>• Is there justification for triangulation, or for not triangulating?<br>• Do the methods investigate what they claim to?	Reliable ✓<br>Unreliable<br>Not sure	Comments:
**Analysis**		
**8. Is the data analysis sufficiently rigorous?**<br>For example:<br>• Is the procedure explicit – i.e. is it clear how the data was analysed to arrive at the results?<br>• How systematic is the analysis, is the procedure	Rigorous<br>Not rigorous<br>Not sure/not reported ✓	Comments: Analysis is briefly outlined, reports using thematic analysis and grounded theory principles but no more information is provided.<br>.

For peer review only

reliable/dependable?<br>• Is it clear how the themes and concepts were derived from the data?		
**9. Is the data 'rich'?**<br>For example:<br>• How well are the contexts of the data described?<br>• Has the diversity of perspective and content been explored?<br>• How well has the detail and depth been demonstrated?<br>• Are responses compared and contrasted across groups/sites?	Rich ✓<br>Poor<br>Not sure/not reported	Comments: The data includes relevant conversation excerpts and photographs of work completed by the groups.
**10. Is the analysis reliable?**<br>For example:<br>• Did more than 1 researcher theme and code transcripts/data?<br>• If so, how were differences resolved?<br>• Did participants feed back on the transcripts/data if possible and relevant?<br>• Were negative/discrepant results addressed or ignored?	Reliable<br>Unreliable<br>Not sure/not reported ✓	Comments:

For peer review only

**11. Are the findings convincing?**<br><br>For example:<br><br>• Are the findings clearly presented?<br>• Are the findings internally coherent?<br>• Are extracts from the original data included?<br>• Are the data appropriately referenced?<br>• Is the reporting clear and coherent?	Convincing ✓<br><br>Not convincing<br><br>Not sure	Comments: Clear and comprehensive reporting of the findings with inclusion of extracts from the original data in the form of quotations and photographs.
**12. Are the findings relevant to the aims of the study?**	Relevant ✓<br><br>Irrelevant<br><br>Partially relevant	Comments:
**13. Conclusions**<br><br>For example:<br><br>• How clear are the links between data, interpretation and conclusions?<br>• Are the conclusions plausible and coherent?<br>• Have alternative explanations been explored and discounted?<br>• Does this enhance understanding of the research topic?<br>• Are the implications of the research clearly defined?	Adequate ✓<br><br>Inadequate<br><br>Not sure	Comments:

For peer review only

**Is there adequate discussion of any limitations encountered?**		
**Ethics**		
**14. How clear and coherent is the reporting of ethics?**<br><br>For example:<br><br>• Have ethical issues been taken into consideration?<br><br>• Are they adequately discussed e.g. do they address consent and anonymity?<br><br>• Have the consequences of the research been considered i.e. raising expectations, changing behaviour?<br><br>• Was the study approved by an ethics committee?	Appropriate<br><br>Inappropriate<br><br>Not sure/not reported ✓	Comments: No detailed consideration of ethical issues. Ethical approval was reported in the paper.
**Overall assessment**		
**As far as can be ascertained from the paper, how well was the study conducted? (see guidance notes)**	++<br><br>+ ✓<br><br>–	Comments:

For peer review only

**Study identification:** Include author, title, reference, year of publication	Tollyfield, R. 2014 Facilitating an accelerated experience-based co-design project. British Journal of Nursing, 23, (3):136-141	
**Guidance topic:** Co-production in acute healthcare settings.	**Key research question/aim:** Aims of the paper are stated but not the aims of the actual study. Later in the paper, it is stated that the authors explored the potential of creating and testing a 35-minute film using patient and carer narrative from a national archive of interviews held by the Health Experiences Research Group at the University of Oxford.	
**Checklist completed by:**	RH	
**Theoretical approach:** No specific theoretical framework was reported		
**1. Is a qualitative approach appropriate?**<br>For example:<br>• Does the research question seek to understand processes or structures, or illuminate subjective experiences or meanings?<br>• Could a quantitative approach better have addressed the research question?	Appropriate<br>Inappropriate<br>Not sure ✓	Comments: This paper does not report a research study specifically – there is another study by the team that is cited. This is a discussion of facilitating the EBCD process which also addressed the barriers and facilitators to using EBCD in acute healthcare settings.
**2. Is the study clear in what it seeks to do?**<br>For example:<br>• Is the purpose of the study discussed – aims/objectives/research question/s?	Clear<br>Unclear<br>Mixed ✓	Comments: The study in which Tollyfield acted as facilitator for an AEBCD project is clear but not reported in this paper.

• Is there adequate/appropriate reference to the literature?<br>• Are underpinning values/assumptions/theory discussed?		
**Study design**		
**3. How defensible/rigorous is the research design/methodology?**<br>For example:<br>• Is the design appropriate to the research question?<br>• Is a rationale given for using a qualitative approach?<br>• Are there clear accounts of the rationale/justification for the sampling, data collection and data analysis techniques used?<br>• Is the selection of cases/sampling strategy theoretically justified?	Defensible<br>Indefensible<br>Not sure ✓	Comments:
**Data collection**		
**4. How well was the data collection carried out?**<br>For example:	Appropriately<br>Inappropriately	Comments:

For peer review only

• Are the data collection methods clearly described?<br>• Were the appropriate data collected to address the research question?<br>• Was the data collection and record keeping systematic?	Not sure/inadequately reported ✓	
**Trustworthiness**		
**5. Is the role of the researcher clearly described?**<br>For example:<br>• Has the relationship between the researcher and the participants been adequately considered?<br>• Does the paper describe how the research was explained and presented to the participants?	Clearly described<br>Unclear ✓<br>Not described	Comments:
**6. Is the context clearly described?**<br>For example:<br>• Are the characteristics of the participants and settings clearly defined?	Clear<br>Unclear<br>Not sure ✓	Comments:

For peer review only

• Were observations made in a sufficient variety of circumstances<br>• Was context bias considered		
**7. Were the methods reliable?**<br>For example:<br>• Was data collected by more than 1 method?<br>• Is there justification for triangulation, or for not triangulating?<br>• Do the methods investigate what they claim to?	Reliable<br>Unreliable<br>Not sure ✓	Comments: not reported
**Analysis**		
**8. Is the data analysis sufficiently rigorous?**<br>For example:<br>• Is the procedure explicit – i.e. is it clear how the data was analysed to arrive at the results?<br>• How systematic is the analysis, is the procedure reliable/dependable?<br>• Is it clear how the themes and concepts	Rigorous<br>Not rigorous<br>Not sure/not reported ✓	Comments: not reported

For peer review only

were derived from the data?		
**9. Is the data 'rich'?**<br>For example:<br>• How well are the contexts of the data described?<br>• Has the diversity of perspective and content been explored?<br>• How well has the detail and depth been demonstrated?<br>• Are responses compared and contrasted across groups/sites?	Rich<br>Poor<br>Not sure/not reported ✓	Comments: There is a lot of useful information about facilitating EBCD
**10. Is the analysis reliable?**<br>For example:<br>• Did more than 1 researcher theme and code transcripts/data?<br>• If so, how were differences resolved?<br>• Did participants feed back on the transcripts/data if possible and relevant?<br>• Were negative/discrepant results addressed or ignored?	Reliable<br>Unreliable<br>Not sure/not reported ✓	Comments: Not much detail given

For peer review only

**11. Are the findings convincing?**<br>For example:<br>• Are the findings clearly presented?<br>• Are the findings internally coherent?<br>• Are extracts from the original data included?<br>• Are the data appropriately referenced?<br>• Is the reporting clear and coherent?	Convincing ✓<br>Not convincing<br>Not sure	Comments: The findings aren't really 'findings' as such. It is a discussion of implementing AEBCD. The information is convincing in terms of what we learn about the challenges and facilitators of using AEBCD
**12. Are the findings relevant to the aims of the study?**	Relevant ✓<br>Irrelevant<br>Partially relevant	Comments:
**13. Conclusions**<br>For example:<br>• How clear are the links between data, interpretation and conclusions?<br>• Are the conclusions plausible and coherent?<br>• Have alternative explanations been explored and discounted?<br>• Does this enhance understanding of the research topic?	Adequate ✓<br>Inadequate<br>Not sure	Comments:

For peer review only

• Are the implications of the research clearly defined?<br>**Is there adequate discussion of any limitations encountered?**		
**Ethics**		
**14. How clear and coherent is the reporting of ethics?**<br>For example:<br>• Have ethical issues been taken into consideration?<br>• Are they adequately discussed e.g. do they address consent and anonymity?<br>• Have the consequences of the research been considered i.e. raising expectations, changing behaviour?<br>• Was the study approved by an ethics committee?	Appropriate<br>Inappropriate<br>Not sure/not reported ✓	Comments:
**Overall assessment**		
**As far as can be ascertained from the paper, how well was the study conducted? (see guidance notes)**	++<br>+ ✓<br>-	Comments: Reports on facilitation of the Locock et al (2014) study. Provides a different perspective in focusing on the author's experiences and reflections on facilitating an EBCD process. The paper does not report the research aspects of the Locock et al study.

For peer review only

**Study identification:** Include author, title, reference, year of publication	Tollyfield, R. 2014 Facilitating an accelerated experience-based co-design project. British Journal of Nursing, 23, (3):136-141	
**Guidance topic:** Co-production in acute healthcare settings.	**Key research question/aim:** Aims of the paper are stated but not the aims of the actual study.	
**Checklist completed by:**	DJC	
**Theoretical approach:** No specific theoretical framework was reported		
**1. Is a qualitative approach appropriate?**<br>For example:<br>• Does the research question seek to understand processes or structures, or illuminate subjective experiences or meanings?<br>• Could a quantitative approach better have addressed the research question?	Appropriate<br>Inappropriate<br>Not sure ✓	Comments: This is a personal reflection and description of facilitating an AEBCD project. The value in the CREATE RES is that the paper directly addresses the barriers and facilitators to using AEBCD/EBCD in acute healthcare settings.
**2. Is the study clear in what it seeks to do?**<br>For example:<br>• Is the purpose of the study discussed – aims/objectives/research question/s?<br>• Is there adequate/appropriate reference to the literature?	Clear<br>Unclear<br>Mixed ✓	Comments: The study in which Tollyfield acted as AEBCD facilitator is clear but the research conducted is not the focus of this paper.

For peer review only

• Are underpinning values/assumptions/theory discussed?		
**Study design**		
**3. How defensible/rigorous is the research design/methodology?**<br>For example:<br>• Is the design appropriate to the research question?<br>• Is a rationale given for using a qualitative approach?<br>• Are there clear accounts of the rationale/justification for the sampling, data collection and data analysis techniques used?<br>• Is the selection of cases/sampling strategy theoretically justified?	Defensible<br>Indefensible<br>Not sure ✓	Comments:
**Data collection**		
**4. How well was the data collection carried out?**<br>For example:<br>• Are the data collection methods clearly described?	Appropriately<br>Inappropriately<br>Not sure/inadequately	Comments:

For peer review only

• Were the appropriate data collected to address the research question?<br>• Was the data collection and record keeping systematic?	reported ✓	
**Trustworthiness**		
**5. Is the role of the researcher clearly described?**<br>For example:<br>• Has the relationship between the researcher and the participants been adequately considered?<br>• Does the paper describe how the research was explained and presented to the participants?	Clearly described<br>Unclear ✓<br>Not described	Comments:
**6. Is the context clearly described?**<br>For example:<br>• Are the characteristics of the participants and settings clearly defined?<br>• Were observations made in a sufficient variety of circumstances	Clear<br>Unclear<br>Not sure ✓	Comments:

For peer review only

• Was context bias considered		
**7. Were the methods reliable?**<br>For example:<br>• Was data collected by more than 1 method?<br>• Is there justification for triangulation, or for not triangulating?<br>• Do the methods investigate what they claim to?	Reliable<br>Unreliable<br>Not sure ✓	Comments:
**Analysis**		
**8. Is the data analysis sufficiently rigorous?**<br>For example:<br>• Is the procedure explicit – i.e. is it clear how the data was analysed to arrive at the results?<br>• How systematic is the analysis, is the procedure reliable/dependable?<br>• Is it clear how the themes and concepts were derived from the data?	Rigorous<br>Not rigorous<br>Not sure/not reported ✓	Comments:
**9. Is the data 'rich'?**	Rich	Comments: Provides a range of information about facilitating AEBCD/EBCD projects in

For peer review only

For example:<br>• How well are the contexts of the data described?<br>• Has the diversity of perspective and content been explored?<br>• How well has the detail and depth been demonstrated?<br>• Are responses compared and contrasted across groups/sites?	Poor<br>Not sure/not reported ✓	practice.
**10. Is the analysis reliable?**<br>For example:<br>• Did more than 1 researcher theme and code transcripts/data?<br>• If so, how were differences resolved?<br>• Did participants feed back on the transcripts/data if possible and relevant?<br>• Were negative/discrepant results addressed or ignored?	Reliable<br>Unreliable<br>Not sure/not reported ✓	Comments:
**11. Are the findings convincing?**<br>For example:<br>• Are the findings clearly presented?	Convincing ✓<br>Not convincing<br>Not sure	Comments: Does not report research findings per se but rather the authors experiences in facilitating an AEBCD project.

For peer review only

• Are the findings internally coherent?<br>• Are extracts from the original data included?<br>• Are the data appropriately referenced?<br>• Is the reporting clear and coherent?		
**12. Are the findings relevant to the aims of the study?**	Relevant ✓<br>Irrelevant<br>Partially relevant	Comments: Very useful information on barriers and facilitators to AEBCD/EBCD in an acute care setting.
**13. Conclusions**<br>For example:<br>• How clear are the links between data, interpretation and conclusions?<br>• Are the conclusions plausible and coherent?<br>• Have alternative explanations been explored and discounted?<br>• Does this enhance understanding of the research topic?<br>• Are the implications of the research clearly defined?<br>**Is there adequate discussion of any limitations**	Adequate ✓<br>Inadequate<br>Not sure	Comments:

For peer review only

**encountered?**		
**Ethics**		
**14. How clear and coherent is the reporting of ethics?**<br>For example:<br>• Have ethical issues been taken into consideration?<br>• Are they adequately discussed e.g. do they address consent and anonymity?<br>• Have the consequences of the research been considered i.e. raising expectations, changing behaviour?<br>• Was the study approved by an ethics committee?	Appropriate<br>Inappropriate<br>Not sure/not reported ✓	Comments:
**Overall assessment**		
**As far as can be ascertained from the paper, how well was the study conducted? (see guidance notes)**	++<br>+ ✓<br>-	Comments: Reports on facilitation of the Locock et al (2014) study but focuses on Tollyfield's experiences and reflections in facilitating an EBCD process.

For peer review only

**Study identification:** Include author, title, reference, year of publication	Tsianakas,V et al, 2012 Implementing patient-centred cancer care: using experience-based co-design to improve patient experience in breast and lung cancer services. Supportive Care in Cancer (2012) 20:2639–2647	
**Guidance topic:** Co-production in acute healthcare settings.	**Key research question/aim:** The aims of this paper are to describe the process by which patients and staff in these two services identified and implemented improvements in patient experience before (1) comparing the issues identified as shaping patient experiences in the different tumour groups and (2) exploring participants' reflections on the value and key characteristics of this approach to improving patient experiences.	
**Checklist completed by:**	RH	
**Theoretical approach:** No specific theoretical framework was reported		
**1. Is a qualitative approach appropriate?**<br>For example:<br>• Does the research question seek to understand processes or structures, or illuminate subjective experiences or meanings?<br>• Could a quantitative approach better have addressed the research question?	Appropriate ✓<br>Inappropriate<br>Not sure	Comments:
**2. Is the study clear in what it seeks to do?**<br>For example:<br>• Is the purpose of the study discussed – aims/objectives/research question/s?	Clear✓<br>Unclear<br>Mixed	Comments:

For peer review only

• Is there adequate/appropriate reference to the literature?<br>• Are underpinning values/assumptions/theory discussed?		
**Study design**		
**3. How defensible/rigorous is the research design/methodology?**<br>For example:<br>• Is the design appropriate to the research question?<br>• Is a rationale given for using a qualitative approach?<br>• Are there clear accounts of the rationale/justification for the sampling, data collection and data analysis techniques used?<br>• Is the selection of cases/sampling strategy theoretically justified?	Defensible ✓<br>Indefensible<br>Not sure	Comments:
**Data collection**		
**4. How well was the data collection carried out?**<br>For example:<br>• Are the data collection methods clearly described?<br>• Were the appropriate data collected to address the	Appropriately ✓<br>Inappropriately<br>Not sure/inadequately reported	Comments:

For peer review only

research question?<br>• Was the data collection and record keeping systematic?		
**Trustworthiness**		
**5. Is the role of the researcher clearly described?**<br>For example:<br>• Has the relationship between the researcher and the participants been adequately considered?<br>• Does the paper describe how the research was explained and presented to the participants?	Clearly described✓<br>Unclear<br>Not described	Comments:
**6. Is the context clearly described?**<br>For example:<br>• Are the characteristics of the participants and settings clearly defined?<br>• Were observations made in a sufficient variety of circumstances<br>• Was context bias considered	Clear<br>Unclear✓<br>Not sure	Comments:

For peer review only

**7. Were the methods reliable?**<br>For example:<br>• Was data collected by more than 1 method?<br>• Is there justification for triangulation, or for not triangulating?<br>• Do the methods investigate what they claim to?	Reliable ✓<br>Unreliable<br>Not sure	Comments:
**Analysis**		
**8. Is the data analysis sufficiently rigorous?**<br>For example:<br>• Is the procedure explicit – i.e. is it clear how the data was analysed to arrive at the results?<br>• How systematic is the analysis, is the procedure reliable/dependable?<br>• Is it clear how the themes and concepts were derived from the data?	Rigorous ✓<br>Not rigorous<br>Not sure/not reported	Comments:
**9. Is the data 'rich'?**<br>For example:<br>• How well are the contexts of the data described?<br>• Has the diversity of perspective and content been explored?	Rich ✓<br>Poor<br>Not sure/not reported	Comments:

For peer review only

• How well has the detail and depth been demonstrated?<br>• Are responses compared and contrasted across groups/sites?		
**10. Is the analysis reliable?**<br>For example:<br>• Did more than 1 researcher theme and code transcripts/data?<br>• If so, how were differences resolved?<br>• Did participants feed back on the transcripts/data if possible and relevant?<br>• Were negative/discrepant results addressed or ignored?	Reliable ✓<br>Unreliable<br>Not sure/not reported	Comments:
**11. Are the findings convincing?**<br>For example:<br>• Are the findings clearly presented?<br>• Are the findings internally coherent?<br>• Are extracts from the original data included?<br>• Are the data appropriately referenced?<br>• Is the reporting clear and coherent?	Convincing ✓<br>Not convincing<br>Not sure	Comments:

For peer review only

**12. Are the findings relevant to the aims of the study?**	Relevant ✓<br>Irrelevant<br>Partially relevant	Comments:
**13. Conclusions**<br>For example:<br>• How clear are the links between data, interpretation and conclusions?<br>• Are the conclusions plausible and coherent?<br>• Have alternative explanations been explored and discounted?<br>• Does this enhance understanding of the research topic?<br>• Are the implications of the research clearly defined?<br>**Is there adequate discussion of any limitations encountered?**	Adequate ✓<br>Inadequate<br>Not sure	Comments:
**Ethics**		
**14. How clear and coherent is the reporting of ethics?**<br>For example:<br>• Have ethical issues been taken into consideration?<br>• Are they adequately discussed e.g. do they address consent and anonymity?	Appropriate<br>Inappropriate<br>Not sure/not reported ✓	Comments: Not addressed in the paper

For peer review only

• Have the consequences of the research been considered i.e. raising expectations, changing behaviour?<br>• Was the study approved by an ethics committee?		
**Overall assessment**		
**As far as can be ascertained from the paper, how well was the study conducted? (see guidance notes)**	++ ✓<br>+<br>−	Comments:

**Study identification:** Include author, title, reference, year of publication	Tsianakas,V et al, 2012 Implementing patient-centred cancer care: using experience-based co-design to improve patient experience in breast and lung cancer services. Supportive Care in Cancer (2012) 20:2639–2647
**Guidance topic:** Co-production in acute healthcare settings.	**Key research question/aim:**<br><br>To use EBCD to enhance experiences for breast and lung cancer service patients; paper briefly reports on the process which led to service improvement s. But main questions focus on comparing issues identified as shaping experience in the different tumour groups 2) exploring participants' reflections on the value and key characteristics of the approach to improving patient experiences
**Checklist completed by:**	FJ
**Theoretical approach:** evaluation of existing EBCD improvement projects; no other details about theoretical approach	

For peer review only

**1. Is a qualitative approach appropriate?**<br>For example:<br>• Does the research question seek to understand processes or structures, or illuminate subjective experiences or meanings?<br>• Could a quantitative approach better have addressed the research question?	Appropriate ✓<br>Inappropriate<br>Not sure	Comments: qualitative approach part of EBCD (observations and interviews)
**2. Is the study clear in what it seeks to do?**<br>For example:<br>• Is the purpose of the study discussed – aims/objectives/research question/s?<br>• Is there adequate/appropriate reference to the literature?<br>• Are underpinning values/assumptions/theory discussed?	Clear ✓<br>Unclear<br>Mixed	Comments:
**Study design**		
**3. How defensible/rigorous is the research design/methodology?**<br>For example:<br>• Is the design appropriate to the	Defensible<br>Indefensible<br>Not sure ✓	Comments:<br>Not much detail about sampling. Selection of cases not justified.

For peer review only

research question?<br>• Is a rationale given for using a qualitative approach?<br>• Are there clear accounts of the rationale/justification for the sampling, data collection and data analysis techniques used?<br>• Is the selection of cases/sampling strategy theoretically justified?		
**Data collection**		
**4. How well was the data collection carried out?**<br>For example:<br>• Are the data collection methods clearly described?<br>• Were the appropriate data collected to address the research question?<br>• Was the data collection and record keeping systematic?	Appropriately ✓<br>Inappropriately<br>Not sure/inadequately reported	Comments:
**Trustworthiness**		

For peer review only

**5. Is the role of the researcher clearly described?**<br>For example:<br>• Has the relationship between the researcher and the participants been adequately considered?<br>• Does the paper describe how the research was explained and presented to the participants?	Clearly described<br>Unclear ✓<br>Not described	Comments: two researchers involved in viewing films independently and sharing understanding of touchpoints; 2 researchers (including 1 different researcher) developed composite films. All 3 researchers were involved with observations, interviews and analysis in some way - but relationship and explanations not given
**6. Is the context clearly described?**<br>For example:<br>• Are the characteristics of the participants and settings clearly defined?<br>• Were observations made in a sufficient variety of circumstances<br>• Was context bias considered	Clear ✓<br>Unclear<br>Not sure	Comments: no comment on content bias
**7. Were the methods reliable?**<br>For example:<br>• Was data collected by more than 1 method?	Reliable ✓<br>Unreliable<br>Not sure	Comments:

For peer review only

• Is there justification for triangulation, or for not triangulating?<br>• Do the methods investigate what they claim to?		
**Analysis**		
**8. Is the data analysis sufficiently rigorous?**<br>For example:<br>• Is the procedure explicit – i.e. is it clear how the data was analysed to arrive at the results?<br>• How systematic is the analysis, is the procedure reliable/dependable?<br>• Is it clear how the themes and concepts were derived from the data?	Rigorous<br>Not rigorous<br>Not sure/not ✓reported	Comments:<br>Not clear how observational data were managed and analysed
**9. Is the data 'rich'?**<br>For example:<br>• How well are the contexts of the data described?<br>• Has the diversity of perspective and content been explored?<br>• How well has the detail and depth been	Rich ✓<br>Poor<br>Not sure/not reported	Comments:

For peer review only

demonstrated?<br>• Are responses compared and contrasted across groups/sites?		
**10. Is the analysis reliable?**<br>For example:<br>• Did more than 1 researcher theme and code transcripts/data?<br>• If so, how were differences resolved?<br>• Did participants feed back on the transcripts/data if possible and relevant?<br>• Were negative/discrepant results addressed or ignored?	Reliable<br>Unreliable<br>Not sure/not reported ✓	Comments: no comment about how differences were resolved in thematic analysis ; participant feedback was an implicit part of the staff, patient groups
**11. Are the findings convincing?**<br>For example:<br>• Are the findings clearly presented?<br>• Are the findings internally coherent?<br>• Are extracts from the original data included?<br>• Are the data appropriately referenced?	Convincing ✓<br>Not convincing<br>Not sure	Comments:<br>Finding reported clearly but as a commentary rather than using a traditional way with themes/sub-themes –illustrative quotes etc.

For peer review only

• Is the reporting clear and coherent?		
**12. Are the findings relevant to the aims of the study?**	Relevant ✓<br>Irrelevant<br>Partially relevant	Comments:
**13. Conclusions**<br>For example:<br>• How clear are the links between data, interpretation and conclusions?<br>• Are the conclusions plausible and coherent?<br>• Have alternative explanations been explored and discounted?<br>• Does this enhance understanding of the research topic?<br>• Are the implications of the research clearly defined?<br>**Is there adequate discussion of any limitations encountered?**	Adequate ✓<br>Inadequate<br>Not sure	Comments:
**Ethics**		
**14. How clear and coherent is the reporting**	Appropriate	Comments:

For peer review only

**of ethics?**<br><br>For example:<br><br>• Have ethical issues been taken into consideration?<br><br>• Are they adequately discussed e.g. do they address consent and anonymity?<br><br>• Have the consequences of the research been considered i.e. raising expectations, changing behaviour?<br><br>• Was the study approved by an ethics committee?	Inappropriate<br><br>Not sure/not reported ✓	No mention of ethical approval
**Overall assessment**		
**As far as can be ascertained from the paper, how well was the study conducted? (see guidance notes)**	+<br><br>Following independent third review agreed to be ++	Comments:

For peer review only

***For this study which adopted a mixed methods approach, the CASP RCT checklist was used as well as the NICE (2012) qualitative research approval tool.** See http://media.wix.com/ugd/dded87_40b9ff0bf53840478331915a8ed8b2fb.pdf
The RCT was small scale and exploratory, nonetheless we regarded the quality, rigour and reporting of the study to be satisfactory to good.

**Study identification:** Include author, title, reference, year of publication	Enhancing the experience of carers in the chemotherapy outpatient setting: an exploratory randomised controlled trial to test impact, acceptability and feasibility of a complex intervention co-designed by carers and staff V. Tsianakas & G. Robert & A. Richardson & R. Verity & C. Oakley & T. Murrells & M. Flynn & E. Ream. Support Care Cancer. 2015. DOI 10.1007/s00520-015-2677-x	
**Guidance topic:**	**Key research question/aim:** To test the feasibility and acceptability of a complex intervention for carers that was co-designed by staff and carers of patients starting chemotherapy.	
**Checklist completed by:**	SH	
**Theoretical approach**		
**1. Is a qualitative approach appropriate?**<br><br>For example:<br><br>• Does the research question seek to understand processes or structures, or illuminate subjective experiences or meanings?<br><br>• Could a quantitative approach better have addressed the research question?	Appropriate ✓<br><br>Inappropriate<br><br>Not sure	Comments: Appropriate as the study seeks to elicit the subjective views and meanings of carers and staff. A quantitative approach could not accomplish this.
**2. Is the study clear in what it seeks to do?**<br><br>For example:	Clear ✓<br><br>Unclear	Comments: The aims are discussed and are clear. There is adequate reference to the literature and underpinning theories, including international studies examining the information needs of cancer

For peer review only

• Is the purpose of the study discussed – aims/objectives/research question/s?<br>• Is there adequate/appropriate reference to the literature?<br>• Are underpinning values/assumptions/theory discussed?	Mixed	patients.
**Study design**		
**3. How defensible/rigorous is the research design/methodology?**<br>For example:<br>• Is the design appropriate to the research question?<br>• Is a rationale given for using a qualitative approach?<br>• Are there clear accounts of the rationale/justification for the sampling, data collection and data analysis techniques used?<br>• Is the selection of cases/sampling strategy theoretically justified?	Defensible ✓<br>Indefensible<br>Not sure	Comments: The design is appropriate for a feasibility and acceptability study. The rationale for the sampling and data collection is discussed. Carers of patients with lung, breast and colon cancer were chosen as they are among the most common cancers in the UK.
**Data collection**		
**4. How well was the data collection carried out?**<br>For example:	Appropriately ✓<br>Inappropriately	Comments: the focus groups are clearly described and were appropriate for the research question.

For peer review only

• Are the data collection methods clearly described?<br>• Were the appropriate data collected to address the research question?<br>• Was the data collection and record keeping systematic?	Not sure/inadequately reported	
**Trustworthiness**		
**5. Is the role of the researcher clearly described?**<br>For example:<br>• Has the relationship between the researcher and the participants been adequately considered?<br>• Does the paper describe how the research was explained and presented to the participants?	Clearly described<br>Unclear ✓<br>Not described	Comments: Not described in detail.
**6. Is the context clearly described?**<br>For example:<br>• Are the characteristics of the participants and settings clearly defined?<br>• Were observations made in a sufficient variety of circumstances<br>• Was context bias considered	Clear ✓<br>Unclear<br>Not sure	Comments: The outpatient setting was clearly described.

For peer review only

**7. Were the methods reliable?**<br><br>For example:<br><br>• Was data collected by more than 1 method?<br><br>• Is there justification for triangulation, or for not triangulating?<br><br>• Do the methods investigate what they claim to?	Reliable ✓<br><br>Unreliable<br><br>Not sure	Comments: The study described here is the qualitative part of a feasibility trial that included questionnaires and focus groups. The methods do investigate what they claim to.
**Analysis**		
**8. Is the data analysis sufficiently rigorous?**<br><br>For example:<br><br>• Is the procedure explicit – i.e. is it clear how the data was analysed to arrive at the results?<br><br>• How systematic is the analysis, is the procedure reliable/dependable?<br><br>• Is it clear how the themes and concepts were derived from the data?	Rigorous<br><br>Not rigorous<br><br>Not sure/not reported ✓	Comments: The method of analysis is only briefly described without sufficient detail to make a judgement about its rigour.
**9. Is the data 'rich'?**<br><br>For example:<br><br>• How well are the contexts of the data described?<br><br>• Has the diversity of perspective and content been explored?<br><br>• How well has the detail and depth been	Rich ✓<br><br>Poor<br><br>Not sure/not reported	Comments: the data is 'rich' in that it was exploring the acceptably of an intervention as part of a small feasibility study i.e. it set out what it aimed to do within the confines of the study.

For peer review only

demonstrated?<br>• Are responses compared and contrasted across groups/sites?		
**10. Is the analysis reliable?**<br>For example:<br>• Did more than 1 researcher theme and code transcripts/data?<br>• If so, how were differences resolved?<br>• Did participants feed back on the transcripts/data if possible and relevant?<br>• Were negative/discrepant results addressed or ignored?	Reliable<br>Unreliable<br>Not sure/not reported ✓	Comments: The qualitative analysis was not reported in any detail.
**11. Are the findings convincing?**<br>For example:<br>• Are the findings clearly presented?<br>• Are the findings internally coherent?<br>• Are extracts from the original data included?<br>• Are the data appropriately referenced?<br>• Is the reporting clear and coherent?	Convincing ✓<br>Not convincing<br>Not sure	Comments: The findings are convincing in the context of a small feasibility study. The weaknesses are cited as the small size and the fact that the study was conducted at only one centre.
**12. Are the findings relevant to the aims of the study?**	Relevant ✓	Comments: Yes the findings indicate that the intervention was acceptable.

For peer review only

	Irrelevant<br><br>Partially relevant	
**13. Conclusions**<br><br>For example:<br><br>• How clear are the links between data, interpretation and conclusions?<br><br>• Are the conclusions plausible and coherent?<br><br>• Have alternative explanations been explored and discounted?<br><br>• Does this enhance understanding of the research topic?<br><br>• Are the implications of the research clearly defined?<br><br>**Is there adequate discussion of any limitations encountered?**	Adequate ✓<br><br>Inadequate<br><br>Not sure	Comments: the conclusions are plausible and indicate further research would be valuable.<br><br>The weaknesses are cited as the small size and the fact that the study was conducted at only one centre.
**Ethics**		
**14. How clear and coherent is the reporting of ethics?**<br><br>For example:<br><br>• Have ethical issues been taken into consideration?<br><br>• Are they adequately discussed e.g. do they address consent and anonymity?	Appropriate<br><br>Inappropriate<br><br>Not sure/not reported ✓	Comments: The study had ethical approval. No other details were reported.

For peer review only

• Have the consequences of the research been considered i.e. raising expectations, changing behaviour?<br>• Was the study approved by an ethics committee?		
**Overall assessment**		
**As far as can be ascertained from the paper, how well was the study conducted? (see guidance notes)**	++ ✓<br>+<br>−	Comments:

**Study identification:** Include author, title, reference, year of publication	Tsianakas,V. Robert,G. Richardson,A., Verity,R., Oakley,C. Murrells,T., Flynn,M., Ream,E. 2015 Enhancing the experience of carers in the chemotherapy outpatient setting: an exploratory randomised controlled trial to test impact, acceptability and feasibility of a complex intervention co-designed by carers and staff Ream. Support Care Cancer. DOI 10.1007/s00520-015-2677-x
**Guidance topic:**	**Key research question/aim:** To test the feasibility and acceptability of a complex intervention for carers that was co-designed by staff and carers of patients starting chemotherapy.
**Checklist completed by:**	DJC
**Theoretical approach:** Not reported, though locates the feasibility RCT in the context of the MRC guidance for development of complex interventions.	

For peer review only

**1. Is a qualitative approach appropriate?**<br><br>For example:<br><br>• Does the research question seek to understand processes or structures, or illuminate subjective experiences or meanings?<br><br>• Could a quantitative approach better have addressed the research question?	Appropriate ✓<br><br>Inappropriate<br><br>Not sure	Comments:
**2. Is the study clear in what it seeks to do?**<br><br>For example:<br><br>• Is the purpose of the study discussed – aims/objectives/research question/s?<br><br>• Is there adequate/appropriate reference to the literature?<br><br>• Are underpinning values/assumptions/theory discussed?	Clear ✓<br><br>Unclear<br><br>Mixed	Comments:
**Study design**		
**3. How defensible/rigorous is the research design/methodology?**<br><br>For example:<br><br>• Is the design appropriate to the research question?<br><br>• Is a rationale given for using a qualitative	Defensible ✓<br><br>Indefensible<br><br>Not sure	Comments:

For peer review only

approach?<br>• Are there clear accounts of the rationale/justification for the sampling, data collection and data analysis techniques used?<br>• Is the selection of cases/sampling strategy theoretically justified?		
**Data collection**		
**4. How well was the data collection carried out?**<br>For example:<br>• Are the data collection methods clearly described?<br>• Were the appropriate data collected to address the research question?<br>• Was the data collection and record keeping systematic?	Appropriately ✓<br>Inappropriately<br>Not sure/inadequately reported	Comments:
**Trustworthiness**		
**5. Is the role of the researcher clearly described?**<br>For example:<br>• Has the relationship between the researcher and the participants been adequately considered?<br>• Does the paper describe how the research was	Clearly described<br>Unclear ✓<br>Not described	Comments: Not described.

For peer review only

explained and presented to the participants?		
**6. Is the context clearly described?**<br><br>For example:<br><br>• Are the characteristics of the participants and settings clearly defined?<br><br>• Were observations made in a sufficient variety of circumstances<br><br>• Was context bias considered	Clear ✓<br><br>Unclear<br><br>Not sure	Comments:
**7. Were the methods reliable?**<br><br>For example:<br><br>• Was data collected by more than 1 method?<br><br>• Is there justification for triangulation, or for not triangulating?<br><br>• Do the methods investigate what they claim to?	Reliable ✓<br><br>Unreliable<br><br>Not sure	Comments: The study described is the qualitative part of a feasibility trial that included questionnaires and focus groups.
**Analysis**		
**8. Is the data analysis sufficiently rigorous?**<br><br>For example:<br><br>• Is the procedure explicit – i.e. is it clear how the data was analysed to arrive at the results?<br><br>• How systematic is the analysis, is the procedure	Rigorous<br><br>Not rigorous<br><br>Not sure/not reported ✓	Comments:

For peer review only

reliable/dependable?<br>• Is it clear how the themes and concepts were derived from the data?		
**9. Is the data 'rich'?**<br>For example:<br>• How well are the contexts of the data described?<br>• Has the diversity of perspective and content been explored?<br>• How well has the detail and depth been demonstrated?<br>• Are responses compared and contrasted across groups/sites?	Rich ✓<br>Poor<br>Not sure/not reported	Comments
**10. Is the analysis reliable?**<br>For example:<br>• Did more than 1 researcher theme and code transcripts/data?<br>• If so, how were differences resolved?<br>• Did participants feed back on the transcripts/data if possible and relevant?<br>• Were negative/discrepant results addressed or ignored?	Reliable<br>Unreliable<br>Not sure/not reported ✓	Comments: Very limited reporting of analysis.

For peer review only

**11. Are the findings convincing?**<br><br>For example:<br>• Are the findings clearly presented?<br>• Are the findings internally coherent?<br>• Are extracts from the original data included?<br>• Are the data appropriately referenced?<br>• Is the reporting clear and coherent?	Convincing ✓<br><br>Not convincing<br><br>Not sure	Comments:
**12. Are the findings relevant to the aims of the study?**	Relevant ✓<br><br>Irrelevant<br><br>Partially relevant	Comments:
**13. Conclusions**<br><br>For example:<br>• How clear are the links between data, interpretation and conclusions?<br>• Are the conclusions plausible and coherent?<br>• Have alternative explanations been explored and discounted?<br>• Does this enhance understanding of the research topic?<br>• Are the implications of the research clearly defined?	Adequate ✓<br><br>Inadequate<br><br>Not sure	Comments:

For peer review only

**Is there adequate discussion of any limitations encountered?**		
**Ethics**		
**14. How clear and coherent is the reporting of ethics?**<br><br>For example:<br><br>• Have ethical issues been taken into consideration?<br><br>• Are they adequately discussed e.g. do they address consent and anonymity?<br><br>• Have the consequences of the research been considered i.e. raising expectations, changing behaviour?<br><br>• Was the study approved by an ethics committee?	Appropriate<br><br>Inappropriate<br><br>Not sure/not reported ✓	Comments: Ethical approval is reported.
**Overall assessment**		
**As far as can be ascertained from the paper, how well was the study conducted? (see guidance notes)**	++ ✓<br><br>+<br><br>−	Comments:

For peer review only

**Study identification:** Include author, title, reference, year of publication	Vennik et al, Co-production in healthcare: rhetoric and practice. International Review of Administrative Sciences. 82(1) 150–168	
**Guidance topic:** Co-production in acute healthcare settings.	**Key research question/aim:** Why are hospitals interested in co-production activities and what are the experiences with co-production in practice?	
**Checklist completed by:**	RH	
**Theoretical approach:** No specific theoretical framework was reported		
**1. Is a qualitative approach appropriate?**<br>For example:<br>• Does the research question seek to understand processes or structures, or illuminate subjective experiences or meanings?<br>• Could a quantitative approach better have addressed the research question?	Appropriate ✓<br>Inappropriate<br>Not sure	Comments:
**2. Is the study clear in what it seeks to do?**<br>For example:<br>• Is the purpose of the study discussed – aims/objectives/research question/s?<br>• Is there adequate/appropriate reference to the literature?<br>• Are underpinning values/assumptions/theory discussed?	Clear✓<br>Unclear<br>Mixed	Comments:

For peer review only

**Study design**		
**3. How defensible/rigorous is the research design/methodology?**<br>For example:<br>• Is the design appropriate to the research question?<br>• Is a rationale given for using a qualitative approach?<br>• Are there clear accounts of the rationale/justification for the sampling, data collection and data analysis techniques used?<br>• Is the selection of cases/sampling strategy theoretically justified?	Defensible ✓<br>Indefensible<br>Not sure	Comments:
**Data collection**		
**4. How well was the data collection carried out?**<br>For example:<br>• Are the data collection methods clearly described?<br>• Were the appropriate data collected to address the research question?<br>• Was the data collection and record keeping systematic?	Appropriately ✓<br>Inappropriately<br>Not sure/inadequately reported	Comments:
**Trustworthiness**		

For peer review only

**5. Is the role of the researcher clearly described?**<br>For example:<br>• Has the relationship between the researcher and the participants been adequately considered?<br>• Does the paper describe how the research was explained and presented to the participants?	Clearly described✓<br>Unclear<br>Not described	Comments:
**6. Is the context clearly described?**<br>For example:<br>• Are the characteristics of the participants and settings clearly defined?<br>• Were observations made in a sufficient variety of circumstances<br>• Was context bias considered	Clear ✓<br>Unclear<br>Not sure	Comments:
**7. Were the methods reliable?**<br>For example:<br>• Was data collected by more than 1 method?<br>• Is there justification for triangulation, or for not triangulating?<br>• Do the methods investigate what they claim to?	Reliable ✓<br>Unreliable<br>Not sure	Comments:

For peer review only

Analysis		
**8. Is the data analysis sufficiently rigorous?**<br>For example:<br>• Is the procedure explicit – i.e. is it clear how the data was analysed to arrive at the results?<br>• How systematic is the analysis, is the procedure reliable/dependable?<br>• Is it clear how the themes and concepts were derived from the data?	Rigorous ✓<br>Not rigorous<br>Not sure/not reported	Comments:
**9. Is the data 'rich'?**<br>For example:<br>• How well are the contexts of the data described?<br>• Has the diversity of perspective and content been explored?<br>• How well has the detail and depth been demonstrated?<br>• Are responses compared and contrasted across groups/sites?	Rich ✓<br>Poor<br>Not sure/not reported	Comments:
**10. Is the analysis reliable?**<br>For example:<br>• Did more than 1 researcher theme and code transcripts/data?<br>• If so, how were differences resolved?<br>• Did participants feed back on the transcripts/data if possible	Reliable ✓<br>Unreliable<br>Not sure/not reported	Comments:

For peer review only

and relevant?<br>• Were negative/discrepant results addressed or ignored?		
**11. Are the findings convincing?**<br>For example:<br>• Are the findings clearly presented?<br>• Are the findings internally coherent?<br>• Are extracts from the original data included?<br>• Are the data appropriately referenced?<br>• Is the reporting clear and coherent?	Convincing ✓<br>Not convincing<br>Not sure	Comments:
**12. Are the findings relevant to the aims of the study?**	Relevant ✓<br>Irrelevant<br>Partially relevant	Comments:
**13. Conclusions**<br>For example:<br>• How clear are the links between data, interpretation and conclusions?<br>• Are the conclusions plausible and coherent?<br>• Have alternative explanations been explored and discounted?<br>• Does this enhance understanding of the research topic?	Adequate ✓<br>Inadequate<br>Not sure	Comments:

For peer review only

• Are the implications of the research clearly defined?<br>**Is there adequate discussion of any limitations encountered?**		
**Ethics**		
**14. How clear and coherent is the reporting of ethics?**<br>For example:<br>• Have ethical issues been taken into consideration?<br>• Are they adequately discussed e.g. do they address consent and anonymity?<br>• Have the consequences of the research been considered i.e. raising expectations, changing behaviour?<br>• Was the study approved by an ethics committee?	Appropriate<br>Inappropriate<br>Not sure/not reported ✓	Comments: Not addressed in the paper
**Overall assessment**		
**As far as can be ascertained from the paper, how well was the study conducted? (see guidance notes)**	++ ✓<br>+<br>−	Comments:

For peer review only

**Study identification:** Include author, title, reference, year of publication	Vennik et al, 2016 Co-production in healthcare: rhetoric and practice. International Review of Administrative Sciences, 82(1) 150–168	
**Guidance topic:**	**Key research question/aim:** See below, refers to the aim of the paper and not directly to the aim of the study directly. Does state: The article focuses on (research examining) hospitals involving patients and healthcare professionals in order to improve the quality of care on Dutch hospital wards.<br><br>The research question guiding this article is: Why are hospitals interested in co-production activities and what are the experiences with co-production in practice?	
**Checklist completed by:**	DJC	
**Theoretical approach:** Hospitals participating described as utilising co-production methods but the term co-design is also used. Four of five project teams indicated they would use EBCD methods but for various reasons decided to amend or remove some of the standard 6 stages in EBCD. Therefore, the approach is based on but not directly comparable with other EBCD projects. For example, patients were not always involved in the improvement and implementation process.		
**1. Is a qualitative approach appropriate?**<br><br>For example:<br><br>• Does the research question seek to understand processes or structures, or illuminate subjective experiences or meanings?<br><br>• Could a quantitative approach better have addressed the research question?	Appropriate ✓<br><br>Inappropriate<br><br>Not sure	Comments:<br><br>Describes a mixed methods qualitative study designed to understand why and how the 5 hospital project teams were involving patients in co-production projects designed to lead to service quality improvement. Use of observations, interviews (about process and outcomes in the projects) and documentary analysis provided for data triangulation.
**2. Is the study clear in what it seeks to do?**<br><br>For example:<br><br>• Is the purpose of the study discussed – aims/objectives/research question/s?	Clear ✓<br><br>Unclear<br><br>Mixed	Comments:<br><br>The paper reviews some of the co-production literature both in the wider public services arena and also in the more limited health services context.<br><br>There is an overview of the aim of the study and a single research

For peer review only

• Is there adequate/appropriate reference to the literature?<br>• Are underpinning values/assumptions/theory discussed?		question: *Why are hospitals interested in co-production activities and what are the experiences with co-production in practice?*<br>The research is a mixed methods approach to the above rather than an evaluation per se. The researcher's own assumptions and values are not discussed although the values and descriptive theory underpinning co-production is considered.
**Study design**		
**3. How defensible/rigorous is the research design/methodology?**<br>For example:<br>• Is the design appropriate to the research question?<br>• Is a rationale given for using a qualitative approach?<br>• Are there clear accounts of the rationale/justification for the sampling, data collection and data analysis techniques used?<br>• Is the selection of cases/sampling strategy theoretically justified?	Defensible √<br>Indefensible<br>Not sure	Comments:
**Data collection**		
**4. How well was the data collection carried out?**<br>For example:<br>• Are the data collection methods clearly	Appropriately √<br>Inappropriately	Comments:

For peer review only

described?<br>• Were the appropriate data collected to address the research question?<br>• Was the data collection and record keeping systematic?	Not sure/inadequately reported	
**Trustworthiness**		
**5. Is the role of the researcher clearly described?**<br>For example:<br>• Has the relationship between the researcher and the participants been adequately considered?<br>• Does the paper describe how the research was explained and presented to the participants?	Clearly described<br>Unclear √<br>Not described	Comments:
**6. Is the context clearly described?**<br>For example:<br>• Are the characteristics of the participants and settings clearly defined?<br>• Were observations made in a sufficient variety of circumstances<br>• Was context bias considered	Clear √<br>Unclear<br>Not sure	Comments:
**7. Were the methods reliable?**	Reliable √	Comments:

For peer review only

For example:<br>• Was data collected by more than 1 method?<br>• Is there justification for triangulation, or for not triangulating?<br>• Do the methods investigate what they claim to?	Unreliable<br>Not sure	
**Analysis**		
**8. Is the data analysis sufficiently rigorous?**<br>For example:<br>• Is the procedure explicit – i.e. is it clear how the data was analysed to arrive at the results?<br>• How systematic is the analysis, is the procedure reliable/dependable?<br>• Is it clear how the themes and concepts were derived from the data?	Rigorous ✓<br>Not rigorous<br>Not sure/not reported	Comments:
**9. Is the data 'rich'?**<br>For example:<br>• How well are the contexts of the data described?<br>• Has the diversity of perspective and content been explored?<br>• How well has the detail and depth been demonstrated?	Rich ✓<br>Poor<br>Not sure/not reported	Comments:

For peer review only

• Are responses compared and contrasted across groups/sites?		
**10. Is the analysis reliable?**<br><br>For example:<br><br>• Did more than 1 researcher theme and code transcripts/data?<br><br>• If so, how were differences resolved?<br><br>• Did participants feed back on the transcripts/data if possible and relevant?<br><br>• Were negative/discrepant results addressed or ignored?	Reliable ✓<br><br>Unreliable<br><br>Not sure/not reported	Comments:
**11. Are the findings convincing?**<br><br>For example:<br><br>• Are the findings clearly presented?<br><br>• Are the findings internally coherent?<br><br>• Are extracts from the original data included?<br><br>• Are the data appropriately referenced?<br><br>• Is the reporting clear and coherent?	Convincing ✓<br><br>Not convincing<br><br>Not sure	Comments:
**12. Are the findings relevant to the aims of the study?**	Relevant √<br><br>Irrelevant	Comments:

For peer review only

	Partially relevant	
**13. Conclusions**<br><br>For example:<br><br>• How clear are the links between data, interpretation and conclusions?<br><br>• Are the conclusions plausible and coherent?<br><br>• Have alternative explanations been explored and discounted?<br><br>• Does this enhance understanding of the research topic?<br><br>• Are the implications of the research clearly defined?<br><br>**Is there adequate discussion of any limitations encountered?**	Adequate √<br><br>Inadequate<br><br>Not sure	Comments:
**Ethics**		
**14. How clear and coherent is the reporting of ethics?**<br><br>For example:<br><br>• Have ethical issues been taken into consideration?<br><br>• Are they adequately discussed e.g. do they address consent and anonymity?<br><br>• Have the consequences of the research been	Appropriate<br><br>Inappropriate<br><br>Not sure/not reported √	Comments: There are no comments made about ethical review and approval. There is no discussion of consent or other ethical issues which may have arisen in relation to observations or interviews. Permission for recording of interviews and some observations is referred to briefly.

For peer review only

considered i.e. raising expectations, changing behaviour?<br><br>• Was the study approved by an ethics committee?		
**Overall assessment**		
**As far as can be ascertained from the paper, how well was the study conducted? (see guidance notes)**	++ √<br><br>+<br><br>–	Comments:

For peer review only

PRISMA 2009 Checklist

Section/topic	#	Checklist item	Reported on page #
**TITLE**			
Title	1	Identify the report as a systematic review, meta-analysis, or both.	4
**ABSTRACT**			
Structured summary	2	Provide a structured summary including, as applicable: background; objectives; data sources; study eligibility criteria, participants, and interventions; study appraisal and synthesis methods; results; limitations; conclusions and implications of key findings; systematic review registration number.	2
**INTRODUCTION**			
Rationale	3	Describe the rationale for the review in the context of what is already known.	3-4
Objectives	4	Provide an explicit statement of questions being addressed with reference to participants, interventions, comparisons, outcomes, and study design (PICOS).	4
**METHODS**			
Protocol and registration	5	Indicate if a review protocol exists, if and where it can be accessed (e.g., Web address), and, if available, provide registration information including registration number.	N/A
Eligibility criteria	6	Specify study characteristics (e.g., PICOS, length of follow-up) and report characteristics (e.g., years considered, language, publication status) used as criteria for eligibility, giving rationale.	5-6
Information sources	7	Describe all information sources (e.g., databases with dates of coverage, contact with study authors to identify additional studies) in the search and date last searched.	5
Search	8	Present full electronic search strategy for at least one database, including any limits used, such that it could be repeated.	Supp file1
Study selection	9	State the process for selecting studies (i.e., screening, eligibility, included in systematic review, and, if applicable, included in the meta-analysis).	6
Data collection process	10	Describe method of data extraction from reports (e.g., piloted forms, independently, in duplicate) and any processes for obtaining and confirming data from investigators.	6
Data items	11	List and define all variables for which data were sought (e.g., PICOS, funding sources) and any assumptions and simplifications made.	5-6
Risk of bias in individual studies	12	Describe methods used for assessing risk of bias of individual studies (including specification of whether this was done at the study or outcome level), and how this information is to be used in any data synthesis.	N/A
Summary measures	13	State the principal summary measures (e.g., risk ratio, difference in means).	N/A
Synthesis of results	14	Describe the methods of handling data and combining results of studies, if done, including measures of consistency (e.g., I^2) for each meta-analysis.	6

For peer review only

PRISMA 2009 Checklist

Section/topic	#	Checklist item	Reported on page #
Risk of bias across studies	15	Specify any assessment of risk of bias that may affect the cumulative evidence (e.g., publication bias, selective reporting within studies).	N/A
Additional analyses	16	Describe methods of additional analyses (e.g., sensitivity or subgroup analyses, meta-regression), if done, indicating which were pre-specified.	N/A
**RESULTS**			
Study selection	17	Give numbers of studies screened, assessed for eligibility, and included in the review, with reasons for exclusions at each stage, ideally with a flow diagram.	6 and 7
Study characteristics	18	For each study, present characteristics for which data were extracted (e.g., study size, PICOS, follow-up period) and provide the citations.	Supp file 2
Risk of bias within studies	19	Present data on risk of bias of each study and, if available, any outcome level assessment (see item 12).	N/A
Results of individual studies	20	For all outcomes considered (benefits or harms), present, for each study: (a) simple summary data for each intervention group (b) effect estimates and confidence intervals, ideally with a forest plot.	Supp file 2
Synthesis of results	21	Present results of each meta-analysis done, including confidence intervals and measures of consistency.	N/A
Risk of bias across studies	22	Present results of any assessment of risk of bias across studies (see Item 15).	N/A
Additional analysis	23	Give results of additional analyses, if done (e.g., sensitivity or subgroup analyses, meta-regression [see Item 16]).	N/A
**DISCUSSION**			
Summary of evidence	24	Summarize the main findings including the strength of evidence for each main outcome; consider their relevance to key groups (e.g., healthcare providers, users, and policy makers).	7-10 and Table 3
Limitations	25	Discuss limitations at study and outcome level (e.g., risk of bias), and at review-level (e.g., incomplete retrieval of identified research, reporting bias).	10-11
Conclusions	26	Provide a general interpretation of the results in the context of other evidence, and implications for future research.	
**FUNDING**			
Funding	27	Describe sources of funding for the systematic review and other support (e.g., supply of data); role of funders for the systematic review.	12

From: Moher D, Liberati A, Tetzlaff J, Altman DG, The PRISMA Group (2009). Preferred Reporting Items for Systematic Reviews and Meta-Analyses: The PRISMA Statement. PLoS Med 6(6): e1000097. doi:10.1371/journal.pmed1000097

For more information, visit: **www.prisma-statement.org**.

For peer review only - http://bmjopen.bmj.com/site/about/guidelines.xhtml
